# Hepatocellular carcinoma hosts cholinergic neural cells and tumoral hepatocytes harboring targetable muscarinic receptors^☆^

**DOI:** 10.1016/j.jhepr.2024.101245

**Published:** 2024-11-12

**Authors:** Charlotte A. Hernandez, Claire Verzeroli, Armando Andres Roca-Suarez, Abud-José Farca-Luna, Laurie Tonon, Roger Esteban-Fabró, Roser Pinyol, Marie-Laure Plissonnier, Ievgeniia Chicherova, Anaëlle Dubois, Pascale Bellaud, Marine Seffals, Bruno Turlin, Alain Fautrel, Gabriel Ichim, Michel Rivoire, Guillaume Passot, Zuzana Macek-Jilkova, Thomas Decaens, Alain Viari, Barbara Testoni, Sandra Rebouissou, Josep M. Llovet, Fabien Zoulim, Romain Parent

**Affiliations:** 1 Hepatitis Viruses and Pathobiology of Chronic Liver Diseases – LabEx DEVweCAN, Inserm U1052, Cancer Research Centre of Lyon – Hepatology Institute of Lyon F – IHU EVEREST, University of Lyon 1, ISPB, France, CNRS UMR5286, Centre Léon, Lyon, France; 2 Fondation Synergie Lyon Cancer, Gilles Thomas Bioinformatics Plateform, Centre Léon Bérard, F-69008 Lyon, France; 3 Translational Research in Hepatic Oncology Group, Liver Unit, Institut d'Investigacions Biomèdiques August Pi i Sunyer (IDIBAPS), Hospital Clínic, University of Barcelona, Barcelona, Catalonia, Spain; 4 Epigenetics, Microenvironment, and Liver Cancer, U1052, Cancer Research Centre of Lyon – Hepatology Institute of Lyon – IHU EVEREST, University of Lyon 1, ISPB, CNRS UMR5286, F-69083 Lyon, France, Centre Léon Bérard, Lyon, France; 5 H2P2 platform, University of Rennes, Rennes, France; 6 Cancer Cell Death team – LabEx DEVweCAN, Inserm U1052, Cancer Research Centre of Lyon, F-69003 Lyon, France, University of Lyon, F-69003 Lyon, University of Lyon 1, ISPB, Lyon, F-69622, France, CNRS UMR5286, F-69083 Lyon, France, Centre Léon Bérard, F-69008 Lyon, France; 7 Department of Surgical Oncology, Centre Léon Bérard, F-69008 Lyon, France; 8 Hospices Civils de Lyon, Service of Gastroenterology, F-69600 Oullins, France; 9 Institute for Advanced Biosciences, Inserm U1209, University of Grenoble-Alpes, F-38700 La Tronche, France; 10 Service d’hépato-Gastroentérologie, Pôle Digidune, CHU Grenoble-Alpes, 38700 La Tronche, France; 11 Centre de Recherche des Cordeliers, Inserm, Sorbonne Université, USPC, Université Paris Descartes, Université Paris Diderot, Paris, France; 12 Mount Sinai Liver Cancer Program, Division of Liver Diseases, Tisch Cancer Institute, Icahn School of Medicine at Mount Sinai, New York, NY, USA; 13 Institució Catalana de Recerca i Estudis Avançats (ICREA), Barcelona, Catalonia, Spain; 14 Hospices Civils de Lyon, Service of Hepato-Gastroenterology, F-69001 Lyon, France

**Keywords:** HCC, autonomic nervous system, neuronal score, transcriptomics, scRNA-seq, cholinergic, M3 muscarinic receptor, spheroids, synergy, TKI resistance

## Abstract

**Background & Aims:**

Owing to unexplained interpatient variation and treatment failure in hepatocellular carcinoma (HCC), novel therapeutic approaches remain an urgent clinical need. Hepatic neurons, belonging to the autonomic nervous system (ANS), mediate liver/whole body crosstalk. Pathological innervation of the ANS has been identified in cancer, nurturing tumor stroma and conferring stronger carcinogenic properties.

**Methods:**

We characterized the innervation of liver tumors from the French Liver Biobank, then applied bioinformatics to TCGA (The Cancer Genome Atlas), several other datasets and a European validation cohort, to re-evaluate patient stratification. Cell biology and pharmacology studies were also performed.

**Results:**

Densely packed nucleated DCX^+^, synaptophysin^+^, NeuN^+^, VAChT^+^, TH^-^, CD31^-^, CD45^-^ clusters, to date undetected, were identified in human HCCs, and independently confirmed by single-cell RNA sequencing data. Using the new concept of a neuronal score, human and rat HCCs displayed tightly netrin-1-associated neural reconfiguration towards cholinergic polarity, which was associated with chronic liver disease progression, cancer onset and many features of aggressive (proliferative class) HCC, including shortened survival. This score was conditioned by tumoral hepatocytes, and predicted sorafenib efficacy in the STORM HCC phase III trial. Conversely, intratumoral adrenergic lymphocytes were enriched in TEMRA and cytotoxic phenotypes. Amongst all cholinergic transcripts, the medically targeted CHRM3 receptor was enriched and associated with pathogenic traits in HCC, as well as poor prognosis in HCC stages 1-2, while its level dropped upon experimental re-differentiation. Its pharmacological inhibition with low concentrations of anticholinergic drugs, but not cholinomimetics, decreased anchorage-independent growth and anoikis, synergized with sorafenib and lenvatinib in HCC class 1 to 3 lines, yet not in primary human hepatocytes, and preserved mature hepatocyte functions.

**Conclusion:**

These data identify cholinergic processes as instrumental in liver carcinogenesis and support the use of EMA/FDA-approved cholinergic drugs in HCC research.

**Impact and implications:**

Hepatocellular carcinoma (HCC) care has long been hampered by the enigmatic nature of disease evolution, as well as of response or resistance to treatment. Hepatic neurons are likely the least studied liver cell type and mediate patients singularities from the ANS to the organ in real-time. Cholinergic inputs identified in this study as pathogenic may be targeted with the well charted pharmacopoeia of neurotropic drugs already available, for basic or clinical research purposes, with an expected high level of safety.

## Highlights

• The liver hosts autonomic neurons that mediate liver/whole body crosstalk.
• HCCs display neural reconfiguration with several immature and cholinergic markers.
• This orientation quantified by a ‘neuronal score’ correlates with aggressive HCCs.
• Low-dose cholinergic targeting decreased cell growth and synergized with TKIs.
• These data identify cholinergic processes as instrumental in liver carcinogenesis.

## Graphical abstract

**Figure:**
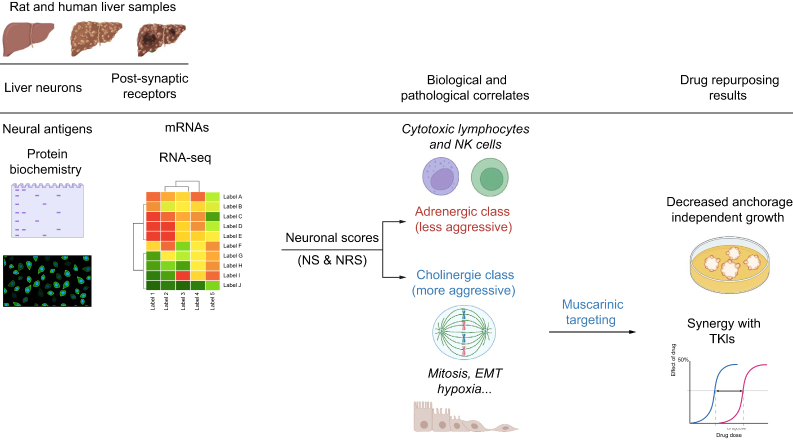

## Introduction

Despite the development of effective therapies against HBV and HCV, deaths related to hepatocellular carcinoma (HCC) have continued to increase. Liver comorbidities, such as MASLD (metabolic dysfunction-associated steatotic liver disease) and alcohol-related liver disease (ALD) combined with metabolic syndrome (MetALD) or not, are long-term cooperators or independent factors fostering the onset of HCC and enhancing disease heterogeneity.^1^ Despite multifactorial etiologies, HCC typically develops in patients with cirrhosis. Treatments with tyrosine kinase inhibitors (TKIs) for instance lead to short-term, unavoidable relapse,^2^ whereas immune checkpoint or growth factor inhibitors currently provide hope for only a fraction of patients with unresectable HCC.

In this respect, cellular/tissular structures linking the general pathophysiology of the patient with HCC are of interest, as they may uncover novel ways of stratifying patients. Several recent works and reviews^3^ have highlighted the relevance of studying neural aspects of cancers in peripheral organs. For instance, pathological innervation and involvement or dysregulation of the autonomic nervous system (ANS) have been identified in ovarian, prostate, gastric and pancreatic cancers,^3^^,^^4^ nurturing tumor stroma and conferring stronger carcinogenic properties. Little, however, is known about the potential role of the ANS in liver oncogenesis.

The ANS comprises the sympathetic (adrenergic signaling) and parasympathetic (cholinergic signaling) arms that relay signals both ways along the brain/liver neural axis in order to regulate involuntary physiological or pathological processes. Oddly, liver nerves are seldom mentioned in anatomical reference textbooks.^4^ The liver is an innervated organ that hosts afferent and efferent ANS nerves, in constant communication with the central nervous system (CNS)^5^ to regulate liver functions.

As notably pointed out by Tracey’s theory,^6^ neural signals also regulate several processes that may impact HCC onset and growth. Portal hypertension, a recognized risk factor for HCC development and recurrence,^7^ is correlated with ANS dysfunction.^8^ The global orientation of liver innervation in chronic liver disease is currently debated in mice and humans,^9^^,^^10^ yet these studies agree on recurrent adrenergic nerve degeneration. Conversely, cholinergic signaling was shown to attenuate apoptosis in the mouse liver^11^ and foster HCC growth.^12^ Interestingly, choline acetyltransferase (ChAT)^+^ regulatory T cells and dysfunctional programmed cell death 1 (PD-1)^+^ T cells, were observed in HCC-bearing mice.^13^ The present study describes neural features in human HCC, and their likely contributions to pathogenesis. Moreover, these results identify a new neural signature that could be relevant for targeting HCC.

## Materials and methods

Expanded experimental biology methods (origin and processing of biological samples, Western blotting, gene expression, immunofluorescence, cell culture approaches, pharmacology, and RNA-seq processing) and statistical methods are detailed in the supplementary file.

### Neuronal receptor score calculation and cohort classification

Gene set scores were calculated using single-sample gene set-enrichment analysis (GSEA) for bulk transcriptomic data^14^ and gene set-variation analysis for single-cell transcriptomic data.^15^ Here, two gene set scores were calculated from both lists of receptors, one including all adrenergic ones, and the other all cholinergic ones, in order to obtain an adrenergic and a cholinergic score, respectively. To obtain a global neuronal receptor score (NRS), the difference (the adrenergic score minus the cholinergic score) was calculated for each sample. The use of a gene set score difference instead of a ratio of gene expression has several advantages. First, the score is computed by taking the entire transcriptome into account, thus overcoming cases where a sample is less covered. This method is common to all transcriptome profiling technologies. Second, NRS values always vary linearly with the evolution of any term of the equation, which is not the case with ratios. Finally, this method is preferential to evaluate the activity of a pathway in a sample by transcriptomics, as it is independent of the number of genes evaluated.

### Transcriptomic datasets

Single-cell RNA sequencing (scRNA-seq) data from patients with HCC was obtained from the Gene Expression Omnibus database accession GSE149614 (n = 10) and https://lambrechtslab.sites.vib.be/en/aHCC (n = 38). Bulk RNA-seq data from paired HCC and non-tumor tissues were extracted from GSE124535 (n = 35), GSE144269 (n = 70) and microarray data from GSE64041 (n = 60). Microarray data from two cohorts of mixed liver disease etiologies were obtained from GSE32879 (n = 37) and GSE89377 (n = 107). Microarray data from patients with HCC treated with sorafenib (n = 67) or placebo (n = 73) were retrieved from GSE109211. Single-nuclei RNA sequencing (snRNA-seq) from MASLD (n = 2) and healthy individuals (n = 2) from GSE174748. Bulk RNA-seq data from paired cholangiocarcinoma and non-tumor tissues from GSE107943 (n = 27), GSE119336 (n = 15) and microarray data from GSE76297 (n = 90).

## Results

### Neural progenitors of cholinergic orientation in human and rat HCC samples

Comprehensive maps of ANS features and innervation are currently lacking in HCC. Interestingly, human liver ANS innervation is more developed than in rodents, as it extends deeper into the lobule,^5^ increasing its regulatory capacities and suggesting that ANS-related mechanisms observed in animals may play more important roles in patients. Human samples, obtained from the French National HCC biobank, were selected across the four major HCC etiologies (HBV, HCV, former ALD, former NASH; 24-26% each). The main characteristics of the patients are provided in Table S1. To characterize HCC innervation, the following classically validated neuron markers were considered: neuronal nuclear antigen (NeuN, phospho- and total, RBFOX3) as a mature, nuclear, neuron marker;^16^ and doublecortin (DCX) and internexin neuronal intermediate filament protein alpha (INA) as immature neuron markers.^17^ Additionally, tyrosine hydroxylase (TH, TY3H) for adrenergic, and vesicular acetylcholine transporter (VAChT) for cholinergic neurons,^18^ were used.

**Fig. 1:**
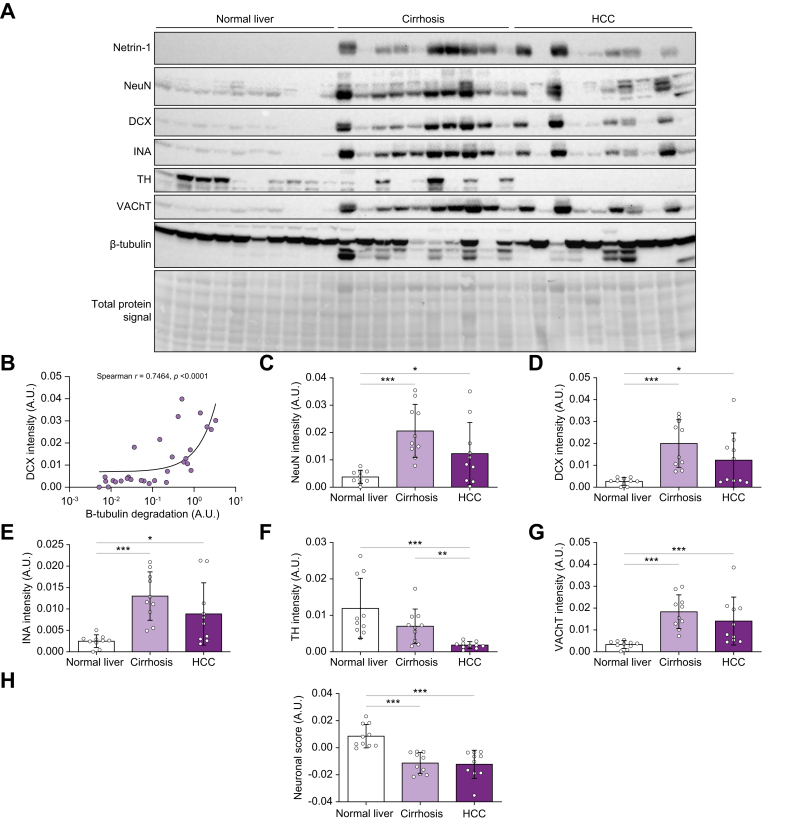
Expression of mature and progenitor neural markers in human HCC. (A) Immunoblotting of netrin-1, NeuN, DCX and INA, TH and VAChT markers on 10 normal liver, 10 cirrhotic (F4) and 10 HCC samples. (B) DCX induction is correlated with parenchymal remodeling. DCX levels were plotted against ratios of full-length *vs.* degraded tubulin signals. Spearman test (∗∗∗*p <*0.001). (C–H) Signal quantification was done using total protein normalization.^41^ Mann-Whitney or *t* test (after normality test, ∗*p <*0.05, ∗∗*p <*0.01, ∗∗∗*p <*0.001). n = 30 patients, from the four main HCC etiologies.

We first investigated the presence of ANS markers in normal human samples (both uninfected and non-fibrotic) *vs.* cirrhotic (minimum distance of 2 cm from tumor) and tumor samples (HCC). Western blotting highlighted positive staining for DCX and INA in tumor samples, and a lower expression of the mature neural marker NeuN, strongly suggesting the presence of immature neurons. In addition, HCC samples lacked the adrenergic marker TH but showed normal expression of the cholinergic neural marker VAChT (representative large blot of 30 patients, Fig. 1A), prompting further analysis. DCX levels were sharply correlated with β-tubulin degradation, suggesting the association of neural alterations with parenchymal remodeling in human samples (Fig. 1B). In an attempt to quantify ANS dynamics, we defined a neuronal score (NS) as the difference between adrenergic and cholinergic signals (see Methods, NS = TH - VAChT). These markers evolved towards a more cholinergic orientation with disease progression (Fig. 1C-H). We then compared the expression levels of such markers in normal livers, cirrhotic and tumoral lesions in samples from the four main HCC etiologies (HBV [n = 14], HCV [n = 9], former ALD [n = 14] and former NASH [n = 14], total of 51 patients). Extensive blots are provided in Figs S1-2 and summarized in Table S2. Comprehensive technical validations for NeuN, DCX, TH and VAChT antibodies are provided in Supplementary information 1. This phenotype was similar to that of the cirrhotic HCC rat model of reference (Supplementary information 2 and Figs S3-5). As in the rat, neurogenic netrin-1 expression was strongly (r >0.85) correlated with proteolyzed β-tubulin and DCX levels, as well as cirrhosis onset, and HCC (Fig. S6). Such clinical data depict cholinergic-oriented alterations of neural networks in late-stage chronic liver disease and HCC.

Given that tissue markers may change with disease progression, we sought to gain insight into the localization of neural signals in human samples. As a first approach, we performed standard fluorescence staining of a cohort of 24 tumors. These were subjected to Masson’s trichrome or HES staining to expose tissue architecture, and then NeuN, DCX, TH and VAChT immunostaining coupled with DAPI staining. Technical validations are provided in Fig. S7. In accordance with blots, TH staining was negligible in both frequency and intensity throughout samples (Fig. S8A-F). Importantly, DCX and VAChT were found in the tumor bulk, where they displayed co-localization (Fig. S8G-J). In order to further confirm these findings, we probed the same samples with CD31 staining to locate vessels, CD45 to exclude leucocytes as potential sources of the signals, and synaptophysin as another independent neuron marker. As shown in Fig. S9, HCC also hosts VAChT^+^, synaptophysin^+^, CD45^-^ and CD31^-^ cells, also nucleated (consistently with NeuN staining), as in prostate cancer with muscarinic signaling.^19^ Although the comprehensive phenotype of these cells remains to be characterized, these data indicate that HCC hosts neural cells of cholinergic orientation. As observed by western blotting, the predominant ANS co-labeling was specific to immature DCX^+^ fibers and cholinergic neural cells, indicating that the neural alterations highlighted herein are likely cholinergic, in line with previous data on steatohepatitis.^10^ These data are consistent with the presence of cholinergic intrahepatic neural cells in the diseased liver as confirmed by snRNA-seq investigation (Supplementary information 3). Such observations were similar between HCC etiologies, and substantiated findings on other solid malignancies,^3^^,^^4^ in which these tumors host nerves with migratory potential, likely tuning their interactions with post-synaptic receptors.

### Greater cholinergic orientation of ANS receptors from normal liver to HCC

**Fig. 2:**
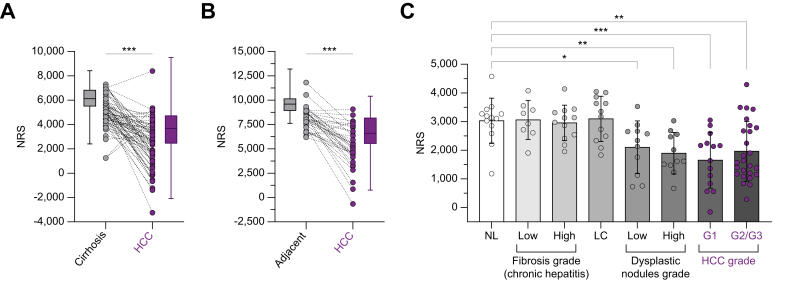
The NRS selectively decreases in HCC *vs.* all other histological states of chronic liver disease. (A) NRS in cirrhosis *vs.* tumors. Wilcoxon matched-pairs signed rank test (∗∗∗*p <*0.001), n = 70 (GSE144269). (B) NRS in adjacent *vs.* tumors. Same test (∗∗∗*p <*0.001), n = 35 (GSE124535). (C) Comparison of NRS between stages (GSE89377). Kruskal-Wallis test corrected with a Dunn’s test (∗*p <*0.05, ∗∗*p <*0.01, ∗∗∗*p <*0.001). Bars represent mean ± SD. NL, normal liver; LC, liver cirrhosis; HCC G1-3, Edmonson grades 1-3 (n = 107 patients total). NRS, neuronal receptor score; HCC, hepatocellular carcinoma.

The balance between adrenergic and cholinergic signals defines a unified ANS output in each innervated organ. To investigate such signals, we first defined a post-synaptic neuro-signature encompassing all adrenergic and cholinergic receptor transcripts. We thus quantified the expression of all transcripts encoding ANS receptors in HCC samples (Table S3). The previously described NS was then adapted to the post-synaptic status of such targets. Hence, its counterpart was termed ‘neuronal receptor score’, NRS. Functional biochemistry data pertaining to the functioning of each receptor are mostly absent in the liver or HCC. As for the NS, the NRS corresponds to the difference between the sums of all adrenergic receptors (except ADRA2, being presynaptic) and all cholinergic receptor signals, providing an integrated view of the balance between ANS receptors in the tissue. Paralleling neural data yet with a delay, the NRS decreased in low-grade dysplastic nodules and more intensely in cancer in three independent datasets based on paired or unpaired samples (Fig. 2A-C), indicating the relevance of further investigations into the relationships between the cholinergic branch of the ANS and HCC.

### Bioinformatics highlight the pathogenic implication of the cholinergic orientation in HCC evolution

To map the interplay between autonomic functions and HCC, we performed a bioinformatics study on the previously published HCC (LIHC) TCGA dataset. Salient features were then considered in an independent cohort of 171 HCC samples from a previous study,^20^ hereafter referred to as the ‘validation cohort’.

**Fig. 3:**
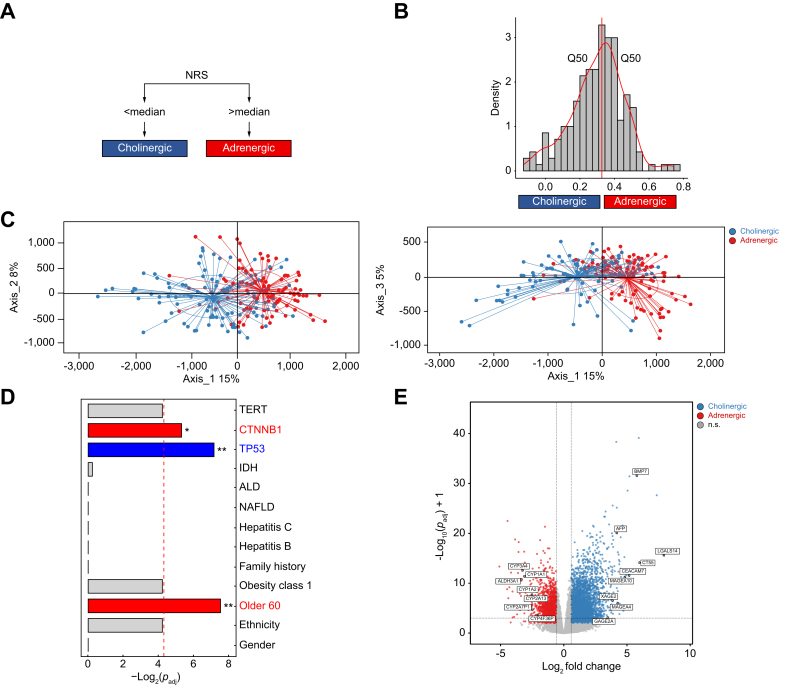
Neural classification based on adrenergic and cholinergic receptors in HCC. (A) NRS calculation: adrenergic and cholinergic enrichment scores were calculated, then cholinergic scores were subtracted from adrenergic scores. (B) Samples were grouped into cholinergic and adrenergic classes by comparison with NRS median. (C) Sample distribution after dimensional reduction (PCA) based on the 10% most variable genes. (D) Associations between classes and main HCC clinico-biological features. Fisher test’s adjusted *p* values per variable (∗*p*_adj_ <0.05, ∗∗*p*_adj_ <0.01). (E) Volcano plot showing significantly differentially expressed genes. Horizontal red bar: adjusted *p* value threshold at 0.001. Genes with lowest adjusted *p* value are indicated. n = 196 patients (TCGA cohort). Modulated genes were considered if absolute Log_2_ fold-change value was higher than 0.58 and *p*_adj_ <0.001. ALD, alcoholic liver disease; NAFLD, non-alcoholic fatty liver disease.

After NRS calculation, samples were split into two classes: those with a higher difference than median were named adrenergic and those with a lower difference than median were named cholinergic (Fig. 3A, see the Methods section). The distribution diagram of NRS values obtained and PCA (principal component analysis) projection of those two classes are shown in Fig. 3B,C. As expected, adrenergic receptors were more strongly expressed in the ‘NRS > median’ class, while cholinergic receptors were more strongly expressed in the ‘NRS < median’ class (Table S3, Fig. S10).

Then, to identify a potential association between ANS orientation and standard parameters in HCC, we tested the correlation between both neuronal classes and gender, ethnicity, etiology, obesity and mutational profile (*hTERT, TP53, CTNNB1*). A Fisher's exact test comparing the signature of each class to each variable was constructed (Table S4). Importantly, no association could be seen between either neural class and gender, ethnicity, obesity, family history, or any of the four main HCC etiologies (Fig. 3D). *CTNNB1* mutations and age >60 years emerged as positively associated with the adrenergic class. *TP53* mutations emerged as positively associated with the cholinergic class. A strong trend for association between the adrenergic class and *CTNNB1* mutations was also found in the ‘validation cohort’ (Table S5). These data suggest that these two classes defined by this ANS-based signature may redefine the current stratification of HCC heterogeneity based on genetics. This question warrants further investigations across different disease stages and ethnic backgrounds, as suggested for instance in HCC studies on Mongolian samples.^21^

To identify genes associated with tumor ANS features, we performed differential gene expression analysis between the two neuronal classes (*p*_adj_ <0.01 and absolute Log_2_ fold-change >0.58). Results are illustrated in a volcano plot (Fig. 3E). Data related to the 100 most significantly up- and downregulated genes for the adrenergic and cholinergic signatures are shown in Tables S6 and S7, respectively. Differentially expressed genes upregulated in the cholinergic signature include many dedifferentiation-related antigens, unlike transcripts enriched in the adrenergic signature (*e.g., CYP450* mRNAs, Table S8). Of importance, no *CYP450* mRNA was found in the cholinergic class. Altogether, such data support that the cholinergic signature may be correlated with less differentiated HCC tumors.

**Fig. 4:**
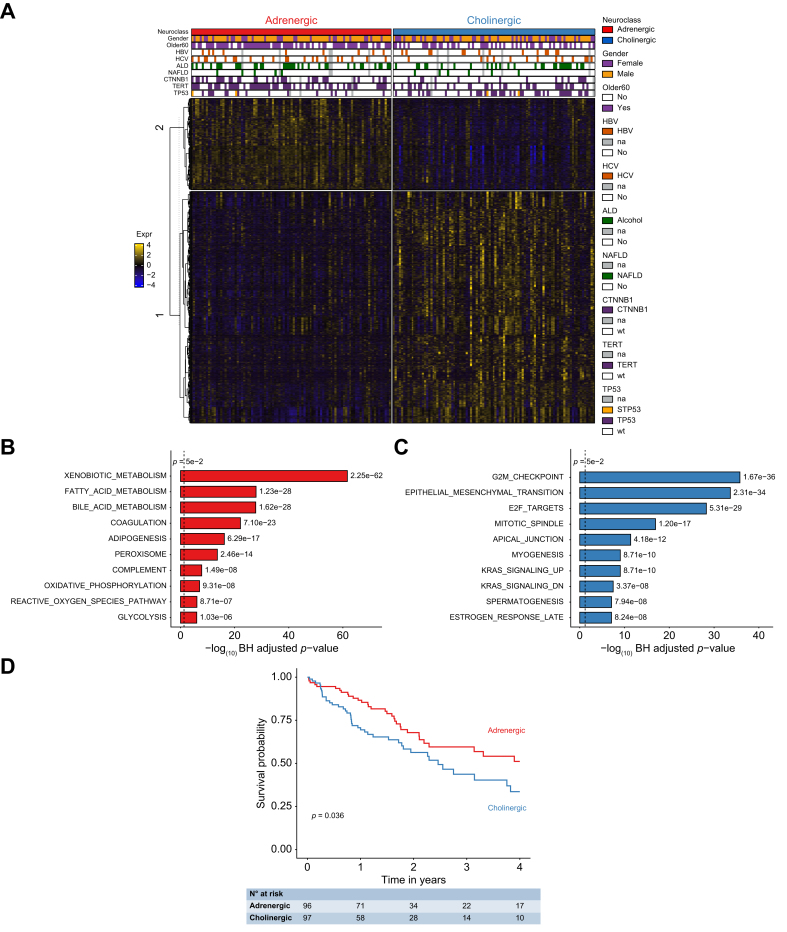
Pathway enrichment and outcome analyses. (A) Heatmap of the normalized levels of DEGs (TCGA cohort) between both neuronal classes, showing 3,264 genes over-expressed in the cholinergic class and 1,288 in the adrenergic one. (B–C) Top 10 over-represented hallmark pathways from the over-expressed genes in the adrenergic (B) and the cholinergic (C) classes, listed in Tables S9 and S10, respectively. Pathways are ordered by adjusted *p* value. (D) Kaplan-Meier representation of the predictive value with respect to the overall survival in TCGA samples. *p* value of the log-rank test is indicated. ALD, alcoholic liver disease; HBV, hepatitis B virus; HCV, hepatitis C virus; NAFLD, non-alcoholic fatty liver disease.

Next, to better understand the phenotypic relevance by deciphering the different molecular pathways defining adrenergic and cholinergic tumors, we performed a Hanzelmann overrepresentation analysis of gene sets, using the differentially expressed genes identified above and shown in a heatmap (Fig. 4A). Genes over-expressed in each tumor class were used as input against Hallmark gene sets of the MsigDB. On the one hand, the most enriched pathways in genes over-expressed in adrenergic tumors corresponded to differentiated, hepatocytic metabolic functions, such as XENOBIOTIC_METABOLISM (71 genes), BILE_ACID_METABOLISM (35 genes), FATTY_ACID_METABOLISM (40 genes) and PEROXISOME (22 genes) (Fig. 4B, Table S9). All these functions are linked with a *CTNNB1* mutational profile^1^ that is associated with this neuronal class. As a control, the β-catenin target *GLUL* was also positively associated with this adrenergic class (+1.52 Log_2_; rank 241). On the other hand, many pathways associated with cell cycle and proliferation were significantly enriched in cholinergic tumors. Indeed, more than 50 genes were associated with pathogenic, proliferative pathways such as, amongst others with consistent adverse, pro-mitotic, outcomes: G2M_CHECKPOINT (72 genes), E2F_TARGETS (64 genes) and EPITHELIAL_MESENCHYMAL_TRANSITION (70 genes) (Fig. 4C, Table S10), in agreement with the frequent *TP53* mutation found in these tumors. Interestingly, panels B and C corroborate genetic data of Fig. 3D. Indeed, TP53mut tumors are enriched in mitotic pathways and cholinergic lesions are also enriched in these pathways. Control pathways such as cardiac (adrenergic) and nausea/vomiting (cholinergic) pathways were identified in their expected classes. In line with differentiation data, these results suggest a more deleterious profile for the cholinergic class. Survival analyses confirmed this hypothesis, showing an association between the adrenergic class and longer overall survival within a timeframe of 4 years (Fig. 4D). Data were confirmed by GSEA on the MSigDB C2 and C5 gene set collections, that showed an enrichment in *CTNNB1* mutation-associated metabolic functions in adrenergic samples and *TP53* mutation-linked proliferative and mitogenic pathways in cholinergic samples (Figs S11-12).

**Fig. 5:**
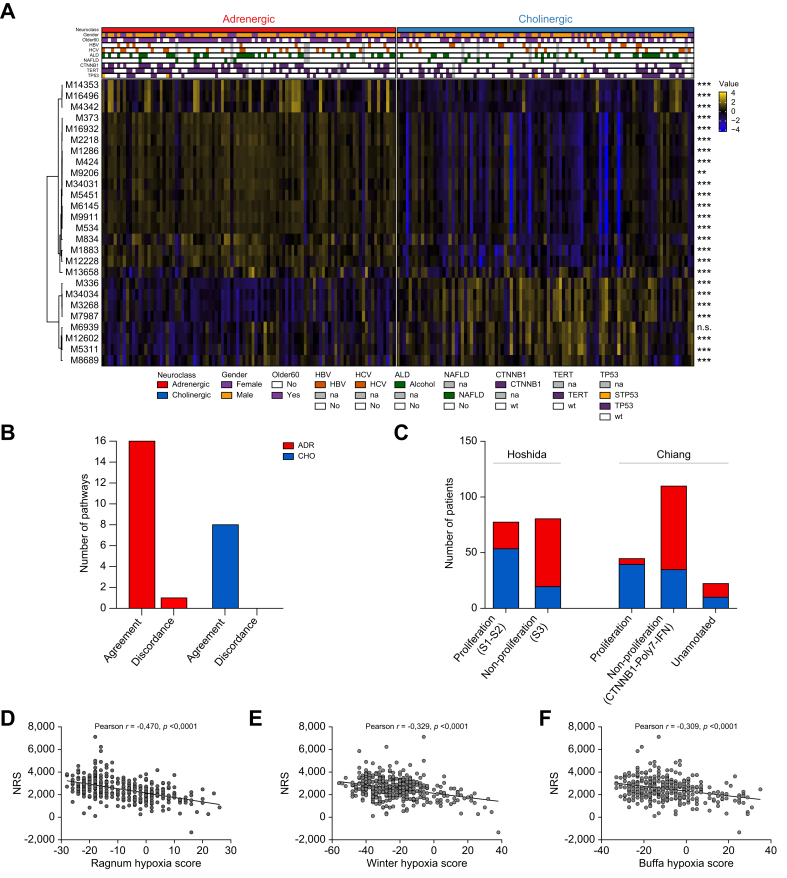
Association between adrenergic or cholinergic orientations and HCC pathological criteria. (A) Enrichment in good or worse prognosis-associated pathways for the adrenergic and cholinergic classes, respectively. Pathway accession numbers (listed in Table S11) are shown to the left. Wilcoxon test was performed for each sample between the two neuronal classes (∗∗*p*_adj_ <0.01, ∗∗∗*p*_adj_ <0.001, n = 293 patients). (B) Canonical HCC pathways in accordance or discordance with adrenergic or cholinergic functional transcriptomics. Pathways associated are depicted in Table S11. (C) Verification of TCGA findings on the ‘validation cohort’ (n = 171 patients). (D-F) Cholinergic HCC orientation is correlated with hypoxia parameters of reference. Pearson’s correlation ∗∗∗*p <*0.001, n = 366 patients, TCGA cohort). ALD, alcoholic liver disease; HBV, hepatitis B virus; HCV, hepatitis C virus; NAFLD, non-alcoholic fatty liver disease.

Among all pathways unveiled, we focused on HCC-specific signatures known to be related to good or poor prognosis.^1^ We performed single-sample GSEA quantification of all these pathways for each sample and compared the two neuronal classes using Wilcoxon’s tests. Almost all of these HCC-specific signatures (Table S11) were differentially enriched between both classes (Fig. 5A). The adrenergic class was statistically associated with 17 HCC canonical signatures, 16 of which were functionally consistent with the transcriptomics of this class (*i.e.,* related to better prognosis). The cholinergic class was linked to eight HCC canonical signatures, all related to poor prognosis (Fig. 5B, Table S12). The present study yielded consistent results in the ‘validation cohort’ (Fig. 5C). Likewise, cholinergic polarity was repeatedly associated with increased hypoxia, a process linked to tumor aggressiveness and TKI resistance,^22^ using three representative hypoxia scores (Fig. 5D-F). All these findings argue in favor of a worse prognosis for patients with HCC and higher cholinergic signaling.

### Dedifferentiated hepatocytes display low NRS values in HCC

**Fig. 6:**
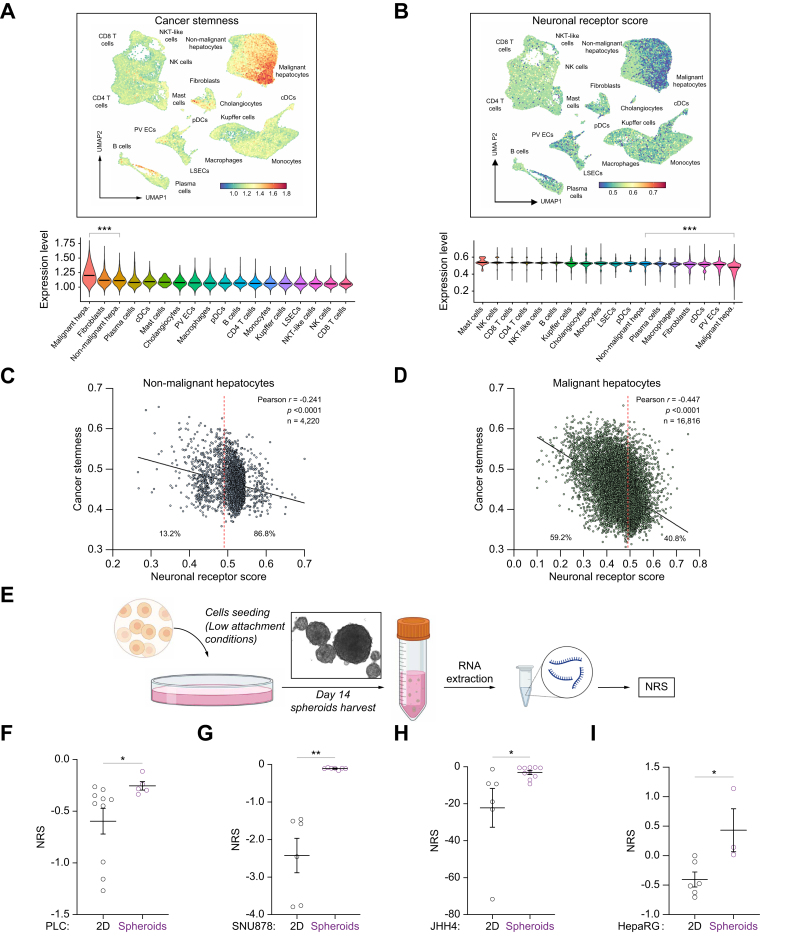
Cholinergic HCC orientation (low NRS) is conditioned by dedifferentiated hepatocytes. (A) GSVA scores for the cancer stemness signature in each cell type, represented as UMAP (top) and violin plot (bottom). Two-tailed *t* test (∗∗∗*p <*0.001, GSE149614, n = 10 patients). Violin plots depict the mean of each population. (B) GSVA scores for the NRS in each cell type, represented as UMAP (top) and violin plot (bottom). Two-tailed *t* test (∗∗∗*p <*0.001, GSE149614, n = 10 patients). (C-D) Correlation between NRS and malignancy (cancer stemness signature) in non-malignant (C) and malignant (D) hepatocytes. Percentages denote hepatocytes above (adrenergic) or below (cholinergic) the median NRS (GSE149614). (E) Workflow. Created with BioRender. (F–I) The NRS becomes more adrenergic (*i.e.,* increases) upon experimental hepatocytic re-differentiation (PLC, SNU878, and JHH4 spheroids; n = 5-10 independent experiments) or upon HepaRG 2D differentiation; n = 3-6 independent experiments). Mann-Whitney test or *t* test (depending on normality, ∗*p <*0.05, ∗∗*p <*0.01). NRS, neuronal receptor score.

Tumors are heterogenous in terms of cell types, and bulk analyses may not address this issue thoroughly. To provide cell-type relevance to these data, we first searched for tumor cell type(s) likely dictating cholinergic-oriented (*i.e.,* lower) NRS in HCC samples. Dedifferentiated (or ‘malignant’) hepatocytes, specifically, were identified as such by scRNA-seq (Figs 6A-D and S13). The ‘malignant hepatocyte’ signature was used as defined in a reference study.^23^ Data were confirmed functionally using hepatocyte-like spheroids and a differentiation protocol for 2D cultures. Consistently with previous data, the NRS increased (*i.e.,* becomes more adrenergic) with cell differentiation (Fig. 6E-I, functional validation of re-differentiation in Fig. S14, as published elsewhere^24^) paving the way for downstream perturbation studies. Functional and pharmacological data were derived from the three classes of the currently admitted classification of HCC lines^25^ after assessment of their suitability for each assay (Table S13).

### The cholinergic receptor CHRM3 participates in cancer cell growth, dedifferentiation and resistance to HCC-relevant TKIs

**Fig. 7:**
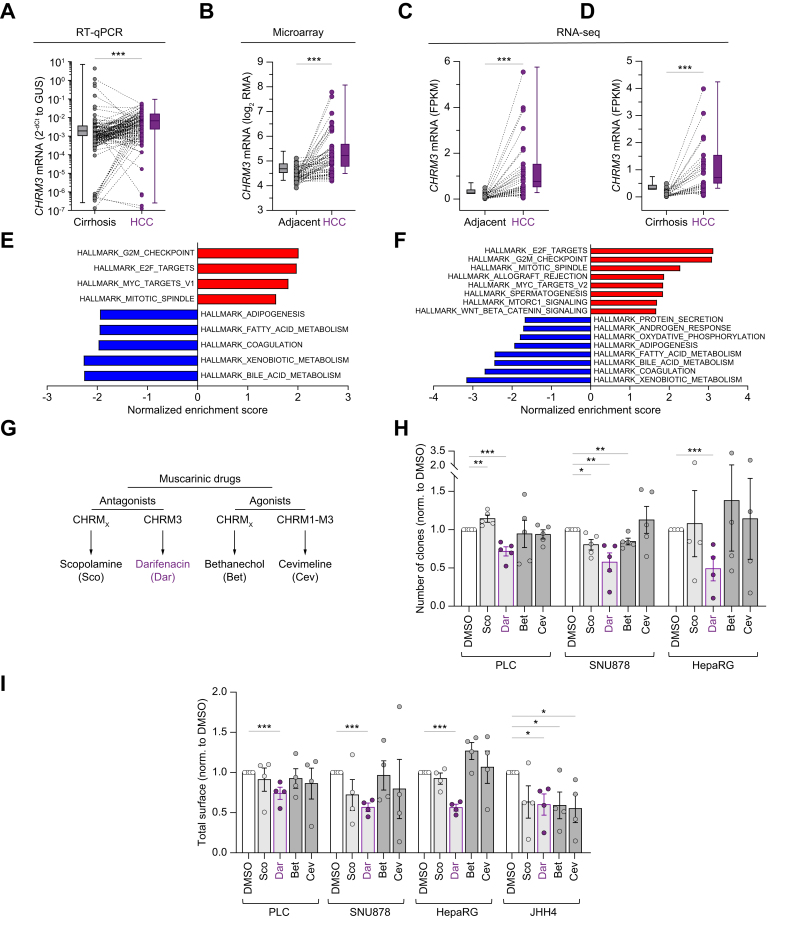
Muscarinic receptors are druggable target candidates in HCC. (A) *CHRM3* expression (French national liver biobank, all four main etiologies 24-26% of total, paired tissues, n = 168 cases). (B) *CHRM3* expression is upregulated in HCC lesions mainly associated with alcohol abuse. ALD etiology corresponds to 72% of the cohort (paired tissues, GSE64041, n = 60 patients, Y-axis units: Log_2_ RMA). (C-D) *CHRM3* expression is upregulated in HCC lesions (paired tissues, GSE124535, n = 35 patients, Y-axis units: FPKM). (B–C) All adjacent tissues; (D) cirrhotic adjacent tissues only. (A-D) Wilcoxon matched-pairs signed rank test, ∗∗∗*p <*0.001. (E-F) *CHRM3*^high^ tumors are correlated with aggressive HCC. Patients were classified into the highest 25 percentile (*CHRM3*^high^) and lowest 25 percentile (*CHRM3*^lo^). GSEA was performed using the MSigDB gene sets H, C2 and C5. Figures show normalized enrichment scores from differentially modulated hallmark gene sets in *CHRM3*^high^ tumor samples (FDR <0.05). (G) Dendrogram describing muscarinic drugs. (H–I) Soft agar (n = 4 or 5) and anoikis (n = 4) assays were conducted on PLC, SNU878, HepaRG and JHH4 (JHH4 unsuitable for soft-agar assay). Student’s *t* test after normality test, ∗*p <*0.05, ∗∗*p <*0.01, ∗∗∗*p <*0.001. HCC, hepatocellular carcinoma.

Within the cholinergic branch, the *CHRM3* transcript encodes a receptor targetable by the FDA- and EMA-approved drug darifenacin. *CHRM3* was frequently and strongly upregulated in tumor lesions compared to adjacent tissue in three cohorts, using cirrhotic or non-cirrhotic tissue as non-tumor controls (Fig. 7A-D). In addition, HCC was one of the few cancer types where *CHRM3* was expressed at moderate to strong immunoreactivity levels in the Protein Atlas database^26^ (Supplementary information 4). *CHRM3* was also repeatedly correlated with several pathogenic hallmarks of proliferative HCC (Fig. 7E,F). To provide causal data concerning hepatocytic cells, we evaluated the sensitivity of HCC lines belonging to all classes^25^ to cholinergic drugs. The soft-agar assay, which provides results in close correlation with *in vivo* HCC data in treatment studies^27^^,^^28^ was used. Importantly, these lines span the entire spectrum of *CHRM3* expression in HCC lines, all of which express it robustly in the Liver Cancer Cell Line Database (https://lccl.zucmanlab.com/hcc/home), as in numerous HCC cases.^26^ Data indicate that targeted functional blockade of the CHRM3 receptor using low concentrations of darifenacin (see Supplementary information 5) hampered colony formation, whereas no phenotype could be obtained using either scopolamine a non-selective muscarinic CHRM3 antagonist, or agonists (Fig. 7G,H). Since none of the class 3 HCC lines tested grew in this context, we then submitted a similar yet larger panel of HCC lines to an anoikis induction protocol. Likewise, limited doses of darifenacin displayed the most robust inhibitory capacity (Fig. 7I). To corroborate such data, we then induced polarization of 2D cultures of these HCC lines into spheroids (re-differentiation previously validated in Fig. S14). Conversely, we observed an important drop (>10-fold) in *CHRM3* transcript levels, indicating that it correlated with hepatocytic differentiation (Fig. S15). Because of this dataset, we then evaluated the potential poor prognostic status of *CHRM3* expression in HCC using the ‘kmplot’ interface (https://kmplot.com) that considers the LIHC TCGA cohort. When specifically analyzing HCC samples at stages 1 or 1+2 (*i.e*., early or well-differentiated cases, n = 171 or n = 253 patients, respectively), significance was reached (log-rank *p* = 0.042 and 0.023, Fig. S16). CHRM3-mediated pathogenic contribution to early carcinogenesis in particular deserves further investigation, as its targeting could be beneficial to patients receiving loco-regional therapy. Altogether, this dataset identifies the CHRM3 receptor as implicated in the pathogenic properties of HCC cells.

**Fig. 8:**
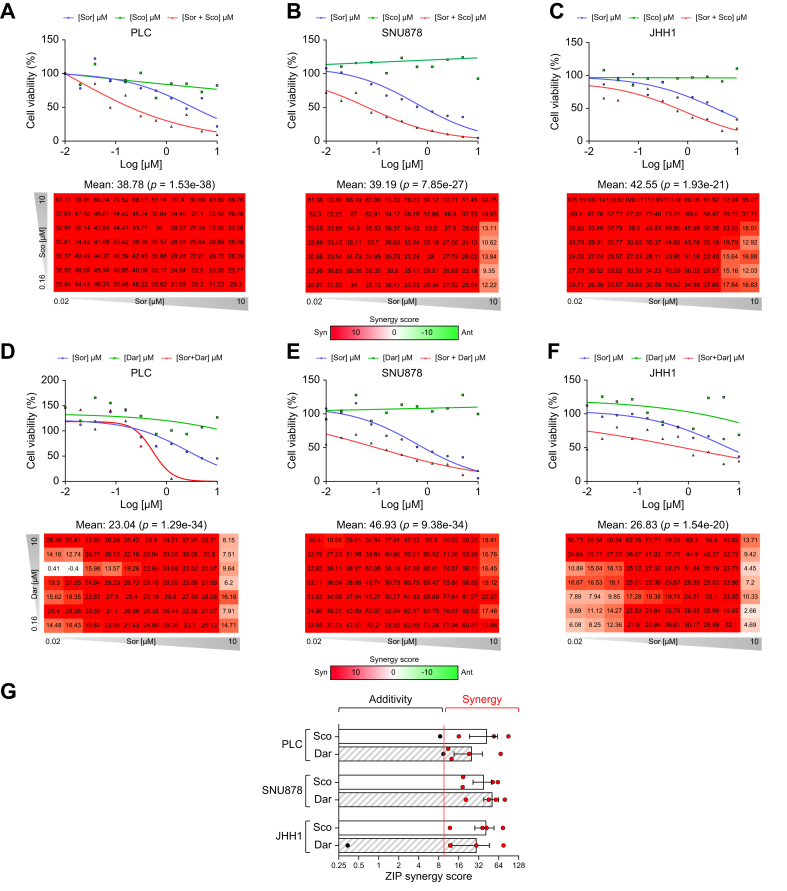
Muscarinic blockade synergizes with the first-line TKI sorafenib. (A-F) Activity curves and 2D matrices on scopolamine (A-C) and darifenacin (D-F), respectively on PLC (class 1), SNU878 (class 2) and JHH1 (class 3) HCC lines. Chou-Talalay ZIP scores >10 indicate synergy. (G) Plate-wide average ZIP synergy score values calculated from 96-well matrices for each experiment (n = 4-6). Dar, darifenacin; Sco, scopolamine.

In patients with HCC, TKIs have objective but limited efficacy over time due to near unavoidable tumor escape.^1^ We tested a possible association between low NRS (*i.e.* cholinergic tumor orientation as a marker of proliferation) and sorafenib activity in the context of the STORM HCC clinical trial, aimed at evaluating sorafenib as an adjuvant therapy for early HCC after resection or local ablation.^29^ Molecular characterization of this cohort identified patients benefiting from sorafenib in terms of objective sensitivity (‘sorafenib responders’) and patients for whom sorafenib had no effect (‘non-responders’).^30^ In this context, specifically in the sorafenib-treated group, a low NRS was associated with sensitivity to the drug (Fig. S16). This result is in agreement with sorafenib’s multikinase inhibitor status, affecting, amongst others, the MAPK/ERK pathway, which notably targets HCC cell proliferation^31^ and further supports the relevance of the NRS and cholinergic cues in the disease. Acquired resistance to sorafenib is an issue in HCC research.^22^ Consequently, HCC lines were subjected to accepted synergy assays between neuroactive drugs and two HCC-relevant TKIs (Chou-Talalay derived ZIP method^32^). These assays identified a synergistic relationship between sorafenib and scopolamine (Fig. 8A-C), as a pan-muscarinic inhibitor. We then searched for the implicated receptor using darifenacin and reproduced this dataset, indicating that the M3 receptor supports this phenotype (Fig. 8D-F). Quantitative values of average synergy scores are provided in Fig. 8G. Data were mostly confirmed with the co-first-line TKI lenvatinib (Fig. S17). Highlighting the importance of inhibiting such function, no activity could be observed upon usage of muscarinic agonists (Fig. S18). Primary human hepatocytes used as controls yielded neither sensitivity to cholinergic drugs nor synergy after combination with TKIs (Fig. S19). Moreover, in the HepaRG differentiation model,^33^ none of these drugs displayed dedifferentiation effects on tested exocrine or endocrine functions, considering bile canaliculi counts, hepatocyte nuclear factor 4-α DNA binding levels, or secreted albumin levels (Fig. S20). The CHRM3 receptor functionality was verified (Supplementary information 5). These functional data confirm the known absence of hepatotoxicity of these drugs in the clinic, as well as their known absence of hepatic drug interaction with any of the TKIs of interest herein. Of note, highly similar cholinergic phenotypes were observed in cholangiocarcinoma (Supplementary information 6). Altogether, these results indicate the compatibility of these strategies with cancer-predisposed, even if functionally weakened, livers.

## Discussion

Intra- and interpatient heterogeneity is a major challenge for HCC research. It is fueled by a plethora of combinations between etiologies, differentiation grades, immune features, genetics, and histological subtypes. Like many other organs, the liver permanently communicates with the brain through afferent and efferent nerves.^5^ It is therefore possible to propose that patient neurological features interact or interfere with intrahepatic neural processes, including in HCC. Genetic mutations are discrete events, likely caused by local, mutagenic events that have escaped overwhelmed DNA repair pathways in a context of chronic proliferation. As an ANS-influenced value, the NRS likely fluctuates at the cross-roads of hepatic and systemic influences longitudinally in a single patient, and across patients as well. This suggests that the NRS may constitute a basis for the development of whole organism-sensitive criteria for HCC stratification. However, the causal relationship between mutations and evolution of the NRS values remains undefined, warranting *in vivo* experiments in the future. The main limitations of our study are two-fold. First, none of the six anti-CHRM3 antibodies tested by western blotting or flow cytometry was found to be RNAi- or Crispr/Cas9 sensitive, hampering loss-of-function studies. Second, although both neural classes identified herein were clearly associated with stronger (cholinergic-oriented) or weaker (adrenergic-oriented) pathogenic HCC phenotypes, where higher *ADRA2B* expression is related to good prognosis,^34^ proteomic confirmation of these findings on a large set of ethnically diverse patients^21^ will be necessary to confirm RNA data. The NRS is a brain/body-conditioned, physiologically integrative, quantitative index that may help identify ANS drugs in any innervated organ or tissue. Our data enrich the current landscape of predictive transcriptomic signatures, since, beyond the traditionally admitted genetic criteria, current stratification based on NRS distribution identified a druggable set of cholinergic receptors. Charting their expression (including *CHRM3*’s) upon HCC recurrence will be of substantial clinical interest as well.

In the liver, Walter Cannon’s (1915) *fight-or-flight* model, historically accepted to describe ANS functions,^35^ predicts that adrenergic signaling mobilizes intracellular hepatocytic energy pools for peripheral energetic needs, whereas cholinergic signaling fosters intrahepatic nutrient storage and related processes, such as liver expansion. This model seems relevant in HCC, where liver expansion also implicates an increase in liver cell size.^36^ Frequent comorbidities associated with liver carcinogenesis are excessive body mass index and alcohol intake (as an important energetic source). This suggests that cholinergic signals aiming at fostering liver expansion, due to their implication in the *rest-and-digest* related functions,^37^ could be hijacked by the tumor in a context of excessive nutrient availability. As was shown recently in metabolic dysfunction-associated steatohepatitis,^10^ adrenergic innervation of HCC seems to be weaker than any other stage. Here, cholinergic tumors are associated with many poor prognosis-related pathways. The likely protective and adverse roles of coffee^38^ and tobacco,^38^^,^^39^ respectively, as adrenergic and cholinergic agonists, support these findings.

The second hallmark of this study consists in the identification of (i) muscarinic receptors as *bona fide* targets for impeding HCC cell resistance to first-line HCC TKIs, (ii) their predictive status with respect to sorafenib response in an adjuvant therapy setting, and (iii) the applicability of this approach, which generates no unwanted effects on primary human hepatocyte viability or major hepatocyte functions. The differential effects of M3-selective and pan-muscarinic inhibition in anchorage-independent growth assays *vs.* synergy assays are discussed in Supplementary information 7. On the immune side, two studies implicate general cholinergic^13^ and CHRM3-specific signaling events^40^ in tumor lymphocytes. While the former depicts a stimulating role for acetylcholine esterase-expressing lymphocytes in these processes,^13^ the latter instead suggests, in accordance with our findings, a deleterious role for cholinergic inputs in general and on CD8^+^ T cells when mediated by the murine CHRM3 receptor. Moreover, our analysis of publicly available scRNA-seq data derived from intra-tumoral immune populations suggests that CD8^+^ T effector memory re-expressing CD45RA (TEMRA) and cytotoxic NK cells are the sub-populations with the strongest adrenergic polarization, in contrast to exhausted CD8^+^ T cells that present markedly lower NRS levels (Supplementary information 8). We believe that the diverging conclusions between studies may be related to, as acknowledged by Zheng *et al.*,^13^ the differential involvement of the strong diversity of cholinergic receptors. Both studies however suggest future important discoveries in the field of ACh and HCC immunity.

This study documents a new neural contribution in the pathogenesis of human HCC and describes its potential clinical implications for HCC, classification of other liver cancers and patient stratification. This approach identified targets that have been engaged by EMA- and FDA-approved medicines for decades, and that are therefore adequate for subsequent HCC research aimed at improving patient care.

## Abbreviations

ANS, autonomic nervous system; ALD, alcohol-related liver disease; CHRM3, cholinergic receptor muscarinic 3; CNS, central nervous system; DCX, doublecortin; GSEA, gene set-enrichment analysis; INA, internexin neuronal intermediate filament protein alpha; NeuN, neuronal nuclear protein; NRS, neuronal receptor score; NS, neuronal score; ScRNA-seq, single-cell RNA sequencing; TH, tyrosine hydroxylase; TKI, tyrosine kinase inhibitor; VAChT, vesicular acetylcholine transporter.

## Financial support

This work was supported by INCa (PRT-K 19-033, PLBIO 23-031), La Ligue contre le cancer (France, Comité du Rhône, #R19147CC), financial support from ITMO Cancer of Aviesan within the framework of the 2021-2030 Cancer Control Strategy, on funds administered by 10.13039/501100001677Inserm (PCSI call), and the DevWeCan Laboratories of Excellence network (ANR-LABX-061) and the 10.13039/501100001665French National Research Agency (ANR) within the framework of the IHU EVEREST (ANR-23-IAHU-0008) as part of the program “Investissements d’Avenir”. AARS is the recipient of an 10.13039/501100003323ANRS grant (ECTZ206376). IC is the recipient of an 10.13039/501100003323ANRS predoctoral fellowship (ECTZ63958). JML is supported by grants from the 10.13039/501100000780European Commission (Horizon Europe-Mission Cancer, THRIVE, Ref. 101136622), by an Accelerator Award from 10.13039/501100000289Cancer Research UK, Fondazione per la Ricerca sul Cancro (AIRC) and 10.13039/501100002704Fundación Científica de la Asociación Española Contra el Cáncer (FAECC) (HUNTER, Ref. C9380/A26813), by the NIH (R01-CA273932-01, RO1DK56621 and RO1DK128289), the 10.13039/100001384Samuel Waxman Cancer Research Foundation, by the Spanish National Health Institute (Project PID2022-139365OB-I00, funded by MICIU/AEI/10.13039/501100011033 and FEDER), by the Asociación Española Contra el Cáncer (Proyectos Generales: PRYGN223117LLOV; Reto AECC 70% Supervivencia: RETOS245779LLOV), the 10.13039/501100002809Generalitat de Catalunya (AGAUR, 2021-SGR 01347), and from "la Caixa" Banking Foundation. JML is supported by the Fundació de Recerca Clínic Barcelona -IDIBAPS and a grant from the Spanish National Health Institute (MICINN, PID2022-139365OB-I00, funded by MICIU/AEI/10.13039/501100011033 and FEDER).

## Authors’ contributions

CAH, CV, AARS, IC, AD, AJFL, LT, MS, PB, ZMJ, RE, RPo, and RP generated and analyzed experimental data. PB, GI, GP, AF and ZMJ developed critical approaches and methods for the study. LT, BrT, AF, ZMJ, TD, BaT, SR, AV, JML, and FZ critically amended and conceptually enriched the paper. RP wrote the paper.

## Data availability statement

De-identified individual participant data will be made available upon request 3 months after publication for a period of 5 years after the publication date.

## Conflict of interest

Research Support to JML: Bayer Pharmaceuticals, Eisai Inc, Bristol-Myers Squibb and Ipsen. Consultancy (JML): Bayer HealthCare Pharmaceuticals, Eisai Inc, Merck, Bristol-Myers Squibb, Eli Lilly, Roche, Genentech, Ipsen, Glycotest, AstraZeneca, Omega Therapeutics, Mina Alpha, Boston Scientific, Exelixis, Bluejay, Captor Therapeutics.

Please refer to the accompanying ICMJE disclosure forms for further details.
