# Supplementary material for: Hepatocellular carcinoma hosts cholinergic neural cells and tumoral hepatocytes harboring targetable muscarinic receptors
Source: JHEP Rep. 2024 Nov 12;7(1):101245. doi: 10.1016/j.jhepr.2024.101245 (PMC11663970; doi:10.1016/j.jhepr.2024.101245)
Supplement: Multimedia component 1 [file mmc1.pdf]

Hepatocellular carcinoma hosts cholinergic neural cells and tumoral hepatocytes harboring targetable muscarinic receptors

Charlotte A. Hernandez, Claire Verzeroli, Roca-Suarez AA, Abud-José Farca-Luna, Laurie Tonon, Roger Esteban, Roser Pinyol, Marie-Laure Plissonnier, Ievgeniia Chicherova, Anaëlle Dubois, Pascale Bellaud, Marine Seffals, Bruno Turlin, Alain Fautrel, Gabriel Ichim, Michel Rivoire, Guillaume Passot, Zuzana Macek-Jilkova, Thomas Decaens, Alain Viari, Barbara Testoni, Sandra Rebouissou, Josep M. Llovet, Fabien Zoulim, Romain Parent

Table of contents

Expanded materials and methods 2

Supplementary figures 10

Supplementary tables 31

Supplementary information 37

Supplementary references 56

EXPANDED MATERIALS AND METHODS

Clinical liver samples

HCC samples used in this study were obtained from the French Liver Biobank network (INCa, BB-0033-00085, under IRB agreement of Inserm Ethics Committee (CEEI, #12-063) and the TCGA Research Network (https://www.cancer.gov/tcga) HCC-LIHC cohort. The table presenting expression data was downloaded with the tool TCGA biolinks http://bioconductor.org/packages/release/bioc/html/TCGAbiolinks.html Data were crossed with previously reported metadata to obtain a cohort of 193 patients. Normal liver samples (French South-East region IRB agreement #A16-207) obtained from safety margins of hepatic resections of colorectal cancer metastasis, were histologically normal and devoid of HBV or HCV infection. All research was conducted in accordance with both the Declarations of Helsinki and Istanbul. All research was approved by the appropriate ethics and/or institutional review committee(s), and written consent was given in writing by all subjects.

Rat liver samples

Methods conform to the ARRIVE guidelines. 6-week-old Fischer 334 male rats (Janvier Laboratories) were acclimated for two weeks, given diethyl-nitrosamine (DEN) at 50 mg/kg weekly from day 0 to week 14, to foster progression from chronic liver disease to fibrosis, cirrhosis, and HCC. The experimental unit was a cage of 3 to 4 animals for a total of 28 animals that were fed *ad libitum*. One to two experimental units were allocated to each group for a total of 28 rats. Five rats represented the untreated group. Seven rats represented the DEN+ fibrotic group. Eight rats represented the DEN+ cirrhotic group, likewise in the DEN+ HCC group. Previous publications on this model indicate that at least 7 rats are necessary in DEN-treated groups for robust statistics. No rat was excluded from the analysis. Mixing of all groups in similar proportions was used to allocate experimental units to control and to each treatment group. Animals were sedated using ketamine prior to sacrifice. Rat outcome was measured using the molecular markers depicted in **Fig. S4**. Spearman and Mann-Whitney tests were used under GraphPad/Prism. The status of the data related to the null hypothesis was considered. Rat samples were obtained under the Grenoble-Alpes University agreement #B 38 516 10 006. No conflict of interest interfered with the study's design.

Western blotting

Immunoblotting was performed using 40 µg of lysates processed in lysis buffer (50 mM Tris HCl (pH 8.0), 150 mM NaCl, 1% NP-40, 0.5% sodium deoxycholate, 0.1% SDS, 10 mM sodium fluoride, 50 mM orthovanadate, 1X protease inhibitor cocktail (Roche))-processed cell lysates, then resolved on 8 or 10% SDS-PAGE, blotted onto nitrocellulose membranes (Amersham Biosciences, Saclay, France), blocked using 5% low fat dried milk in TBS Tween 0.1% for 1 h at room temperature (RT) and probed overnight at 4°C with corresponding antibodies listed in the **CTAT Table**. After three washes in TBS-Tween 0.1%, membranes were incubated for 1 h at RT with secondary antibodies coupled to HRP (1/5000, Sigma-Aldrich, St-Quentin, France) prior to chemiluminescence-based visualization using the Clarity Western ECL substrate (Bio-Rad, Versailles, France). Total protein levels were used for normalization because of high variability of housekeeping protein signals in clinical samples, as verified in the present study. Quantification was done on non-saturated images using Fiji and ImageLab software.

Total RNA extraction and RT-qPCR

Total RNA was extracted using Trizol (Invitrogen). RNA samples (1µg) were DNAse I-digested (Promega, Charbonnières, France) and reverse transcribed using SuperScript VILO reverse transcriptase (ThermoFischer, Les Ulis, France) according to the manufacturer's instructions. Quantitative real-time PCR was performed on 1/5th diluted samples on a LightCycler 96 device (Roche, Meylan, France) using the No Rox qPCR mix (Bioline, Paris, France) or using a microfluidic qPCR device (Biomark^{TM} Standard Biotools, San Francisco, USA). PCR primer sequences (5′-3′) and qPCR conditions are listed in **CTAT Table**. Specificity of all primers was assessed by melting curve analyses and agarose gel electrophoresis. Efficacy of all primers was quantified using 3-fold serial dilutions of target templates.

Immunofluorescence

All reagents were from Sigma-Aldrich (St. Quentin Fallavier, France) unless otherwise stated. Samples were fixed for 24 h in 4% formaldehyde (pH 7.0). Progressive dehydration was performed using ethanol and xylene through 70, 80, and 95% ethanol (45 min each), followed by 3 changes of 100% ethanol (1 h each). Tissue was cleared through 2 changes of xylene, for 1 h each. Tissue was then immersed in 3 changes of paraffin, for 1 h each. Inclusion was

automatically performed in a Histocentre3 Shandon (Loughborough, United Kingdom) device prior to cutting 4 µm-thick sections, transferred to SuperFrost slides (Euromedex, Souffelweyersheim, France), and dehydrated at 56°C for 1 h. For Masson's trichrome staining, the whole procedure was performed in a Leica ST5020 (Jena, Germany) apparatus. Paraffin removal was done using xylene and ethanol (backwards compared to protocol described above), followed by water rinsing. Gill hematoxylin was then added for 10 min followed by water rinsing. Saturated lithium carbonate was then used for 5 s before rinsing in water. Hydrogen chloride (0.5%) was then added for a few seconds to stain specimens in pink before another cycle of lithium carbonate. Fuschin Ponceau was then added for 5 min, and phosphomolybdic acid was added for 10 s before 5 min incubation. Lastly, a Light Green staining was done for 5 min, followed by acetified water for 30 s. Slides were finally dehydrated and mounted as depicted above. Final acquisition of images was done using the NanoZoomer Digital Pathology software (Hamamatsu, Massy, Japan). For immunofluorescence *per se*, the Discovery Ultra device (Roche, Illkirch, France) was used. Paraffin removal was done for 8 min (75°C) using the EZPrep reagent (Roche, Illkirch, France), followed by antigen retrieval (8 min / 95°C then 28 min / 100°C in Tris EDTA pH 8.0), followed by incubations of primary antibodies (**CTAT Table**), prior to washing three times with PBS + 0.1% Tween-20 and incubation of secondary antibodies (60 min, 37°C, see figure legend) and identical washing. The Discovery DCC Kit 455 (Roche), the Discovery FAM Kit 505 (Roche), the Discovery Cy5 Kit 660 (Roche), and the Opal Polaris 780 Reagent (Akoya) were used. Counterstaining was performed using DAPI (250 ng/mL) before mounting on SuperFrost slides (Euromedex, Souffelweyersheim, France). Final acquisition of images was done using the NanoZoomer Digital Pathology software (Hamamatsu, Massy, France).

Cell culture

Cells were grown in a 5% CO_2 humidified atmosphere at 37°C. All reagents for cell culture were purchased from ThermoFischer Scientific (Courtaboeuf, France) unless otherwise indicated. Primary human hepatocytes (PHH) were isolated from the regional platform of primary hepatic cells. They were seeded in 96-well plates previously coated with collagen 0.1% in PBS at a density of $2.10^5/cm^2$, and grown in Williams' medium supplemented with 5 mg/mL insulin (Sigma, Saint-Quentin, France), 5.10^{-5} mol/L hydrocortisone hemisuccinate (Merck, Fontenay, France), 2 mM glutamax, 100 IU/mL penicillin, 100 µg/mL streptomycin, 5% fetal bovine serum (FBS) (Hyclone/Perbio, Velizy, France) and 2% DMSO (Sigma-Aldrich). Four different HCC cell lines (PLC, SNU878, JHH4, JHH1) obtained from J. Zucman-Rossi's

laboratory (Inserm, Paris, France) were used after complete STR characterization. All shared common features with the proliferative class of HCC. PLC (CVCL_0485) are hepatoblast-like cells belonging to the CL1 subgroup, SNU878 (CVCL_5102) and HepaRG (CVCL_9720) are mixed epithelial-mesenchymal cells belonging to the CL2 subgroup, JHH1 (CVCL_2785) belong to the CL3 subgroup or are unclassified depending on the criteria of interest. PLC, SNU878, JHH1, JHH4 were cultured in Dulbecco's Modified Eagle Medium (DMEM) supplemented with 10% FBS, 100IU/mL penicillin, 100 µg/mL streptomycin, 2 mM Glutamax, Sodium Pyruvate and 1X MEM Non-Essential Amino Acid Solution. Cells were seeded at a density of $3.10^4/cm^2$. HepaRG cells were seeded at a density of $4.10^4/cm^2$ in William's E medium containing insulin, hydrocortisone hemisuccinate, 100IU/mL penicillin, 100 µg/mL streptomycin, 2 mM Glutamax and 10% FBS. Differentiation of HepaRG cells consisted in 14 days of culture in conventional medium followed by 14 additional days of culture in the same medium supplemented with 1.8% DMSO, as described [1].

Soft Agar

Cells were seeded at the following densities: PLC: $10.500/cm^2$, SNU878: $21.000/cm^2$, HepaRG: $6.500/cm^2$ in 6-well plates. Six wells were used per condition. 2X medium was reconstituted from powder (ThermoFisher Scientific, 52100021), supplemented with sodium bicarbonate (3.7 g/L, Gibco, 25080094), and above-mentioned supplements were added to each above-mentioned medium. Lower and upper agarose layers were constituted of 1 and 0.3% agar (Sigma, A9414) in complete medium, respectively, before being overlaid with 2 mL of complete medium. Culture was performed for 4 weeks. Fresh medium (2 mL) was added to the wells after 2 weeks. Cells were treated with 2 µM of the indicated drugs one day after seeding. Treatments then consisted in 1 µM of each drug twice a week. Clones were stained with 0.005% crystal violet in 10% ethanol.

Anoikis assay

Cells were seeded at the following densities. PLC: $1.250/cm^2$, SNU878: $2.000/cm^2$, HepaRG: $1.250/cm^2$, JHH4: $2.000/cm^2$ in 6-well plates. Six wells were used per condition in a low attachment setting. Culture was performed for 2 weeks. Cells were treated with 2 µM of the indicated drugs one day after seeding. Treatments then consisted in 1 µM of each drug twice a week. At the end of the experiment, micrographs were taken and the total surface (due to colonies fusions and budding processes) of live colonies was measured using the NIS Elements

software, NIS.ai (Nikon, Tokyo, Japan).

Generation of spheroids

Seeding densities were: PLC: 8.000/cm^2, SNU878 and JHH1: 16.000/cm^2. Cells were cultivated in 100-mm Petri dishes on an agarose layer (1%) for 2 weeks overlaid with 20 mL complete medium. Spheroids were harvested by aspiration of medium and low speed (1.000xg) centrifugation.

Drug treatment

Prior to treatment, cells were seeded in 96-well plates (5,000 cells/well for PLC; 10,500 cells/well for SNU878 and JHH1) and allowed to adhere overnight before treatment the next day. The cytotoxic effect of tyrosine kinase inhibitors (TKIs, *i.e.*, sorafenib and lenvatinib) and four cholinergic drugs (scopolamine, darifenacin, bethanechol and cevimeline) in single and combination studies was evaluated after 72 h of treatment. All experiments were carried out using duplicate 96-well plates. First, the half-maximal inhibitory concentration (IC_{50}) value was determined for each drug alone in all HCC cell lines, using a concentration ranging from 0 to 10 µM. Combination studies were then performed by combining TKIs and each cholinergic drug in the same range of concentrations (0 to 10 µM). Control treatment was DMSO. Doxorubicin (10 µM) was used as a control for death induction.

Cell viability assay (MTT)

The effects of single treatments and of neuroactive drugs and TKI combinations on the viability of HCC cell lines and PHH were evaluated using MTT (AbMole, Brussels, Belgium). After 72 h of treatment, 20 µL of MTT solution (10 mg/mL in PBS) was added to each well, including one set of wells devoid of cells as a background control. Cells were incubated in the dark for 3.5 h at 37°C. Then 100 µL/well of DMSO was added to solubilize the formazan crystals prior to a 15 minutes agitation step. Absorbance was measured at 570 nm on a Thermo Scientific Multiskan GO device.

Analysis of drug interactions

To quantify drug interactions between darifenacin/scopolamine and TKIs, viability data derived from neuroactive drugs and TKI combinations were analyzed using the SynergyFinder Plus software [2] (U. of Helsinki, Finland). The principle of dose matrix analysis in drug

combination studies is that each dose of one drug is combined with each dose of the second drug and thus varying dose ratios provide the basis for the final determination of the type of drug interaction. The SynergyFinder Plus software estimates the synergy scores of the drug pairs using four different reference models: Highest Single Agent (HSA), Loewe, Bliss and zero interaction potency (ZIP) scores. Synergy scores <-10 mean that the interaction between two drugs was likely to be antagonistic; from -10 to 10, the interaction was likely to be additive; and if >10 the interaction was likely to be synergistic. ZIP synergy scoring was used in this study (calculation of the expected effect of two drugs assuming that they do not potentiate).

Evaluation of hepatocytic differentiation

DNA binding capacities of endogenous HNF4α were quantified using the Abcam functional kit (Abcam, ab207208) according to the manufacturer's instructions. Secreted human albumin was quantified after low-speed (1000xg) centrifugation using the Abcam human albumin specific kit (Abcam, ab179887) according to the manufacturer's instructions. Absence of appreciable signal in naive (unconditioned) complete medium was verified prior to drawing conclusions. As for the quantification of bile canaliculi densities, a previously published protocol was used [3]. The cells were incubated for 10 min in a medium supplemented with 10 μg/mL of fluorescein diacetate. After changing to a fresh medium, the cells were incubated to excrete fluorescein for 20 min. Then, after moving the cells to fresh medium, the cells were incubated for a further 30 min to allow excretion to occur. Fluorescent foci were then counted (3 random fields per well, 3 well per biological condition) using DAPI stained nuclei for normalization and the Fiji software.

Sc/snRNA-seq data processing

Integration of samples was performed using the *FindIntegrationAnchors* and *IntegrateData* functions of Seurat [4]. Cell clustering was performed using the *FindClusters* function of Seurat and a resolution of 0.5 for the major cell types and 2 for sub-clustering. Marker genes for each of the clusters were identified with the *FindMarkers* function of Seurat. Gene set variation analysis (GSVA) [5] was employed to assign activity estimates for the NRS, neuronal signaling pathways and the cancer stemness signature [6]. Cell type labels were assigned to each cluster using the markers identified with Seurat, the cancer stemness signature scores and a list of previously described cell type-specific markers [7]. Activities of the NRS and the cancer

stemness signature were compared between malignant and non-malignant hepatocytes using an unpaired two-tailed T-test.

Bulk RNA-seq and microarray data processing

Differential gene expression was performed with the DESeq2 Bioconductor R package relative to previous clustering (classes by synaptic receptor, adrenergic-cholinergic signature). The adjusted *p*-value was set to 0.01 and Log2 fold change (FC) abs > 0.58. The "High" (adrenergic) class was considered as reference. For the analysis of datasets GSE124535, GSE89377, GSE144269 and GSE109211, data was pre-processed with the *CollapseDataset* tool available at GenePattern. Activity of the NRS was estimated using single-sample gene set enrichment analysis (ssGSEA). For the TCGA LIHC cohort, after crossing with mRNA data, miRNA quantitative data for 189 samples was obtained. As for the mRNA, a differential expression analysis with DESeq2 was done in order to compare features obtained upon clustering the adrenergic and cholinergic signature classes.

Pathway enrichment analysis

Overrepresentation analysis of pathways was performed with the enricher function from the clusterProfiler R package, using as input over- and under-expressed genes previously obtained by differential gene expression analysis. Hallmark gene sets from the MSigDB were used, and pathways enriched with an adjusted *q*-value < 0.1 were considered. For GSEA, the R package fgsea was used. Genes were pre-ranked by the signal to noise ratio between cholinergic and adrenergic samples, and the enrichment analyses were performed on the C2 and C5 gene set collections from the MsigDB.

Statistics

Statistical analyses and tests pertaining to the bioinformatics analysis were carried out with the R software and GraphPad Prism. Heatmaps were generated with ComplexeHeatmap, principal component analysis was completed with ade4, and plotted with factoextra or ggplot. Gaussian finite models were performed with Cluster and ClusterProfiler. Figures were created using the R software. Neuronal receptor scores (NRSs) were compared between paired adjacent and tumor tissues in the datasets GSE124535 and GSE144269 using a Wilcoxon matched-pairs signed rank test. NRSs were compared between liver disease stages in the dataset GSE89377

using a Kruskal-Wallis test corrected for multiple comparisons with a Dunn's test. NRSs in the dataset GSE109211 were compared between responders and non-responders to therapy using a Kruskall-Wallis test. Statistics on samples derived from the French National HCC biobank cohort were done as follows. Normal distribution of data was first assessed using the Shapiro-Wilk test. The associations between receptor transcript levels and clinico-pathological variables were determined in multiple comparison by Kruskal-Wallis test, Mann-Whitney test and Spearman correlation. For the TCGA LIHC cohort, paired tissues were analyzed with the Wilcoxon test. Association between the NRS and the Ragnum, Winter and Buffa hypoxia scores [8-10] was evaluated using a Pearson correlation coefficient. Kaplan-Meier plots and log-rank tests were used to evaluate the prognostic value of the receptors by univariate Cox proportional hazards regression model. The survival data (OS, PFI) were extracted from Liu *et al*. [11]. Survival analyses were conducted with survival [12, 13] and survminer [14] R packages. The survival analysis was performed at 2, 3, 5 and 10 years. $p<0.05$ was considered significant.

SUPPLEMENTARY FIGURES

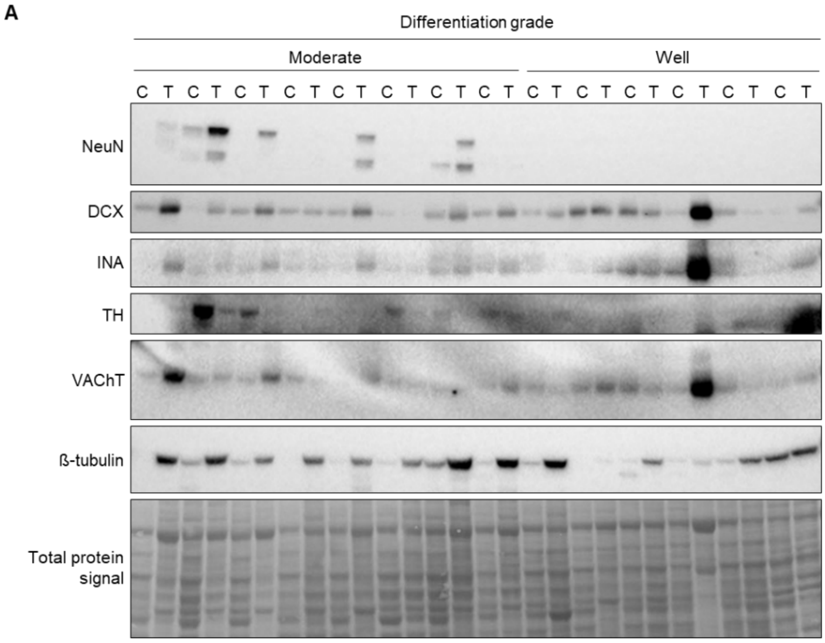

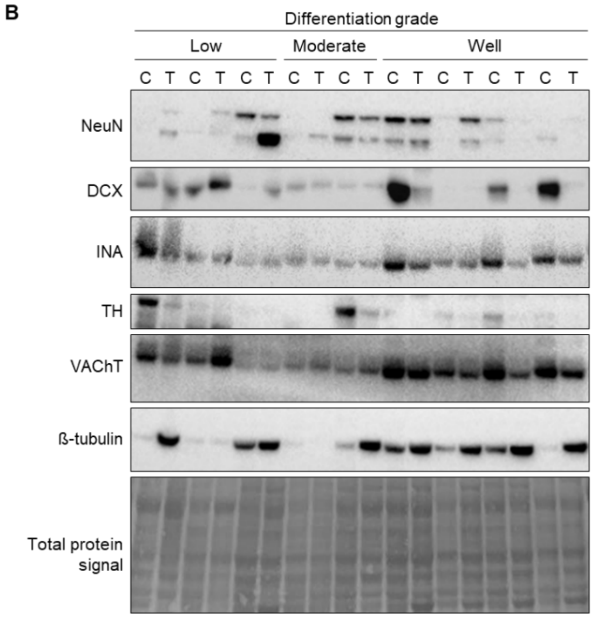

Fig. S1. Expression of mature and progenitor neural markers in human HCC of HBV or HCV origin. Immunoblotting using antibodies against NeuN, DCX, INA, TH and VAChT neural markers and β-tubulin as an internal control. Quantification was done after total protein normalization. Results are shown in **Table S3**. (**A**) HBV etiology (n=14 patients). (**B**) HCV etiology (n=9 patients). C, cirrhosis; DCX, doublecortin; INA, internexin neuronal intermediate filament protein alpha; NeuN, neuronal nuclear antigen; T, tumor; TH, tyrosine hydroxylase; VAChT, vesicular acetylcholine transporter (*SLC18A3*).

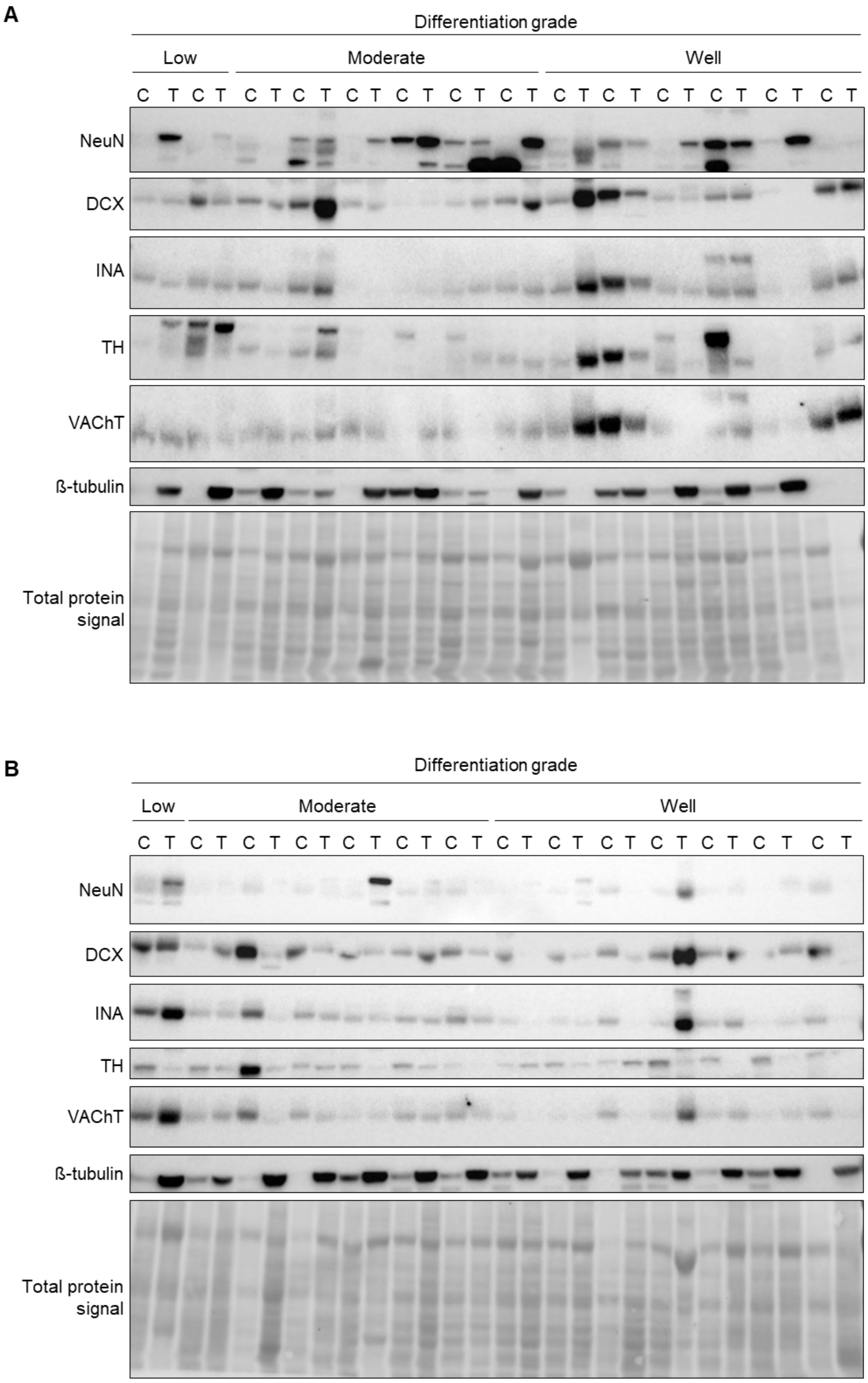

Fig. S2. **Expression of mature and progenitor neural markers in human HCC of ALD or NASH origin.** Immunoblotting using antibodies against NeuN, DCX, INA, TH and VAChT neural markers and β-tubulin as an internal control. Quantification was done after total protein normalization. Results are shown in **Table S3**. (**A**) ALD etiology (n=14 patients) (**B**) NASH etiology (n=14 patients). ALD, alcoholic liver disease; C, cirrhosis; DCX, doublecortin; INA, internexin neuronal intermediate filament protein alpha; NASH, non-alcoholic steatohepatitis; NeuN, neuronal nuclear antigen; T, tumor; TH, tyrosine hydroxylase; VAChT, vesicular acetylcholine transporter (*SLC18A3*).

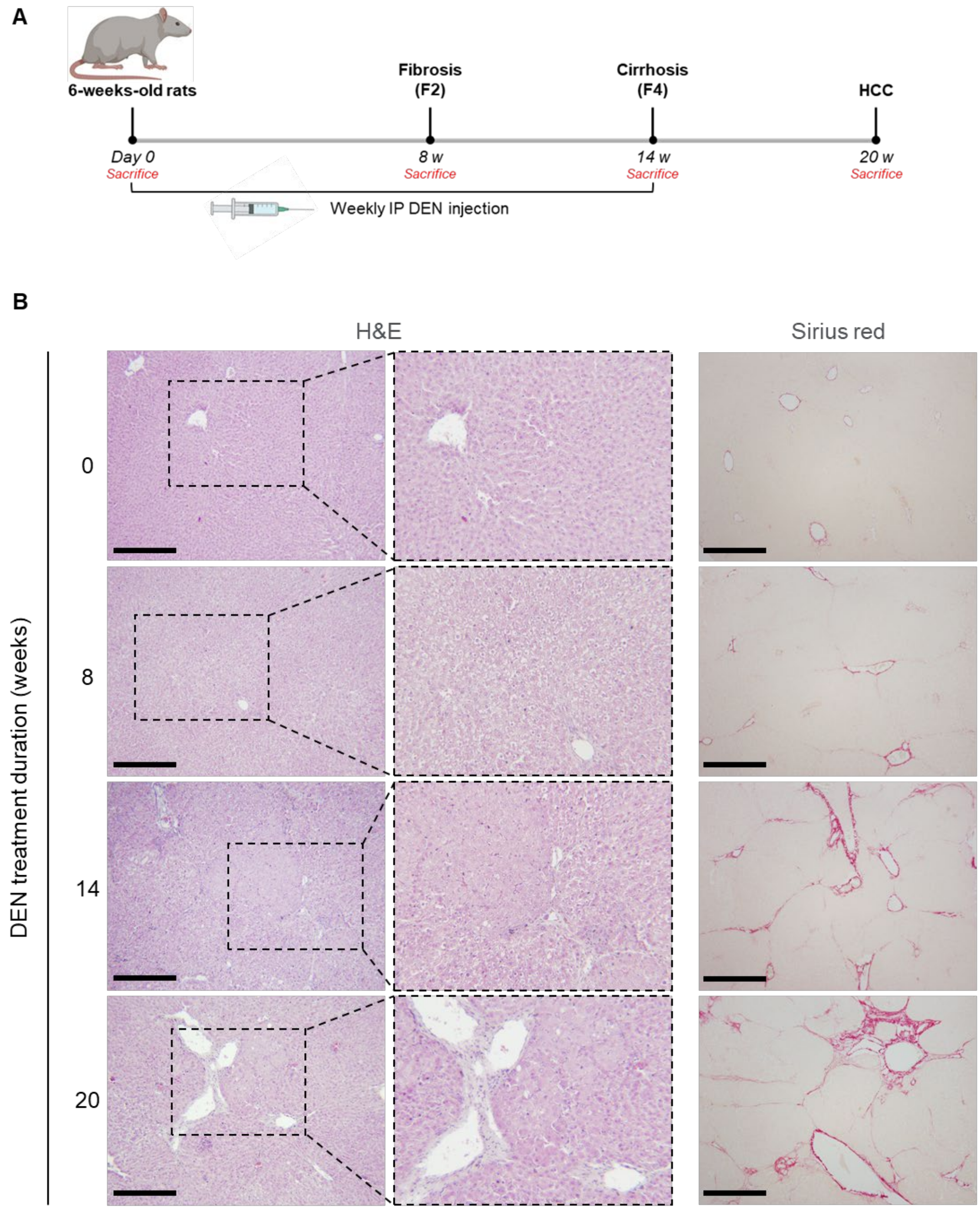

Fig. S3. Hematoxylin/eosin and Sirius Red staining of CLD and HCC rat samples. (**A**) Experimental outline. 6-week-old Fischer 334 rats were given DEN at 50 mg/kg weekly from day 0 to week 20, allowing progression of chronic liver disease to fibrosis, cirrhosis, and HCC. Created with BioRender. (**B**) The liver was then fixed, sectioned and stained with H&E or Sirius Red, and visualized under a bright field microscope. Data are representative of 5, 8, 8, and 7 individuals for the following time points: 0, 8, 14 and 20 weeks, respectively. Scale bar: 500 µm. CLD, chronic liver disease; DEN, diethyl-nitrosamine.

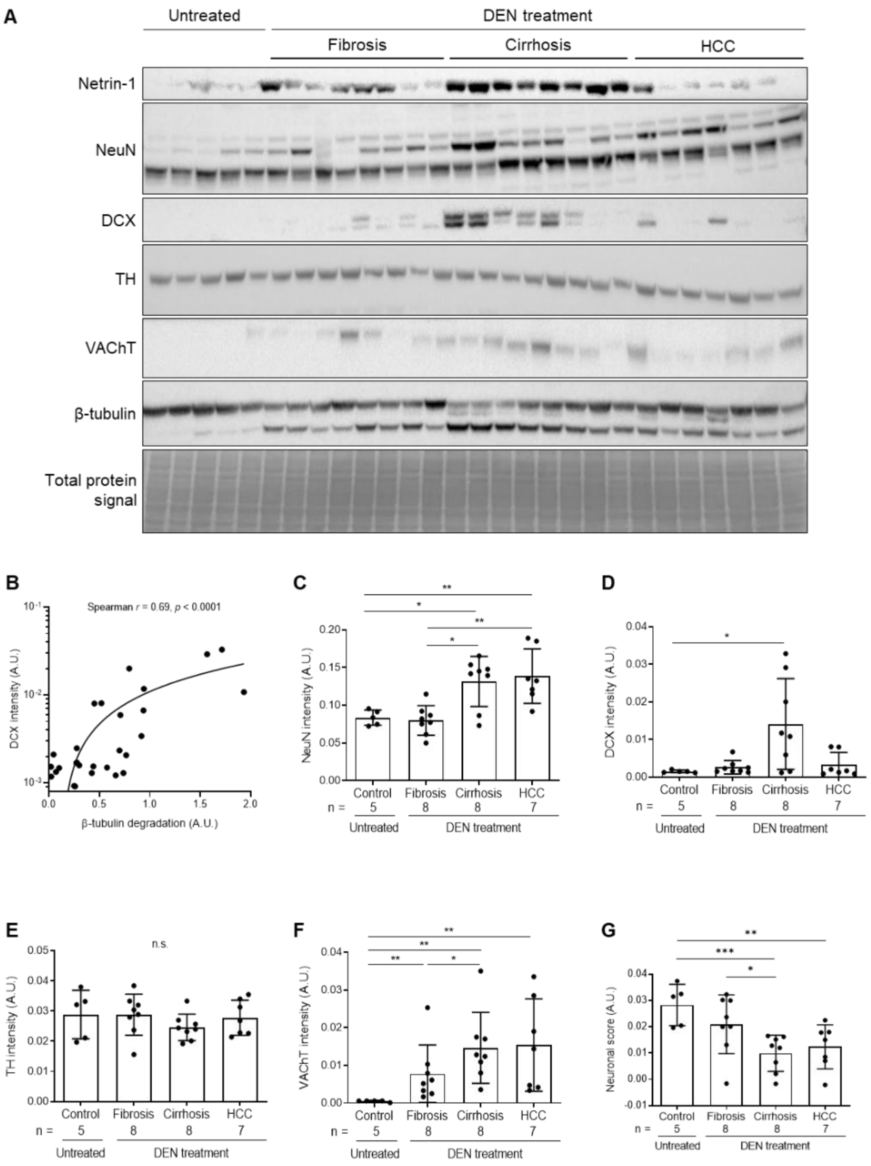

Fig. S4. Evolution towards HCC and hepatic remodeling are correlated with potential neurogenesis and cholinergic orientation in the cirrhotic rat. (**A**) Immunoblotting of netrin-1 (neurogenic factor), NeuN (mature neurons), DCX (immature neurons), TH (adrenergic) and VAChT (cholinergic) neural markers. (**B**) DCX induction is correlated with parenchymal remodeling. DCX levels were plotted against ratios of full-length versus degraded β-tubulin signals shown in panel B. Spearman test (*** $p<0.001$). (**C-G**) Signal quantification was done using the Fiji software on non-saturated images, using total protein normalization prior to statistical plotting using the Mann-Whitney or T-test (after normality test, * $p<0.05$, ** $p<0.01$, *** $p<0.001$). The NS is defined as the difference between TH and VAChT signals. Five to eight rats were used per time point. DCX, doublecortin; DEN, diethyl-nitrosamine; NeuN, neuronal nuclear antigen; TH, tyrosine hydroxylase; VAChT, vesicular acetylcholine transporter (*SLC18A3*).

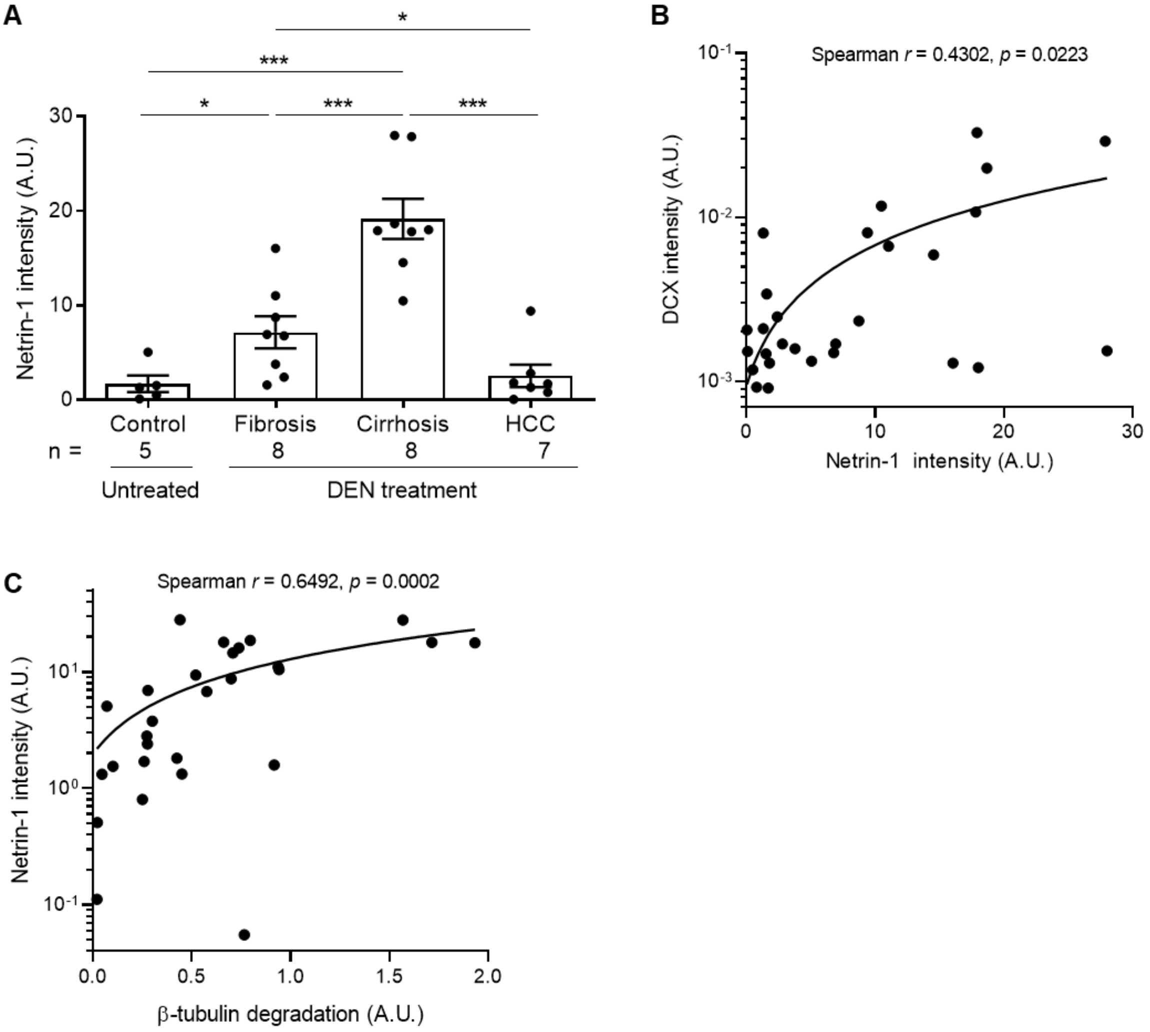

Fig. S5. Netrin-1 is correlated with neural remodeling, parenchymal remodeling and disease progression in the rat. (**A**) Netrin-1 signal quantification, Mann-Whitney test or T-test (after normality tests, * p<0.05, *** p<0.001). (**B**) Netrin-1 is correlated with DCX, Spearman test (* p<0.05). (**C**) Netrin-1 levels are correlated with parenchymal remodeling. Netrin-1 levels were plotted against ratios of full-length versus degraded β-tubulin signals blotted in **Fig. S4A**. Spearman test (* p<0.05, *** p<0.001). Quantification was done after total protein normalization. Five to eight identical rats to **Fig. S4** were used per time point. DCX, doublecortin; DEN, diethyl-nitrosamine.

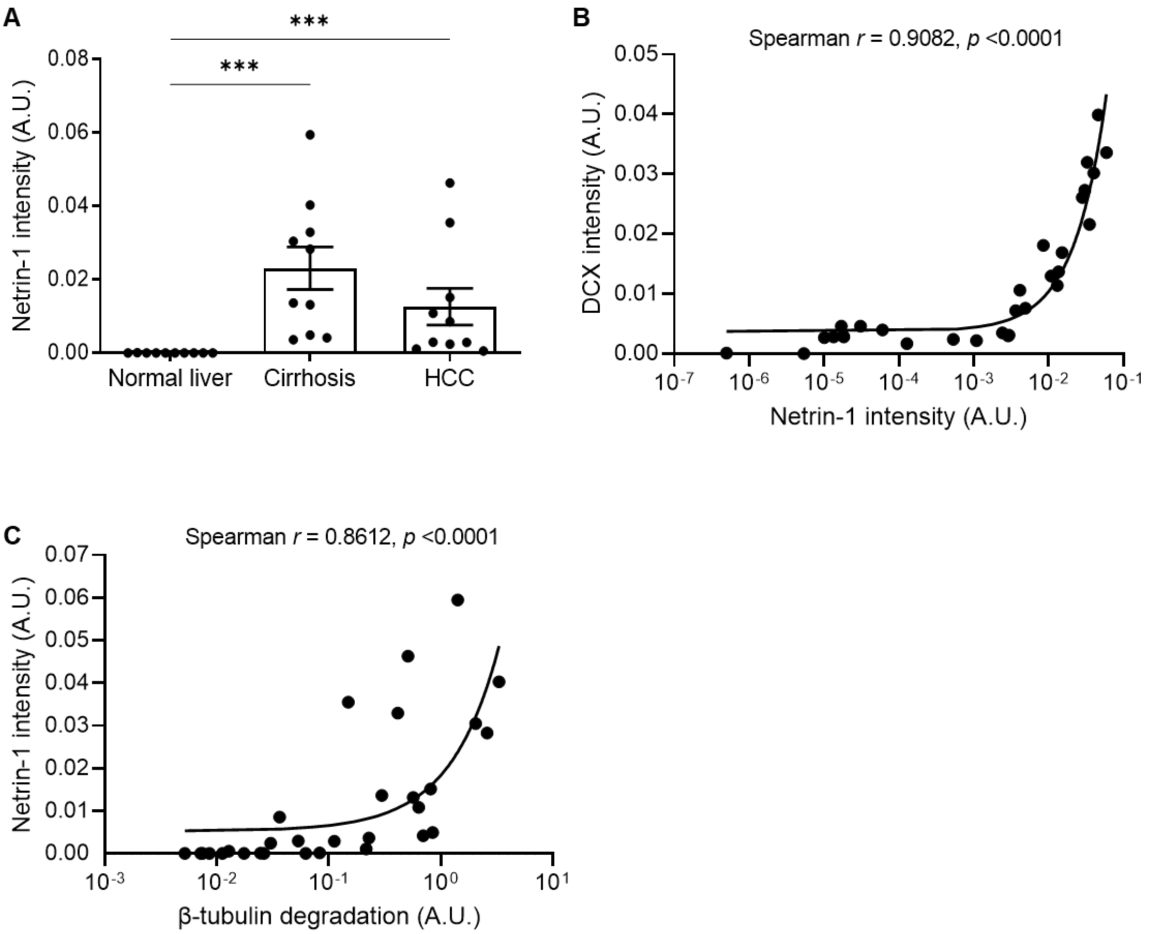

Fig. S6. Netrin-1 is correlated with neural remodeling, parenchymal remodeling and disease progression in patients. (**A**) Netrin-1 signal quantification and statistical plotting (Mann-Whitney test after normality test, *** p<0.001). (**B**) Netrin-1 is correlated with DCX, Spearman test (after normality test, *** p<0.001). (**C**) Netrin-1 induction is correlated with parenchymal remodeling. Netrin-1 levels were plotted against ratios of full-length versus degraded β-tubulin signals blotted in **Fig. 1A**, Spearman test (after normality test, *** p<0.001). Quantification was done after total protein normalization. DCX, doublecortin.

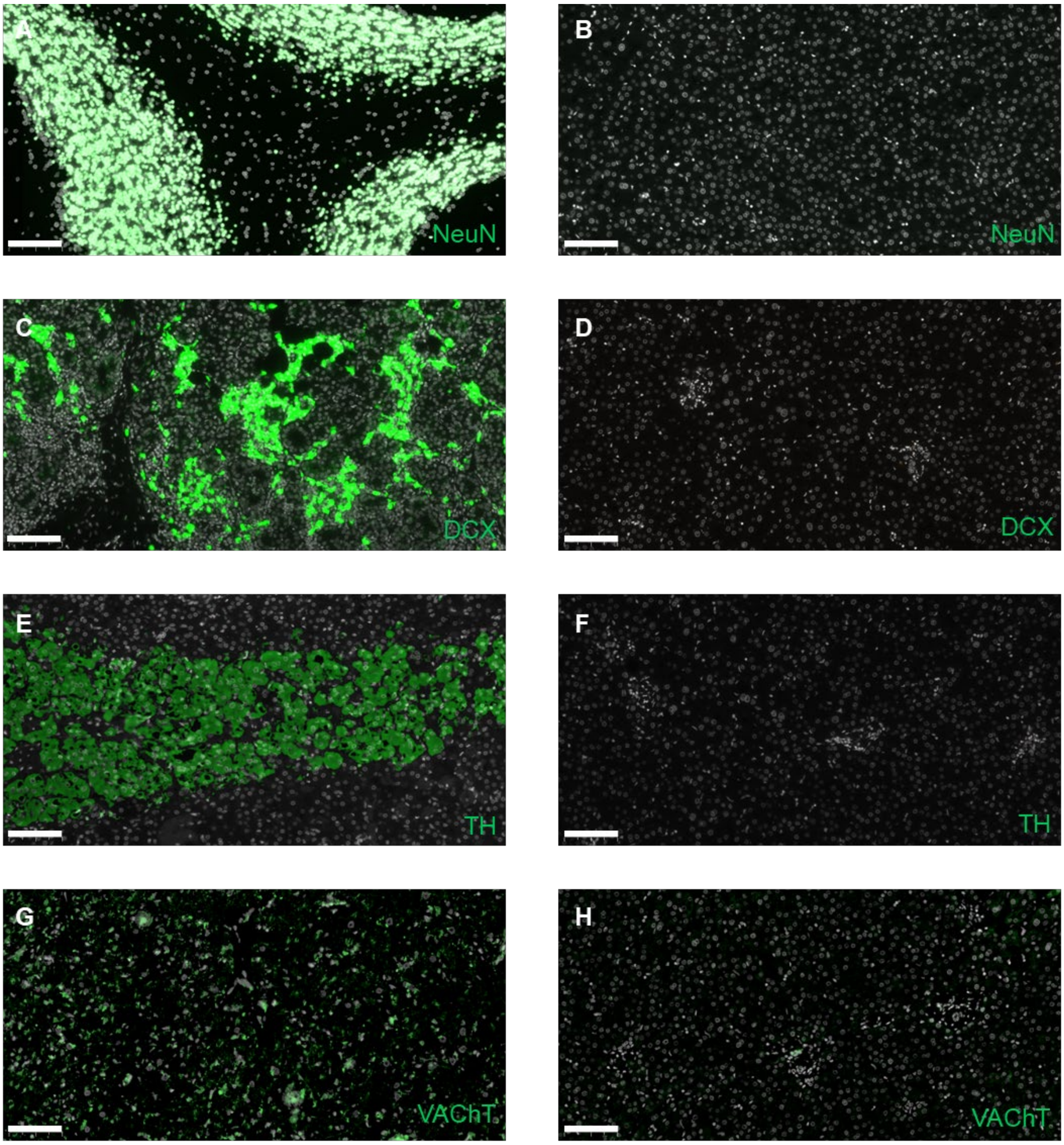

Fig. S7. Validation of antibodies used by IHC. Anti-NeuN, DCX, TH and VAChT antibodies were validated by immunofluorescence on *ad hoc* human tissue samples prior to their use on HCC specimens. (**A**) Cerebellum. (**C**) Parotid gland tumor. (**E**) Adrenal gland. (**G**) Cerebral cortex. (**B, D, F, H**) Normal liver (hepatocytic areas). In every panel, nuclei were stained with DAPI (grey signal). Antigens of interest are shown in green. Scale bar: 100 µm. DCX, doublecortin; NeuN, neuronal nuclear antigen; TH, tyrosine hydroxylase; VAChT, vesicular acetylcholine transporter (*SLC18A3*).

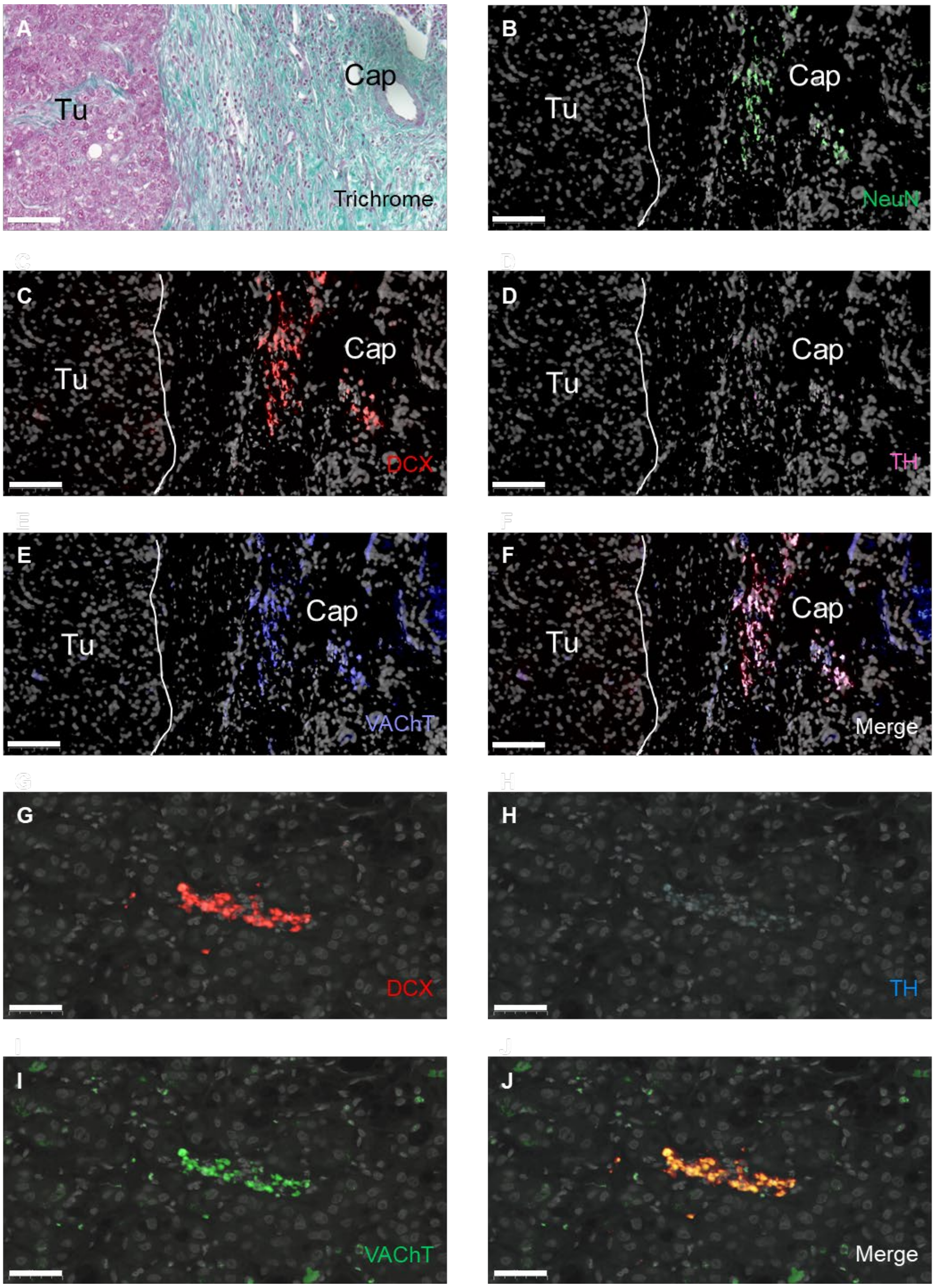

Fig. S8. Human HCC samples harbor NeuN⁺, DCX⁺, VAChT⁺ nucleated cells (IF approach). A panel of 24 HCC samples derived from all four main etiologies (HBV: n=7; HCV: n=4; ALD: n=9; NASH: n=4) was probed for immunolocalization of NeuN, DCX, TH and VAChT by multiplex IHC. (**A-F**) Masson's trichrome and IHC staining of a representative capsule-bearing tumor. (**G-J**) Staining of a representative tumor bulk. In every panel, DAPI-stained nuclei appear as grey. Scale bars: 50 µm. ALD, alcoholic liver disease; Cap, capsule; DCX, doublecortin; NASH, non-alcoholic steatohepatitis; NeuN, neuronal nuclear antigen; T, tumor; TH, tyrosine hydroxylase; Tu, tumoral bulk; VAChT, vesicular acetylcholine transporter (*SLC18A3*).

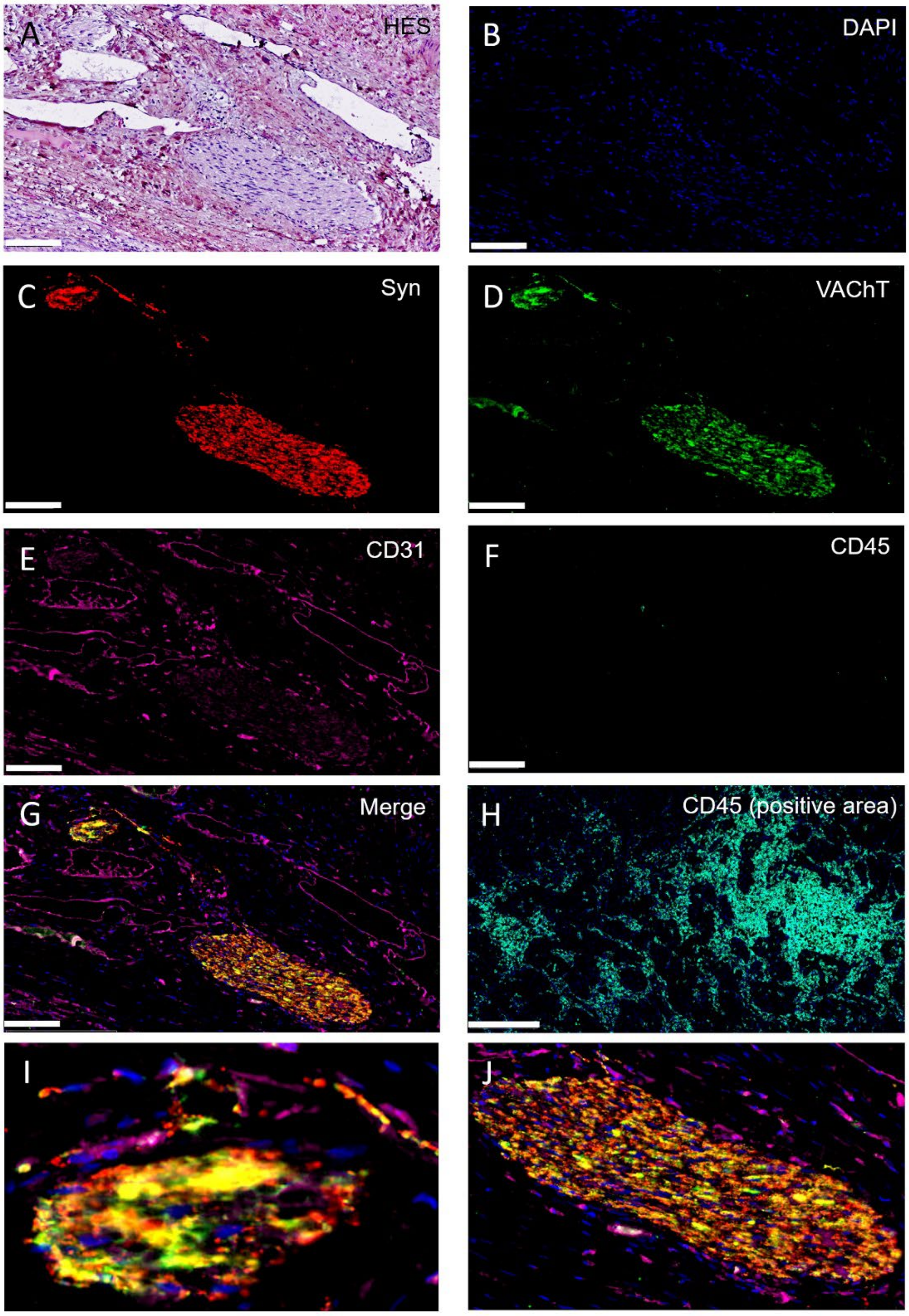

Fig. S9. Human HCC samples harbor synaptophysin$^+$, VAChT$^+$, CD45$^-$, CD31$^-$ nucleated cells (IF approach). Samples from the cohort described in **Fig. S8** showing (**A**) HES staining, (**B**) DAPI-stained nuclei, (**C**) Syn, (**D**) VAChT, (**E**) CD31 and (**F**) CD45. (**G**) Merged IF images. (**H**) Anti-CD45 Ab reactivity control sample + DAPI (HBV$^+$ liver tissue from the same cohort). Scale bars: 250 µm. (**I-J**) Focus on both structures of interest shown in (G). Syn, synaptophysin; VAChT, vesicular acetylcholine transporter (*SLC18A3*).

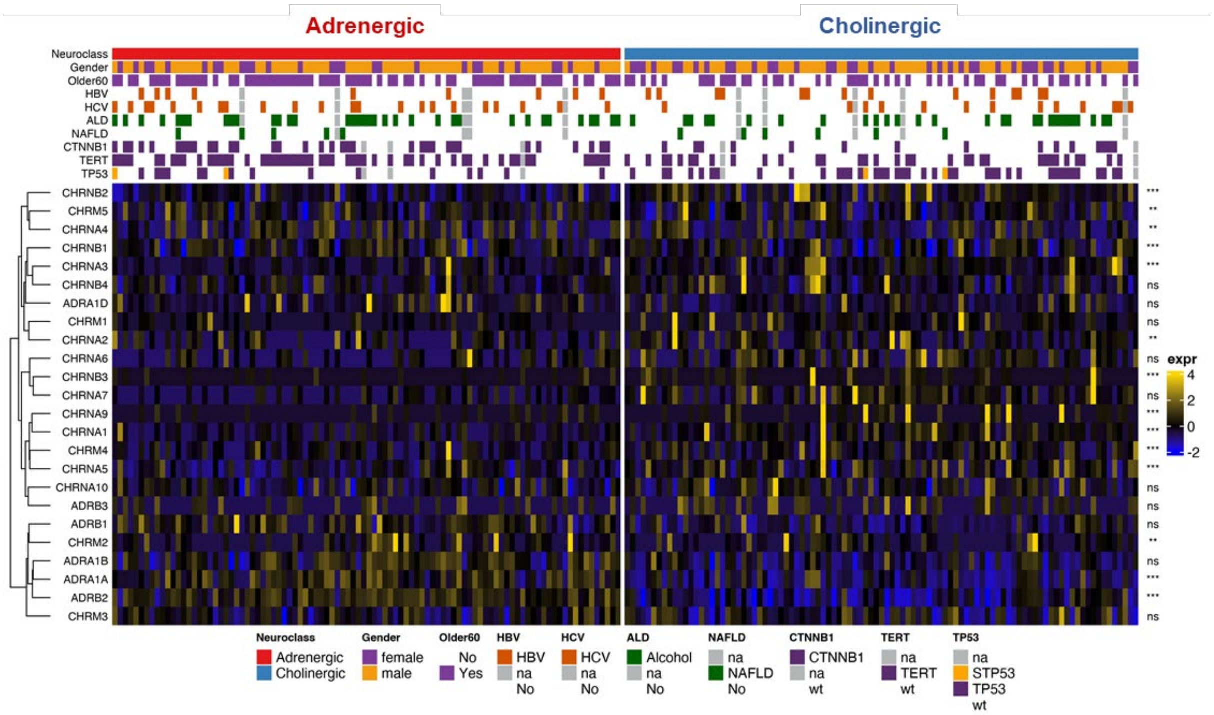

Fig. S10. Heatmap of relative expression levels of all adrenergic and cholinergic receptors in HCC tumor samples. Once samples were classified in cholinergic and adrenergic receptor expression classes based on previously obtained signature scores, differential expression analysis was performed and expression values normalized (variance stabilization transformation) and shown in the heatmap. T-test, ** $p<0.01$, *** $p<0.001$. Older60, patients older than 60; ALD, alcoholic liver disease; NAFLD, non-alcoholic fatty liver disease; Mutations: TP53, CTNNB1, TERT; Wt, wild type; Na, no information available.

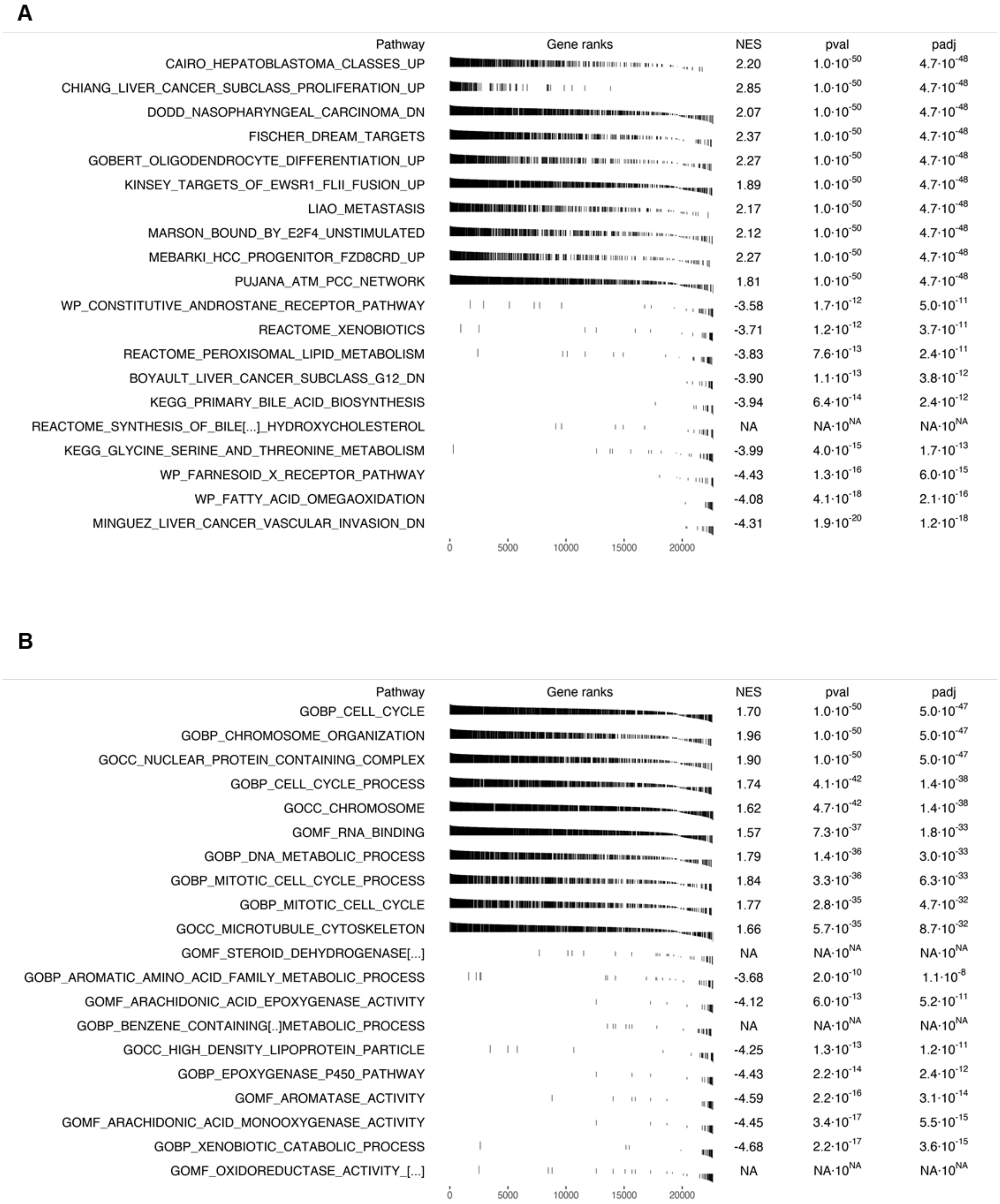

Fig. S11. Confirmation of pathway analysis results found on the Hallmark collection dataset by gene set enrichment analyses on the C2 and C5 gene sets of the MSigDB. (**A**) Adrenergic and (**B**) cholinergic samples.

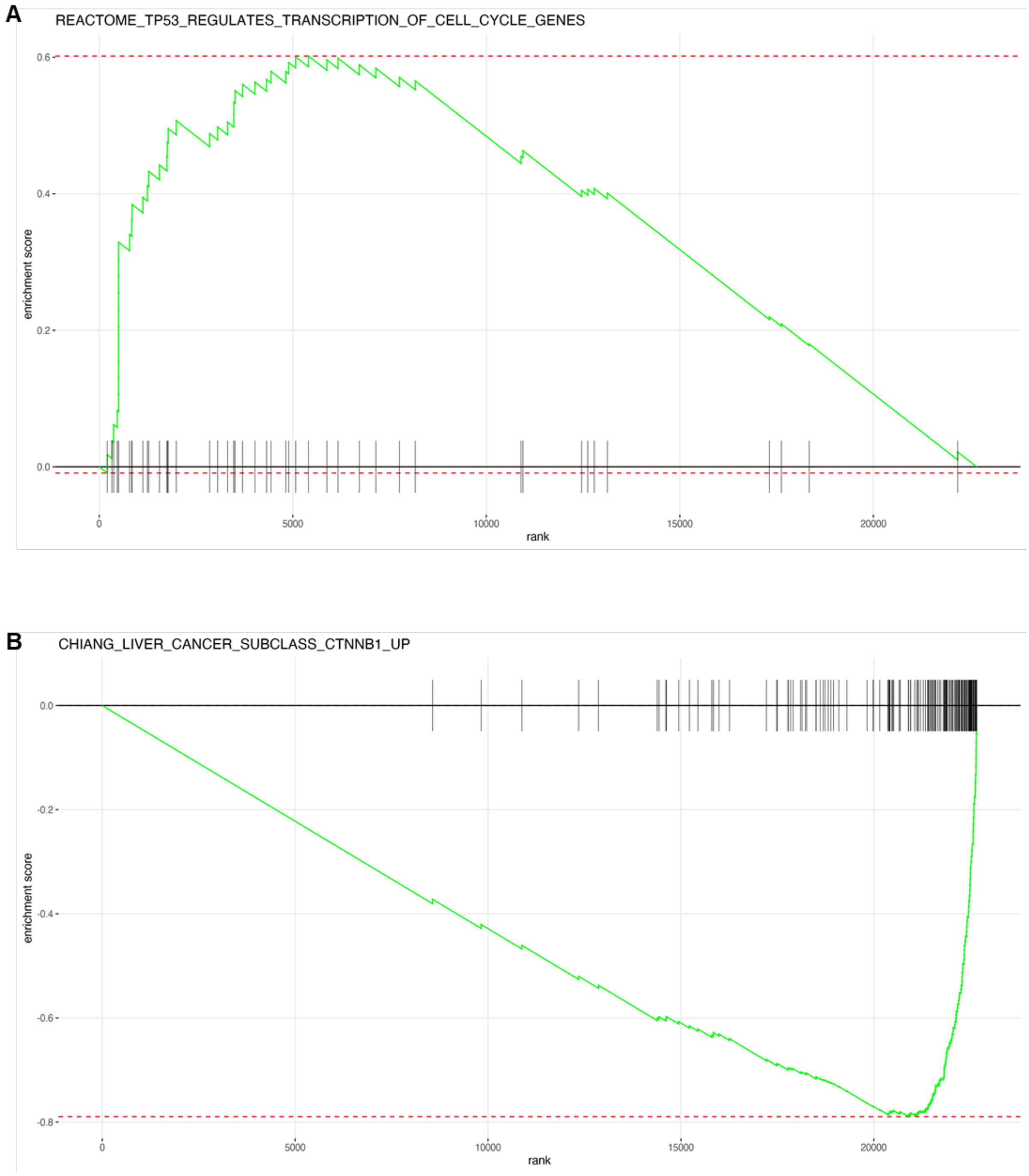

Fig. S12. Example of (A) enrichment in *TP53* mutations and of (B) depletion in *CTNNB1* mutation-associated metabolic functions in the cholinergic samples.

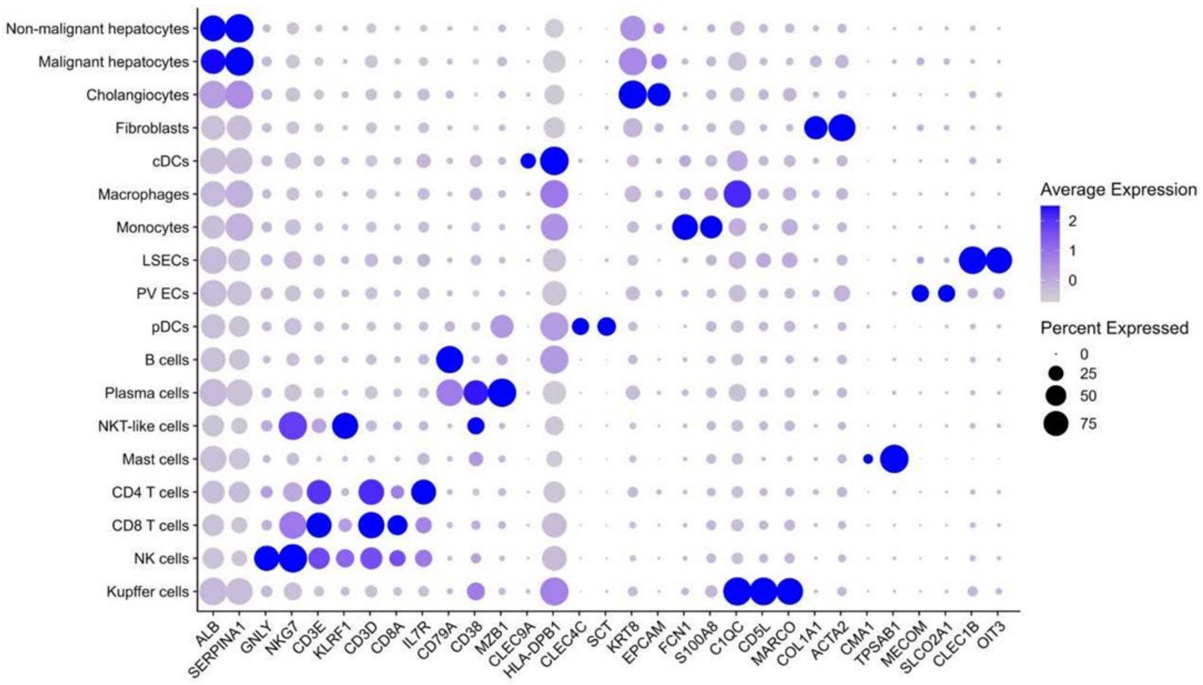

Fig. S13. Identification of hepatic cell types in HCC samples. Average expression levels and percentage of cells expressing canonical cell type markers in each of the 18 cell types identified. Data obtained from GSE149614 (n=10). cDCs, classical dendritic cells; NK, natural killer cells; LSECs, liver sinusoidal endothelial cells; PVECs, portal vein endothelial cells; pDCs, plasmacytoid dendritic cells.

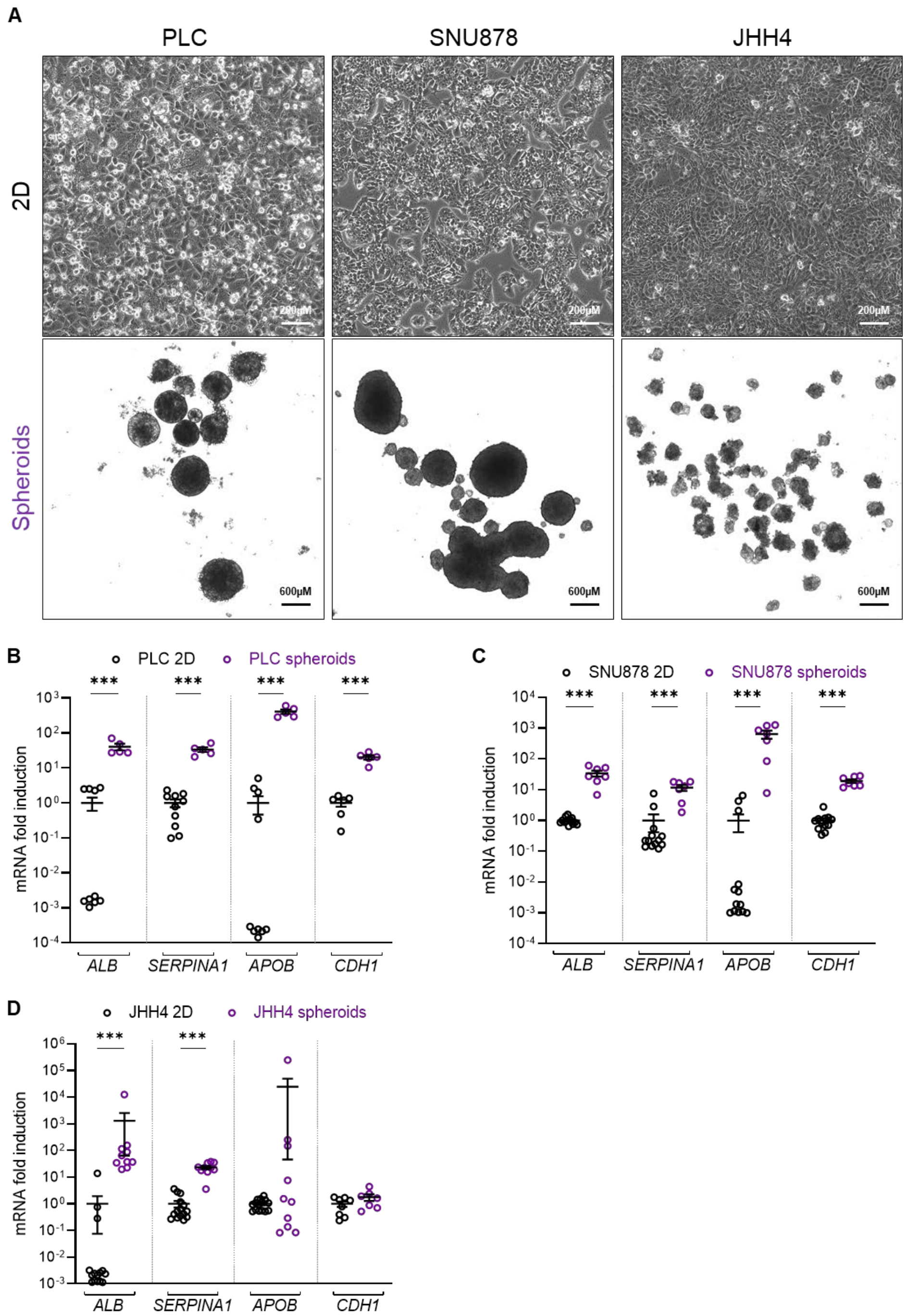

Fig. S14. Experimental validation of generated spheroids. (**A**) Corresponding micrographs of spheroids grown in the conditions described in Expanded materials. (**B-D**) Re-induction of HNF4α-controlled hepatocytic differentiation-related transcripts upon spheroid formation (n≥5 independent experiments for each cell line, Mann-Whitney test after normality test, *** p<0.001). ALB, albumin; APOB, apolipoprotein B; CDH1, cadherin 1; HNF4α, hepatocyte nuclear factor 4 alpha; SERPINA1, serpin family A member 1.

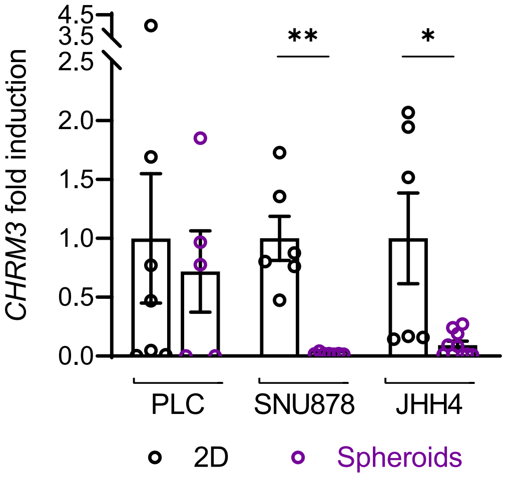

Fig. S15. Experimental re-differentiation represses *CHRM3* expression. PLC, SNU878, and JHH4 spheroids were analyzed by RT-qPCR. Mann-Whitney test or T-test (depending on normality, * p<0.05, ** p<0.01), (n≥5 independent experiments for each cell line). CHRM3, cholinergic receptor muscarinic 3.

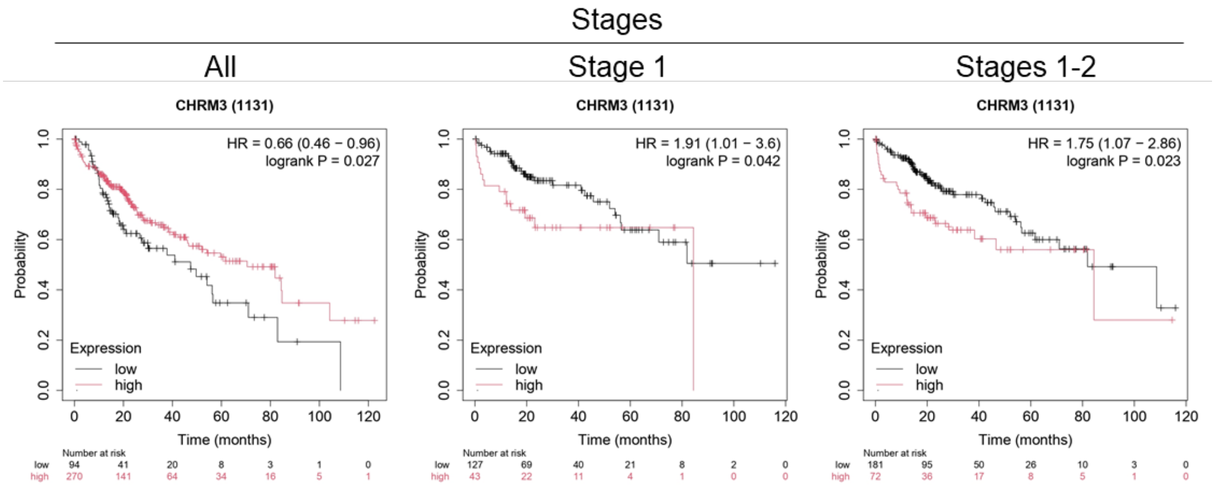

Fig. S16. High *CHRM3* predicts shorter survival in stages 1-2 HCC. Log-Rank test and Kaplan-Meier plot on the association between *CHRM3* expression (defined as below and above median) and survival. Left panel: all TCGA LIHC patients considered. Center panel: data derived from Stage 1 patients. Right panel: data derived from Stage 1-2 patients. Log-rank p<0.05.

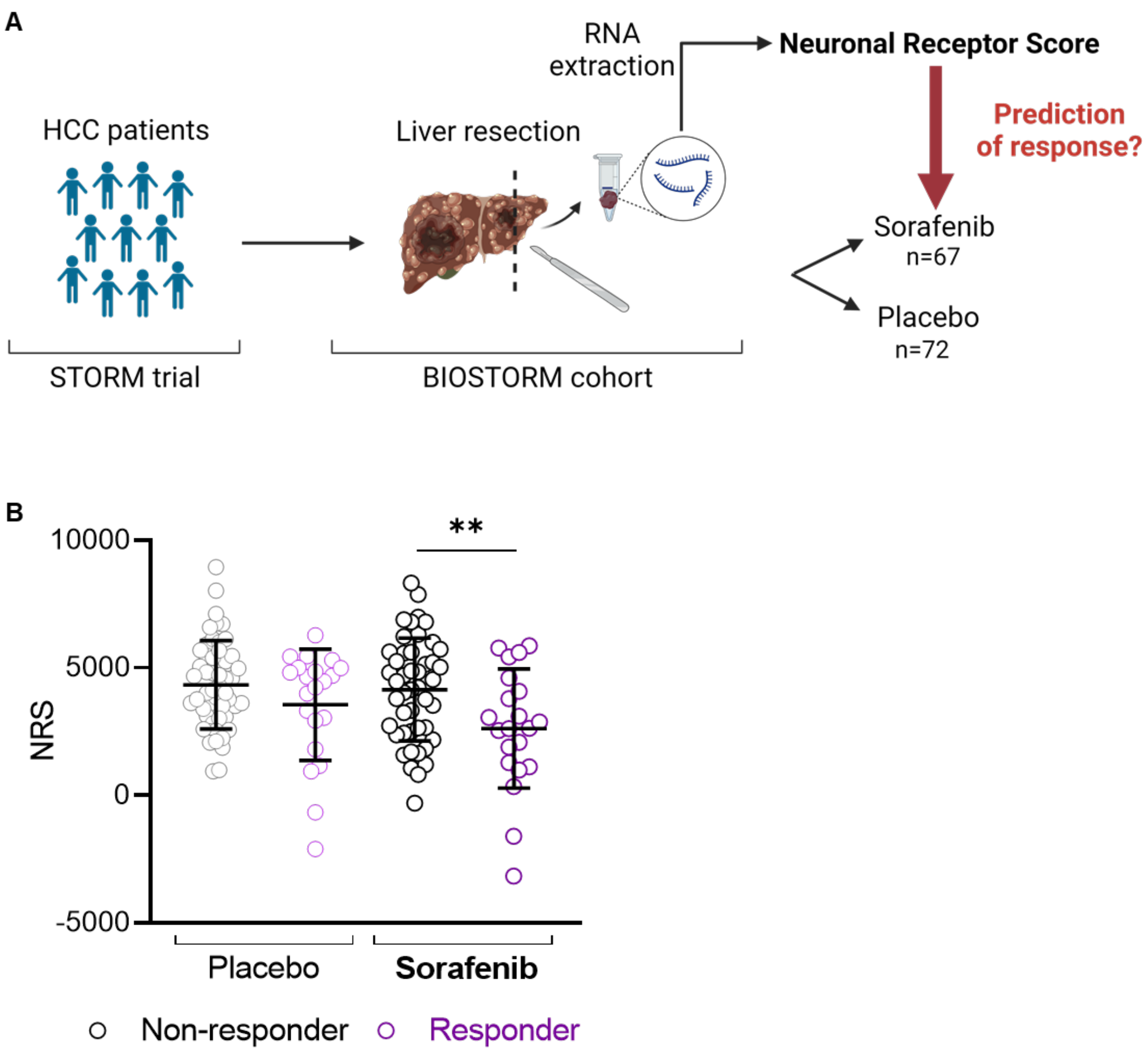

Fig. S17. Cholinergic orientation (low NRS) predicts response to sorafenib in the STORM (BIOSTORM cohort) trial. (**A**) Description of the experimental approach. Created with BioRender. (**B**) Comparison of NRS in tumor tissues from placebo non-responders to treatment (n=51), placebo responders (n=21), sorafenib non-responders (n=46) and sorafenib responders (n=21). Groups were defined in the original study based on objective response criteria [15]. Kruskal-Wallis test (GSE109211) after normality test (** p<0.01). Bars represent mean ± SD. NRS, neuronal receptor score.

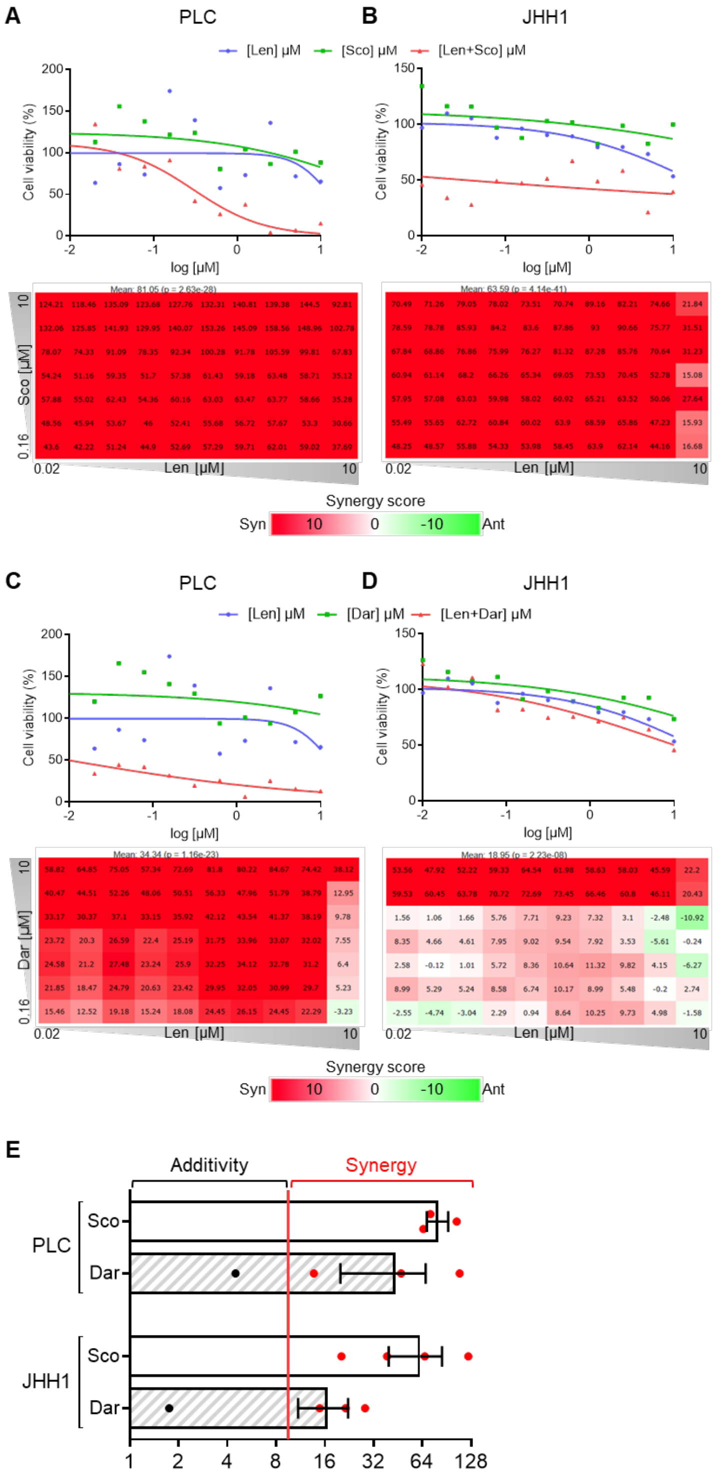

Fig. S18. Muscarinic blockade synergizes with the standard-of-care TKI lenvatinib. (**A-D**) Sigmoid activity curves and 2D matrices on (**A-B**) scopolamine and (**C-D**) darifenacin, respectively on PLC (class 1) and JHH1 (class 3) HCC lines. Chou-Talalay ZIP scores >10 indicate synergy. (**E**) Plate-wide average ZIP score values calculated from two separate 96-well matrices for each experiment. Sigmoids are representative of n=3-5 independent experiments. Dar, darifenacin; Len, lenvatinib; Sco, scopolamine.

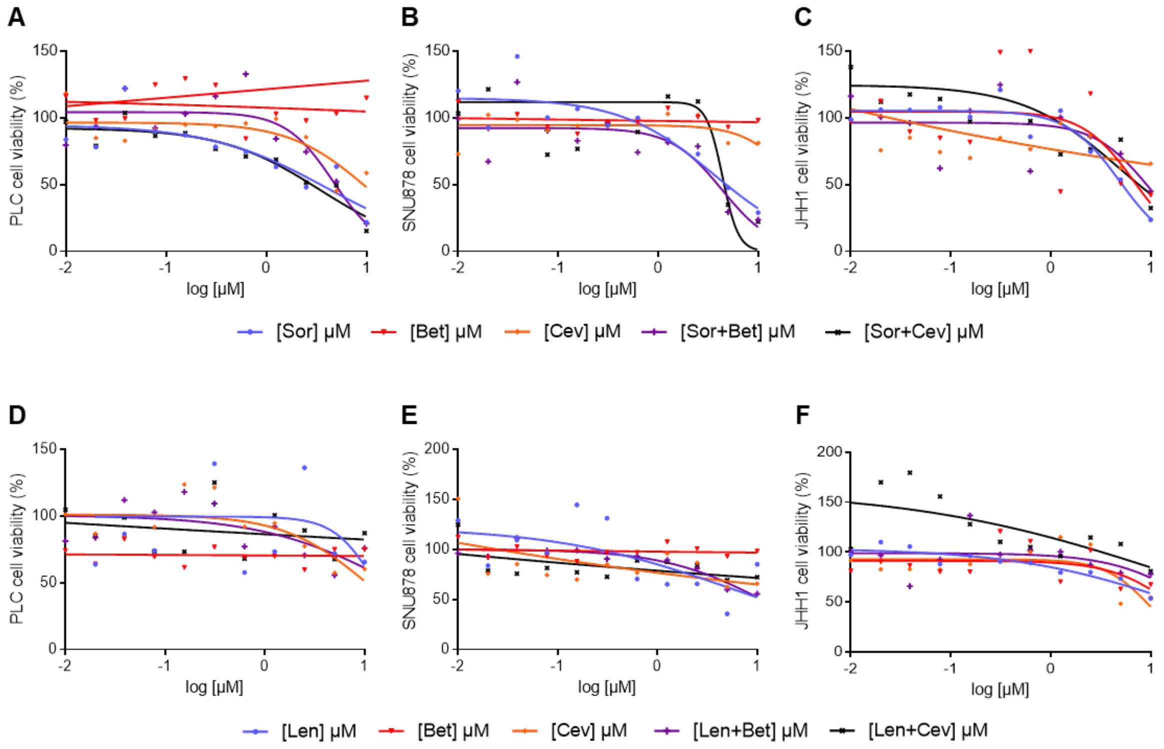

Fig. S19. Muscarinic agonists bethanechol and cevimeline do not affect TKI activities. (**A-F**) Activity curves of sorafenib (**A-C**) and lenvatinib (**D-F**), respectively on PLC (class 1), SNU878 (class 2) and JHH1 (class 3) treated HCC lines. Sigmoids are representative of n=3 independent experiments. Cev, cevimeline; Len, lenvatinib; Sor, sorafenib; TKI, tyrosine kinase inhibitor.

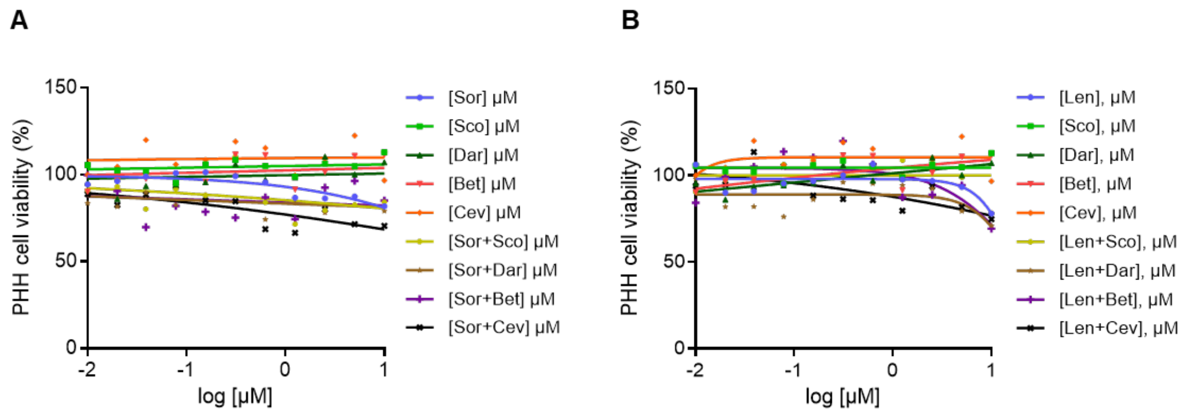

Fig. S20. Muscarinic targeting does not affect viability of PHH with or without TKI combination. (**A-B**) Activity curves of sorafenib and lenvatinib, respectively. n=3 independent experiments carried out on PHH, each derived from one distinct patient. Cev, cevimeline; Dar, darifenacin; Len, lenvatinib; PHH, primary human hepatocytes; Sco, scopolamine; Sor, sorafenib; TKI, tyrosine kinase inhibitor.

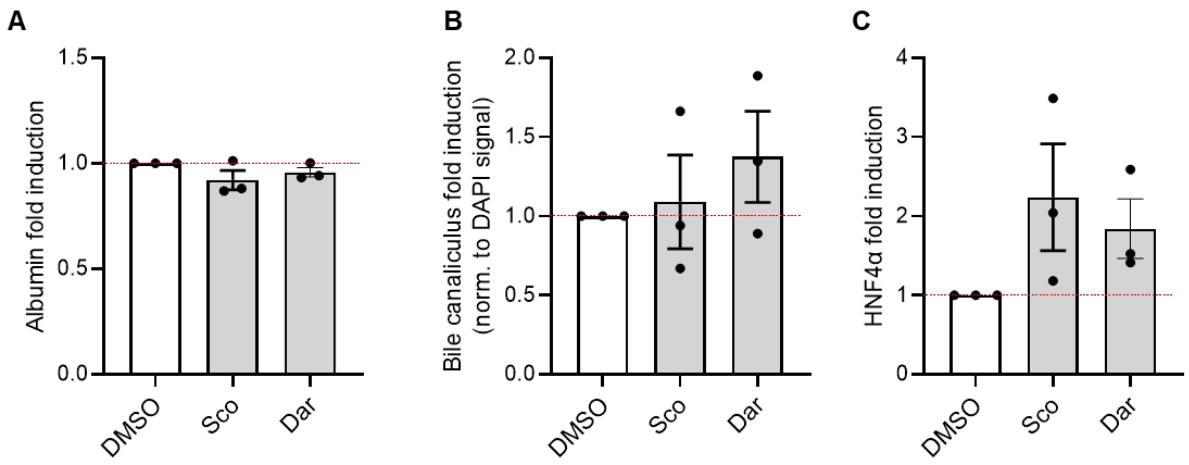

Fig. S21. Muscarinic targeting preserves mature hepatocytic functions. (**A**) Secreted human albumin quantified by Elisa. (**B**) Bile canaliculus density alterations normalized against DAPI signal. (**C**) Changes in HNF4α DNA binding activity. T-test after normality test. n.s. Cells underwent 28 days of differentiation. n=3 independent experiments. Dar, darifenacin; HNF4α, hepatocyte nuclear factor 4 alpha; Sco, scopolamine.

SUPPLEMENTARY TABLES

Table S1. Characteristics of paired F4/HCC samples used in the study. Samples were obtained from the French liver biobank network. Median ± SD is shown for all qualitative data.

Variable	Available data (Total: 166)	Values
**Demographics**	166	
Age, *y* ± SD	166	69 ± 10
Gender (male/female)	166	140 (84%)/26 (16%)
**Etiology (%)**	166	
Alcoholic liver disease		36 (21%)
Hepatitis B virus		46 (28%)
Hepatitis C virus		39 (24%)
Non-alcoholic steato-hepatitis		45 (27%)
**Liver disease parameters**		
Liver fibrosis score METAVIR (F4) (%)	166	166 (100%)
Inflammation activity score (%)	137	
METAVIR 0		51 (37%)
METAVIR 1		55 (40%)
METAVIR 2		23 (17%)
METAVIR 3		8 (6%)
Serum alpha-fetoprotein, >100 ng/mL	111	17 (16%)
Child-Pugh score (A/B/C) (%)	87	72 (83%)/9 (11%)/6 (6%)
Prothrombin, % ± SD	146	81 ± 22
Bilirubin, µMol/L ± SD	137	14 ± 75
Albumin, g/L ± SD	100	36 ± 7
Platelet count, G/L ± SD	149	131 ± 102
Encephalopathy (%)	164	18 (11%)
Ascites (%)	164	37 (3%)
Jaundice (%)	162	22 (14%)
Esophageal varices (%)	156	62 (40%)
**Tumors characteristics**		
Tumor size, *mm* ± SD	165	34 ± 31
Tumor localization (left liver/right liver/double)	165	47 (29%)/112 (68%)/6 (4%)
Intact tumor capsule (%)	143	91 (64%)
Satellite nodules (%)	165	34 (21%)
Macrovascular invasion, Microvascular invasion (%)	151	22 (15%), 81 (52%)
Differentiation grade (poor/moderately/well) (%)	165	15 (9%)/74 (45%)/76 (46%)
Architectural pattern (%)	136	
Trabecular		104 (76%)
Pseudoglandular		10 (7%)
Compact		5 (4%)
Clear cells		4 (3%)
Others		13 (9%)

Tumoral steatosis (%)	165	108 (66%)
Tumoral necrosis (%)	150	84 (56%)

Table S2. Significance assessment of neural features in HCC. Wilcoxon matched-pairs signed rank test was used (* p<0.05, ** p<0.01).

Evolution of values from cirrhosis to HCC (paired samples)						
	DCX	NEUN	INA	TH	VAChT	**Neuronal score (TH-VACHT)**
HBV (n=14)	↗*	↗*				
HCV (n=9)				↘**		
ALCOHOL (n=14)						
NASH (n=14)				↘**		
**All etiologies (n=51)**		↗*		↘**		↘**

Table S3 (Excel file). List of adrenergic and cholinergic receptors expressed in TCGA HCC samples. Their differential gene expression grouped by neural class was analyzed by the DESeq2 method.

Table S4 (Excel file). Association levels between neural classes and major HCC-related clinico-biological variables. Fisher test was used; TCGA cohort.

Table S5. Association levels between neural classes and major HCC-related clinico-biological variables. Fisher test was used; validation cohort.

**Variable 1**	**p-value**
Gender	0.1628
Older60	0.3646
HBV	0.6338
HBV / HDV	0.8315
HCV	0.6389
ALD	0.3697
CTNNB1	0.0562
TP53	0.5948

Table S6 (Excel file). List of the 100 genes most associated with the adrenergic class. Log2foldchange: alteration levels considering the cholinergic class as a reference. Lfcse: Log2foldchange of standard error. padj: adjusted p-value.

Table S7 (Excel file). List of the 100 genes most associated with the cholinergic class. Log2foldchange: alteration levels considering the adrenergic class as a reference. Lfcse: Log2foldchange of standard error. padj: adjusted p-value.

Table S8. Main differentially expressed genes of interest in adrenergic and cholinergic TCGA LIHC samples.

	Gene symbol	Log2 fold change	Function	PMID
Up-regulated in the cholinergic class	*LGALS14*	8	T-cell apoptosis related antigen	12678492
	CT55	6	Cancer testis antigen	35189384
	BMP7	6	TGF-β family growth factor	12808448
	CEACAM7	5	Carcinoembryonic antigen-related cell adhesion molecule 7	22195770
	MAGEA4, MAGEA10	4	HCC progression drivers	34166362
	XAGE2	4	Fetal/reproductive tissue tumor-related antigen	11992404
	GAGE2A	3	Germ-cell and tumor antigen	23029259
	AFP	4	HCC diagnosis-related antigen	32923383
Up-regulated in the adrenergic class	*ALDH3A1*	3	Xenobiotic-inducible hepatocytic differentiation markers	27279633
	CYP1A1, 3A4, 1A2 *CYP2A13, 2A7P1, 3F36P*	2 to 3		20645049

Table S9 (Excel file). Pathways enriched in the adrenergic class. Over Representation Analysis with hypergeometric test. $p<0.01$. Besides signature names listed in this table ($p<0.01$ Wilcoxon test), attention needs to be paid to their expanded biological significance extracted from the ssGSEA database before drawing conclusions with respect to pathology.

Table S10 (Excel file). Pathways enriched in the cholinergic class. Over Representation Analysis with hypergeometric test. $p<0.01$. Besides signature names listed in this table ($p<0.01$ Wilcoxon test), attention needs to be paid to their expanded biological significance extracted from the ssGSEA database before drawing conclusions with respect to pathology.

Table S11. Biological descriptions of prognosis pathways associated with both HCC neuroclasses. Besides signature names listed in this table, attention needs to be paid to their expanded biological significance extracted from the ssGSEA database before drawing conclusions with respect to pathology.

Standard name	Brief description	Systematic name
ANDERSEN_LIVER_CANCER_KRT19_DN	Genes under-expressed in KRT19-positive [GeneID=3880] hepatocellular carcinoma.	M424
BOYAULT_LIVER_CANCER_SUBCLASS_G1_DN	Down-regulated genes in hepatocellular carcinoma (HCC) subclass G1, defined by unsupervised clustering	M1883
BOYAULT_LIVER_CANCER_SUBCLASS_G12_DN	Down-regulated genes in hepatocellular carcinoma (HCC) subclass G12, defined by unsupervised clustering	M12228
BOYAULT_LIVER_CANCER_SUBCLASS_G123_DN	Down-regulated genes in hepatocellular carcinoma (HCC) subclass G123, defined by unsupervised clustering.	M2218
BOYAULT_LIVER_CANCER_SUBCLASS_G6_UP	Up-regulated genes in hepatocellular carcinoma (HCC) subclass G6, defined by unsupervised clustering.	M4342
CHIANG_LIVER_CANCER_SUBCLASS_CTNNB1_DN	Top 200 marker genes down-regulated in the 'CTNNB1' subclass of hepatocellular carcinoma (HCC); characterized by activated CTNNB1 [GeneID=1499].	M8689
CHIANG_LIVER_CANCER_SUBCLASS_CTNNB1_UP	Top 200 marker genes up-regulated in the 'CTNNB1' subclass of hepatocellular carcinoma (HCC); characterized by activated CTNNB1 [GeneID=1499].	M16496
CHIANG_LIVER_CANCER_SUBCLASS_INTERFERON_DN	All marker genes down-regulated in the 'interferon' subclass of hepatocellular carcinoma (HCC).	M14353

CHIANG_LIVER_CANCER_SUBCLASS_POLYSOMY7_UP	Marker genes up-regulated in the 'chromosome 7 polysomy' subclass of hepatocellular carcinoma (HCC); characterized by polysomy of chromosome 7 and by a lack of gains of chromosome 8q.	M834
CHIANG_LIVER_CANCER_SUBCLASS_PROLIFERATION_DN	Top 200 marker genes down-regulated in the 'proliferation' subclass of hepatocellular carcinoma (HCC); characterized by increased proliferation, high levels of serum AFP [GeneID=174], and chromosomal instability.	M16932
CHIANG_LIVER_CANCER_SUBCLASS_PROLIFERATION_UP	Top 200 marker genes up-regulated in the 'proliferation' subclass of hepatocellular carcinoma (HCC); characterized by increased proliferation, high levels of serum AFP [GeneID=174], and chromosomal instability.	M3268
DESERT_PERIPORTAL_HEPATOCELLULAR_CARCINOMA_SUBCLASS_UP	Genes up-regulated in the periportal-type subclass of hepatocellular carcinomas.	M34031
DESERT_STEM_CELL_HEPATOCELLULAR_CARCINOMA_SUBCLASS_UP	Genes up-regulated in the stem cell-type subclass of hepatocellular carcinomas.	M34034
HOSHIDA_LIVER_CANCER_LATE_RECURRENCE_DN	Genes whose expression correlated with lower risk of late recurrence of hepatocellular carcinoma (HCC).	M13658
HOSHIDA_LIVER_CANCER_SUBCLASS_S1	Genes from 'subtype S1' signature of hepatocellular carcinoma (HCC): aberrant activation of the WNT signaling pathway.	M5311
HOSHIDA_LIVER_CANCER_SUBCLASS_S3	Genes from 'subtype S3' signature of hepatocellular carcinoma (HCC): hepatocyte differentiation.	M1286
HOSHIDA_LIVER_CANCER_SURVIVAL_DN	Survival signature genes defined in adjacent liver tissue: genes correlated with good survival of hepatocellular carcinoma (HCC) patients.	M5451
HOSHIDA_LIVER_CANCER_SURVIVAL_UP	Survival signature genes defined in adjacent liver tissue: genes correlated with poor survival of hepatocellular carcinoma (HCC) patients.	M6939
KIM_LIVER_CANCER_POOR_SURVIVAL_DN	Genes under-expressed in hepatocellular carcinoma (HCC) with poor survival	M534
LEE_LIVER_CANCER_SURVIVAL_DN	Genes highly expressed in hepatocellular carcinoma with worse survival.	M7987
LEE_LIVER_CANCER_SURVIVAL_UP	Genes highly expressed in hepatocellular carcinoma with better survival.	M6145
VILLANUEVA_LIVER_CANCER_KRT19_DN	Genes under-expressed in KRT19-positive [GeneID=3880] hepatocellular carcinoma (HCC).	M373

VILLANUEVA_LIVER_CANCER_KRT19_UP	Genes over-expressed in KRT19-positive [GeneID=3880] hepatocellular carcinoma (HCC).	M336
WOO_LIVER CANCER RECURRENCE_DN	Genes negatively correlated with recurrence free survival in patients with hepatitis B-related (HBV) hepatocellular carcinoma (HCC).	M9911
WOO_LIVER_CANCER_RECURRENCE_UP	Genes positively correlated with recurrence free survival in patients with hepatitis B-related (HBV) hepatocellular carcinoma (HCC).	M12602
YAMASHITA_LIVER_CANCER_STEM_CELL_DN	Genes down-regulated in hepatocellular carcinoma (HCC) cells with hepatic stem cell properties.	M9206

Table S12 (Excel file). Association of adrenergic and cholinergic tumors with canonical HCC signatures. Besides signature names listed in this table, attention needs to be paid to their expanded biological significance extracted from the ssGSEA database before drawing conclusions with respect to pathology (padj<0.001 Fischer test).

Table S13. Suitability of HCC lines for the approaches used in this study. Classes refer to the 2019 Caruso transcriptomic classification of HCC lines.

	**Subgroups**				
	**Cl. 1**	**Cl. 2**		**Cl. 3**	
	**PLC**	**SNU878**	**HepaRG**	**JHH1**	**JHH4**
**Spheroids**	suitable	suitable	suitable	no growth	suitable
**Soft Agar**	suitable	suitable	suitable	no growth	no growth
**Anoikis**	suitable	suitable	suitable	no growth	suitable

SUPPLEMENTAL INFORMATION 1

Validation of anti-NeuN, DCX, INA, TH and VAChT antibodies by Western Blot.

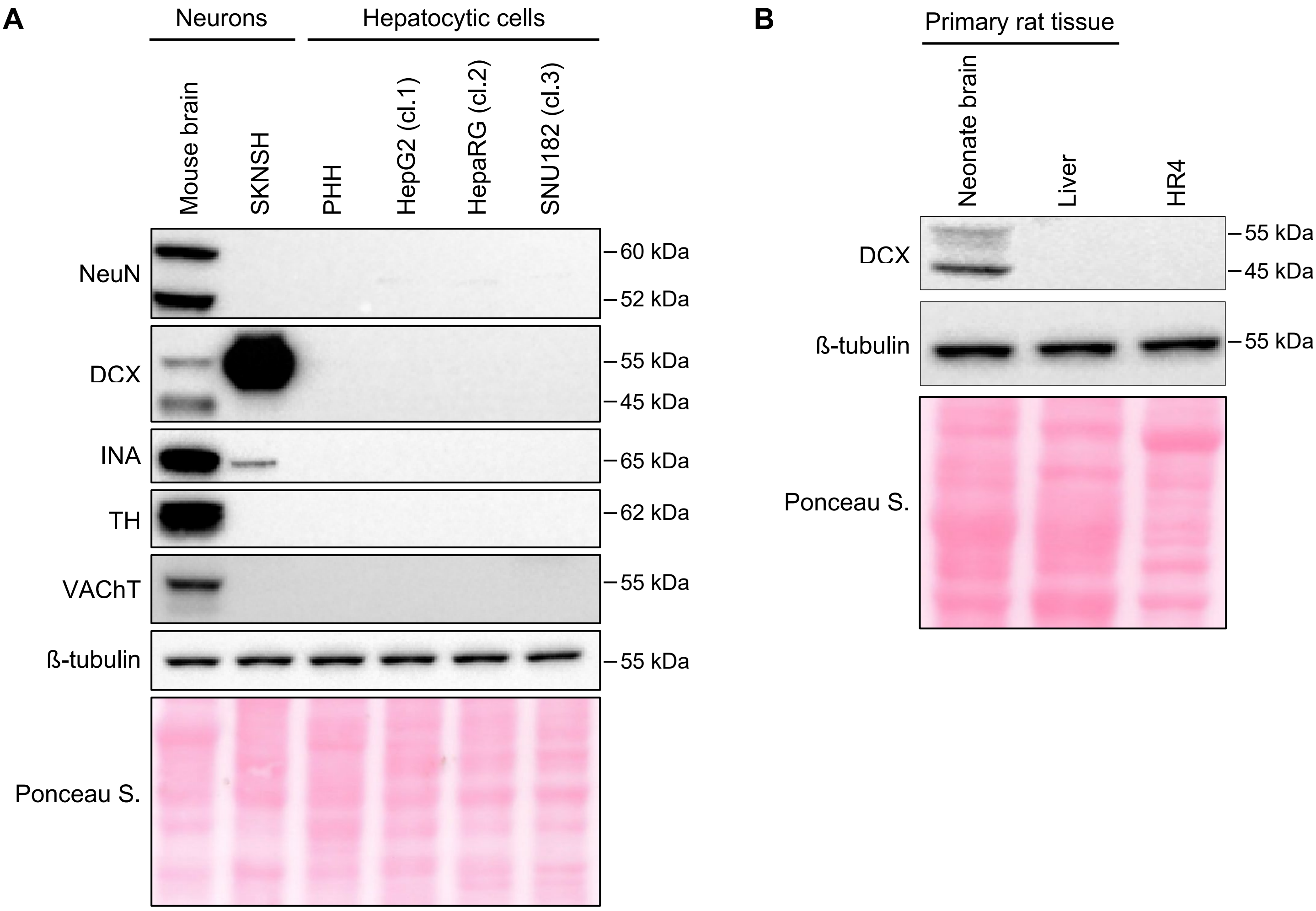

Fig. S22. Validation of anti-NeuN, DCX, INA, TH and VAChT antibodies by Western Blot. (**A**) Extracts from neonate mouse brain, SKNSH, PHH, and human HCC lines belonging to transcriptomic classes 1, 2 and 3 were processed for detection of the indicated targets. (**B**) The same strategy was used for the validation of an anti-DCX antibody specifically suitable for rat epitopes. DCX, doublecortin; INA, internexin neuronal intermediate filament protein alpha; NeuN, neuronal nuclear antigen; PHH, primary human hepatocytes; TH, tyrosine hydroxylase; VAChT, vesicular acetylcholine transporter (*SLC18A3*).

SUPPLEMENTARY INFORMATION 2

Neurogenesis of cholinergic orientation in a cirrhosis-associated HCC rat model

HCC occurs in a cirrhotic background in 80% of cases. A rat model that reproduces the development of HCC from cirrhosis, via DEN treatment, has been extensively characterized [16] (also in **Fig. S3**) and shows documented clinical relevance in particular with respect to the proliferative class of HCC [17]. In this context, we herein evaluated the ability of this DEN-treated HCC rat model to recapitulate such neural, cancer-related, processes [18-22]. The methodology used for the experimental induction of HCC is shown in **Fig. S3A**. Total NeuN signals increased throughout disease progression. Interestingly, signals related to the DCX progenitor marker increased transiently, yet sharply, in samples harboring cirrhosis and small HCC nodules (**Fig. S3B** and **Fig. S4A**). In the case of autonomic nervous system (ANS)-specific markers, as in clinical samples, an increase in the cholinergic VAChT marker was observed in rats suffering from HCC. Unlike in humans, levels of the TH marker (adrenergic) remained unaltered throughout disease progression, while being of less functional importance than in the clinic because of microanatomical reasons [23]. Degradation of β-tubulin was correlated with DEN-treatment and was likely derived from hepatic cytolysis and release of cytosolic contents (**Fig. S4A**). Consistently, a tight correlation was observed between DCX expression and β-tubulin degradation throughout progression of HCC-predisposing chronic liver disease (CLD) (**Fig. S4B**). This suggests that HCC neural remodeling occurs as a consequence of cytolysis or parenchymal remodeling, as recently demonstrated in steato-hepatitis [24]. This prompted us to analyze the quantitative evolution of neural markers at different stages of liver injury: fibrosis, cirrhosis and HCC. The results confirmed neo-neurogenesis, and the cholinergic neural features of HCC in the rat (**Fig. S4C-F**). The ANS oscillates between two opposite polarities, prompting for the use of a unified immunoreactive score. Accordingly, as for human samples, the neuronal score (NS) was defined as the difference between adrenergic and cholinergic signals (see Methods, NS = TH - VAChT). The NS provides integrated estimation of ANS inputs to the liver, and decreased with disease progression (**Fig. S4G**), indicating evolution towards cholinergic functions. Interestingly, netrin-1, a neurogenic protein which is frequently upregulated in CLD [25-27], was sharply induced with disease progression in this model and was correlated with β-tubulin degradation and DCX induction (**Fig. S5**). Altogether, such data indicate that alteration of hepatic neural features and their cholinergic orientation is a novel signature of progression towards HCC *in vivo* in the rat model.

SUPPLEMENTARY INFORMATION 3

Identification of intra-hepatic neural cells by snRNA-seq

In order to corroborate Western blot data with approaches combining high resolution and sensitivity, we tested by snRNA-seq the presence of immature and mature neuronal markers in the diseased liver. **Fig. S23** shows the average expression levels and percentage of cells expressing neuronal markers in each hepatic population.

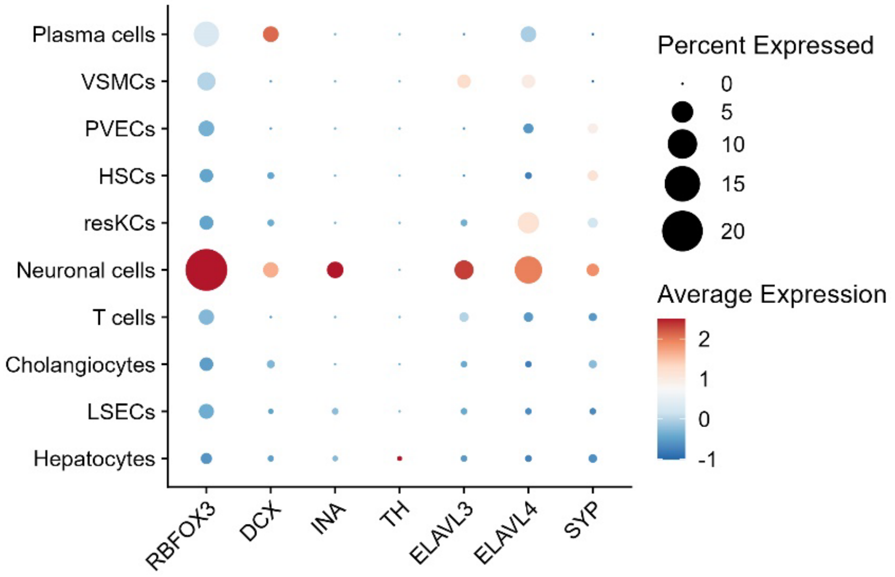

Fig. S23. Average expression levels and percentage of cells expressing neuronal markers in each hepatic population. Identified cell types following the analysis of liver snRNA-seq data from MASLD patients (n=2) and healthy individuals (n=2) (GSE174748) [28]. Interestingly, most markers of interest for general neuron identification were found, namely: *RBFOX3* (NeuN, mature neuron), *DCX* and *INA* (immature neuron), *TH* (sympathetic neuron, very low level as expected), *ELAVL3/4* (Huc proteins, immature neuron) and *SYP* (mature neuron/neuroendocrine cells). HSCs, hepatic stellate cells; MASLD, metabolic dysfunction-associated steatotic liver disease; LSECs, liver sinusoidal endothelial cells; PVECs, portal vein endothelial cells; resKCs, resident Kupffer cells; VSMCs, vascular smooth muscle cells.

Using these markers, the number of neuronal cells was estimated at around 5% in the Dimplot shown below (**Fig. S24**). This percentage is comparable to that of cholangiocytes, resident Kupffer cells and hepatic stellate cells. In that sense, the odds of irrelevant detection of neural cells in this setting are not greater than for these three traditionally-monitored liver resident cell

types. This population individualizes well in a uniform manifold approximation and projection (UMAP) plot (**Fig. S24**).

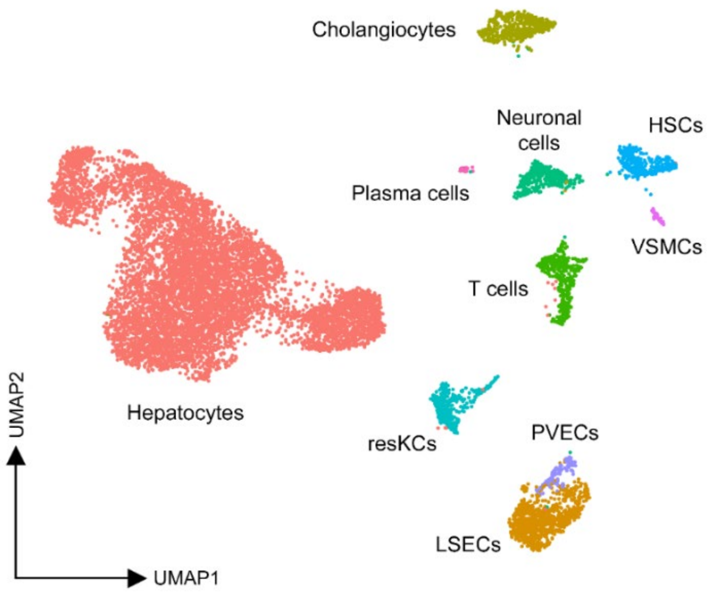

Fig. S24. UMAP plot of 10 relevant liver cell types. Populations identified following the analysis of liver snRNA-seq data from MASLD (n=2) patients and healthy individuals (n=2) (GSE174748) [28]. HSCs, hepatic stellate cells; MASLD, metabolic dysfunction-associated steatotic liver disease; LSECs, liver sinusoidal endothelial cells; PVECs, portal vein endothelial cells; resKCs, resident Kupffer cells; UMAP, uniform manifold approximation and projection; VSMCs, vascular smooth muscle cells.

These data compared neurons with several other liver-located cell types. None of the latter express such neural markers at appreciable levels, except in a minority of plasma cells for *DCX*. This work has been done in addition to immunohistochemistry (IHC) data, that shows coherent overlapping signals by several neural markers, *i.e.*, co-staining of the same structures by NeuN, DCX and VAChT antibodies, in the absence of TH signal, as was the case at the WB level in human and rat samples (**Fig. 1** and **Fig. S4**). The morphology of plasma cells being readily distinguishable from that of neurons, further supports the notion that the intrahepatic neural pool has genuinely been detected.

In order to further challenge these findings, we analyzed the potential selective enrichment of the previously identified neural candidate pool when searching for 'Reactome' genesets

associated with neural functions. Previously identified hepatic neural cells were enriched in several canonical cholinergic functions (**Fig. S25**).

Hence, we consider that the odds of falsely assigning non-neural cells as neural cells are limited at the level of this study.

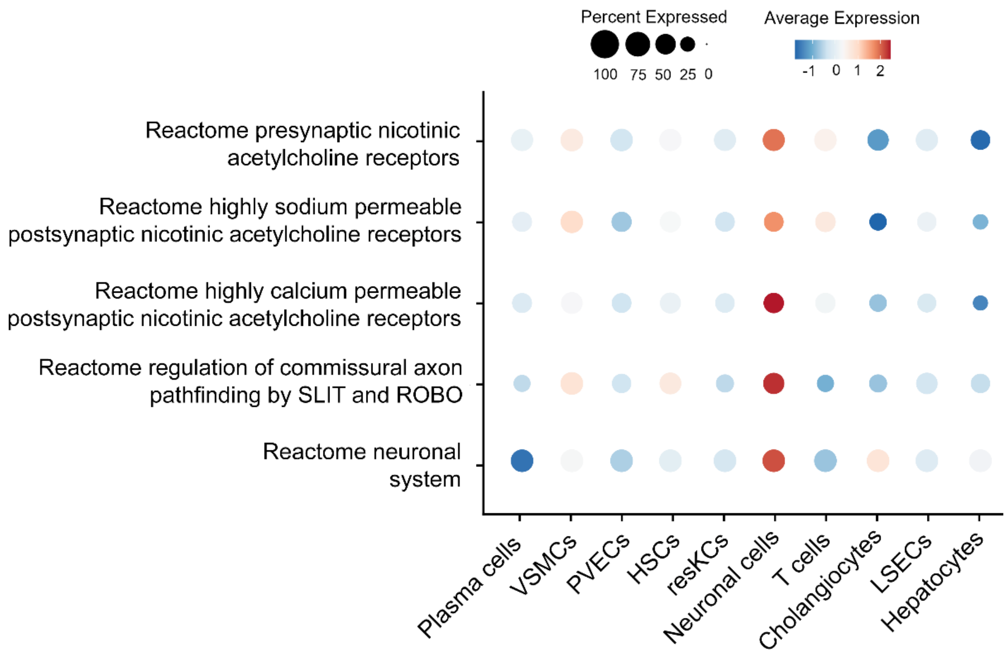

Fig. S25. Average expression levels and percentage of cells expressing signatures associated with neural function. Scores in each of the 10 relevant liver cell types identified following the analysis of liver snRNA-seq data from MASLD patients (n=2) and healthy individuals (n=2) (GSE174748). HSCs, hepatic stellate cells; MASLD, metabolic dysfunction-associated steatotic liver disease; LSECs, liver sinusoidal endothelial cells; PVECs, portal vein endothelial cells; resKCs, resident Kupffer cells; VSMCs, vascular smooth muscle cells.

SUPPLEMENTARY INFORMATION 4

Additional arguments for specific consideration of the CHRM3 receptor

RNA and protein data were considered. First, two independent datasets of paired HCC and non-tumor tissues were used for investigations at the RNA level (*i.e.*, GSE64041 and GSE124535).

In GSE64041, amongst all human cholinergic receptors (*CHRNA1* to *10* - *CHRNA8* being not expressed in humans, *CHRNB1* to *4*, *CHRNE-G*, and *CHRM1* to *5*), *CHRNA5* and *7* were the only ones to be significantly upregulated in paired comparisons with respect to non-tumoral tissues (see below), aside from *CHRM3* that was documented as HCC-induced in all tested datasets in the main body of the paper.

In GSE124535, *CHRNA1*, *2*, *5* and *7* were the only ones to be significantly up-regulated in paired comparisons with respect to non-tumoral tissues (see below), aside from *CHRM3* that was as said documented in the initial version of the paper.

Taking into consideration both cohorts, *CHRNA5* and *7* were repeatedly up-regulated across these datasets. Of note, differing from muscarinic receptors, nicotinic receptors are made of combinatory associations of several alpha and beta subunits, increasing their functional geometric diversity [29], and making their activity in the epithelia challenging to characterize, though they likely assemble in an organ-specific manner [29]. Specifically, we have not been able to identify *CHRNA5* and/or *7*-specific molecules with inverse agonist or antagonist activities in a controlled context. The identity of the beta chain partners of these alpha chains remains uncharted in the liver. Quantification data are below.

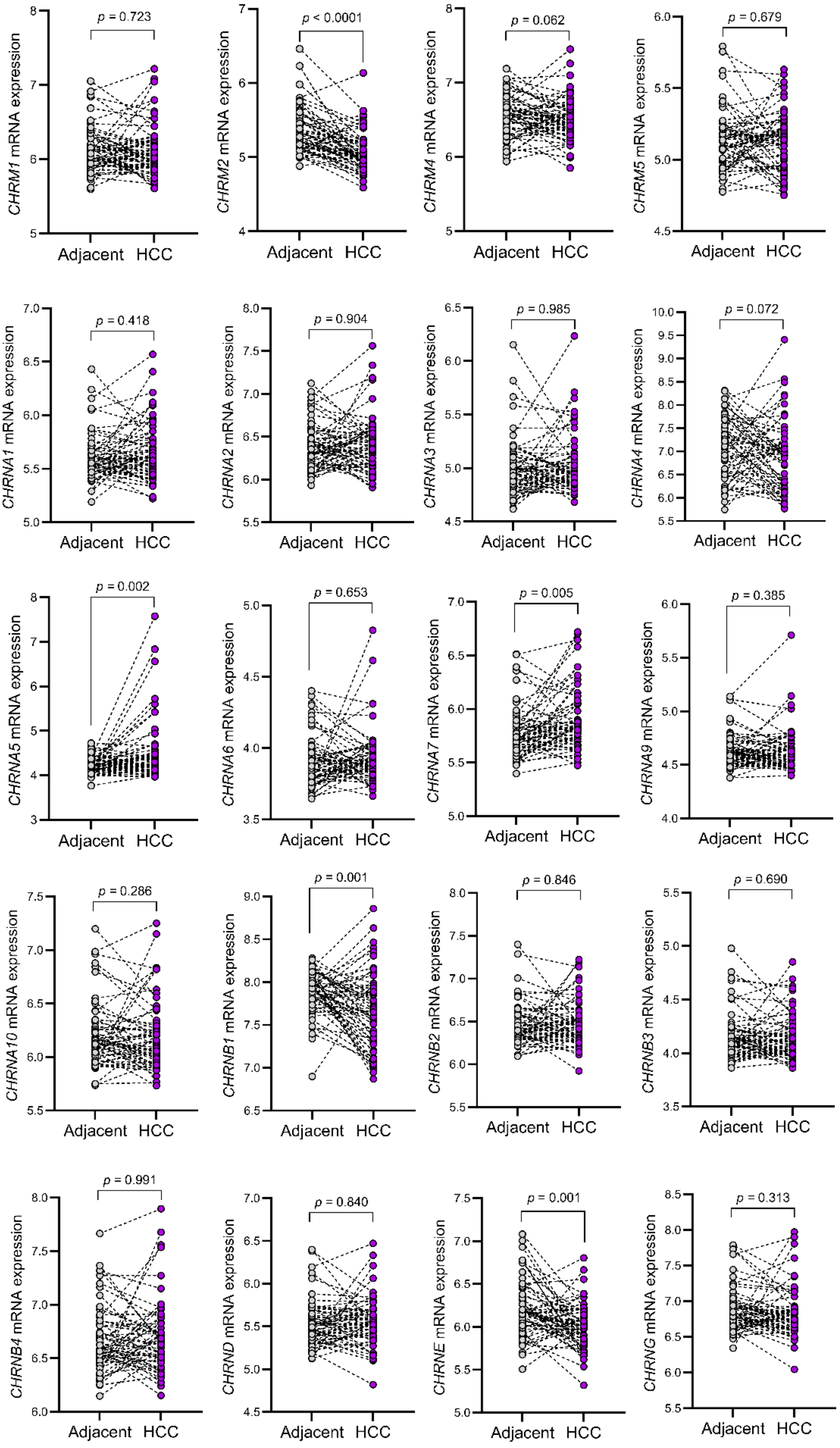

Fig. S26. Comparison of the expression of neuronal receptors between adjacent and HCC tumor tissues. Wilcoxon matched-pairs signed rank test, n=60 (GSE64041).

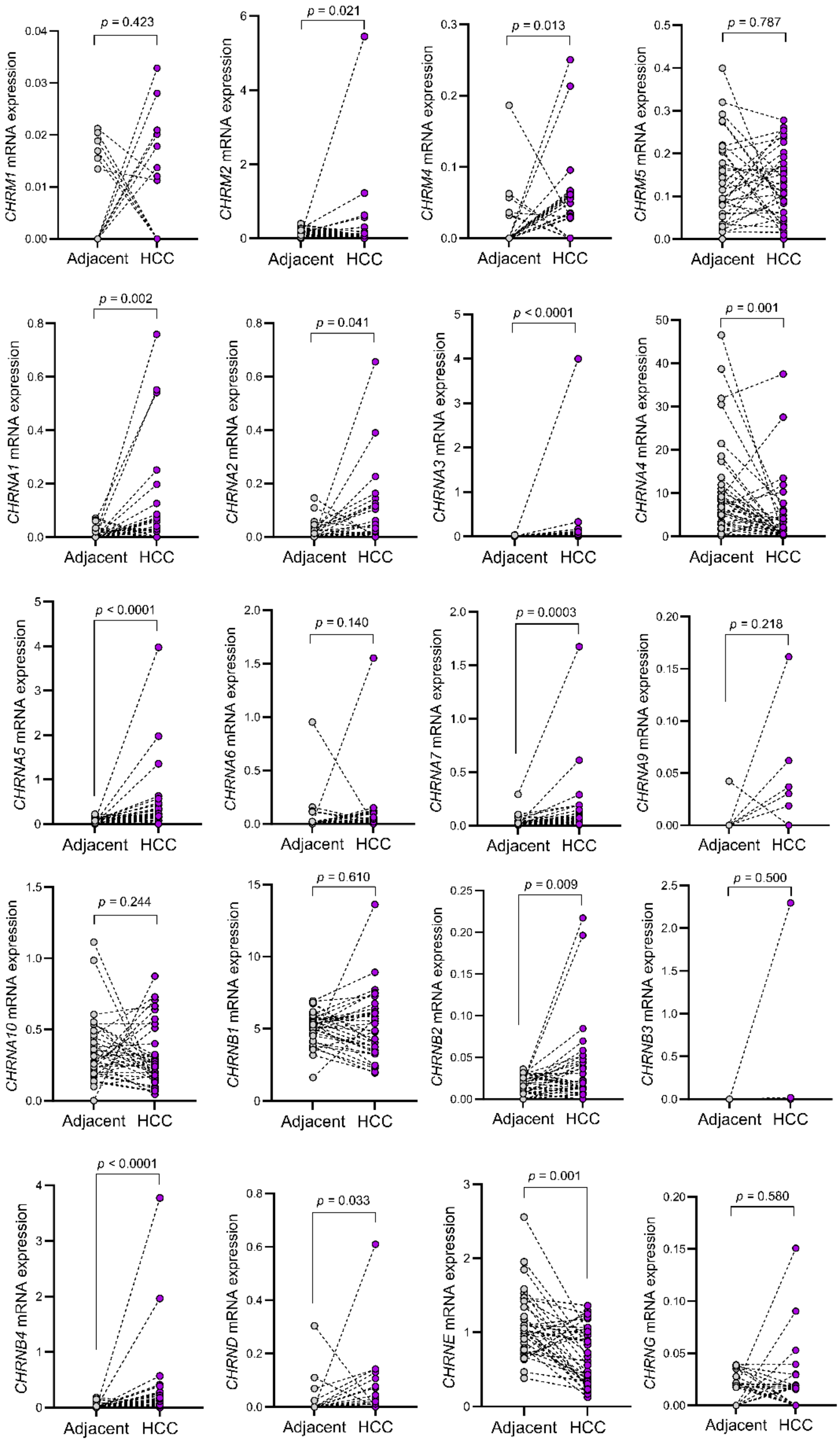

Fig. S27. Comparison of the expression of neuronal receptors between adjacent and HCC tumor tissues. Wilcoxon matched-pairs signed rank test, n=35 (GSE124535).

Regulation data are summarized in **Table S14** below.

Table S14. Regulation data of cholinergic receptors in the GSE64041 and GSE124535 datasets, as non-tumoral / tumoral pairs. Paired Wilcoxon test. Cut-off of 0.05. Ns, non-significant.

	p-values (tumoral over non tumoral comparison)		
	GSE64041	GSE124535	Commonly induced targets
CHRNA1	ns	0.002 (induction)	
CHRNA2	ns	0.04 (induction)	
CHRNA3	ns	<0.0001 (induction)	
CHRNA4	ns	0.001 (repression)	
CHRNA5	0.002 (induction)	<0.0001 (induction)	*CHRNA5*
CHRNA6	ns	ns	
CHRNA7	0.005 (induction)	0.0003 (induction)	*CHRNA7*
CHRNA8	Unexpressed in humans	Unexpressed in humans	
CHRNA9	ns	ns	
CHRNA10	ns	ns	
CHRNB1	0.001 (repression)	0.001 (repression)	
CHRNB2	ns	0.009 (induction)	
CHRNB3	ns	ns	
CHRNB4	ns	<0.0001 (induction)	
CHRND	ns	0.03 (induction)	
CHRNE	ns	0.001 (repression)	
CHRNG	ns	ns	
CHRM1	ns	0.021 (repression)	
CHRM2	<0.0001 (repression)	<0.0001 (repression)	
CHRM3			
CHRM4	ns	0.013 (induction)	
CHRM5	ns	ns	

We then explored these notions at the protein level, using the ProteinAtlas database that proposes HCC protein levels generated with antibodies benchmarked after technical validation by IHC. Protein expression data were available for *CHRNA1, 3, 4, 5* and *7* nicotinic receptors and for *CHRM1* to *5* muscarinic receptors. RNA data gathered on *CHRNA5* and *7* were likely not confirmed at the protein level (n=10 cases analyzed by IHC), while, again *CHRM3* expression was sharply induced at the protein level (n=11 cases). See **Fig. S28** below.

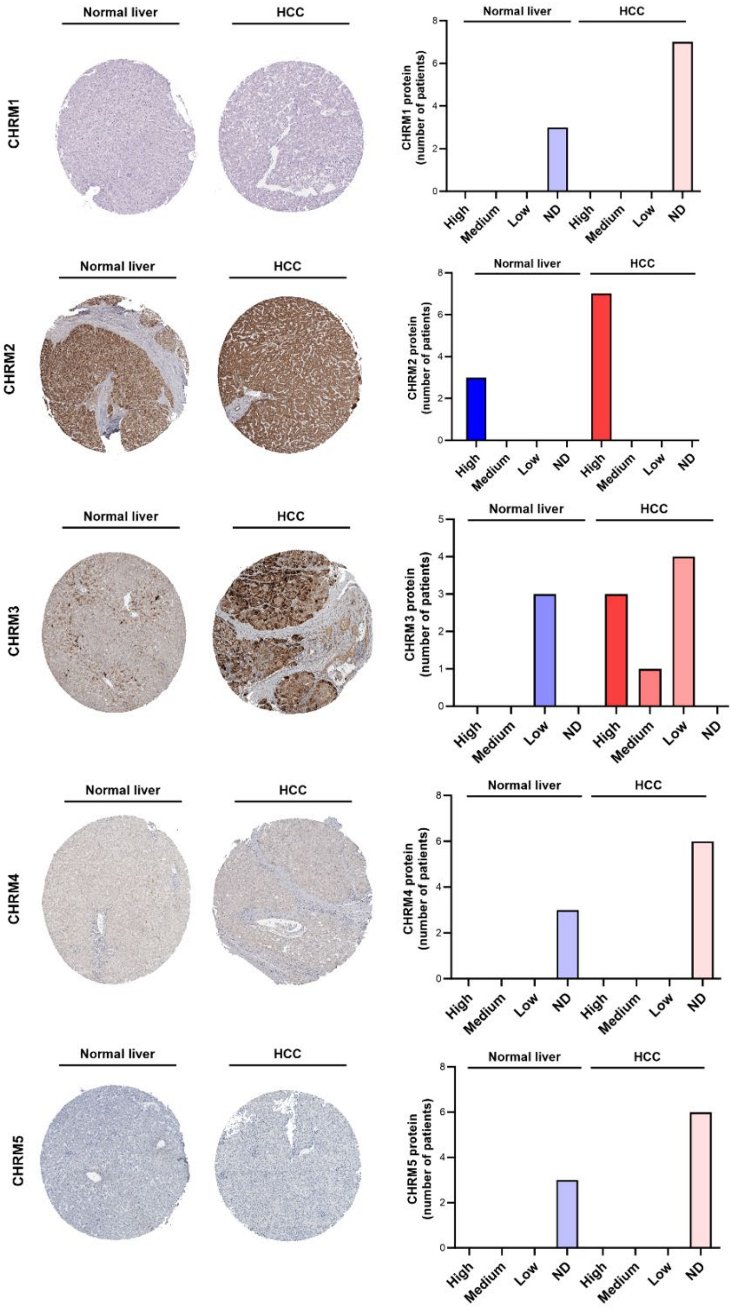

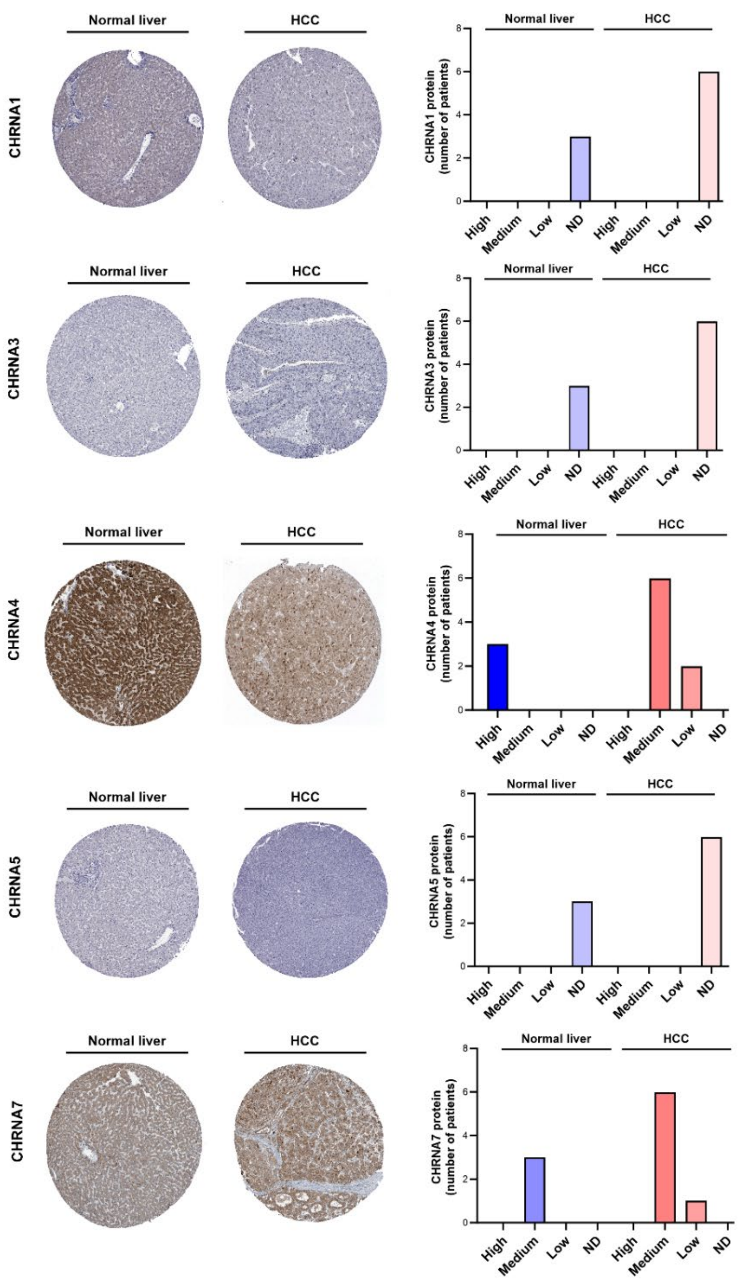

Fig. S28. Cholinergic receptor protein expression data retrieved from the ProteinAtlas database. n=10 to 11 cases.

We also explored ProteinAtlas data with respect to CHRM3 frequency of staining across cancer types. HCC ranks 2nd out of 20 cancer types in the ProteinAtlas database with respect to frequency of staining of this antigen using a validated antibody, see https://www.proteinatlas.org/ENSG00000133019-CHRM3/pathology in a context where hepatocytes themselves bear the signal (see 2D matrix of the following link): https://www.proteinatlas.org/ENSG00000133019-CHRM3/single+cell+type/liver. Such data

suggest that, in patients, HCC may more often rely on CHRM3 than other cancer types for its onset or development.

Interestingly, in the BIOSTORM cohort [15], only hepatocytic phosphorylated extracellular signal-regulated kinase (pERK) and microvascular invasion, as elsewhere [30], predicted poor recurrence-free survival after sorafenib. This is of interest with respect to the known ability of CHRM3 to activate ERK [31], and to be expressed by portal vein endothelial cells (PVECs) as a likely important cell type cytologically defining affected sites of microvascular invasion (see scRNA-seq data below).

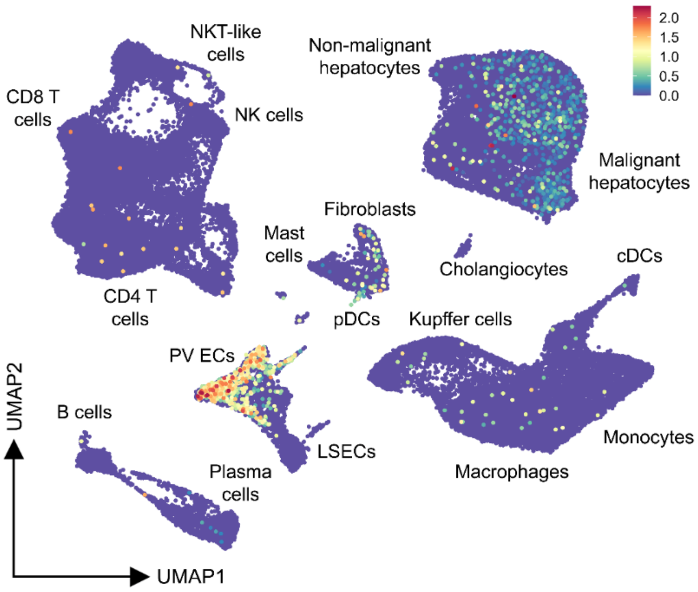

Fig. S29. *CHRM3* expression level in each cell type represented as UMAP in HCC samples. Data obtained from GSE149614 (n=10). cDCs, classical dendritic cells; CHRM3, cholinergic receptor muscarinic 3; NK, natural killer cells; LSECs, liver sinusoidal endothelial cells; PVECs, portal vein endothelial cells; pDCs, plasmacytoid dendritic cells; UMAP, uniform manifold approximation and projection.

Altogether, because of (i) the evidence for concordant increases from non-tumoral to tumoral tissues restricted to CHRM3 amongst all cholinergic receptors, both at the RNA and protein levels, (ii) the frequency of CHRM3-positive staining in HCC versus 20 other cancer types, (iii) the absence of robust structural features for selective targeting of any other induced receptor, and (iv) the association of CHRM3 with adverse markers of sensitivity to sorafenib and of poor survival (activation of Erk and microvascular invasion) in the literature, CHRM3 was selected as the most adequate cholinergic protein for subsequent HCC targeting in the study.

SUPPLEMENTARY INFORMATION 5

Engagement of the CHRM3 receptor impacts phosphorylation of EGFR, STAT3, MAPK and YAP pathways markers.

HCC lines or interest and PHH were treated with darifenacin or cevimeline as agonist or antagonist of the CHRM3 receptor. Canonical pathways of interest in HCC pathology and susceptible to be functionally linked to CHRM3 [31, 32] were then probed considering their main respective markers.

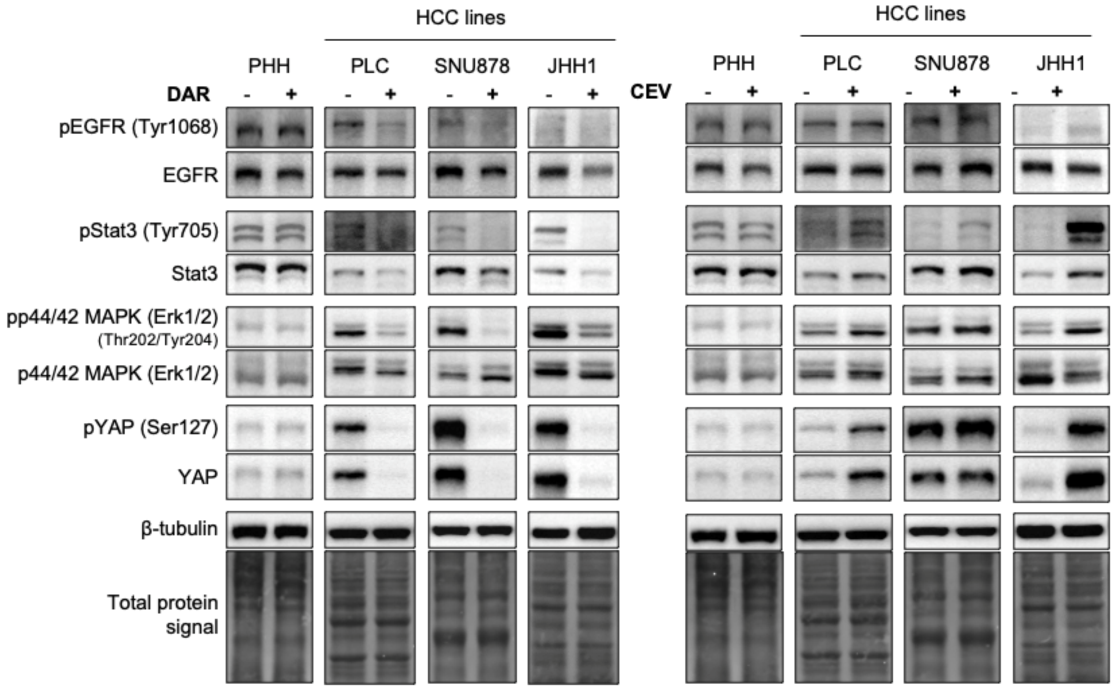

Fig. S30. CHRM3 engagement modulates EGFR, STAT3, MAPK and YAP in class 1, 2, and 3 human HCC lines following treatment with antagonist darifenacin (A) or agonist cevimeline (B). Incubation times vary from 4 to 48 h depending on cell line. PHH were used as non-cancer cell controls (n=3). EGFR, epidermal growth factor receptor; ERK, extracellular signal-regulated kinase; MAPK, mitogen-activated protein kinase; PHH, primary human hepatocytes; STAT3, signal transducer and activator of transcription 3; YAP, yes1 associated transcriptional regulator.

SUPPLEMENTARY INFORMATION 6

Applicability of the NRS to other hepatic malignancies

Using the same computational strategy as for all previous work in this study, we calculated the NRS values pertaining to cholangiocarcinoma (CCA, also named ICC, see below) in three publicly available bioinformatics datasets related to these conditions. Control tissues were paired adjacent tissues or normal livers. **Fig. S31** data below indicate that, as in HCC, lower NRS values characterize tumor tissues in these three malignancies.

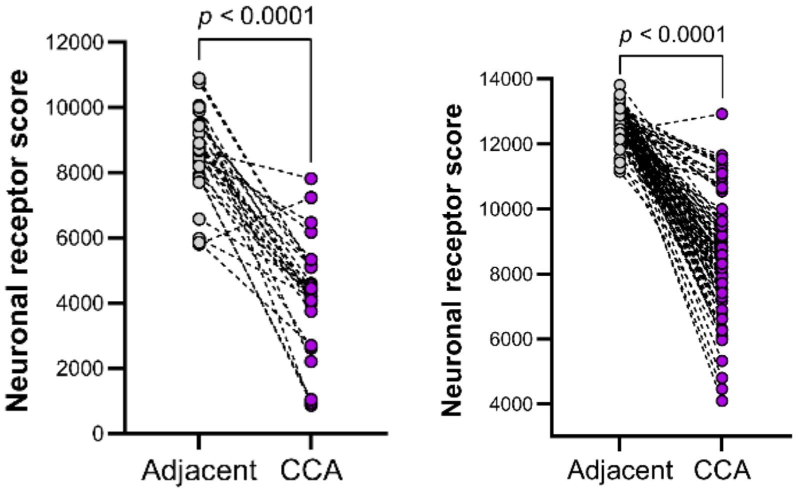

Fig. S31. Comparison of neuronal receptor scores between adjacent and CCA tumor tissues. From left to right: GSE107943, n=27 [33]; GSE76297, n=90 [34] ; Wilcoxon matched-pairs signed rank test. NRS values were calculated as depicted in the *Materials and Methods* section. CCA, cholangiocarcinoma.

Of note, such cholinergic orientation was confirmed in another comparative study that considered normal livers as controls (**Fig. S32**).

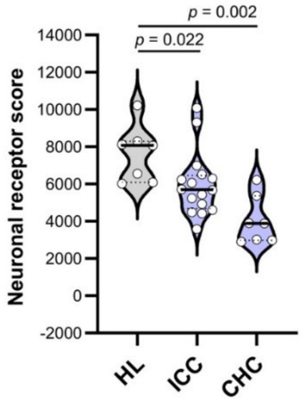

Fig. S32. Comparison of neuronal receptor scores across types of liver cancer (GSE32879). Samples include HL (n=7), ICC (n=16) and CHC (n=7). Mann-Whitney test versus HL. CHC, combined hepatocellular cholangiocarcinoma; HL, healthy liver; ICC, intrahepatic cholangiocarcinoma.

We then wondered whether *CHRM3* was also regulated in all these etiologies. **Fig. S33** below indicates that this potential target was up-regulated across all these types of cancers.

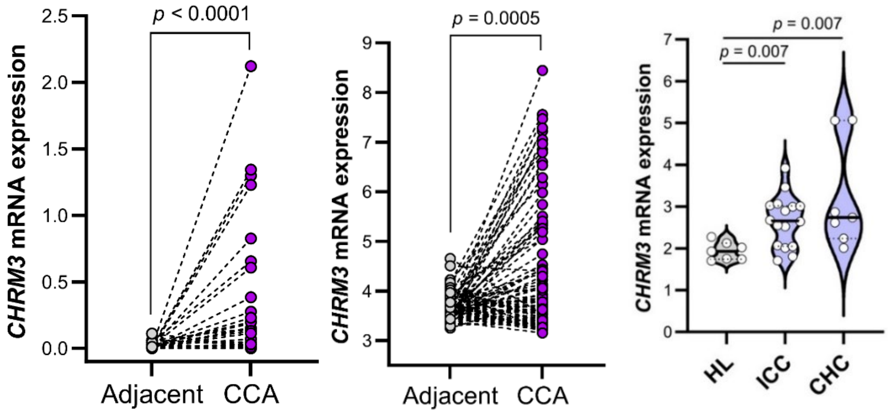

Fig. S33. Comparison of *CHRM3* mRNA expression between adjacent and CCA tumor tissues. Three panels above: Wilcoxon matched-pairs signed rank test, n=27 (GSE107943), between adjacent and CCA tumor tissues. Wilcoxon matched-pairs signed rank test, n=90 (GSE76297), between adjacent and CCA tumor tissues. Wilcoxon matched-pairs signed rank test, n=18 (GSE132037), and across liver disease etiologies (GSE32879). To the right, this last group of samples include HL (n=7), ICC (n=16) and CHC (n=7). CCA, cholangiocarcinoma; CHC, combined hepatocellular cholangiocarcinoma; CHRM3, cholinergic receptor muscarinic 3; HL, healthy liver; ICC, intrahepatic cholangiocarcinoma.

Altogether, these data indicate that a cholinergic oriented phenotype characterizes cancer lesions of the liver, with CHRM3 being a potential target for further investigations in CCA as well.

SUPPLEMENTARY INFORMATION 7

Transduction considerations related to targeted sub-groups of muscarinic receptors

Scopolamine is a pan-muscarinic antagonist whereas darifenacin is a M3 selective antagonist. The family of muscarinic acetylcholine receptors encompasses five members in mammals, encoded by the *CHRM1*-5 genes. These are G-protein-coupled receptors, which can be divided into the following two subfamilies: CHRM1, CHRM3, and CHRM5 receptors coupling to $G_{q/11}$; and CHRM2 and CHRM4 receptors coupling to $G_{i/o}$. Activation of CHRM1, CHRM3 and CHRM5 results in phospholipid turnover and changes in cell calcium concentration. Activation of CHRM2 and CHRM4 results in inhibition of adenyl cyclase and reduced levels of cAMP [35, 36]. Of these five muscarinic receptor subtypes, those that activate phospholipid turnover (CHRM1, 3, 5) are conditional oncogenes when expressed in cells capable of proliferation [36].

Anchorage-independent growth assays described herein select colonies according to their ability to withstand stress related to the likely absence of integrin signaling, while this study's pharmacological assays challenge cells on their ability to shift towards dependence to CHRM3 signaling for survival upon inhibition of cellular kinases by sorafenib. The fact that scopolamine becomes active in the latter setting suggests that both phosphoinositides and cAMP participate to survival in these conditions, while phosphoinositides per se are instrumental for survival upon deprivation of integrin signaling, as reviewed [37]. Alternatively, at least in the context of anchorage-independent growth, this could also mean that pan-muscarinic inhibition by scopolamine may hinder the benefits of CHRM1-3-5 inhibition, expected to limit survival, through concomitant engagement of the likely anticancer M2-4 receptors. Given the complexity of processes with respect to this question, this issue will be further explored in a subsequent study.

SUPPLEMENTARY INFORMATION 8

Quantification of neuronal receptors and NRS levels in immune population present in the HCC microenvironment

In order to gain further insights related to the potential of intrahepatic immune populations as targets for neural signals, we analyzed publicly available scRNA-seq data derived from HCC tissues (n=38) [38]. In this dataset constituted of lymphoid and myeloid cell types (**Fig. S34A, B**), quantification of individual neuronal receptors allowed us to observe that *ADRB2*, *CHRNB1* and *CHRNE* present the highest expression (**Fig. S34C**). Moreover, quantification of the NRS showed that CD8 TEMRA and cytotoxic NK cells are the sub-populations with the strongest adrenergic polarization (**Fig. S34D**). These observations are in line with previous reports describing how cholinergic signaling dampens CD8 T cell activity [39], as illustrated by the markedly lower NRS levels in exhausted CD8 populations. Similar results were obtained by re-clustering the lymphoid compartment from the previously described GSE149614 dataset (**Fig. S35**). Nonetheless, it is worth noting that in the particular case of *CHRM3*, malignant hepatocytes present higher expression levels as compared to immune populations (**Fig. S29**).

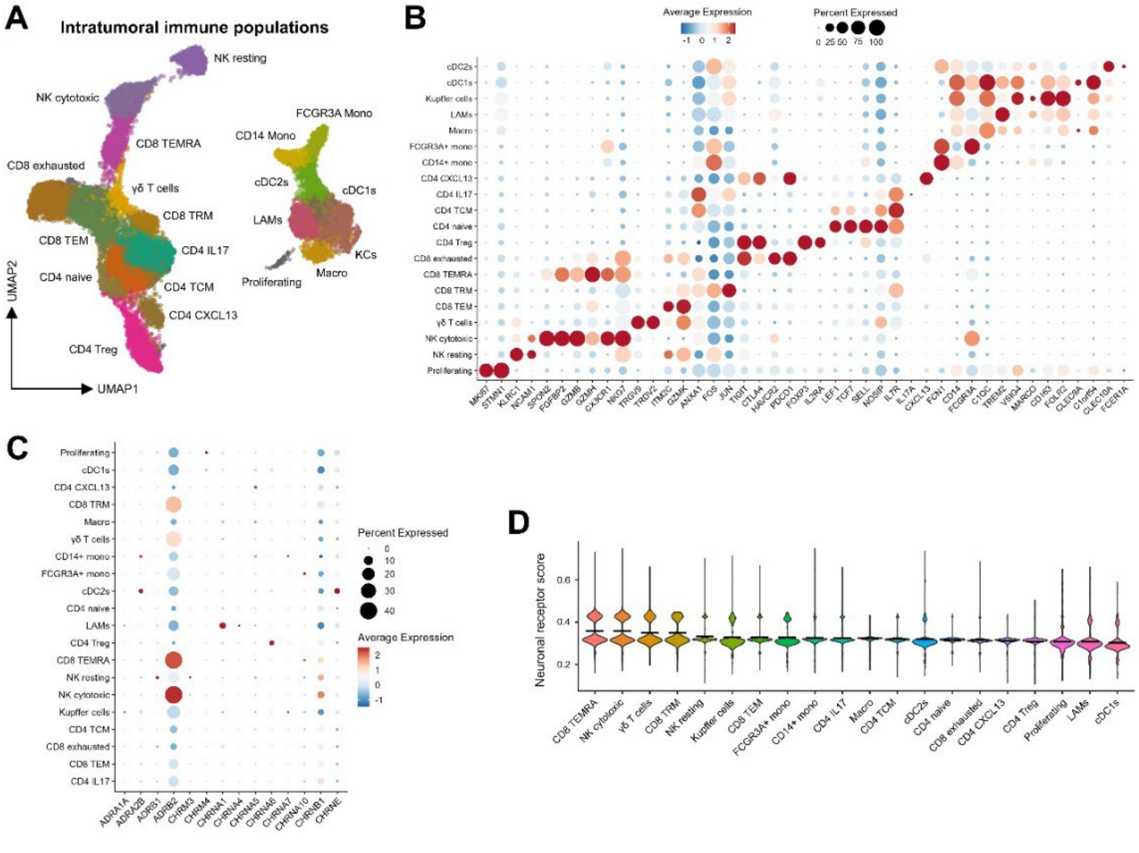

Fig. S34. Expression of individual neuronal receptors and the NRS in each immune cell type found in HCC samples. (**A**) UMAP plot of 20 cell populations identified following analysis of scRNA-seq data from 38 HCC patients [38]. (**B**) Dotplot showing the average expression levels and percentage of cells expressing canonical cell type markers in each of the 20 immune cell types. (**C**) Dotplot showing the average expression levels and percentage of cells expressing individual neuronal receptors in each of the identified cell types. (**D**) GSVA scores for the neuronal receptor signature in each cell type, represented as UMAP (top) and violin plot (bottom). cDCs, classical dendritic cells; KCs, Kupffer cells; NK, natural killer; NRS, neuronal receptor score; LAMs, lipid-associated macrophages; TCM, T central memory; TEM, T effector memory; TEMRA, T effector memory RA; Treg, regulatory T cells; TRM, tissue resident memory; UMAP, uniform manifold approximation and projection.

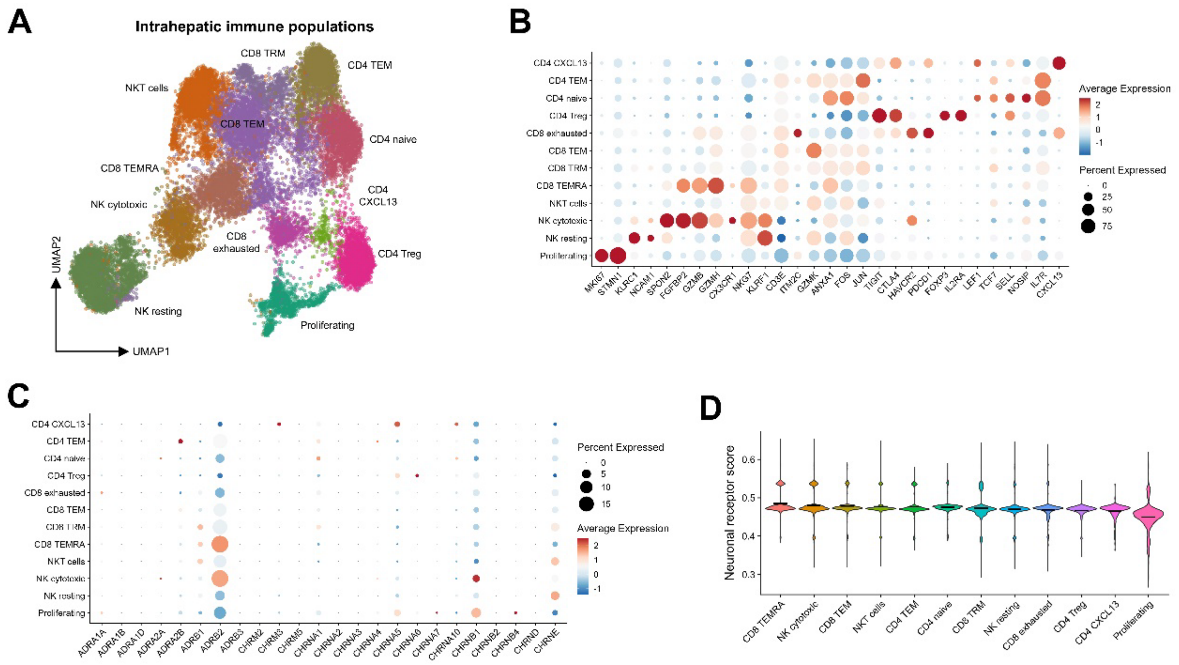

Fig. S35. Expression of individual neuronal receptors and the NRS in each immune cell type found in HCC samples. (**A**) UMAP plot showing 12 lymphoid sub-populations identified following re-analysis of scRNA-seq data from 10 patients (GSE149614). (**B**) Dotplot showing the average expression levels and percentage of cells expressing canonical cell type markers in each of the 20 immune cell types. (**C**) Dotplot showing the average expression levels and percentage of cells expressing individual neuronal receptors in each of the identified cell types. (**D**) GSVA scores for the neuronal receptor signature in each cell type, represented as UMAP (top) and violin plot (bottom). cDCs, classical dendritic cells; KCs, Kupffer cells; NK, natural

killer; NRS, neuronal receptor score; LAMs, lipid-associated macrophages; TCM, T central memory; TEM, T effector memory; TEMRA, T effector memory RA; Treg, regulatory T cells; TRM, tissue resident memory; UMAP, uniform manifold approximation and projection.

SUPPLEMENTARY REFERENCES

[1] Gripon P, Rumin S, Urban S, et al. Infection of a human hepatoma cell line by hepatitis B virus. Proceedings of the National Academy of Sciences of the United States of America 2002;99:15655-15660

[2] Zheng S, Wang W, Aldahdooh J, et al. SynergyFinder Plus: Toward Better Interpretation and Annotation of Drug Combination Screening Datasets. Genomics Proteomics Bioinformatics 2022;20:587-596

[3] Horiuchi S, Kuroda Y, Oyafuso R, et al. Construction of a culture protocol for functional bile canaliculi formation to apply human iPS cell-derived hepatocytes for cholestasis evaluation. Sci Rep 2022;12:15192

[4] Satija R, Farrell JA, Gennert D, et al. Spatial reconstruction of single-cell gene expression data. Nat Biotechnol 2015;33:495-502

[5] Hanzelmann S, Castelo R, Guinney J. GSVA: gene set variation analysis for microarray and RNA-seq data. BMC Bioinformatics 2013;14:7

[6] Miranda A, Hamilton PT, Zhang AW, et al. Cancer stemness, intratumoral heterogeneity, and immune response across cancers. Proc Natl Acad Sci U S A 2019;116:9020-9029

[7] Guilliams M, Bonnardel J, Haest B, Vanderborght B, et al. Spatial proteogenomics reveals distinct and evolutionarily conserved hepatic macrophage niches. Cell 2022;185:379-396 e338

[8] Ragnum HB, Vlatkovic L, Lie AK, et al. The tumour hypoxia marker pimonidazole reflects a transcriptional programme associated with aggressive prostate cancer. Br J Cancer 2015;112:382-390

[9] Buffa FM, Harris AL, West CM, et al. Large meta-analysis of multiple cancers reveals a common, compact and highly prognostic hypoxia metagene. Br J Cancer 2010;102:428-435

[10] Winter SC, Buffa FM, Silva P, et al. Relation of a hypoxia metagene derived from head and neck cancer to prognosis of multiple cancers. Cancer Res 2007;67:3441-3449

[11] Liu J, Lichtenberg T, Hoadley KA, et al. An Integrated TCGA Pan-Cancer Clinical Data Resource to Drive High-Quality Survival Outcome Analytics. Cell 2018;173:400-416 e411

[12] Therneau T. A Package for Survival Analysis in R. R package 2021 [cited version 3.2-13; Available from: https://CRAN.R-project.org/package=survival

[13] Therneau TM GP. Modeling Survival Data: Extending the Cox Model. Springer, New-York 2000

[14] Kassambara AK, M.; Biecek, P. Survminer: Drawing Survival Curves using 'ggplot2'. R package Version 0.4.9

[15] Pinyol R, Montal R, Bassaganyas L, et al. Molecular predictors of prevention of recurrence in HCC with sorafenib as adjuvant treatment and prognostic factors in the phase 3 STORM trial. Gut 2019;68:1065-1075

[16] Roth GS, Macek Jilkova Z, Zeybek Kuyucu A, et al. Efficacy of AKT Inhibitor ARQ 092 Compared with Sorafenib in a Cirrhotic Rat Model with Hepatocellular Carcinoma. Mol Cancer Ther 2017;16:2157-2165

[17] Kurma K, Manches O, Chuffart F, et al. DEN-Induced Rat Model Reproduces Key Features of Human Hepatocellular Carcinoma. Cancers (Basel) 2021;13

[18] Thaker PH, Han LY, Kamat AA, et al. Chronic stress promotes tumor growth and angiogenesis in a mouse model of ovarian carcinoma. Nat Med 2006;12:939-944

[19] Magnon C, Hall SJ, Lin J, et al. Autonomic nerve development contributes to prostate cancer progression. Science 2013;341:1236361

[20] Hayakawa Y, Sakitani K, Konishi M, et al. Nerve Growth Factor Promotes Gastric Tumorigenesis through Aberrant Cholinergic Signaling. Cancer Cell 2017;31:21-34

[21] Renz BW, Takahashi R, Tanaka T, et al. beta2 Adrenergic-Neurotrophin Feedforward Loop Promotes Pancreatic Cancer. Cancer Cell 2018;33:75-90 e77

[22] Renz BW, Tanaka T, Sunagawa M, et al. Cholinergic Signaling via Muscarinic Receptors Directly and Indirectly Suppresses Pancreatic Tumorigenesis and Cancer Stemness. Cancer Discov 2018;8:1458-1473

[23] Jensen KJ, Alpini G, Glaser S. Hepatic nervous system and neurobiology of the liver. Compr Physiol 2013;3:655-665

[24] Adori C, Daraio T, Kuiper R, et al. Disorganization and degeneration of liver sympathetic innervations in nonalcoholic fatty liver disease revealed by 3D imaging. Sci Adv 2021;7

[25] Barnault R, Verzeroli C, Fournier C, et al. Hepatic inflammation elicits production of proinflammatory netrin-1 through exclusive activation of translation. Hepatology 2022;76:1345-1359

[26] Lahlali T, Plissonnier ML, Romero-Lopez C, et al. Netrin-1 Protects Hepatocytes Against Cell Death Through Sustained Translation During the Unfolded Protein Response. Cell Mol Gastroenterol Hepatol 2016;2:281-301 e289

[27] Plissonnier ML, Lahlali T, Michelet M, et al. Epidermal Growth Factor Receptor-Dependent Mutual Amplification between Netrin-1 and the Hepatitis C Virus. PLoS Biol 2016;14:e1002421

[28] Filliol A, Saito Y, Nair A, et al. Opposing roles of hepatic stellate cell subpopulations in hepatocarcinogenesis. Nature 2022;610:356-365

[29] Albuquerque EX, Pereira EF, Alkondon M, et al. Mammalian nicotinic acetylcholine receptors: from structure to function. Physiol Rev 2009;89:73-120

[30] Erstad DJ, Tanabe KK. Prognostic and Therapeutic Implications of Microvascular Invasion in Hepatocellular Carcinoma. Ann Surg Oncol 2019;26:1474-1493

[31] Felton J, Hu S, Raufman JP. Targeting M3 Muscarinic Receptors for Colon Cancer Therapy. Curr Mol Pharmacol 2018;11:184-190

[32] Guo L, Liu Y, Ding Z, et al. Signal transduction by M3 muscarinic acetylcholine receptor in prostate cancer. Oncol Lett 2016;11:385-392

[33] Ahn KS, O'Brien D, Kang YN, et al. Prognostic subclass of intrahepatic cholangiocarcinoma by integrative molecular-clinical analysis and potential targeted approach. Hepatol Int 2019;13:490-500

[34] Chaisaingmongkol J, Budhu A, Dang H, et al. Common Molecular Subtypes Among Asian Hepatocellular Carcinoma and Cholangiocarcinoma. Cancer Cell 2017;32:57-70 e53

[35] Von Rosenvinge EC, Raufman JP. Muscarinic receptor signaling in colon cancer. Cancers (Basel) 2011;3:971-981

[36] Gutkind JS, Novotny EA, Brann MR, et al. Muscarinic acetylcholine receptor subtypes as agonist-dependent oncogenes. Proc Natl Acad Sci U S A 1991;88:4703-4707

[37] DeMali KA, Wennerberg K, Burridge K. Integrin signaling to the actin cytoskeleton. Curr Opin Cell Biol 2003;15:572-582

[38] Cappuyns S, Philips G, Vandecaveye V, et al. PD-1- CD45RA+ effector-memory CD8 T cells and CXCL10+ macrophages are associated with response to atezolizumab plus bevacizumab in advanced hepatocellular carcinoma. Nat Commun 2023;14(1):7825

[39] Bauer KC, Trehan R, Ruf B, et al. The Gut Microbiome Controls Liver Tumors via the Vagus Nerve. bioRxiv [Preprint] 2024;2024.01.23.576951
