# Supplementary material for: Hepatocellular carcinoma hosts cholinergic neural cells and tumoral hepatocytes harboring targetable muscarinic receptors
Source: JHEP Rep. 2024 Nov 12;7(1):101245. doi: 10.1016/j.jhepr.2024.101245 (PMC11663970; doi:10.1016/j.jhepr.2024.101245)
Supplement: Multimedia component 2 [file mmc2.docx]

**Journal of Hepatology**

**CTAT methods**

Tables for a “Complete, Transparent, Accurate and Timely account” (CTAT) are now mandatory for all revised submissions. The aim is to enhance the reproducibility of methods.

- Only include the parts relevant to your study
- Refer to the CTAT in the main text as ‘Supplementary CTAT Table’
- Do not add subheadings
- Add as many rows as needed to include all information
- Only include one item per row

**If the CTAT form is not relevant to your study, please outline the reasons why:**

|  |
| --- |

- 1. **Antibodies**

| **Name** | **Citation** | **Supplier** | **Cat no.** | **Clone no.** | **RRID** |
| --- | --- | --- | --- | --- | --- |
| Beta-tubulin |  | Abcam | Ab6046 | Polyclonal | AB_2210370 |
| NeuN |  | Millipore | MAB377 | A60 | AB_2298772 |
| DCX (WB) |  | Abcam | Ab18723 | Polyclonal | AB_732011 |
| DCX (IHC) |  | Millipore | MABN707 | 2G5 | ND |
| Alpha-internexin |  | Millipore | MAB5224 | 2E3 | AB_2127486 |
| TH (WB) |  | Millipore | AB152 | Polyclonal | AB_390204 |
| TH (IHC) |  | Abcam | Ab112 | Polyclonal | AB_297840 |
| VAChT (WB) |  | Sigma | SAB5200240 | S6-38 | ND |
| VAChT (IHC) |  | SYSY | PA5-85782 | Polyclonal | AB_2992918 |
| Netrin-1 |  | Abcam | Ab126729 | EPR5428 | AB_11131145 |
| Phospho-EGF  Receptor (Tyr1068) |  | Cell Signaling | 3777 | D7A5 | AB_2096270 |
| EGF Receptor |  | Cell Signaling | 4627 | D38B1 | AB_2895042 |
| Phospho-Stat3  (Tyr705) |  | Cell Signaling | 9145 | D3A7 | AB_2491009 |
| Stat3 |  | Cell Signaling | 9139 | 124H6 | AB_331757 |
| Phospho-p44/42  MAPK (Erk1/2)  (Thr202/Tyr204) |  | Cell Signaling | 4370 | D13.14.4E | AB_2315112 |
| p44/42 MAPK  (Erk1/2) |  | Cell Signaling | 4695 | 137F5 | AB_390779 |
| Phospho-YAP  (Ser127) |  | Cell Signaling | 4911 | Polyclonal | AB_2218913 |
| YAP |  | Cell Signaling | 4912 | Polyclonal | AB_2218911 |
| Anti-mouse-HRP |  | Sigma | A4416 | Polyclonal | AB_258167 |
| Anti-Rabbit-HRP |  | Sigma | A6154 | Polyclonal | AB_258284 |
| Synaptophysin |  | ThermoScientific (IHC) | RM-9111-S | SP11 | AB_149938 |
| CD45 (IHC) |  | Invitrogen | 14-9457-82 | CD45-2B11 | AB_11063696 |
| CD31 (IHC) |  | Dako | M0823 | JC70A | AB_2114471 |
| Goat anti-Rabbit-HRP |  | Roche | 7259883001 | Polyclonal | ND |
| Goat anti-Mouse HRP |  | Roche | 7988192001 | Polyclonal | ND |
| Goat anti-Rabbit HRP |  | Roche | 7988150001 | Polyclonal | ND |
| Goat anti-Mouse HRP |  | Akoya | FP1501001KT | Polyclonal | ND |
| DAPI |  | Sigma | D9564-10MG |  | ND |

- 1. **Cell lines**

| **Name** | **Citation** | **Supplier** | **Cat no.** | **Passage no.** | **Authentication test method** |
| --- | --- | --- | --- | --- | --- |
| PLC | Caruso S et al. Gastroenterology. 2019 | J. Zucman-Rossi’s  laboratory | CVCL_0485 | <20 | STR |
| SNU878 | Caruso S et al. Gastroenterology. 2019 | J. Zucman-Rossi’s  laboratory | CVCL_5102 | <20 | STR |
| JHH4 | Caruso S et al. Gastroenterology. 2019 | J. Zucman-Rossi’s  laboratory | CVLC_2787 | <20 | STR |
| JHH1 | Caruso S et al. Gastroenterology. 2019 | J. Zucman-Rossi’s  laboratory | CVCL_2785 | <20 | STR |
| HepaRG | Gripon P et al. PNAS. 2001 | In house | CVCL_9720 | <20 | STR |

- 1. **Organisms**

| **Name** | **Citation** | **Supplier** | **Strain** | **Sex** | **Age** | **Overall n number** |
| --- | --- | --- | --- | --- | --- | --- |
| Rats Fischer 334 | Roth GS et al. Mol Cancer Ther. 2017  Kurma K et al. Cancers (Basel). 2021 | Janvier Labs | N/A | Male | 6-weeks | 5 |
|  |  |  |  | Male | 8-weeks | 8 |
|  |  |  |  | Male | 14-weeks | 8 |
|  |  |  |  | Male | 20-weeks | 7 |

- 1. **Sequence based reagents**

| **Name** | **Genbank**  **acc. #** | **Sequence (5’-3’, F/R)** | **PCR conditions** | **Amplicon**  **length, bp** | **Supplier** |
| --- | --- | --- | --- | --- | --- |
| *GUS* | NM_001293105 | CGTGGTTGGAGAGCTCATTTGGAA  TTCCCCAGCACTCTCGTCGGT | Denaturing, 95°C;  Annealing, 60°C | 72 | Eurogentec |
| *ADRA1A* | NM_000680.4 | CCAAGACGGATGGCGTTTG  TGGACACTGTAATCCTGGCAG |  | 75 |  |
| *ADRA1B* | NM_000679.4 | TGGGGCGGATCTTCTGTGA  GTGACCAGCGTGGGATACTG |  | 136 |  |
| *ADRA1D* | NM_000678.4 | GCCGCTCGGCTCCTTG  GGCTGGAACAGGGGTAGATG |  | 116 |  |
| *ADRA2A* | NM_000681.4 | TCGTCATCATCGCCGTGTTC  AAGCCTTGCCGAAGTACCAG |  | 156 |  |
| *ADRA2B* | NM_000682.7 | AGAGGTCAACGGACACTCGAA  CCCCACAAACACCCTCCTT |  | 140 |  |
| *ADRA2C* | NM_000683.4 | TGGTCATGCCCTTCTCGTTG  AGGTGCAAAACAGCACATCG |  | 102 |  |
| *ADRB1* | NM_000684.3 | ATCGAGACCCTGTGTGTCATT  GTAGAAGGAGACTACGGACGAG |  | 267 |  |
| *ADRB2* | NM_000024.6 | TGGTGTGGATTGTGTCAGGC  GGCTTGGTTCGTGAAGAAGTC |  | 128 |  |
| *ADRB3* | NM_000025.3 | GACCAACGTGTTCGTGACTTC  GCACAGGGTTTCGATGCTG |  | 175 |  |
| *CHRM1* | NM_000738.3 | GGACCCTACAGACCCCTCTTC  TGGGGCTGAAGTGTTCATGG |  | 126 |  |
| *CHRM2* | NM_001006630.2 | AACTCCTCTAACAATAGCCTGGC  GTTCCCGATAATGGTCACCAAA |  | 108 |  |
| *CHRM3* | NM_000740 | TACCTGGAACAGGTGGAGC  GATCCCGGCATAGGACAGAG |  | 73 |  |
| *CHRM4* | NM_000741.5 | CGAGGGGAGCTGGGC  CCACCGTCTCATAGCGATTG |  | 146 |  |
| *CHRM5* | NM_012125.4 | AGTCTGGCTTGTGACCTTTGG  TGTCAAGGGTCTTGTGATGGA |  | 114 |  |
| *CHRNA1* | NM_000079.4 | GGTCCACACAAGCTCCGGTA  ACCACGCTGCTGTAGTCTTT |  | 140 |  |
| *CHRNA2* | NM_000742.4 | CTGTCCATCGCTCAGCTCAT  GGTCACTGCAAACTCCCCAT |  | 207 |  |
| *CHRNA3* | NM_000743.5 | ACCTGTGGCTCAAGCAAATCT  GCAGGGACACGCATGAACT |  | 88 |  |
| *CHRNA4* | NM_000744.7 | CTCCGAGCTCATCTGGCG  TCCCCGTCAGCATTGTTGTA |  | 72 |  |
| *CHRNA5* | NM_000745.4 | AAAGATGGGTTCGTCCTGTGG  AACTGATTTTTCTCATCCACATCCA |  | 100 |  |
| *CHRNA6* | NM_004198.3 | ACACACACGGATTGCAGTGG  GAGCCTCTCCTCAGTTGCAC |  | 232 |  |
| *CHRNA7* | NM_000746.6 | GCTGGTCAAGAACTACAATCCC  CTCATCCACGTCCATGATCTG |  | 106 |  |
| *CHRNA9* | NM_017581.4 | CAGAAAATGTGCCCCTGATAGGTAA  TCGGCCCCACAGAAGTGGATA |  | 109 |  |
| *CHRNA10* | NM_020402.4 | CAGATGCCTACCTACGATGGG  GGGAAGGCTGCTACATCCA |  | 226 |  |
| *CHRNB1* | NM_000747.3 | GCAACTCATCAGCCTGAACG  GAATCGATGCCGTCGTGCTC |  | 117 |  |
| *CHRNB2* | NM_000748.3 | GACCACCAATGTCTGGCTGA  AAGGACACCTCGTACATGCC |  | 162 |  |
| *CHRNB3* | NM_000749.5 | CATCGTCCTTGGCATCCCTT  AGCAGCTCTGCTCTTTCACG |  | 98 |  |
| *CHRNB4* | NM_000750.5 | CGCCTTCCCTGGTCCTTTTC  TCAGCCAGACATTGGTGGTC |  | 85 |  |
| *CHRNE* | NM_000080.4 | CAATGCCGAAGAGGTGGAGT  CCCACTCGCCGTTCTCAGTA |  | 104 |  |
| *CHRND* | NM_000751.3 | CGTGGCACACAAAGAGGAGA  CAATCTCTGGGAGCCACACC |  | 164 |  |
| *CHRNG* | NM_005199.5 | AACGAGACTCGGATGTGGTC  ACTGAGATAGAGCAGGCGGA |  | 228 |  |
| *ALB* | NM_000477 | TGCTTGAATGTGCTGATGACAGGG  AAGGCAAGTCAGCAGGCATCTCATC |  | 162 |  |
| *SERPINA1* | NM_000295.5 | GATCAACGATTACGTGGAGAAGG  CCTAAACGCTTCATCATAGGCA |  | 207 |  |
| *APOB* | NM_000384 | AGCGTTCACCGATCTCCATCTG  TCAGATTCCCGGACCCTCAACT |  | 204 |  |
| *CDH1* | NM_004360.5 | GAGTGCCAACTGGACCATTC  ACCCACCTCTAAGGCCATCT |  | 82 |  |

- 1. **Biological samples**

| **Description** | **Source** | **Identifier** |
| --- | --- | --- |
| Primary human hepatocytes (PHH) | Auvergne Rhone Alpes platform of primary hepatic cells, CRCL, Lyon | French South-East region IRB agreement #A16-207 |
| HCC clinical samples | French Liver Biobank network | INCa, BB-0033-00085, under IRB agreement of Inserm Ethics Committee (CEEI, #12-063) |
| Normal liver samples | CRCL, Lyon | French South-East region IRB agreement #A16-207 |
| Rat liver samples | Janvier Laboratories | Grenoble-Alpes University agreement #B 38 516 10 006 |

- 1. **Deposited data**

| **Name of repository** | **Identifier** | **Link** |
| --- | --- | --- |
| N/A | N/A | N/A |

- 1. **Software**

| **Software name** | **Manufacturer** | **Version** |
| --- | --- | --- |
| Fiji | Wayne Rasband, NIH, USA | 1.53c |
| ImageLab | Bio-Rad | 5.2.1 |
| GraphPad Prism | Dotmatics  GraphPad Software Inc. | 9 |
| NIS Elements software | Nikon, Tokyo, Japan | N/A |
| SynergyFinder Plus | Network Pharmacology for Precision Medicine in the Research Program of System Oncology, Faculty of Medicine at University of Helsinki, Finland | 3.10.3 |
| R package |  | 3.6.1 |
| Single sample gene set enrichment analysis | Genepattern | 4 |
| Seurat | Satija Lab | 4.2 |
| Gene set variation analysis | Robert Castelo, Pablo Sebastian Rodriguez and Justin Guinney | 1.44.5 |
| LightCycler96 software | Roche Diagnostics | 1.1 |
| NDP.view2 | Hamamatsu Photonics K.K | 2.9.29 |

- 1. **Other (e.g. drugs, proteins, vectors etc.)**

| **Drugs** | **Supplier** |
| --- | --- |
| Darifenacin | TargetMol |
| Scopolamine | Abmole |
| Bethanechol | TargetMol |
| Cevimelin | Selleckchem |
| Sorafenib | Abmole |
| Lenvatinib | TargetMol |
| Doxorubicin | Accord healthcare |

- 1. **Please provide the details of the corresponding methods author for the manuscript:**

| Dr. R. Parent romain.parent@inserm.fr |
| --- |

**2.0 Please confirm for randomised controlled trials all versions of the clinical protocol are included in the submission. These will be published online as supplementary information.**

| N/A |
| --- |
