# Supplementary material for: Hepatocellular carcinoma hosts cholinergic neural cells and tumoral hepatocytes harboring targetable muscarinic receptors
Source: JHEP Rep. 2024 Nov 12;7(1):101245. doi: 10.1016/j.jhepr.2024.101245 (PMC11663970; doi:10.1016/j.jhepr.2024.101245)
Supplement: Multimedia component 4 [file mmc4.pdf]

# Hepatocellular carcinoma hosts cholinergic neural cells and tumoral hepatocytes harboring targetable muscarinic receptors☆

**Charlotte A. Hernandez**[1,†], **Claire Verzeroli**[1,†], **Armando Andres Roca-Suarez**[1,†], Abud-José Farca-Luna[2], Laurie Tonon[2], Roger Esteban-Fabró[3], Roser Pinyol[3], Marie-Laure Plissonnier[4], Ievgeniia Chicherova[1], Anaëlle Dubois[1], Pascale Bellaud[5], Marine Seffals[5], Bruno Turlin[5], Alain Fautrel[5], Gabriel Ichim[6], Michel Rivoire[7], Guillaume Passot[8], Zuzana Macek-Jilkova[9,10], Thomas Decaens[9,10], Alain Viari[2], Barbara Testoni[1], Sandra Rebouissou[11], Josep M. Llovet[3,12,13], Fabien Zoulim[1,14], Romain Parent[1,*]

JHEP Reports **2025**. vol. 7 | 1–14

Check for updates

**Background & Aims:** Owing to unexplained interpatient variation and treatment failure in hepatocellular carcinoma (HCC), novel therapeutic approaches remain an urgent clinical need. Hepatic neurons, belonging to the autonomic nervous system (ANS), mediate liver/whole body crosstalk. Pathological innervation of the ANS has been identified in cancer, nurturing tumor stroma and conferring stronger carcinogenic properties.

**Methods:** We characterized the innervation of liver tumors from the French Liver Biobank, then applied bioinformatics to TCGA (The Cancer Genome Atlas), several other datasets and a European validation cohort, to re-evaluate patient stratification. Cell biology and pharmacology studies were also performed.

**Results:** Densely packed nucleated DCX$^+$, synaptophysin$^+$, NeuN$^+$, VAChT$^+$, TH$^-$, CD31$^-$, CD45$^-$ clusters, to date undetected, were identified in human HCCs, and independently confirmed by single-cell RNA sequencing data. Using the new concept of a neuronal score, human and rat HCCs displayed tightly netrin-1-associated neural reconfiguration towards cholinergic polarity, which was associated with chronic liver disease progression, cancer onset and many features of aggressive (proliferative class) HCC, including shortened survival. This score was conditioned by tumoral hepatocytes, and predicted sorafenib efficacy in the STORM HCC phase III trial. Conversely, intratumoral adrenergic lymphocytes were enriched in TEMRA and cytotoxic phenotypes. Amongst all cholinergic transcripts, the medically targeted CHRM3 receptor was enriched and associated with pathogenic traits in HCC, as well as poor prognosis in HCC stages 1-2, while its level dropped upon experimental re-differentiation. Its pharmacological inhibition with low concentrations of anticholinergic drugs, but not cholinomimetics, decreased anchorage-independent growth and anoikis, synergized with sorafenib and lenvatinib in HCC class 1 to 3 lines, yet not in primary human hepatocytes, and preserved mature hepatocyte functions.

**Conclusion:** These data identify cholinergic processes as instrumental in liver carcinogenesis and support the use of EMA/FDA-approved cholinergic drugs in HCC research.

© 2024 The Author(s). Published by Elsevier B.V. on behalf of European Association for the Study of the Liver (EASL). This is an open access article under the CC BY license (http://creativecommons.org/licenses/by/4.0/).

## Introduction

Despite the development of effective therapies against HBV and HCV, deaths related to hepatocellular carcinoma (HCC) have continued to increase. Liver comorbidities, such as MASLD (metabolic dysfunction-associated steatotic liver disease) and alcohol-related liver disease (ALD) combined with metabolic syndrome (MetALD) or not, are long-term co-operators or independent factors fostering the onset of HCC and enhancing disease heterogeneity.[1] Despite multifactorial etiologies, HCC typically develops in patients with cirrhosis. Treatments with tyrosine kinase inhibitors (TKIs) for instance lead to short-term, unavoidable relapse,[2] whereas immune checkpoint or growth factor inhibitors currently provide hope for only a fraction of patients with unresectable HCC.

In this respect, cellular/tissular structures linking the general pathophysiology of the patient with HCC are of interest, as they may uncover novel ways of stratifying patients. Several recent works and reviews[3] have highlighted the relevance of studying neural aspects of cancers in peripheral organs. For instance, pathological innervation and involvement or dysregulation of the autonomic nervous system (ANS) have been identified in ovarian, prostate, gastric and pancreatic cancers,[3,4] nurturing

☆ Given their role as Editor-in-Chief, Josep M Llovet had no involvement in the peer-review of this article and had no access to information regarding its peer-review. Full responsibility for the editorial process for this article was delegated to the Guest Editor Robert F. Schwabe.

* Corresponding author. Address: IHU Everest & Cancer Research Centre of Lyon, 151 cours Albert Thomas, Lyon, France.
*E-mail address:* romain.parent@inserm.fr (R. Parent).
† Equal contributions.

https://doi.org/10.1016/j.jhepr.2024.101245

tumor stroma and conferring stronger carcinogenic properties. Little, however, is known about the potential role of the ANS in liver oncogenesis.

The ANS comprises the sympathetic (adrenergic signaling) and parasympathetic (cholinergic signaling) arms that relay signals both ways along the brain/liver neural axis in order to regulate involuntary physiological or pathological processes. Oddly, liver nerves are seldom mentioned in anatomical reference textbooks.[4] The liver is an innervated organ that hosts afferent and efferent ANS nerves, in constant communication with the central nervous system (CNS)[5] to regulate liver functions.

As notably pointed out by Tracey's theory,[6] neural signals also regulate several processes that may impact HCC onset and growth. Portal hypertension, a recognized risk factor for HCC development and recurrence,[7] is correlated with ANS dysfunction.[8] The global orientation of liver innervation in chronic liver disease is currently debated in mice and humans,[9,10] yet these studies agree on recurrent adrenergic nerve degeneration. Conversely, cholinergic signaling was shown to attenuate apoptosis in the mouse liver[11] and foster HCC growth.[12] Interestingly, choline acetyltransferase (ChAT)$^+$ regulatory T cells and dysfunctional programmed cell death 1 (PD-1)$^+$ T cells, were observed in HCC-bearing mice.[13] The present study describes neural features in human HCC, and their likely contributions to pathogenesis. Moreover, these results identify a new neural signature that could be relevant for targeting HCC.

## Materials and methods

Expanded experimental biology methods (origin and processing of biological samples, Western blotting, gene expression, immunofluorescence, cell culture approaches, pharmacology, and RNA-seq processing) and statistical methods are detailed in the supplementary file.

### Neuronal receptor score calculation and cohort classification

Gene set scores were calculated using single-sample gene set-enrichment analysis (GSEA) for bulk transcriptomic data[14] and gene set-variation analysis for single-cell transcriptomic data.[15] Here, two gene set scores were calculated from both lists of receptors, one including all adrenergic ones, and the other all cholinergic ones, in order to obtain an adrenergic and a cholinergic score, respectively. To obtain a global neuronal receptor score (NRS), the difference (the adrenergic score minus the cholinergic score) was calculated for each sample. The use of a gene set score difference instead of a ratio of gene expression has several advantages. First, the score is computed by taking the entire transcriptome into account, thus overcoming cases where a sample is less covered. This method is common to all transcriptome profiling technologies. Second, NRS values always vary linearly with the evolution of any term of the equation, which is not the case with ratios. Finally, this method is preferential to evaluate the activity of a pathway in a sample by transcriptomics, as it is independent of the number of genes evaluated.

### Transcriptomic datasets

Single-cell RNA sequencing (scRNA-seq) data from patients with HCC was obtained from the Gene Expression Omnibus database accession GSE149614 (n = 10) and https://lambrechtslab.sites.vib.be/en/aHCC (n = 38). Bulk RNA-seq data from paired HCC and non-tumor tissues were extracted from GSE124535 (n = 35), GSE144269 (n = 70) and microarray data from GSE64041 (n = 60). Microarray data from two cohorts of mixed liver disease etiologies were obtained from GSE32879 (n = 37) and GSE89377 (n = 107). Microarray data from patients with HCC treated with sorafenib (n = 67) or placebo (n = 73) were retrieved from GSE109211. Single-nuclei RNA sequencing (snRNA-seq) from MASLD (n = 2) and healthy individuals (n = 2) from GSE174748. Bulk RNA-seq data from paired cholangiocarcinoma and non-tumor tissues from GSE107943 (n = 27), GSE119336 (n = 15) and microarray data from GSE76297 (n = 90).

## Results

### Neural progenitors of cholinergic orientation in human and rat HCC samples

Comprehensive maps of ANS features and innervation are currently lacking in HCC. Interestingly, human liver ANS innervation is more developed than in rodents, as it extends deeper into the lobule,[5] increasing its regulatory capacities and suggesting that ANS-related mechanisms observed in animals may play more important roles in patients. Human samples, obtained from the French National HCC biobank, were selected across the four major HCC etiologies (HBV, HCV, former ALD, former NASH; 24-26% each). The main characteristics of the patients are provided in Table S1. To characterize HCC innervation, the following classically validated neuron markers were considered: neuronal nuclear antigen (NeuN, phospho- and total, RBFOX3) as a mature, nuclear, neuron marker;[16] and doublecortin (DCX) and internexin neuronal intermediate filament protein alpha (INA) as immature neuron markers.[17] Additionally, tyrosine hydroxylase (TH, TY3H) for adrenergic, and vesicular acetylcholine transporter (VAChT) for cholinergic neurons,[18] were used.

We first investigated the presence of ANS markers in normal human samples (both uninfected and non-fibrotic) *vs.* cirrhotic (minimum distance of 2 cm from tumor) and tumor samples (HCC). Western blotting highlighted positive staining for DCX and INA in tumor samples, and a lower expression of the mature neural marker NeuN, strongly suggesting the presence of immature neurons. In addition, HCC samples lacked the adrenergic marker TH but showed normal expression of the cholinergic neural marker VAChT (representative large blot of 30 patients, Fig. 1A), prompting further analysis. DCX levels were sharply correlated with β-tubulin degradation, suggesting the association of neural alterations with parenchymal remodeling in human samples (Fig. 1B). In an attempt to quantify ANS dynamics, we defined a neuronal score (NS) as the difference between adrenergic and cholinergic signals (see Methods, NS = TH - VAChT). These markers evolved towards a more cholinergic orientation with disease progression (Fig. 1C-H). We then compared the expression levels of such markers in normal livers, cirrhotic and tumoral lesions in samples from the four main HCC etiologies (HBV [n = 14], HCV [n = 9], former ALD [n = 14] and former NASH [n = 14], total of 51 patients). Extensive blots are provided in Figs S1-2 and summarized in Table S2. Comprehensive technical validations for NeuN, DCX, TH and VAChT antibodies are provided in

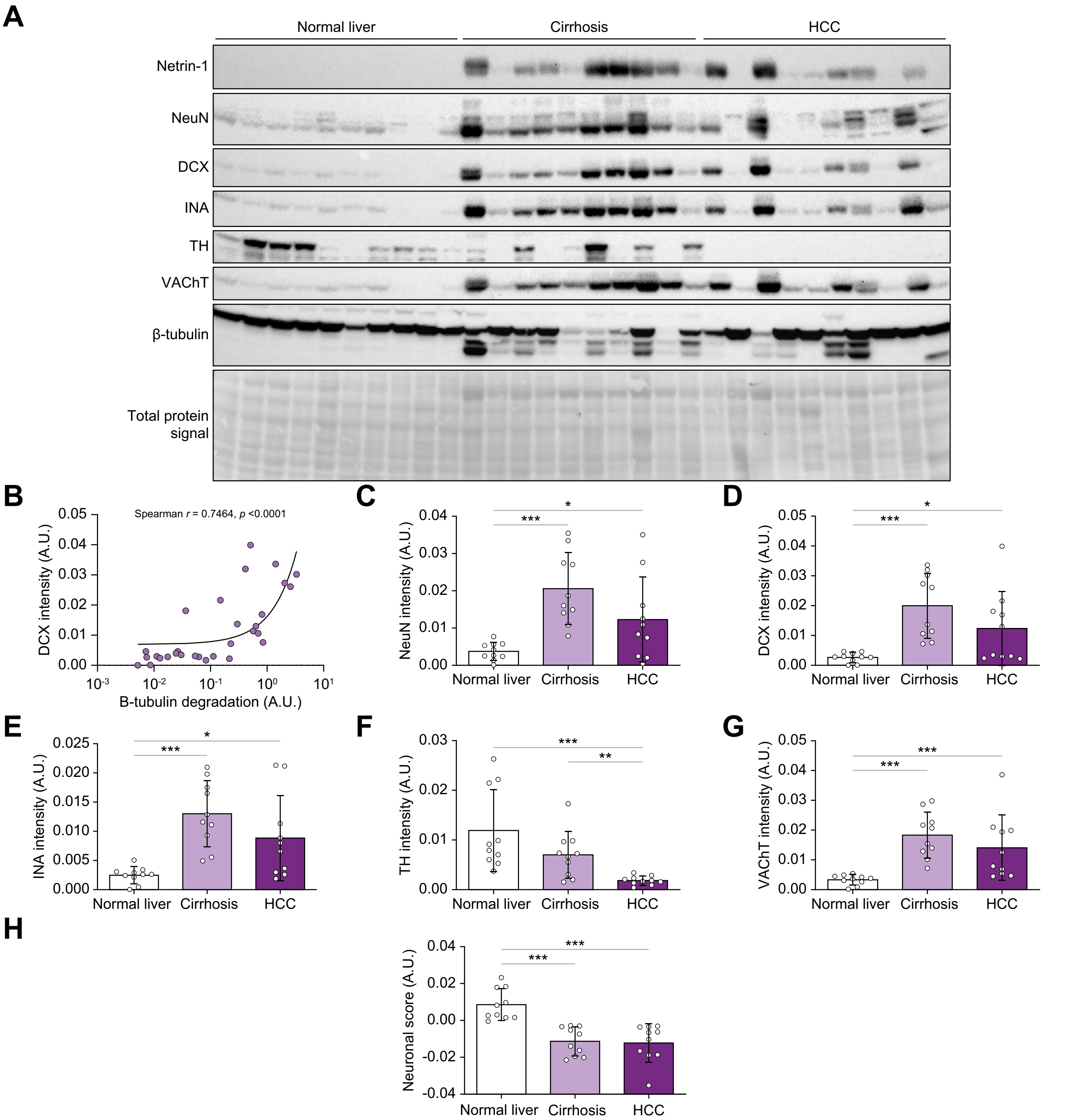


**Fig. 1. Expression of mature and progenitor neural markers in human HCC.** (A) Immunoblotting of netrin-1, NeuN, DCX and INA, TH and VAChT markers on 10 normal liver, 10 cirrhotic (F4) and 10 HCC samples. (B) DCX induction is correlated with parenchymal remodeling. DCX levels were plotted against ratios of full-length *vs.* degraded tubulin signals. Spearman test (****p* <0.001). (C–H) Signal quantification was done using total protein normalization.[41] Mann-Whitney or *t* test (after normality test, **p* <0.05, ***p* <0.01, ****p* <0.001). n = 30 patients, from the four main HCC etiologies.

Supplementary information 1. This phenotype was similar to that of the cirrhotic HCC rat model of reference (Supplementary information 2 and Figs S3-5). As in the rat, neurogenic netrin-1 expression was strongly (r >0.85) correlated with proteolyzed β-tubulin and DCX levels, as well as cirrhosis onset, and HCC (Fig. S6). Such clinical data depict cholinergic-oriented alterations of neural networks in late-stage chronic liver disease and HCC.

Given that tissue markers may change with disease progression, we sought to gain insight into the localization of neural signals in human samples. As a first approach, we performed standard fluorescence staining of a cohort of 24

tumors. These were subjected to Masson's trichrome or HES staining to expose tissue architecture, and then NeuN, DCX, TH and VAChT immunostaining coupled with DAPI staining. Technical validations are provided in Fig. S7. In accordance with blots, TH staining was negligible in both frequency and intensity throughout samples (Fig. S8A-F). Importantly, DCX and VAChT were found in the tumor bulk, where they displayed co-localization (Fig. S8G-J). In order to further confirm these findings, we probed the same samples with CD31 staining to locate vessels, CD45 to exclude leucocytes as potential sources of the signals, and synaptophysin as another independent neuron marker. As shown in Fig. S9, HCC also hosts VAChT$^+$, synaptophysin$^+$, CD45$^-$ and CD31$^-$ cells, also nucleated (consistently with NeuN staining), as in prostate cancer with muscarinic signaling.[19] Although the comprehensive phenotype of these cells remains to be characterized, these data indicate that HCC hosts neural cells of cholinergic orientation. As observed by western blotting, the predominant ANS colabeling was specific to immature DCX$^+$ fibers and cholinergic neural cells, indicating that the neural alterations highlighted herein are likely cholinergic, in line with previous data on steatohepatitis.[10] These data are consistent with the presence of cholinergic intrahepatic neural cells in the diseased liver as confirmed by snRNA-seq investigation (Supplementary information 3). Such observations were similar between HCC etiologies, and substantiated findings on other solid malignancies,[3,4] in which these tumors host nerves with migratory potential, likely tuning their interactions with postsynaptic receptors.

### Greater cholinergic orientation of ANS receptors from normal liver to HCC

The balance between adrenergic and cholinergic signals defines a unified ANS output in each innervated organ. To investigate such signals, we first defined a post-synaptic neuro-signature encompassing all adrenergic and cholinergic receptor transcripts. We thus quantified the expression of all transcripts encoding ANS receptors in HCC samples (Table S3). The previously described NS was then adapted to the post-synaptic status of such targets. Hence, its counterpart was termed 'neuronal receptor score', NRS. Functional biochemistry data pertaining to the functioning of each receptor are mostly absent in the liver or HCC. As for the NS, the NRS corresponds to the difference between the sums of all adrenergic receptors (except ADRA2, being presynaptic) and all cholinergic receptor signals, providing an integrated view of the balance between ANS receptors in the tissue. Paralleling neural data yet with a delay, the NRS decreased in low-grade dysplastic nodules and more intensely in cancer in three independent datasets based on paired or unpaired samples (Fig. 2A-C), indicating the relevance of further investigations into the relationships between the cholinergic branch of the ANS and HCC.

### Bioinformatics highlight the pathogenic implication of the cholinergic orientation in HCC evolution

To map the interplay between autonomic functions and HCC, we performed a bioinformatics study on the previously published HCC (LIHC) TCGA dataset. Salient features were then considered in an independent cohort of 171 HCC samples from a previous study,[20] hereafter referred to as the 'validation cohort'.

After NRS calculation, samples were split into two classes: those with a higher difference than median were named adrenergic and those with a lower difference than median were named cholinergic (Fig. 3A, see the Methods section). The distribution diagram of NRS values obtained and PCA (principal component analysis) projection of those two classes are shown in Fig. 3B,C. As expected, adrenergic receptors were more strongly expressed in the 'NRS > median' class, while cholinergic receptors were more strongly expressed in the 'NRS < median' class (Table S3, Fig. S10).

Then, to identify a potential association between ANS orientation and standard parameters in HCC, we tested the

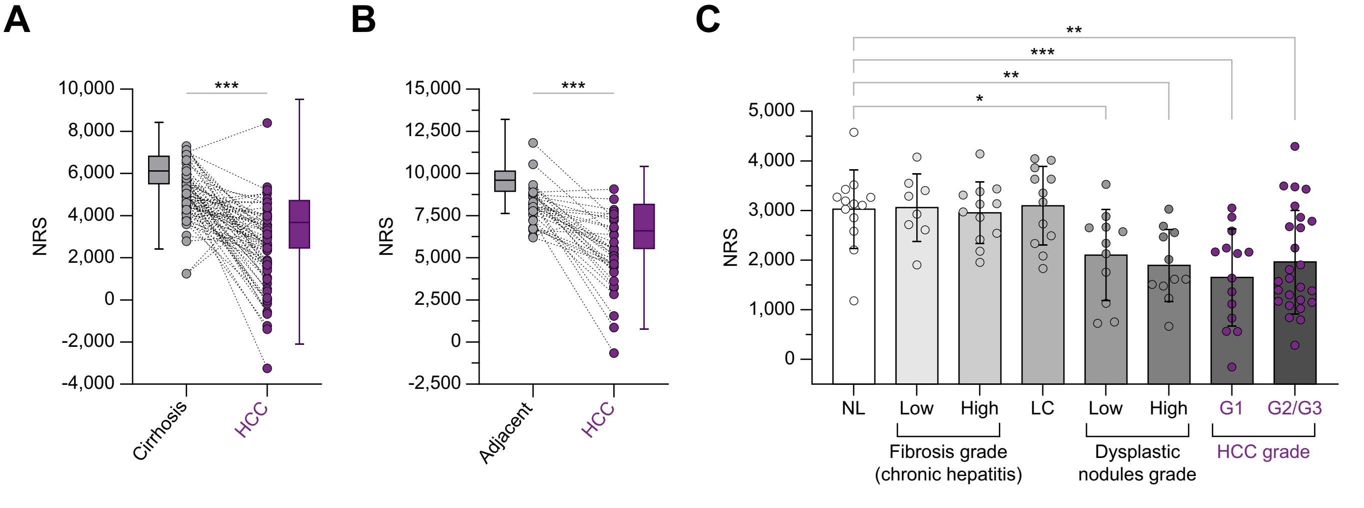


**Fig. 2. The NRS selectively decreases in HCC *vs.* all other histological states of chronic liver disease.** (A) NRS in cirrhosis *vs.* tumors. Wilcoxon matched-pairs signed rank test (***$p$ <0.001), n = 70 (GSE144269). (B) NRS in adjacent *vs.* tumors. Same test (***$p$ <0.001), n = 35 (GSE124535). (C) Comparison of NRS between stages (GSE89377). Kruskal-Wallis test corrected with a Dunn's test (*$p$ <0.05, **$p$ <0.01, ***$p$ <0.001). Bars represent mean ± SD. NL, normal liver; LC, liver cirrhosis; HCC G1-3, Edmonson grades 1-3 (n = 107 patients total). NRS, neuronal receptor score; HCC, hepatocellular carcinoma.

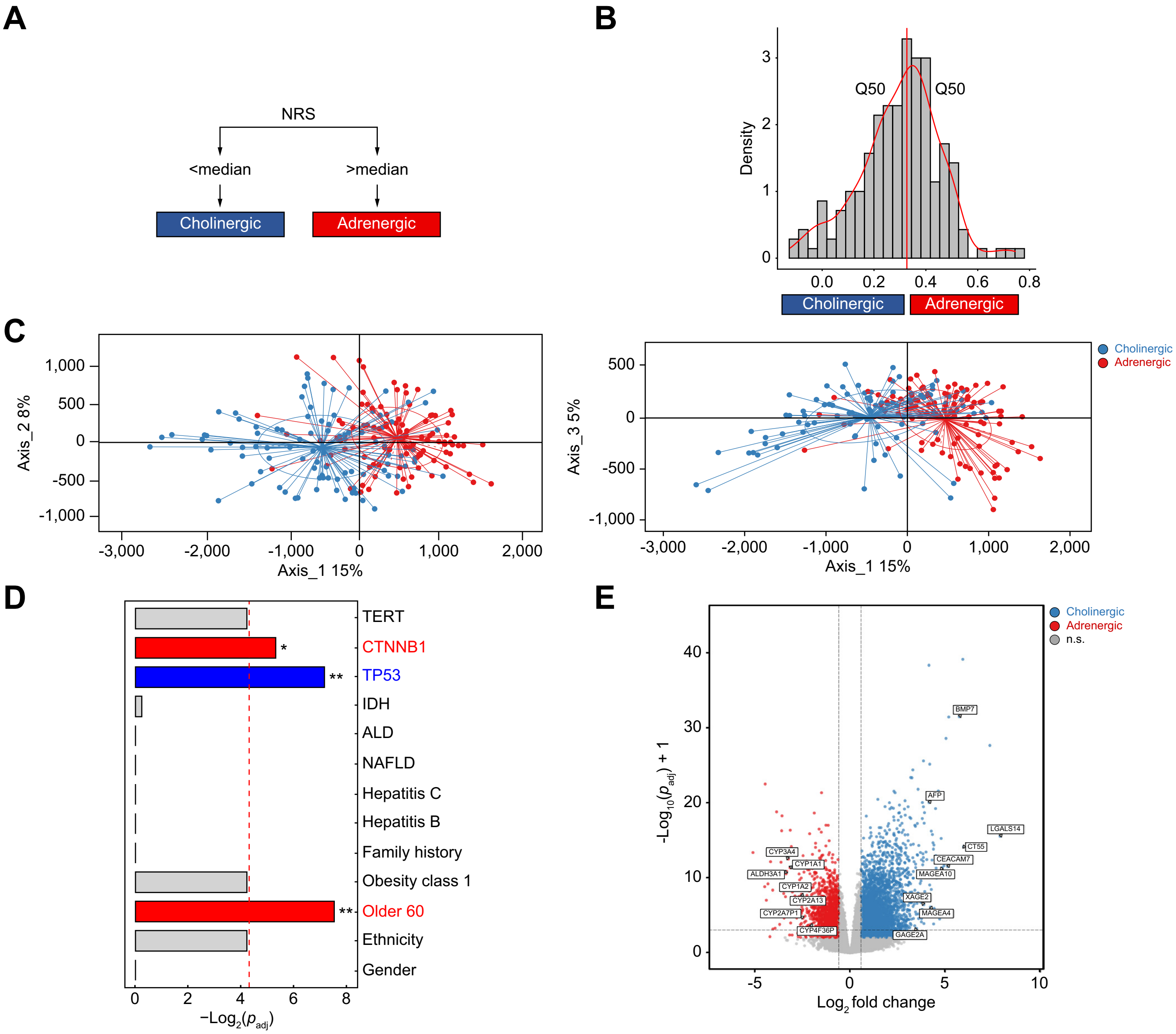


**Fig. 3. Neural classification based on adrenergic and cholinergic receptors in HCC.** (A) NRS calculation: adrenergic and cholinergic enrichment scores were calculated, then cholinergic scores were subtracted from adrenergic scores. (B) Samples were grouped into cholinergic and adrenergic classes by comparison with NRS median. (C) Sample distribution after dimensional reduction (PCA) based on the 10% most variable genes. (D) Associations between classes and main HCC clinico-biological features. Fisher test's adjusted *p* values per variable (*$p_{adj}$ <0.05, **$p_{adj}$ <0.01). (E) Volcano plot showing significantly differentially expressed genes. Horizontal red bar: adjusted *p* value threshold at 0.001. Genes with lowest adjusted *p* value are indicated. n = 196 patients (TCGA cohort). Modulated genes were considered if absolute $Log_2$ fold-change value was higher than 0.58 and $p_{adj}$ <0.001. ALD, alcoholic liver disease; NAFLD, non-alcoholic fatty liver disease.

correlation between both neuronal classes and gender, ethnicity, etiology, obesity and mutational profile (*hTERT, TP53, CTNNB1*). A Fisher's exact test comparing the signature of each class to each variable was constructed (Table S4). Importantly, no association could be seen between either neural class and gender, ethnicity, obesity, family history, or any of the four main HCC etiologies (Fig. 3D). *CTNNB1* mutations and age >60 years emerged as positively associated with the adrenergic class. *TP53* mutations emerged as positively associated with the cholinergic class. A strong trend for association between the adrenergic class and *CTNNB1* mutations was also found in the 'validation cohort' (Table S5). These data suggest that these two classes defined by this ANS-based signature may redefine the current stratification of HCC heterogeneity based on genetics. This question warrants further investigations across different disease stages and ethnic backgrounds, as suggested for instance in HCC studies on Mongolian samples.[21]

To identify genes associated with tumor ANS features, we performed differential gene expression analysis between the two neuronal classes ($p_{adj}$ <0.01 and absolute $Log_2$ fold-change >0.58). Results are illustrated in a volcano plot (Fig. 3E). Data related to the 100 most significantly up- and downregulated genes for the adrenergic and cholinergic

signatures are shown in Tables S6 and S7, respectively. Differentially expressed genes upregulated in the cholinergic signature include many dedifferentiation-related antigens, unlike transcripts enriched in the adrenergic signature (*e.g., CYP450* mRNAs, Table S8). Of importance, no *CYP450* mRNA was found in the cholinergic class. Altogether, such data support that the cholinergic signature may be correlated with less differentiated HCC tumors.

Next, to better understand the phenotypic relevance by deciphering the different molecular pathways defining adrenergic and cholinergic tumors, we performed a Hanzelmann overrepresentation analysis of gene sets, using the differentially expressed genes identified above and shown in a heatmap (Fig. 4A). Genes over-expressed in each tumor class were used as input against Hallmark gene sets of the MsigDB. On the one hand, the most enriched pathways in genes over-expressed in adrenergic tumors corresponded to differentiated, hepatocytic metabolic functions, such as XENOBIOTIC_METABOLISM (71 genes), BILE_ACID_METABOLISM (35 genes), FATTY_ACID_METABOLISM (40 genes) and PEROXISOME (22 genes) (Fig. 4B, Table S9). All these functions are linked with a *CTNNB1* mutational profile[1] that is associated with this neuronal class. As a control, the β-catenin target *GLUL* was also positively associated with this adrenergic class (+1.52 $Log_2$; rank 241). On the other hand, many pathways associated with cell cycle and proliferation were significantly enriched in cholinergic tumors. Indeed, more than 50 genes were associated with pathogenic, proliferative pathways such as, amongst others with consistent adverse, pro-mitotic, outcomes: G2M_CHECKPOINT (72 genes), E2F_TARGETS (64 genes) and EPITHELIAL_MESENCHYMAL_TRANSITION (70 genes) (Fig. 4C, Table S10), in agreement with the frequent *TP53* mutation found in these tumors. Interestingly, panels B and C corroborate genetic data of Fig. 3D. Indeed, TP53mut tumors are enriched in mitotic pathways and cholinergic lesions are also enriched in these pathways. Control pathways such as cardiac (adrenergic) and nausea/vomiting (cholinergic) pathways were identified in their expected classes. In line with differentiation data, these results suggest a more deleterious profile for the cholinergic class. Survival analyses confirmed this hypothesis, showing an association between the adrenergic class and longer overall survival within a timeframe of 4 years (Fig. 4D). Data were confirmed by GSEA on the MSigDB C2 and C5 gene set collections, that showed an enrichment in *CTNNB1* mutation-associated metabolic functions in adrenergic samples and *TP53* mutation-linked proliferative and mitogenic pathways in cholinergic samples (Figs S11-12).

Among all pathways unveiled, we focused on HCC-specific signatures known to be related to good or poor prognosis.[1] We performed single-sample GSEA quantification of all these pathways for each sample and compared the two neuronal classes using Wilcoxon's tests. Almost all of these HCC-specific signatures (Table S11) were differentially enriched between both classes (Fig. 5A). The adrenergic class was statistically associated with 17 HCC canonical signatures, 16 of which were functionally consistent with the transcriptomics of this class (*i.e.,* related to better prognosis). The cholinergic class was linked to eight HCC canonical signatures, all related to poor prognosis (Fig. 5B, Table S12). The present study yielded consistent results in the 'validation cohort' (Fig. 5C). Likewise, cholinergic polarity was repeatedly associated with increased hypoxia, a process linked to tumor aggressiveness and TKI resistance,[22] using three representative hypoxia scores (Fig. 5D-F). All these findings argue in favor of a worse prognosis for patients with HCC and higher cholinergic signaling.

### Dedifferentiated hepatocytes display low NRS values in HCC

Tumors are heterogenous in terms of cell types, and bulk analyses may not address this issue thoroughly. To provide cell-type relevance to these data, we first searched for tumor cell type(s) likely dictating cholinergic-oriented (*i.e.,* lower) NRS in HCC samples. Dedifferentiated (or 'malignant') hepatocytes, specifically, were identified as such by scRNA-seq (Figs 6A-D and S13). The 'malignant hepatocyte' signature was used as defined in a reference study.[23] Data were confirmed functionally using hepatocyte-like spheroids and a differentiation protocol for 2D cultures. Consistently with previous data, the NRS increased (*i.e.,* becomes more adrenergic) with cell differentiation (Fig. 6E-I, functional validation of re-differentiation in Fig. S14, as published elsewhere[24]) paving the way for downstream perturbation studies. Functional and pharmacological data were derived from the three classes of the currently admitted classification of HCC lines[25] after assessment of their suitability for each assay (Table S13).

### The cholinergic receptor CHRM3 participates in cancer cell growth, dedifferentiation and resistance to HCC-relevant TKIs

Within the cholinergic branch, the *CHRM3* transcript encodes a receptor targetable by the FDA- and EMA-approved drug darifenacin. *CHRM3* was frequently and strongly upregulated in tumor lesions compared to adjacent tissue in three cohorts, using cirrhotic or non-cirrhotic tissue as non-tumor controls (Fig. 7A-D). In addition, HCC was one of the few cancer types where *CHRM3* was expressed at moderate to strong immunoreactivity levels in the Protein Atlas database[26] (Supplementary information 4). *CHRM3* was also repeatedly correlated with several pathogenic hallmarks of proliferative HCC (Fig. 7E,F). To provide causal data concerning hepatocytic cells, we evaluated the sensitivity of HCC lines belonging to all classes[25] to cholinergic drugs. The soft-agar assay, which provides results in close correlation with *in vivo* HCC data in treatment studies[27,28] was used. Importantly, these lines span the entire spectrum of *CHRM3* expression in HCC lines, all of which express it robustly in the Liver Cancer Cell Line Database (https://lccl.zucmanlab.com/hcc/home), as in numerous HCC cases.[26] Data indicate that targeted functional blockade of the CHRM3 receptor using low concentrations of darifenacin (see Supplementary information 5) hampered colony formation, whereas no phenotype could be obtained using either scopolamine a non-selective muscarinic CHRM3 antagonist, or agonists (Fig. 7G,H). Since none of the class 3 HCC lines tested grew in this context, we then submitted a similar yet larger panel of HCC lines to an anoikis induction protocol. Likewise, limited doses of darifenacin displayed the most robust inhibitory capacity (Fig. 7I). To corroborate such data, we then induced polarization of 2D cultures of these HCC lines into spheroids (re-differentiation

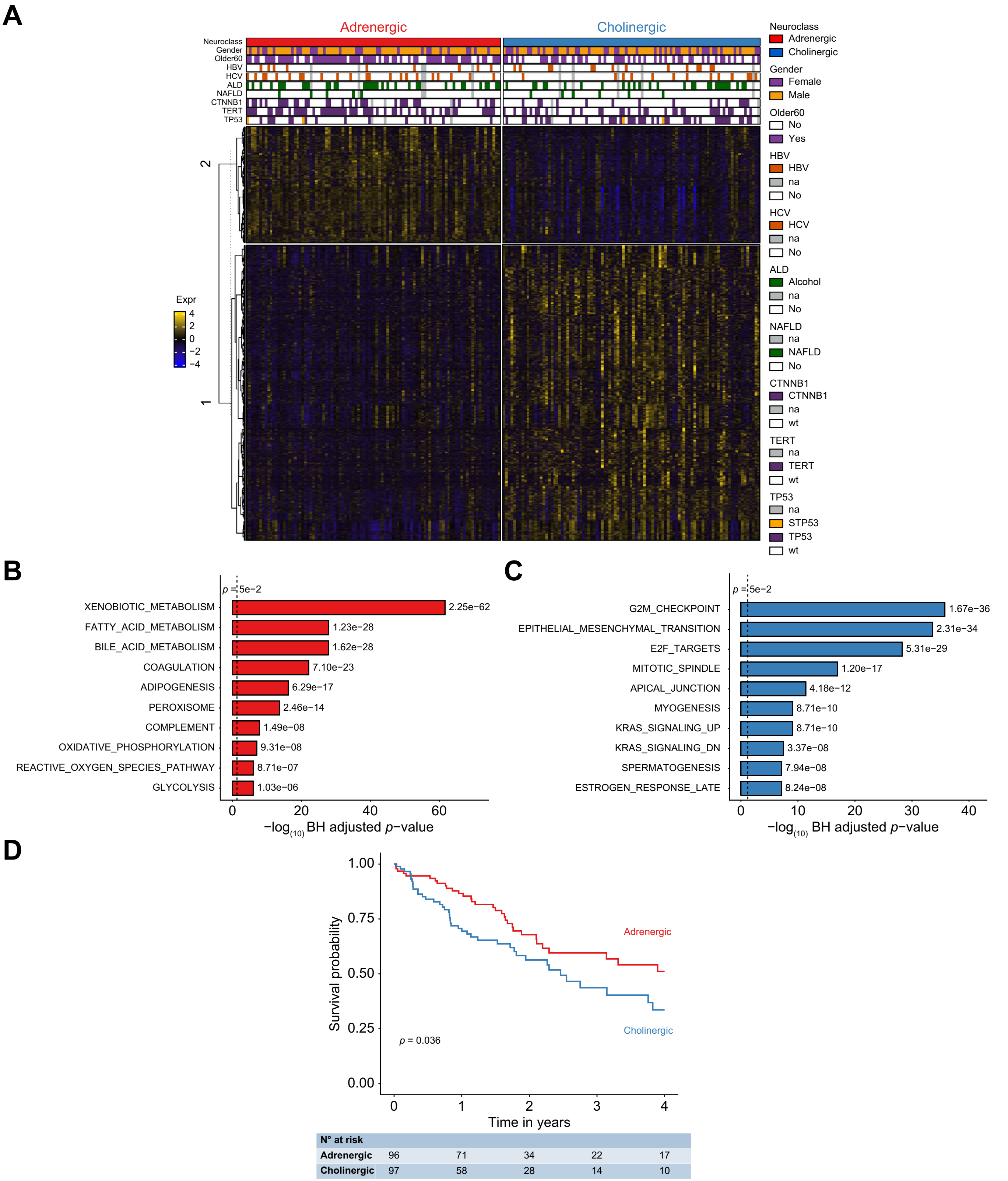


**Fig. 4. Pathway enrichment and outcome analyses.** (A) Heatmap of the normalized levels of DEGs (TCGA cohort) between both neuronal classes, showing 3,264 genes over-expressed in the cholinergic class and 1,288 in the adrenergic one. (B–C) Top 10 over-represented hallmark pathways from the over-expressed genes in the adrenergic (B) and the cholinergic (C) classes, listed in Tables S9 and S10, respectively. Pathways are ordered by adjusted *p* value. (D) Kaplan-Meier representation of the predictive value with respect to the overall survival in TCGA samples. *p* value of the log-rank test is indicated. ALD, alcoholic liver disease; HBV, hepatitis B virus; HCV, hepatitis C virus; NAFLD, non-alcoholic fatty liver disease.

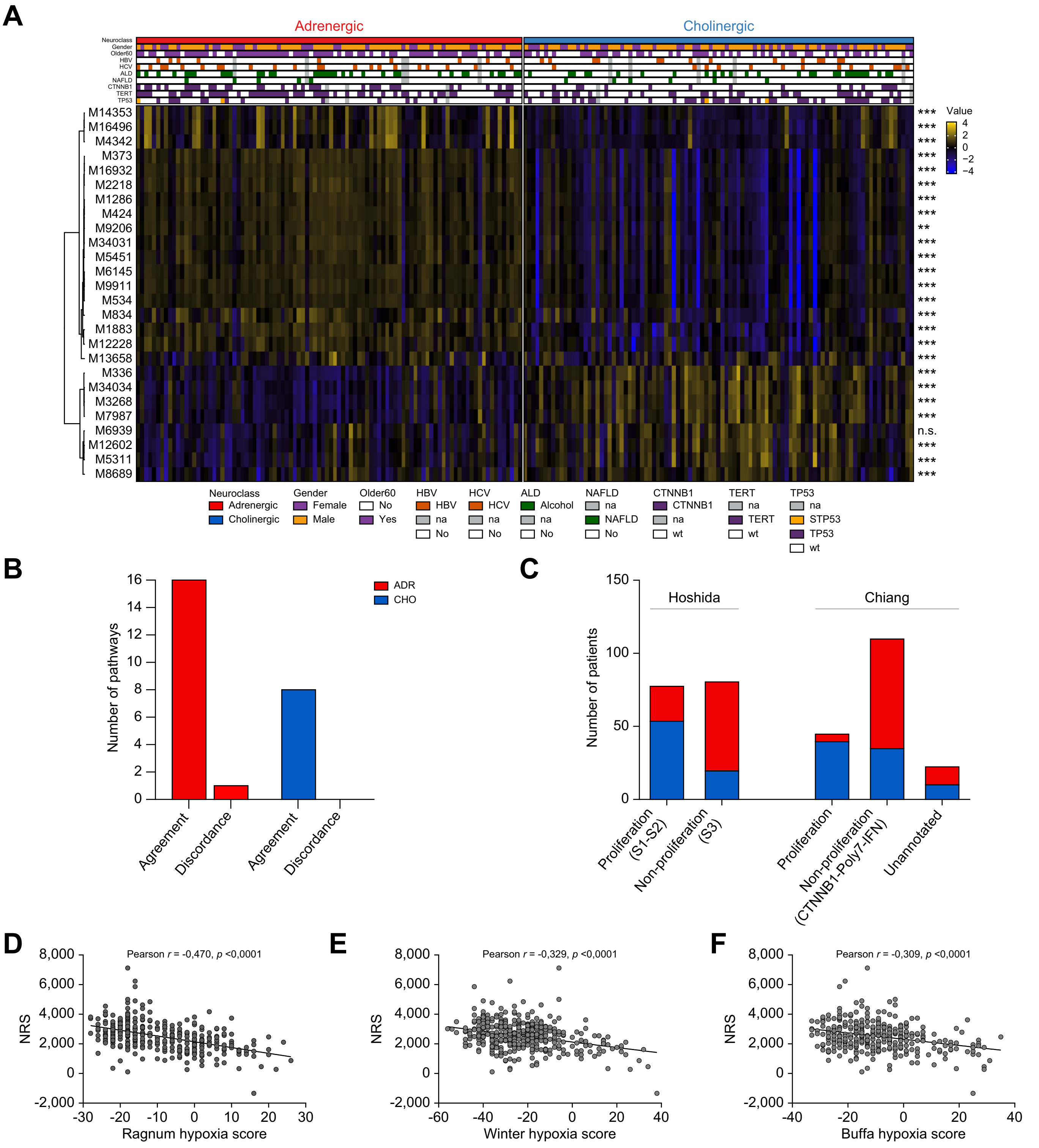


**Fig. 5. Association between adrenergic or cholinergic orientations and HCC pathological criteria.** (A) Enrichment in good or worse prognosis-associated pathways for the adrenergic and cholinergic classes, respectively. Pathway accession numbers (listed in Table S11) are shown to the left. Wilcoxon test was performed for each sample between the two neuronal classes (** $p_{adj}$ <0.01, *** $p_{adj}$ <0.001, n = 293 patients). (B) Canonical HCC pathways in accordance or discordance with adrenergic or cholinergic functional transcriptomics. Pathways associated are depicted in Table S11. (C) Verification of TCGA findings on the 'validation cohort' (n = 171 patients). (D-F) Cholinergic HCC orientation is correlated with hypoxia parameters of reference. Pearson's correlation *** $p$ <0.001, n = 366 patients, TCGA cohort). ALD, alcoholic liver disease; HBV, hepatitis B virus; HCV, hepatitis C virus; NAFLD, non-alcoholic fatty liver disease.

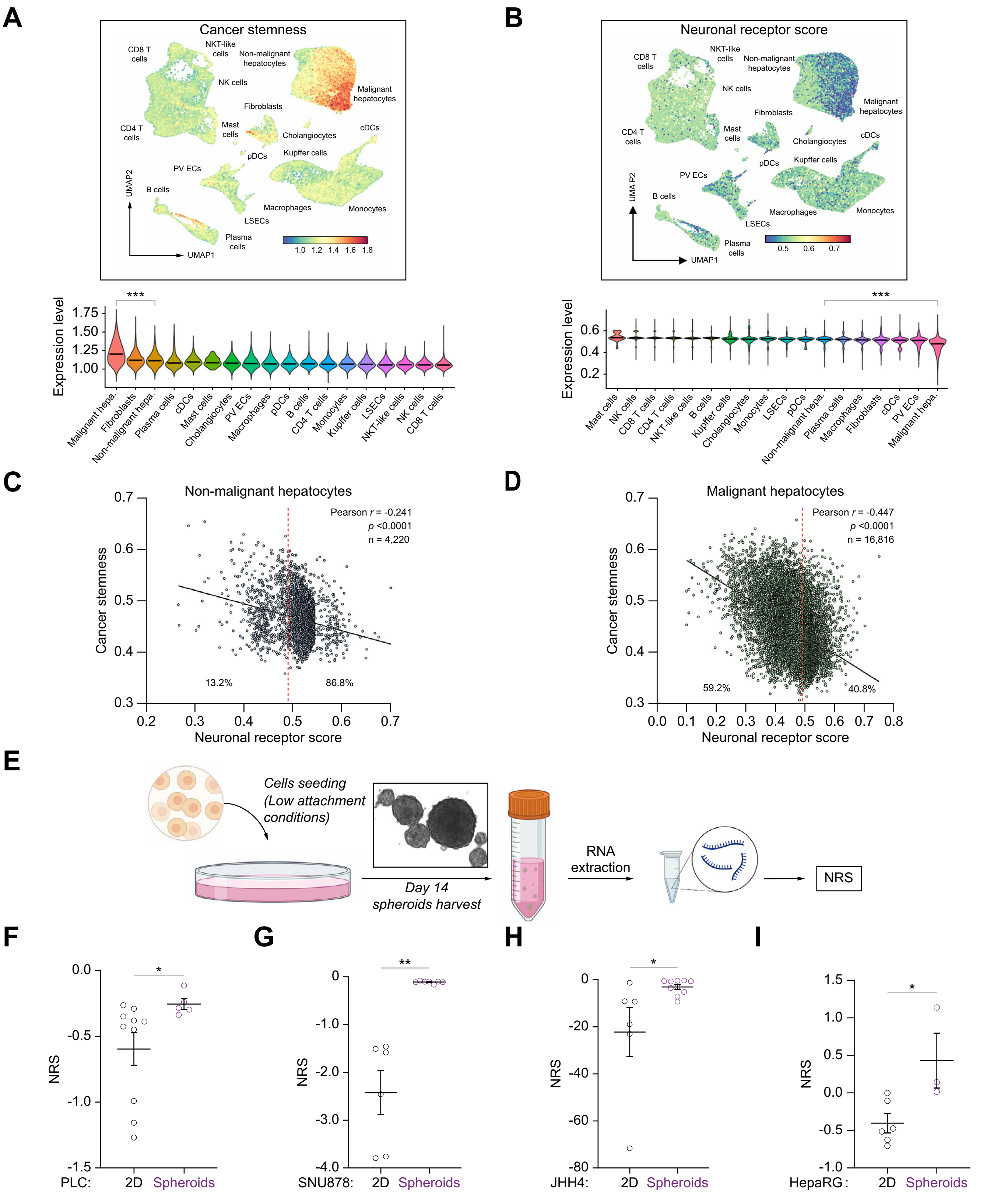


**Fig. 6. Cholinergic HCC orientation (low NRS) is conditioned by dedifferentiated hepatocytes.** (A) GSVA scores for the cancer stemness signature in each cell type, represented as UMAP (top) and violin plot (bottom). Two-tailed *t* test (****p* <0.001, GSE149614, n = 10 patients). Violin plots depict the mean of each population. (B) GSVA scores for the NRS in each cell type, represented as UMAP (top) and violin plot (bottom). Two-tailed *t* test (****p* <0.001, GSE149614, n = 10 patients). (C-D) Correlation between NRS and malignancy (cancer stemness signature) in non-malignant (C) and malignant (D) hepatocytes. Percentages denote hepatocytes above (adrenergic) or below (cholinergic) the median NRS (GSE149614). (E) Workflow. Created with BioRender. (F–I) The NRS becomes more adrenergic (*i.e.,* increases) upon experimental hepatocytic re-differentiation (PLC, SNU878, and JHH4 spheroids; n = 5-10 independent experiments) or upon HepaRG 2D differentiation; n = 3-6 independent experiments). Mann-Whitney test or *t* test (depending on normality, **p* <0.05, ***p* <0.01). NRS, neuronal receptor score.

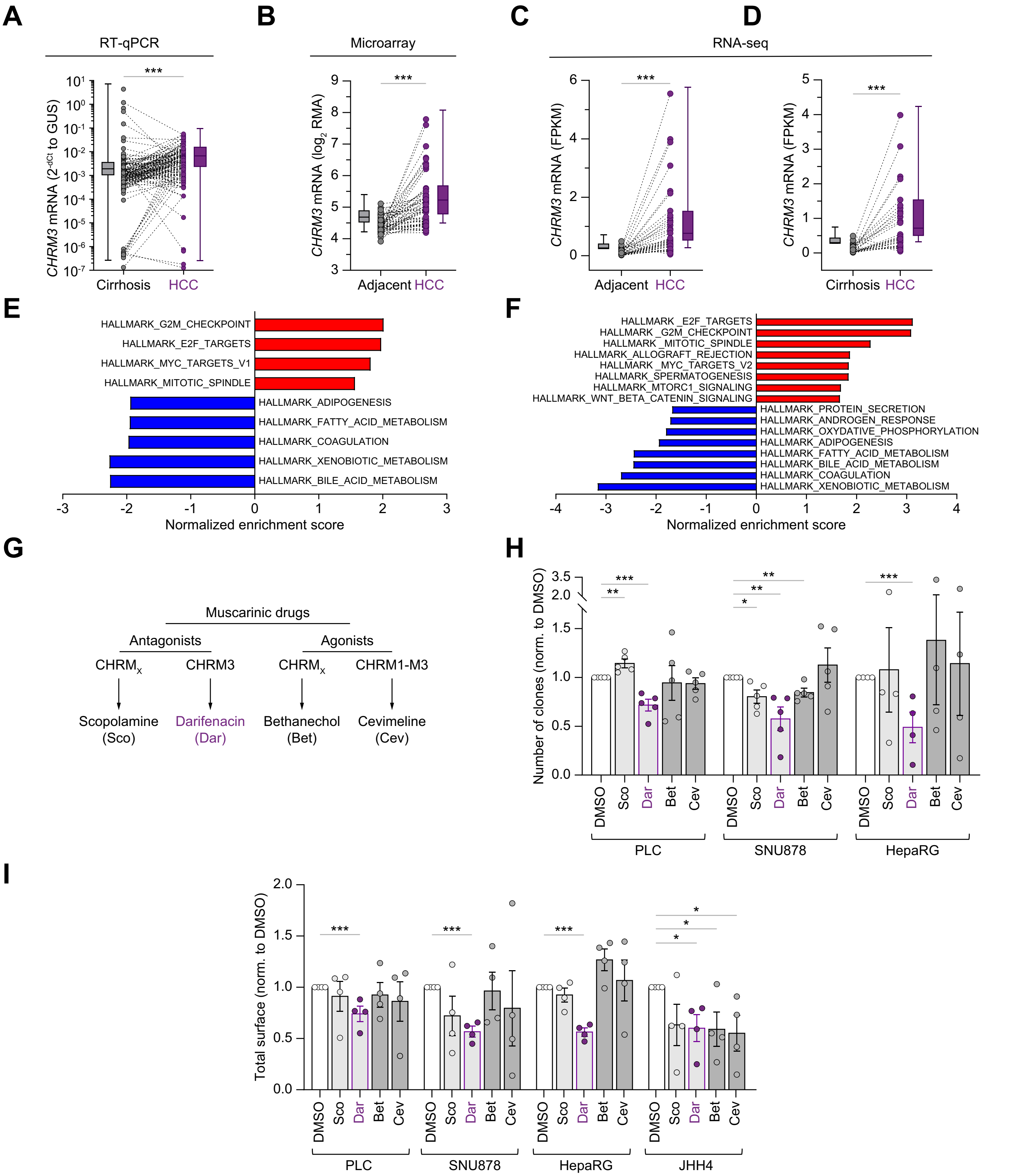


**Fig. 7. Muscarinic receptors are druggable target candidates in HCC.** (A) *CHRM3* expression (French national liver biobank, all four main etiologies 24-26% of total, paired tissues, n = 168 cases). (B) *CHRM3* expression is upregulated in HCC lesions mainly associated with alcohol abuse. ALD etiology corresponds to 72% of the cohort (paired tissues, GSE64041, n = 60 patients, Y-axis units: $Log_2$ RMA). (C-D) *CHRM3* expression is upregulated in HCC lesions (paired tissues, GSE124535, n = 35 patients, Y-axis units: FPKM). (B–C) All adjacent tissues; (D) cirrhotic adjacent tissues only. (A-D) Wilcoxon matched-pairs signed rank test, ***$p$ <0.001. (E-F) $CHRM3^{high}$ tumors are correlated with aggressive HCC. Patients were classified into the highest 25 percentile ($CHRM3^{high}$) and lowest 25 percentile ($CHRM3^{lo}$). GSEA was performed using the MSigDB gene sets H, C2 and C5. Figures show normalized enrichment scores from differentially modulated hallmark gene sets in $CHRM3^{high}$ tumor samples (FDR <0.05). (G) Dendrogram describing muscarinic drugs. (H–I) Soft agar (n = 4 or 5) and anoikis (n = 4) assays were conducted on PLC, SNU878, HepaRG and JHH4 (JHH4 unsuitable for soft-agar assay). Student's *t* test after normality test, *$p$ <0.05, **$p$ <0.01, ***$p$ <0.001. HCC, hepatocellular carcinoma.

previously validated in Fig. S14). Conversely, we observed an important drop (>10-fold) in *CHRM3* transcript levels, indicating that it correlated with hepatocytic differentiation (Fig. S15). Because of this dataset, we then evaluated the potential poor prognostic status of *CHRM3* expression in HCC using the 'kmplot' interface (https://kmplot.com) that considers the LIHC TCGA cohort. When specifically analyzing HCC samples at stages 1 or 1+2 (*i.e.*, early or well-differentiated cases, n = 171 or n = 253 patients, respectively), significance was reached (log-rank $p = 0.042$ and 0.023, Fig. S16). CHRM3-mediated pathogenic contribution to early carcinogenesis in particular deserves further investigation, as its targeting could be beneficial to patients receiving locoregional therapy. Altogether, this dataset identifies the CHRM3 receptor as implicated in the pathogenic properties of HCC cells.

In patients with HCC, TKIs have objective but limited efficacy over time due to near unavoidable tumor escape.[1] We tested a possible association between low NRS (*i.e.* cholinergic tumor orientation as a marker of proliferation) and sorafenib activity in the context of the STORM HCC clinical trial, aimed at evaluating sorafenib as an adjuvant therapy for early HCC after resection or local ablation.[29] Molecular characterization of this cohort identified patients benefiting from sorafenib in terms of objective sensitivity ('sorafenib responders') and patients for whom sorafenib had no effect ('non-responders').[30] In this context, specifically in the sorafenib-treated group, a low NRS was associated with sensitivity to the drug (Fig. S16). This result is in agreement with sorafenib's multikinase inhibitor status, affecting, amongst others, the MAPK/ERK pathway, which notably targets HCC cell proliferation[31] and further supports the relevance of the NRS and cholinergic cues in the disease. Acquired resistance to sorafenib is an issue in HCC research.[22] Consequently, HCC lines were subjected to accepted synergy assays between neuroactive drugs and two HCC-relevant TKIs (Chou-Talalay derived ZIP method[32]). These assays identified a synergistic relationship between sorafenib and scopolamine (Fig. 8A-C), as a pan-muscarinic inhibitor. We then searched for the implicated receptor using darifenacin and reproduced this dataset, indicating that the M3 receptor supports this phenotype (Fig. 8D-F). Quantitative values of average synergy scores are provided in Fig. 8G. Data were mostly confirmed with the co-first-line TKI lenvatinib (Fig. S17). Highlighting the importance of inhibiting such function, no activity could be observed upon usage of muscarinic agonists (Fig. S18). Primary human hepatocytes used as controls yielded neither sensitivity to cholinergic drugs nor synergy after combination with TKIs (Fig. S19). Moreover, in the HepaRG differentiation model,[33] none of these drugs displayed dedifferentiation effects on tested exocrine or endocrine functions, considering bile canaliculi counts, hepatocyte nuclear factor 4-$\alpha$ DNA binding levels, or secreted albumin levels (Fig. S20). The CHRM3 receptor functionality was verified (Supplementary information 5). These functional data confirm the known absence of hepatotoxicity of these drugs in the clinic, as well as their known absence of hepatic drug interaction with any of the TKIs of interest herein. Of note, highly similar cholinergic phenotypes were observed in cholangiocarcinoma (Supplementary information 6). Altogether, these results indicate the compatibility of these strategies with cancer-predisposed, even if functionally weakened, livers.

## Discussion

Intra- and interpatient heterogeneity is a major challenge for HCC research. It is fueled by a plethora of combinations between etiologies, differentiation grades, immune features, genetics, and histological subtypes. Like many other organs, the liver permanently communicates with the brain through afferent and efferent nerves.[5] It is therefore possible to propose that patient neurological features interact or interfere with intrahepatic neural processes, including in HCC. Genetic mutations are discrete events, likely caused by local, mutagenic events that have escaped overwhelmed DNA repair pathways in a context of chronic proliferation. As an ANS-influenced value, the NRS likely fluctuates at the cross-roads of hepatic and systemic influences longitudinally in a single patient, and across patients as well. This suggests that the NRS may constitute a basis for the development of whole organism-sensitive criteria for HCC stratification. However, the causal relationship between mutations and evolution of the NRS values remains undefined, warranting *in vivo* experiments in the future. The main limitations of our study are two-fold. First, none of the six anti-CHRM3 antibodies tested by western blotting or flow cytometry was found to be RNAi- or Crispr/Cas9 sensitive, hampering loss-of-function studies. Second, although both neural classes identified herein were clearly associated with stronger (cholinergic-oriented) or weaker (adrenergic-oriented) pathogenic HCC phenotypes, where higher *ADRA2B* expression is related to good prognosis,[34] proteomic confirmation of these findings on a large set of ethnically diverse patients[21] will be necessary to confirm RNA data. The NRS is a brain/body-conditioned, physiologically integrative, quantitative index that may help identify ANS drugs in any innervated organ or tissue. Our data enrich the current landscape of predictive transcriptomic signatures, since, beyond the traditionally admitted genetic criteria, current stratification based on NRS distribution identified a druggable set of cholinergic receptors. Charting their expression (including *CHRM3*'s) upon HCC recurrence will be of substantial clinical interest as well.

In the liver, Walter Cannon's (1915) *fight-or-flight* model, historically accepted to describe ANS functions,[35] predicts that adrenergic signaling mobilizes intracellular hepatocytic energy pools for peripheral energetic needs, whereas cholinergic signaling fosters intrahepatic nutrient storage and related processes, such as liver expansion. This model seems relevant in HCC, where liver expansion also implicates an increase in liver cell size.[36] Frequent comorbidities associated with liver carcinogenesis are excessive body mass index and alcohol intake (as an important energetic source). This suggests that cholinergic signals aiming at fostering liver expansion, due to their implication in the *rest-and-digest* related functions,[37] could be hijacked by the tumor in a context of excessive nutrient availability. As was shown recently in metabolic dysfunction-associated steatohepatitis,[10] adrenergic innervation of HCC seems to be weaker than any other stage. Here, cholinergic tumors are associated with many poor prognosis-related pathways. The likely protective and adverse roles of coffee[38] and tobacco,[38,39] respectively, as adrenergic and cholinergic agonists, support these findings.

The second hallmark of this study consists in the identification of (i) muscarinic receptors as *bona fide* targets for

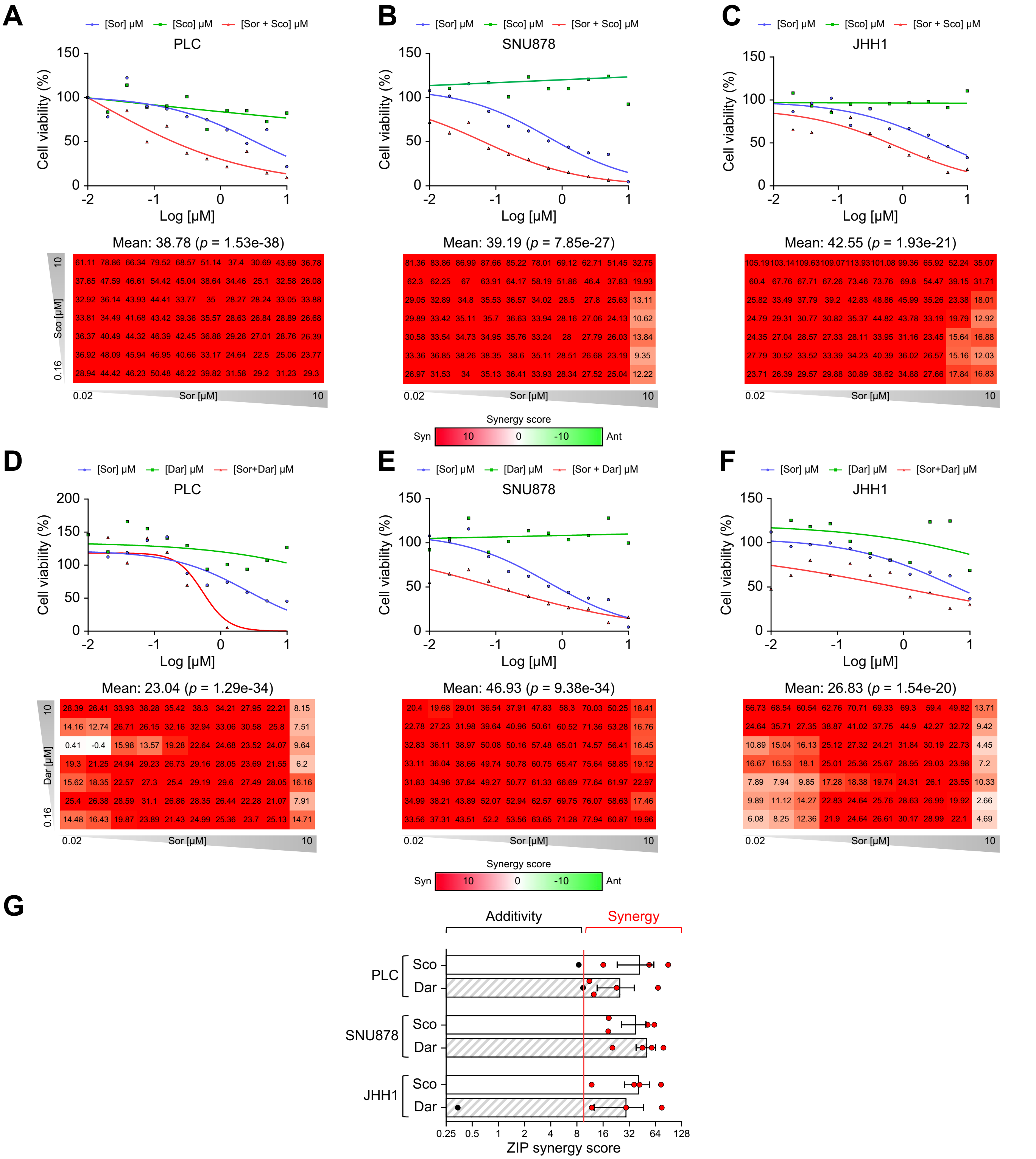


**Fig. 8. Muscarinic blockade synergizes with the first-line TKI sorafenib.** (A-F) Activity curves and 2D matrices on scopolamine (A-C) and darifenacin (D-F), respectively on PLC (class 1), SNU878 (class 2) and JHH1 (class 3) HCC lines. Chou-Talalay ZIP scores >10 indicate synergy. (G) Plate-wide average ZIP synergy score values calculated from 96-well matrices for each experiment (n = 4-6). Dar, darifenacin; Sco, scopolamine.

impeding HCC cell resistance to first-line HCC TKIs, (ii) their predictive status with respect to sorafenib response in an adjuvant therapy setting, and (iii) the applicability of this approach, which generates no unwanted effects on primary human hepatocyte viability or major hepatocyte functions. The differential effects of M3-selective and pan-muscarinic

inhibition in anchorage-independent growth assays *vs.* synergy assays are discussed in Supplementary information 7. On the immune side, two studies implicate general cholinergic[13] and CHRM3-specific signaling events[40] in tumor lymphocytes. While the former depicts a stimulating role for acetylcholine esterase-expressing lymphocytes in these processes,[13] the latter instead suggests, in accordance with our findings, a deleterious role for cholinergic inputs in general and on $CD8^+$ T cells when mediated by the murine CHRM3 receptor. Moreover, our analysis of publicly available scRNA-seq data derived from intra-tumoral immune populations suggests that $CD8^+$ T effector memory re-expressing CD45RA (TEMRA) and cytotoxic NK cells are the sub-populations with the strongest adrenergic polarization, in contrast to exhausted $CD8^+$ T cells that present markedly lower NRS levels (Supplementary information 8). We believe that the diverging conclusions between studies may be related to, as acknowledged by Zheng *et al.*,[13] the differential involvement of the strong diversity of cholinergic receptors. Both studies however suggest future important discoveries in the field of ACh and HCC immunity.

This study documents a new neural contribution in the pathogenesis of human HCC and describes its potential clinical implications for HCC, classification of other liver cancers and patient stratification. This approach identified targets that have been engaged by EMA- and FDA-approved medicines for decades, and that are therefore adequate for subsequent HCC research aimed at improving patient care.


## Affiliations

[1]*Hepatitis Viruses and Pathobiology of Chronic Liver Diseases – LabEx DEVweCAN, Inserm U1052, Cancer Research Centre of Lyon – Hepatology Institute of Lyon F – IHU EVEREST, University of Lyon 1, ISPB, France, CNRS UMR5286, Centre Léon, Lyon, France;* [2]*Fondation Synergie Lyon Cancer, Gilles Thomas Bioinformatics Plateform, Centre Léon Bérard, F-69008 Lyon, France;* [3]*Translational Research in Hepatic Oncology Group, Liver Unit, Institut d'Investigacions Biomèdiques August Pi i Sunyer (IDIBAPS), Hospital Clínic, University of Barcelona, Barcelona, Catalonia, Spain;* [4]*Epigenetics, Microenvironment, and Liver Cancer, U1052, Cancer Research Centre of Lyon – Hepatology Institute of Lyon – IHU EVEREST, University of Lyon 1, ISPB, CNRS UMR5286, F-69083 Lyon, France, Centre Léon Bérard, Lyon, France;* [5]*H2P2 platform, University of Rennes, Rennes, France;* [6]*Cancer Cell Death team – LabEx DEVweCAN, Inserm U1052, Cancer Research Centre of Lyon, F-69003 Lyon, France, University of Lyon, F-69003 Lyon, University of Lyon 1, ISPB, Lyon, F-69622, France, CNRS UMR5286, F-69083 Lyon, France, Centre Léon Bérard, F-69008 Lyon, France;* [7]*Department of Surgical Oncology, Centre Léon Bérard, F-69008 Lyon, France;* [8]*Hospices Civils de Lyon, Service of Gastroenterology, F-69600 Oullins, France;* [9]*Institute for Advanced Biosciences, Inserm U1209, University of Grenoble-Alpes, F-38700 La Tronche, France;* [10]*Service d'hépato-Gastroentérologie, Pôle Digidune, CHU Grenoble-Alpes, 38700 La Tronche, France;* [11]*Centre de Recherche des Cordeliers, Inserm, Sorbonne Université, USPC, Université Paris Descartes, Université Paris Diderot, Paris, France;* [12]*Mount Sinai Liver Cancer Program, Division of Liver Diseases, Tisch Cancer Institute, Icahn School of Medicine at Mount Sinai, New York, NY, USA;* [13]*Institució Catalana de Recerca i Estudis Avançats (ICREA), Barcelona, Catalonia, Spain;* [14]*Hospices Civils de Lyon, Service of Hepato-Gastroenterology, F-69001 Lyon, France*


## Abbreviations

ANS, autonomic nervous system; ALD, alcohol-related liver disease; CHRM3, cholinergic receptor muscarinic 3; CNS, central nervous system; DCX, doublecortin; GSEA, gene set-enrichment analysis; INA, internexin neuronal intermediate filament protein alpha; NeuN, neuronal nuclear protein; NRS, neuronal receptor score; NS, neuronal score; ScRNA-seq, single-cell RNA sequencing; TH, tyrosine hydroxylase; TKI, tyrosine kinase inhibitor; VAChT, vesicular acetylcholine transporter.


## Financial support

This work was supported by INCa (PRT-K 19-033, PLBIO 23-031), La Ligue contre le cancer (France, Comité du Rhône, #R19147CC), financial support from ITMO Cancer of Aviesan within the framework of the 2021-2030 Cancer Control Strategy, on funds administered by Inserm (PCSI call), and the DevWeCan Laboratories of Excellence network (ANR-LABX-061) and the French National Research Agency (ANR) within the framework of the IHU EVEREST (ANR-23-IAHU-0008) as part of the program "Investissements d'Avenir". AARS is the recipient of an ANRS grant (ECTZ206376). IC is the recipient of an ANRS predoctoral fellowship (ECTZ63958). JML is supported by grants from the European Commission (Horizon Europe-Mission Cancer, THRIVE, Ref. 101136622), by an Accelerator Award from Cancer Research UK, Fondazione per la Ricerca sul Cancro (AIRC) and Fundación Científica de la Asociación Española Contra el Cáncer (FAECC) (HUNTER, Ref. C9380/A26813), by the NIH (R01-CA273932-01, RO1DK56621 and RO1DK128289), the Samuel Waxman Cancer Research Foundation, by the Spanish National Health Institute (Project PID2022-139365OB-I00, funded by MICIU/AEI/10.13039/501100011033 and FEDER), by the Asociación Española Contra el Cáncer (Proyectos Generales: PRYGN223117LLOV; Reto AECC 70% Supervivencia: RETOS245779LLOV), the Generalitat de Catalunya (AGAUR, 2021-SGR 01347), and from "la Caixa" Banking Foundation. JML is supported by the Fundació de Recerca Clínic Barcelona -IDIBAPS and a grant from the Spanish National Health Institute (MICINN, PID2022-139365OB-I00, funded by MICIU/AEI/10.13039/501100011033 and FEDER).


## Conflict of interest

Research Support to JML: Bayer Pharmaceuticals, Eisai Inc, Bristol-Myers Squibb and Ipsen. Consultancy (JML): Bayer HealthCare Pharmaceuticals, Eisai Inc, Merck, Bristol-Myers Squibb, Eli Lilly, Roche, Genentech, Ipsen, Glycotest, AstraZeneca, Omega Therapeutics, Mina Alpha, Boston Scientific, Exelixis, Bluejay, Captor Therapeutics.

Please refer to the accompanying ICMJE disclosure forms for further details.

## Authors' contributions

CAH, CV, AARS, IC, AD, AJFL, LT, MS, PB, ZMJ, RE, RPo, and RP generated and analyzed experimental data. PB, GI, GP, AF and ZMJ developed critical approaches and methods for the study. LT, BrT, AF, ZMJ, TD, BaT, SR, AV, JML, and FZ critically amended and conceptually enriched the paper. RP wrote the paper.

## Data availability statement

De-identified individual participant data will be made available upon request 3 months after publication for a period of 5 years after the publication date.


## Acknowledgements

We thank the Lyon regional platform for liver tissues (namely M. Michelet and J. Molle) as well as C. Caron de Fromentel for help with fresh primary liver samples. We thank C. Machu, Nikon France, for help with quantification of microscopy images. We also thank A. Sebillot, H2P2 platform, Rennes, for assistance. Non-TCGA clinical samples are from the French Liver Biobanks network (INCa, BB-0033-00085). We also thank B. Manship for English editing.


## Supplementary data

Supplementary data to this article can be found online at https://doi.org/10.1016/j.jhepr.2024.101245.

## References

*Author names in bold designate shared co-first authorship*


[1] Llovet JM, Kelley RK, Villanueva A, et al. Hepatocellular carcinoma. Nat Rev Dis Primers 2021;7:6.

[2] Llovet JM, Montal R, Sia D, et al. Molecular therapies and precision medicine for hepatocellular carcinoma. Nat Rev Clin Oncol 2018;15:599–616.

[3] Monje M, Borniger JC, D'Silva NJ, et al. Roadmap for the emerging field of cancer neuroscience. Cell 2020;181:219–222.

[4] Zeldovich L, Manish V. Cancer has a lot of nerve. Cold Spring Harbor Stories and Media; 2022 Aug 22.
[5] Jensen KJ, Alpini G, Glaser S. Hepatic nervous system and neurobiology of the liver. Compr Physiol 2013;3:655–665.
[6] **Pavlov VA**, **Tracey KJ**. Neural circuitry and immunity. Immunol Res 2015;63:38–57.
[7] Ganne-Carrie N, Chastang C, Chapel F, et al. Predictive score for the development of hepatocellular carcinoma and additional value of liver large cell dysplasia in Western patients with cirrhosis. Hepatology 1996;23:1112–1118.
[8] Dumcke CW, Moller S. Autonomic dysfunction in cirrhosis and portal hypertension. Scand J Clin Lab Invest 2008;68:437–447.
[9] **Liu K**, **Yang L**, **Wang G**, et al. Metabolic stress drives sympathetic neuropathy within the liver. Cell Metab 2021;33:666–675 e664.
[10] Adori C, Daraio T, Kuiper R, et al. Disorganization and degeneration of liver sympathetic innervations in nonalcoholic fatty liver disease revealed by 3D imaging. Sci Adv 2021;7.
[11] Nishio T, Taura K, Iwaisako K, et al. Hepatic vagus nerve regulates Kupffer cell activation via alpha7 nicotinic acetylcholine receptor in nonalcoholic steatohepatitis. J Gastroenterol 2017;52:965–976.
[12] **Fu Y**, **Ci H**, Du W, et al. CHRNA5 contributes to hepatocellular carcinoma progression by regulating YAP activity. Pharmaceutics 2022;14.
[13] Zheng C, Snow BE, Elia AJ, et al. Tumor-specific cholinergic CD4(+) T lymphocytes guide immunosurveillance of hepatocellular carcinoma. Nat Cancer 2023;4:1437–1454.
[14] Barbie DA, Tamayo P, Boehm JS, et al. Systematic RNA interference reveals that oncogenic KRAS-driven cancers require TBK1. Nature 2009;462:108–112.
[15] Hanzelmann S, Castelo R, Guinney J. GSVA: gene set variation analysis for microarray and RNA-seq data. BMC Bioinformatics 2013;14:7.
[16] Maxeiner S, Glassmann A, Kao HT, et al. The molecular basis of the specificity and cross-reactivity of the NeuN epitope of the neuron-specific splicing regulator, Rbfox3. Histochem Cell Biol 2014;141:43–55.
[17] Bond AM, Ming GL, Song H. Adult mammalian neural stem cells and neurogenesis: five decades later. Cell Stem Cell 2015;17:385–395.
[18] Bolis LLJ, Govoni S. Handbook of the autonomic nervous system in Health and disease. New-York: Marcel Dekker Inc; 2002.
[19] Mauffrey P, Tchitchek N, Barroca V, et al. Progenitors from the central nervous system drive neurogenesis in cancer. Nature 2019;569:672–678.
[20] **Montironi C**, **Castet F**, **Haber PK**, et al. Inflamed and non-inflamed classes of HCC: a revised immunogenomic classification. Gut 2022;72:129–140.
[21] **Torrens L**, **Puigvehi M**, Torres-Martin M, et al. Hepatocellular carcinoma in Mongolia delineates unique molecular traits and a mutational signature associated with environmental agents. Clin Cancer Res 2022;28:4509–4520.
[22] Ladd AD, Duarte S, Sahin I, et al. Mechanisms of drug resistance in HCC. Hepatology 2023;79:926–940.
[23] **Miranda A**, **Hamilton PT**, Zhang AW, et al. Cancer stemness, intratumoral heterogeneity, and immune response across cancers. Proc Natl Acad Sci U S A 2019;116:9020–9029.
[24] **Gunness P**, **Mueller D**, Shevchenko V, et al. 3D organotypic cultures of human HepaRG cells: a tool for in vitro toxicity studies. Toxicol Sci 2013;133:67–78.
[25] **Caruso S**, **Calatayud AL**, **Pilet J**, et al. Analysis of liver cancer cell lines identifies agents with likely efficacy against hepatocellular carcinoma and markers of response. Gastroenterology 2019;157:760–776.
[26] Consortium PA. https://www.proteinatlas.org/ENSG00000133019-CHRM3/pathology. Protein Atlas Website 2003.
[27] **Song H**, **Yu Z**, **Sun X**, et al. Androgen receptor drives hepatocellular carcinogenesis by activating enhancer of zeste homolog 2-mediated Wnt/beta-catenin signaling. EBioMedicine 2018;35:155–166.
[28] **Bera R**, **Chiou CY**, Yu MC, et al. Functional genomics identified a novel protein tyrosine phosphatase receptor type F-mediated growth inhibition in hepatocarcinogenesis. Hepatology 2014;59:2238–2250.
[29] Bruix J, Takayama T, Mazzaferro V, et al. Adjuvant sorafenib for hepatocellular carcinoma after resection or ablation (STORM): a phase 3, randomised, double-blind, placebo-controlled trial. Lancet Oncol 2015;16:1344–1354.
[30] Pinyol R, Montal R, Bassaganyas L, et al. Molecular predictors of prevention of recurrence in HCC with sorafenib as adjuvant treatment and prognostic factors in the phase 3 STORM trial. Gut 2019;68:1065–1075.
[31] Wilhelm SM, Adnane L, Newell P, et al. Preclinical overview of sorafenib, a multikinase inhibitor that targets both Raf and VEGF and PDGF receptor tyrosine kinase signaling. Mol Cancer Ther 2008;7:3129–3140.
[32] Yadav B, Wennerberg K, Aittokallio T, et al. Searching for drug synergy in complex dose-response landscapes using an interaction potency model. Comput Struct Biotechnol J 2015;13:504–513.
[33] Gripon P, Rumin S, Urban S, et al. Infection of a human hepatoma cell line by hepatitis B virus. Proceedings Natl Acad Sci USA 2002;99:15655–15660.
[34] Hoshida Y, Villanueva A, Kobayashi M, et al. Gene expression in fixed tissues and outcome in hepatocellular carcinoma. N Engl J Med 2008;359:1995–2004.
[35] Jansen AS, Nguyen XV, Karpitskiy V, et al. Central command neurons of the sympathetic nervous system: basis of the fight-or-flight response. Science 1995;270:644–646.
[36] Sengupta S, Peterson TR, Laplante M, et al. mTORC1 controls fasting-induced ketogenesis and its modulation by ageing. Nature 2010;468:1100–1104.
[37] LeBouef T, Yaker Z, Whited L. Physiology, autonomic nervous system. Treasure Island (FL): StatPearls; 2022.
[38] **Schulze K**, **Imbeaud S**, **Letouze E**, et al. Exome sequencing of hepatocellular carcinomas identifies new mutational signatures and potential therapeutic targets. Nat Genet 2015;47:505–511.
[39] Kolly P, Knopfli M, Dufour JF. Effect of smoking on survival of patients with hepatocellular carcinoma. Liver Int 2017;37:1682–1687.
[40] Bauer KCTR, Ruf B, Myojin Y, et al. The gut microbiome controls liver tumors via the vagus nerve. Biorxiv 2024;2024. 01.23.576951.
[41] Brooks HL, Lindsey ML. Guidelines for authors and reviewers on antibody use in physiology studies. Am J Physiol Heart Circ Physiol 2018;314:H724–H732.

Keywords: HCC; autonomic nervous system; neuronal score; transcriptomics; scRNA-seq; cholinergic; M3 muscarinic receptor; spheroids; synergy; TKI resistance.
*Received 27 February 2024; received in revised form 23 September 2024; accepted 10 October 2024; Available online 12 November 2024*

Journal of Hepatology, Volume *7*

## Supplemental information

# Hepatocellular carcinoma hosts cholinergic neural cells and tumoral hepatocytes harboring targetable muscarinic receptors

Charlotte A. Hernandez, Claire Verzeroli, Armando Andres Roca-Suarez, Abud-José Farca-Luna, Laurie Tonon, Roger Esteban-Fabró, Roser Pinyol, Marie-Laure Plissonnier, Ievgeniia Chicherova, Anaëlle Dubois, Pascale Bellaud, Marine Seffals, Bruno Turlin, Alain Fautrel, Gabriel Ichim, Michel Rivoire, Guillaume Passot, Zuzana Macek-Jilkova, Thomas Decaens, Alain Viari, Barbara Testoni, Sandra Rebouissou, Josep M. Llovet, Fabien Zoulim, and Romain Parent

# Hepatocellular carcinoma hosts cholinergic neural cells and tumoral hepatocytes harboring targetable muscarinic receptors

Charlotte A. Hernandez, Claire Verzeroli, Roca-Suarez AA, Abud-José Farca-Luna, Laurie Tonon, Roger Esteban, Roser Pinyol, Marie-Laure Plissonnier, Ievgeniia Chicherova, Anaëlle Dubois, Pascale Bellaud, Marine Seffals, Bruno Turlin, Alain Fautrel, Gabriel Ichim, Michel Rivoire, Guillaume Passot, Zuzana Macek-Jilkova, Thomas Decaens, Alain Viari, Barbara Testoni, Sandra Rebouissou, Josep M. Llovet, Fabien Zoulim, Romain Parent

Table of contents

Expanded materials and methods ....................................................................2

Supplementary figures....................................................................................10

Supplementary tables.....................................................................................31

Supplementary information.............................................................................37

Supplementary references...............................................................................56

## EXPANDED MATERIALS AND METHODS

### Clinical liver samples

HCC samples used in this study were obtained from the French Liver Biobank network (INCa, BB-0033-00085, under IRB agreement of Inserm Ethics Committee (CEEI, #12-063) and the TCGA Research Network (https://www.cancer.gov/tcga) HCC-LIHC cohort. The table presenting expression data was downloaded with the tool TCGA biolinks http://bioconductor.org/packages/release/bioc/html/TCGAbiolinks.html Data were crossed with previously reported metadata to obtain a cohort of 193 patients. Normal liver samples (French South-East region IRB agreement #A16-207) obtained from safety margins of hepatic resections of colorectal cancer metastasis, were histologically normal and devoid of HBV or HCV infection. All research was conducted in accordance with both the Declarations of Helsinki and Istanbul. All research was approved by the appropriate ethics and/or institutional review committee(s), and written consent was given in writing by all subjects.

### Rat liver samples

Methods conform to the ARRIVE guidelines. 6-week-old Fischer 334 male rats (Janvier Laboratories) were acclimated for two weeks, given diethyl-nitrosamine (DEN) at 50 mg/kg weekly from day 0 to week 14, to foster progression from chronic liver disease to fibrosis, cirrhosis, and HCC. The experimental unit was a cage of 3 to 4 animals for a total of 28 animals that were fed *ad libitum*. One to two experimental units were allocated to each group for a total of 28 rats. Five rats represented the untreated group. Seven rats represented the DEN+ fibrotic group. Eight rats represented the DEN+ cirrhotic group, likewise in the DEN+ HCC group. Previous publications on this model indicate that at least 7 rats are necessary in DEN-treated groups for robust statistics. No rat was excluded from the analysis. Mixing of all groups in similar proportions was used to allocate experimental units to control and to each treatment group. Animals were sedated using ketamine prior to sacrifice. Rat outcome was measured using the molecular markers depicted in **Fig. S4**. Spearman and Mann-Whitney tests were used under GraphPad/Prism. The status of the data related to the null hypothesis was considered. Rat samples were obtained under the Grenoble-Alpes University agreement #B 38 516 10 006. No conflict of interest interfered with the study's design.

**Western blotting**

Immunoblotting was performed using 40 µg of lysates processed in lysis buffer (50 mM Tris HCl (pH 8.0), 150 mM NaCl, 1% NP-40, 0.5% sodium deoxycholate, 0.1% SDS, 10 mM sodium fluoride, 50 mM orthovanadate, 1X protease inhibitor cocktail (Roche))-processed cell lysates, then resolved on 8 or 10% SDS-PAGE, blotted onto nitrocellulose membranes (Amersham Biosciences, Saclay, France), blocked using 5% low fat dried milk in TBS Tween 0.1% for 1 h at room temperature (RT) and probed overnight at 4°C with corresponding antibodies listed in the **CTAT Table**. After three washes in TBS-Tween 0.1%, membranes were incubated for 1 h at RT with secondary antibodies coupled to HRP (1/5000, Sigma-Aldrich, St-Quentin, France) prior to chemiluminescence-based visualization using the Clarity Western ECL substrate (Bio-Rad, Versailles, France). Total protein levels were used for normalization because of high variability of housekeeping protein signals in clinical samples, as verified in the present study. Quantification was done on non-saturated images using Fiji and ImageLab software.

**Total RNA extraction and RT-qPCR**

Total RNA was extracted using Trizol (Invitrogen). RNA samples (1µg) were DNAse I-digested (Promega, Charbonnières, France) and reverse transcribed using SuperScript VILO reverse transcriptase (ThermoFischer, Les Ulis, France) according to the manufacturer's instructions. Quantitative real-time PCR was performed on $1/5^{th}$ diluted samples on a LightCycler 96 device (Roche, Meylan, France) using the No Rox qPCR mix (Bioline, Paris, France) or using a microfluidic qPCR device (Biomark™ Standard Biotools, San Francisco, USA). PCR primer sequences (5′-3′) and qPCR conditions are listed in **CTAT Table**. Specificity of all primers was assessed by melting curve analyses and agarose gel electrophoresis. Efficacy of all primers was quantified using 3-fold serial dilutions of target templates.

**Immunofluorescence**

All reagents were from Sigma-Aldrich (St. Quentin Fallavier, France) unless otherwise stated. Samples were fixed for 24 h in 4% formaldehyde (pH 7.0). Progressive dehydration was performed using ethanol and xylene through 70, 80, and 95% ethanol (45 min each), followed by 3 changes of 100% ethanol (1 h each). Tissue was cleared through 2 changes of xylene, for 1 h each. Tissue was then immersed in 3 changes of paraffin, for 1 h each. Inclusion was

automatically performed in a Histocentre3 Shandon (Loughborough, United Kingdom) device prior to cutting 4 µm-thick sections, transferred to SuperFrost slides (Euromedex, Souffelweyersheim, France), and dehydrated at 56°C for 1 h. For Masson's trichrome staining, the whole procedure was performed in a Leica ST5020 (Jena, Germany) apparatus. Paraffin removal was done using xylene and ethanol (backwards compared to protocol described above), followed by water rinsing. Gill hematoxylin was then added for 10 min followed by water rinsing. Saturated lithium carbonate was then used for 5 s before rinsing in water. Hydrogen chloride (0.5%) was then added for a few seconds to stain specimens in pink before another cycle of lithium carbonate. Fuschin Ponceau was then added for 5 min, and phosphomolybdic acid was added for 10 s before 5 min incubation. Lastly, a Light Green staining was done for 5 min, followed by acetified water for 30 s. Slides were finally dehydrated and mounted as depicted above. Final acquisition of images was done using the NanoZoomer Digital Pathology software (Hamamatsu, Massy, Japan). For immunofluorescence *per se*, the Discovery Ultra device (Roche, Illkirch, France) was used. Paraffin removal was done for 8 min (75°C) using the EZPrep reagent (Roche, Illkirch, France), followed by antigen retrieval (8 min / 95°C then 28 min / 100°C in Tris EDTA pH 8.0), followed by incubations of primary antibodies (**CTAT Table**), prior to washing three times with PBS + 0.1% Tween-20 and incubation of secondary antibodies (60 min, 37°C, see figure legend) and identical washing. The Discovery DCC Kit 455 (Roche), the Discovery FAM Kit 505 (Roche), the Discovery Cy5 Kit 660 (Roche), and the Opal Polaris 780 Reagent (Akoya) were used. Counterstaining was performed using DAPI (250 ng/mL) before mounting on SuperFrost slides (Euromedex, Souffelweyersheim, France). Final acquisition of images was done using the NanoZoomer Digital Pathology software (Hamamatsu, Massy, France).

**Cell culture**

Cells were grown in a 5% $CO_2$ humidified atmosphere at 37°C. All reagents for cell culture were purchased from ThermoFischer Scientific (Courtaboeuf, France) unless otherwise indicated. Primary human hepatocytes (PHH) were isolated from the regional platform of primary hepatic cells. They were seeded in 96-well plates previously coated with collagen 0.1% in PBS at a density of $2.10^5/cm^2$, and grown in Williams' medium supplemented with 5 mg/mL insulin (Sigma, Saint-Quentin, France), $5.10^{-5}$ mol/L hydrocortisone hemisuccinate (Merck, Fontenay, France), 2 mM glutamax, 100 IU/mL penicillin, 100 µg/mL streptomycin, 5% fetal bovine serum (FBS) (Hyclone/Perbio, Velizy, France) and 2% DMSO (Sigma-Aldrich). Four different HCC cell lines (PLC, SNU878, JHH4, JHH1) obtained from J. Zucman-Rossi's

laboratory (Inserm, Paris, France) were used after complete STR characterization. All shared common features with the proliferative class of HCC. PLC (CVCL_0485) are hepatoblast-like cells belonging to the CL1 subgroup, SNU878 (CVCL_5102) and HepaRG (CVCL_9720) are mixed epithelial-mesenchymal cells belonging to the CL2 subgroup, JHH1 (CVCL_2785) belong to the CL3 subgroup or are unclassified depending on the criteria of interest. PLC, SNU878, JHH1, JHH4 were cultured in Dulbecco's Modified Eagle Medium (DMEM) supplemented with 10% FBS, 100IU/mL penicillin, 100 µg/mL streptomycin, 2 mM Glutamax, Sodium Pyruvate and 1X MEM Non-Essential Amino Acid Solution. Cells were seeded at a density of $3.10^4/cm^2$. HepaRG cells were seeded at a density of $4.10^4/cm^2$ in William's E medium containing insulin, hydrocortisone hemisuccinate, 100IU/mL penicillin, 100 µg/mL streptomycin, 2 mM Glutamax and 10% FBS. Differentiation of HepaRG cells consisted in 14 days of culture in conventional medium followed by 14 additional days of culture in the same medium supplemented with 1.8% DMSO, as described [1].

**Soft Agar**

Cells were seeded at the following densities: PLC: $10.500/cm^2$, SNU878: $21.000/cm^2$, HepaRG: $6.500/cm^2$ in 6-well plates. Six wells were used per condition. 2X medium was reconstituted from powder (ThermoFisher Scientific, 52100021), supplemented with sodium bicarbonate (3.7 g/L, Gibco, 25080094), and above-mentioned supplements were added to each above-mentioned medium. Lower and upper agarose layers were constituted of 1 and 0.3% agar (Sigma, A9414) in complete medium, respectively, before being overlaid with 2 mL of complete medium. Culture was performed for 4 weeks. Fresh medium (2 mL) was added to the wells after 2 weeks. Cells were treated with 2 µM of the indicated drugs one day after seeding. Treatments then consisted in 1 µM of each drug twice a week. Clones were stained with 0.005% crystal violet in 10% ethanol.

**Anoikis assay**

Cells were seeded at the following densities. PLC: $1.250/cm^2$, SNU878: $2.000/cm^2$, HepaRG: $1.250/cm^2$, JHH4: $2.000/cm^2$ in 6-well plates. Six wells were used per condition in a low attachment setting. Culture was performed for 2 weeks. Cells were treated with 2 µM of the indicated drugs one day after seeding. Treatments then consisted in 1 µM of each drug twice a week. At the end of the experiment, micrographs were taken and the total surface (due to colonies fusions and budding processes) of live colonies was measured using the NIS Elements

software, NIS.ai (Nikon, Tokyo, Japan).

**Generation of spheroids**

Seeding densities were: PLC: 8.000/cm$^2$, SNU878 and JHH1: 16.000/cm$^2$. Cells were cultivated in 100-mm Petri dishes on an agarose layer (1%) for 2 weeks overlaid with 20 mL complete medium. Spheroids were harvested by aspiration of medium and low speed (1.000xg) centrifugation.

**Drug treatment**

Prior to treatment, cells were seeded in 96-well plates (5,000 cells/well for PLC; 10,500 cells/well for SNU878 and JHH1) and allowed to adhere overnight before treatment the next day. The cytotoxic effect of tyrosine kinase inhibitors (TKIs, *i.e.*, sorafenib and lenvatinib) and four cholinergic drugs (scopolamine, darifenacin, bethanechol and cevimeline) in single and combination studies was evaluated after 72 h of treatment. All experiments were carried out using duplicate 96-well plates. First, the half-maximal inhibitory concentration ($IC_{50}$) value was determined for each drug alone in all HCC cell lines, using a concentration ranging from 0 to 10 µM. Combination studies were then performed by combining TKIs and each cholinergic drug in the same range of concentrations (0 to 10 µM). Control treatment was DMSO. Doxorubicin (10 µM) was used as a control for death induction.

**Cell viability assay (MTT)**

The effects of single treatments and of neuroactive drugs and TKI combinations on the viability of HCC cell lines and PHH were evaluated using MTT (AbMole, Brussels, Belgium). After 72 h of treatment, 20 µL of MTT solution (10 mg/mL in PBS) was added to each well, including one set of wells devoid of cells as a background control. Cells were incubated in the dark for 3.5 h at 37°C. Then 100 µL/well of DMSO was added to solubilize the formazan crystals prior to a 15 minutes agitation step. Absorbance was measured at 570 nm on a Thermo Scientific Multiskan GO device.

**Analysis of drug interactions**

To quantify drug interactions between darifenacin/scopolamine and TKIs, viability data derived from neuroactive drugs and TKI combinations were analyzed using the SynergyFinder Plus software [2] (U. of Helsinki, Finland). The principle of dose matrix analysis in drug

combination studies is that each dose of one drug is combined with each dose of the second drug and thus varying dose ratios provide the basis for the final determination of the type of drug interaction. The SynergyFinder Plus software estimates the synergy scores of the drug pairs using four different reference models: Highest Single Agent (HSA), Loewe, Bliss and zero interaction potency (ZIP) scores. Synergy scores $<-10$ mean that the interaction between two drugs was likely to be antagonistic; from $-10$ to 10, the interaction was likely to be additive; and if $>10$ the interaction was likely to be synergistic. ZIP synergy scoring was used in this study (calculation of the expected effect of two drugs assuming that they do not potentiate).

**Evaluation of hepatocytic differentiation**

DNA binding capacities of endogenous HNF4α were quantified using the Abcam functional kit (Abcam, ab207208) according to the manufacturer’s instructions. Secreted human albumin was quantified after low-speed (1000xg) centrifugation using the Abcam human albumin specific kit (Abcam, ab179887) according to the manufacturer’s instructions. Absence of appreciable signal in naive (unconditioned) complete medium was verified prior to drawing conclusions. As for the quantification of bile canaliculi densities, a previously published protocol was used [3]. The cells were incubated for 10 min in a medium supplemented with 10 μg/mL of fluorescein diacetate. After changing to a fresh medium, the cells were incubated to excrete fluorescein for 20 min. Then, after moving the cells to fresh medium, the cells were incubated for a further 30 min to allow excretion to occur. Fluorescent foci were then counted (3 random fields per well, 3 well per biological condition) using DAPI stained nuclei for normalization and the Fiji software.

**Sc/snRNA-seq data processing**

Integration of samples was performed using the *FindIntegrationAnchors* and *IntegrateData* functions of Seurat [4]. Cell clustering was performed using the *FindClusters* function of Seurat and a resolution of 0.5 for the major cell types and 2 for sub-clustering. Marker genes for each of the clusters were identified with the *FindMarkers* function of Seurat. Gene set variation analysis (GSVA) [5] was employed to assign activity estimates for the NRS, neuronal signaling pathways and the cancer stemness signature [6]. Cell type labels were assigned to each cluster using the markers identified with Seurat, the cancer stemness signature scores and a list of previously described cell type-specific markers [7]. Activities of the NRS and the cancer

stemness signature were compared between malignant and non-malignant hepatocytes using an unpaired two-tailed T-test.

**Bulk RNA-seq and microarray data processing**

Differential gene expression was performed with the DESeq2 Bioconductor R package relative to previous clustering (classes by synaptic receptor, adrenergic-cholinergic signature). The adjusted $p$-value was set to 0.01 and Log2 fold change (FC) abs > 0.58. The "High" (adrenergic) class was considered as reference. For the analysis of datasets GSE124535, GSE89377, GSE144269 and GSE109211, data was pre-processed with the *CollapseDataset* tool available at GenePattern. Activity of the NRS was estimated using single-sample gene set enrichment analysis (ssGSEA). For the TCGA LIHC cohort, after crossing with mRNA data, miRNA quantitative data for 189 samples was obtained. As for the mRNA, a differential expression analysis with DESeq2 was done in order to compare features obtained upon clustering the adrenergic and cholinergic signature classes.

**Pathway enrichment analysis**

Overrepresentation analysis of pathways was performed with the enricher function from the clusterProfiler R package, using as input over- and under-expressed genes previously obtained by differential gene expression analysis. Hallmark gene sets from the MSigDB were used, and pathways enriched with an adjusted $q$-value < 0.1 were considered. For GSEA, the R package fgsea was used. Genes were pre-ranked by the signal to noise ratio between cholinergic and adrenergic samples, and the enrichment analyses were performed on the C2 and C5 gene set collections from the MsigDB.

**Statistics**

Statistical analyses and tests pertaining to the bioinformatics analysis were carried out with the R software and GraphPad Prism. Heatmaps were generated with ComplexeHeatmap, principal component analysis was completed with ade4, and plotted with factoextra or ggplot. Gaussian finite models were performed with Cluster and ClusterProfiler. Figures were created using the R software. Neuronal receptor scores (NRSs) were compared between paired adjacent and tumor tissues in the datasets GSE124535 and GSE144269 using a Wilcoxon matched-pairs signed rank test. NRSs were compared between liver disease stages in the dataset GSE89377

using a Kruskal-Wallis test corrected for multiple comparisons with a Dunn's test. NRSs in the dataset GSE109211 were compared between responders and non-responders to therapy using a Kruskall-Wallis test. Statistics on samples derived from the French National HCC biobank cohort were done as follows. Normal distribution of data was first assessed using the Shapiro-Wilk test. The associations between receptor transcript levels and clinico-pathological variables were determined in multiple comparison by Kruskal-Wallis test, Mann-Whitney test and Spearman correlation. For the TCGA LIHC cohort, paired tissues were analyzed with the Wilcoxon test. Association between the NRS and the Ragnum, Winter and Buffa hypoxia scores [8-10] was evaluated using a Pearson correlation coefficient. Kaplan-Meier plots and log-rank tests were used to evaluate the prognostic value of the receptors by univariate Cox proportional hazards regression model. The survival data (OS, PFI) were extracted from Liu *et al.* [11]. Survival analyses were conducted with survival [12, 13] and survminer [14] R packages. The survival analysis was performed at 2, 3, 5 and 10 years. $p<0.05$ was considered significant.

## SUPPLEMENTARY FIGURES

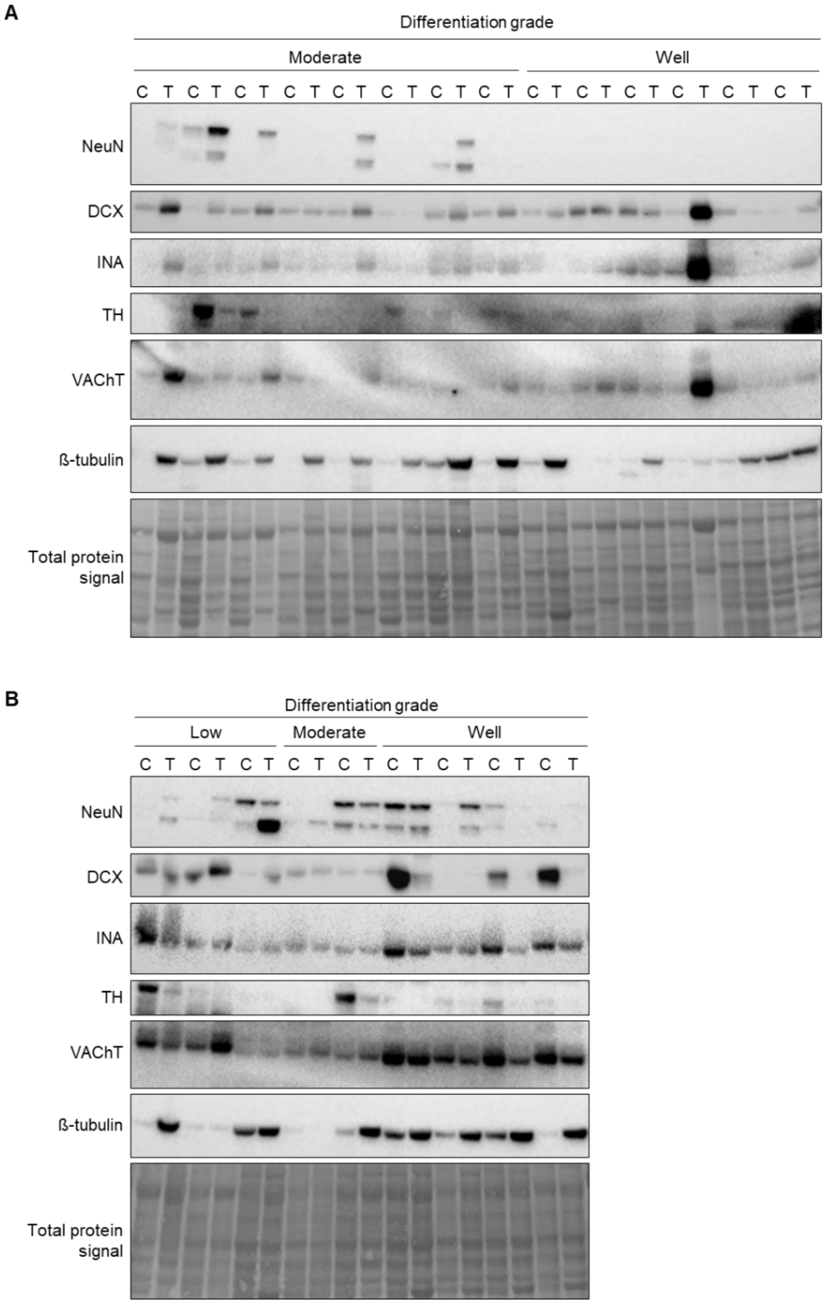


**Fig. S1. Expression of mature and progenitor neural markers in human HCC of HBV or HCV origin.** Immunoblotting using antibodies against NeuN, DCX, INA, TH and VAChT neural markers and β-tubulin as an internal control. Quantification was done after total protein normalization. Results are shown in **Table S3**. (**A**) HBV etiology (n=14 patients). (**B**) HCV etiology (n=9 patients). C, cirrhosis; DCX, doublecortin; INA, internexin neuronal intermediate filament protein alpha; NeuN, neuronal nuclear antigen; T, tumor; TH, tyrosine hydroxylase; VAChT, vesicular acetylcholine transporter (*SLC18A3*).

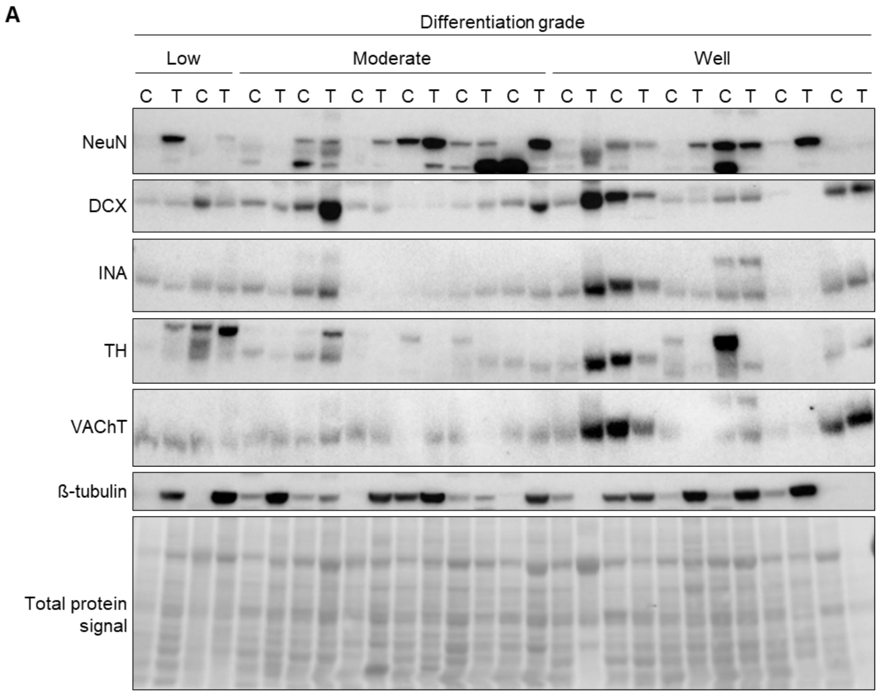


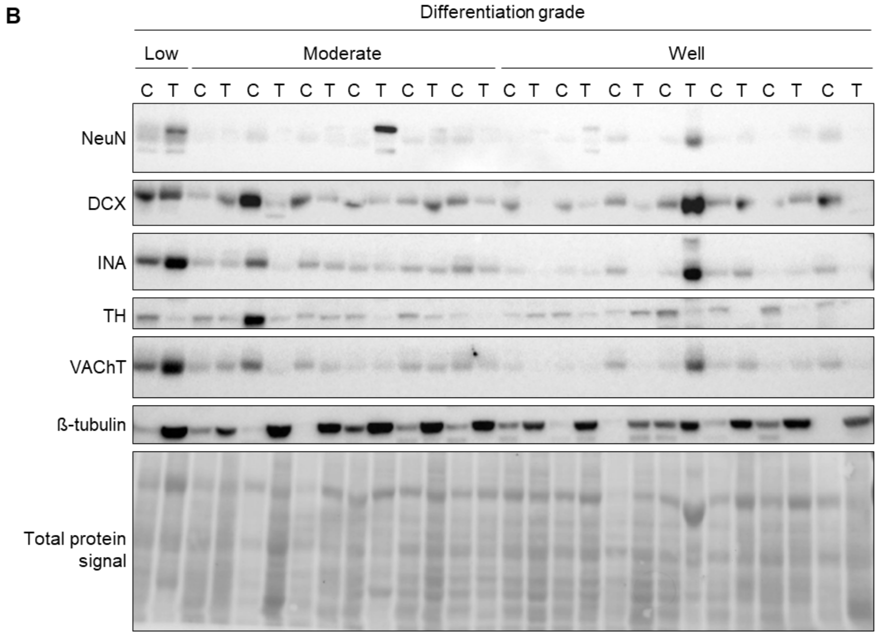


**Fig. S2**. **Expression of mature and progenitor neural markers in human HCC of ALD or NASH origin.** Immunoblotting using antibodies against NeuN, DCX, INA, TH and VAChT neural markers and β-tubulin as an internal control. Quantification was done after total protein normalization. Results are shown in **Table S3**. (**A**) ALD etiology (n=14 patients) (**B**) NASH etiology (n=14 patients). ALD, alcoholic liver disease; C, cirrhosis; DCX, doublecortin; INA, internexin neuronal intermediate filament protein alpha; NASH, non-alcoholic steatohepatitis; NeuN, neuronal nuclear antigen; T, tumor; TH, tyrosine hydroxylase; VAChT, vesicular acetylcholine transporter (*SLC18A3*).

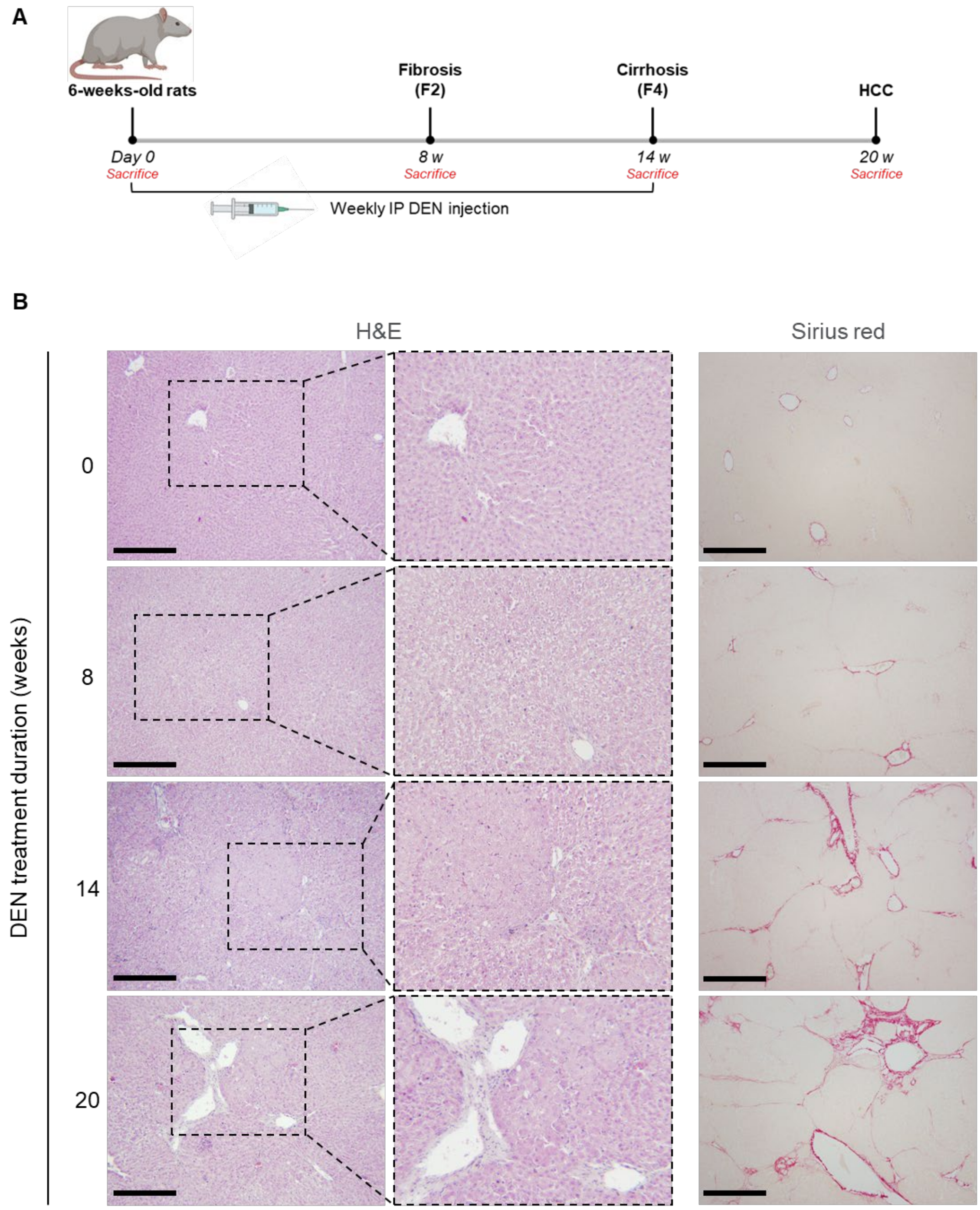


**Fig. S3. Hematoxylin/eosin and Sirius Red staining of CLD and HCC rat samples.** (**A**) Experimental outline. 6-week-old Fischer 334 rats were given DEN at 50 mg/kg weekly from day 0 to week 20, allowing progression of chronic liver disease to fibrosis, cirrhosis, and HCC. Created with BioRender. (**B**) The liver was then fixed, sectioned and stained with H&E or Sirius Red, and visualized under a bright field microscope. Data are representative of 5, 8, 8, and 7 individuals for the following time points: 0, 8, 14 and 20 weeks, respectively. Scale bar: 500 µm. CLD, chronic liver disease; DEN, diethyl-nitrosamine.

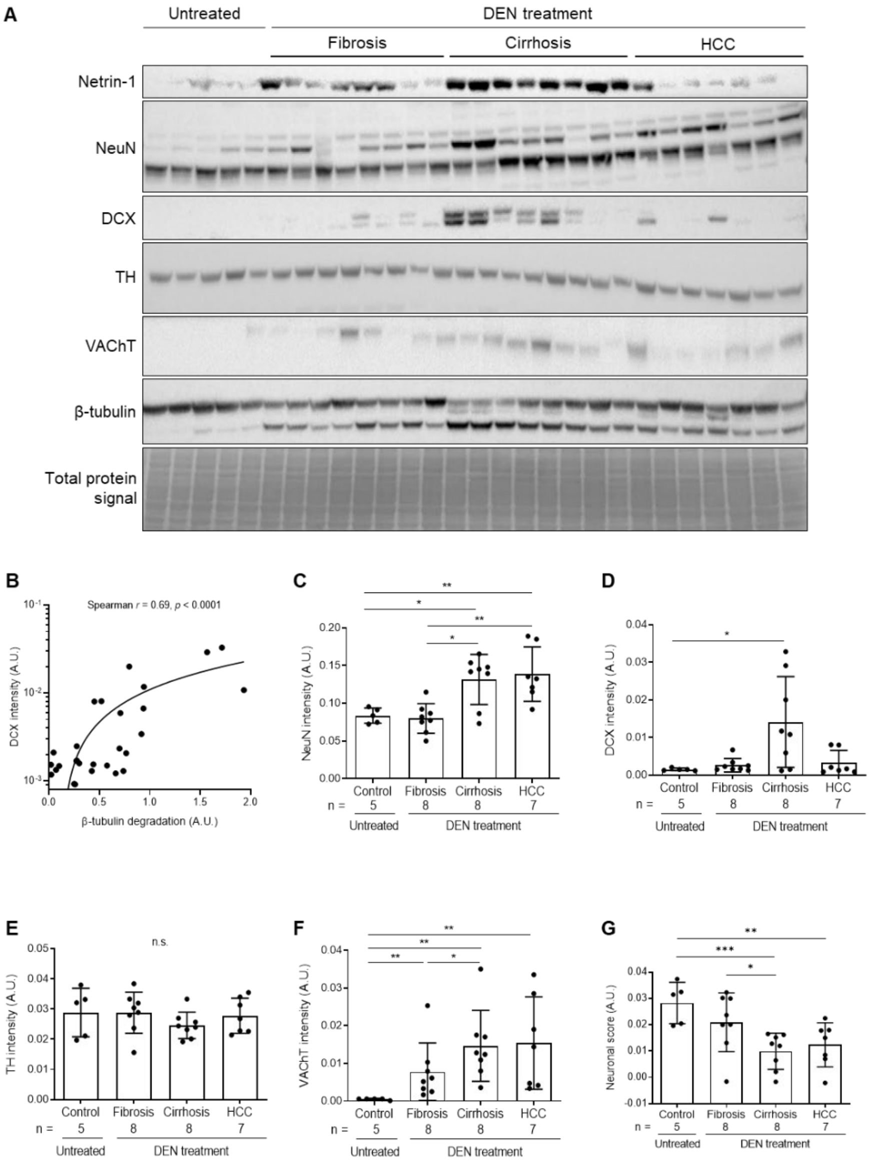


**Fig. S4. Evolution towards HCC and hepatic remodeling are correlated with potential neurogenesis and cholinergic orientation in the cirrhotic rat.** (**A**) Immunoblotting of netrin-1 (neurogenic factor), NeuN (mature neurons), DCX (immature neurons), TH (adrenergic) and VAChT (cholinergic) neural markers. (**B**) DCX induction is correlated with parenchymal remodeling. DCX levels were plotted against ratios of full-length versus degraded β-tubulin signals shown in panel B. Spearman test (*** p<0.001). (**C-G**) Signal quantification was done using the Fiji software on non-saturated images, using total protein normalization prior to statistical plotting using the Mann-Whitney or T-test (after normality test, * p<0.05, ** p<0.01, *** p<0.001). The NS is defined as the difference between TH and VAChT signals. Five to eight rats were used per time point. DCX, doublecortin; DEN, diethyl-nitrosamine; NeuN, neuronal nuclear antigen; TH, tyrosine hydroxylase; VAChT, vesicular acetylcholine transporter (*SLC18A3*).

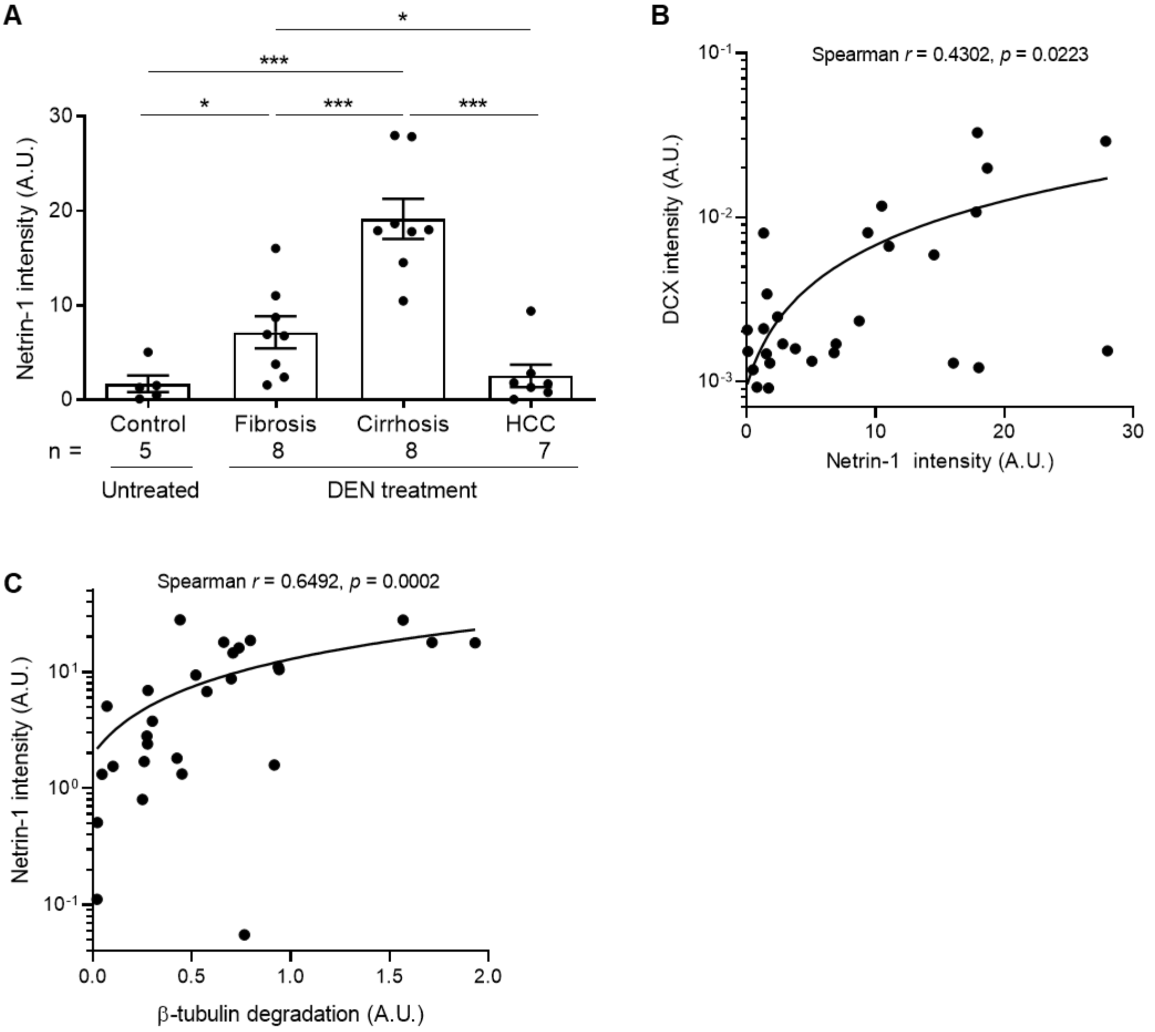


**Fig. S5. Netrin-1 is correlated with neural remodeling, parenchymal remodeling and disease progression in the rat.** (**A**) Netrin-1 signal quantification, Mann-Whitney test or T-test (after normality tests, * p<0.05, *** p<0.001). (**B**) Netrin-1 is correlated with DCX, Spearman test (* p<0.05). (**C**) Netrin-1 levels are correlated with parenchymal remodeling. Netrin-1 levels were plotted against ratios of full-length versus degraded β-tubulin signals blotted in **Fig. S4A**. Spearman test (* p<0.05, *** p<0.001). Quantification was done after total protein normalization. Five to eight identical rats to **Fig. S4** were used per time point. DCX, doublecortin; DEN, diethyl-nitrosamine.

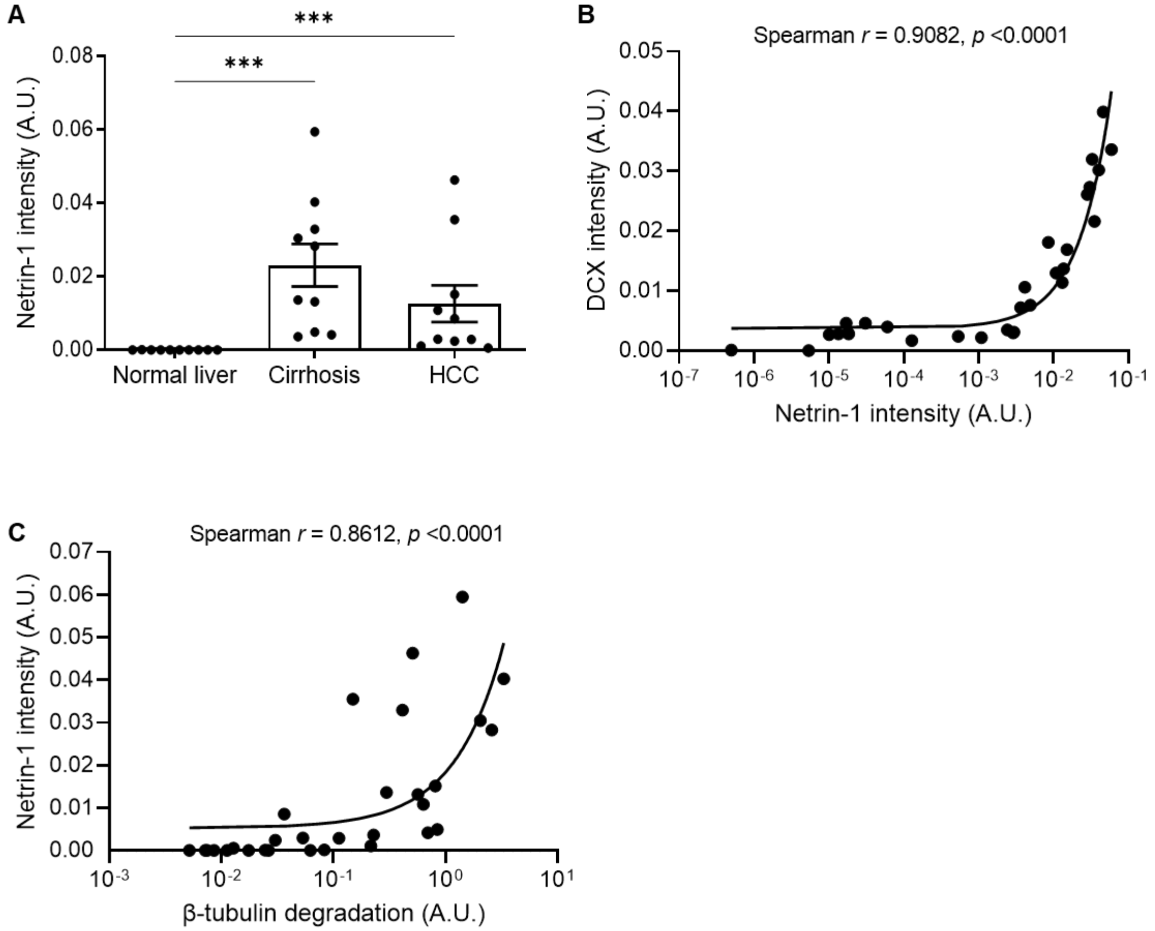


**Fig. S6. Netrin-1 is correlated with neural remodeling, parenchymal remodeling and disease progression in patients.** (**A**) Netrin-1 signal quantification and statistical plotting (Mann-Whitney test after normality test, *** p<0.001). (**B**) Netrin-1 is correlated with DCX, Spearman test (after normality test, *** p<0.001). (**C**) Netrin-1 induction is correlated with parenchymal remodeling. Netrin-1 levels were plotted against ratios of full-length versus degraded β-tubulin signals blotted in **Fig. 1A**, Spearman test (after normality test, *** p<0.001). Quantification was done after total protein normalization. DCX, doublecortin.

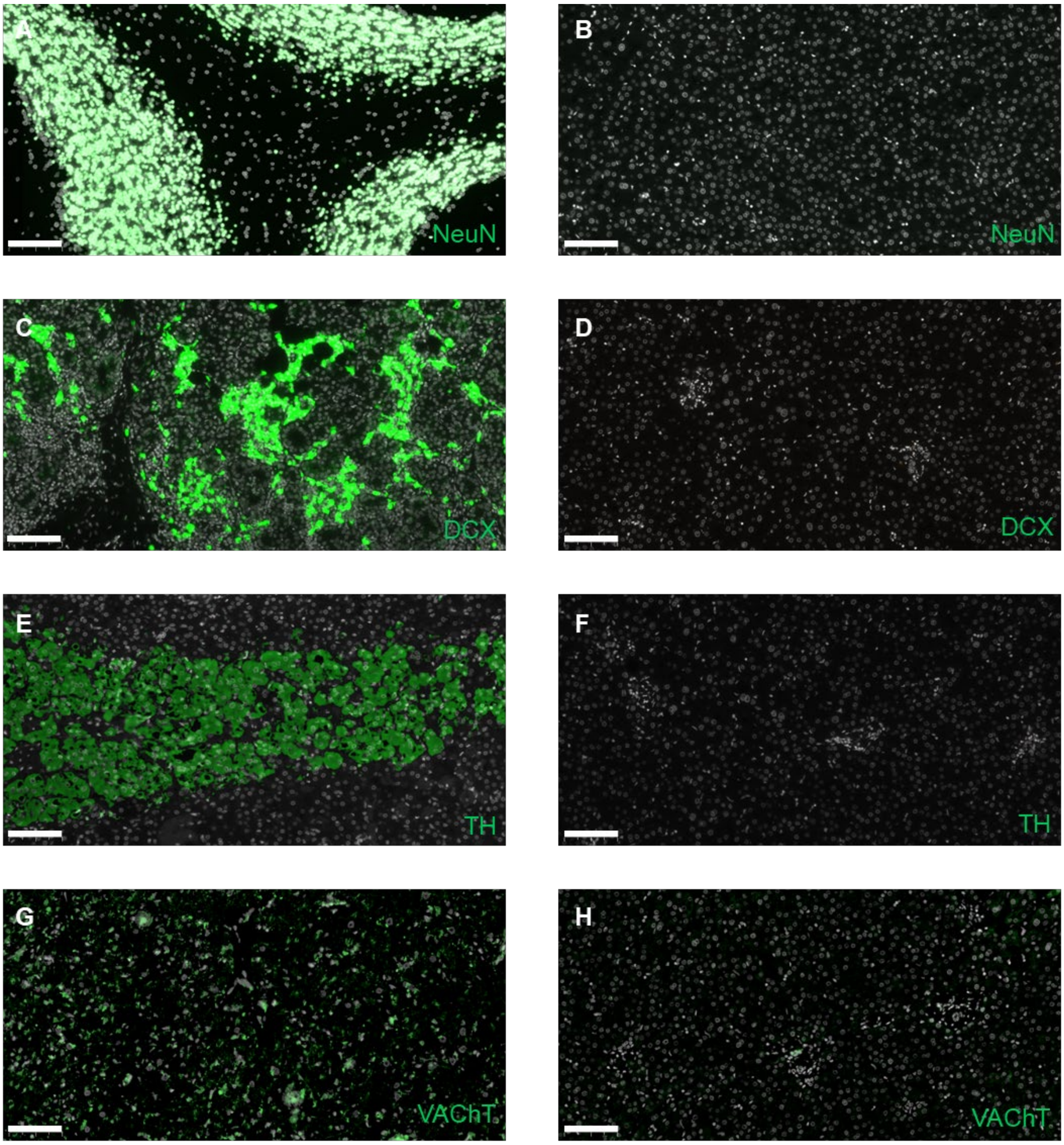


**Fig. S7. Validation of antibodies used by IHC**. Anti-NeuN, DCX, TH and VAChT antibodies were validated by immunofluorescence on *ad hoc* human tissue samples prior to their use on HCC specimens. (**A**) Cerebellum. (**C**) Parotid gland tumor. (**E**) Adrenal gland. (**G**) Cerebral cortex. (**B, D, F, H**) Normal liver (hepatocytic areas). In every panel, nuclei were stained with DAPI (grey signal). Antigens of interest are shown in green. Scale bar: 100 µm. DCX, doublecortin; NeuN, neuronal nuclear antigen; TH, tyrosine hydroxylase; VAChT, vesicular acetylcholine transporter (*SLC18A3*).

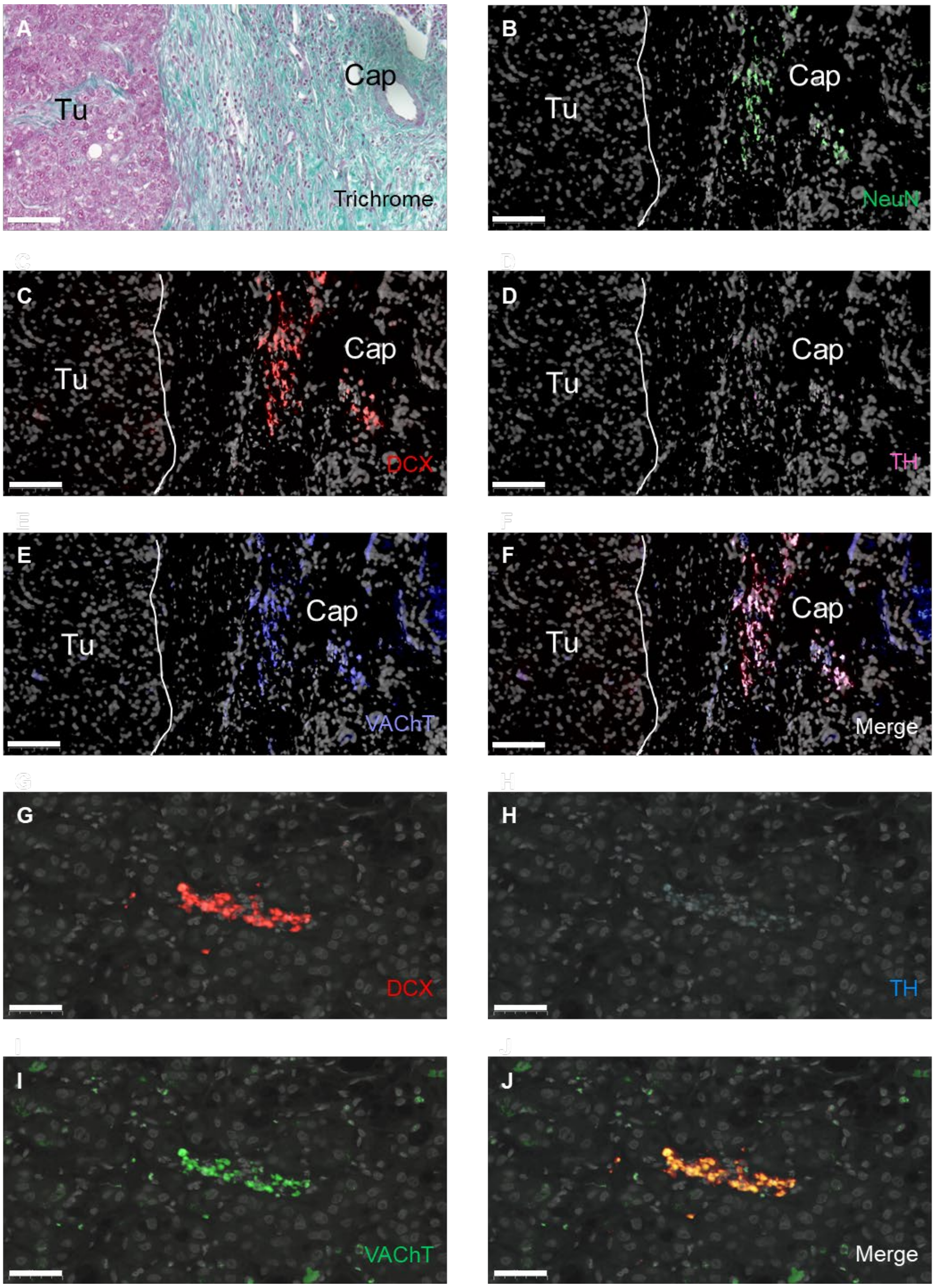


**Fig. S8. Human HCC samples harbor NeuN⁺, DCX⁺, VAChT⁺ nucleated cells (IF approach).** A panel of 24 HCC samples derived from all four main etiologies (HBV: n=7; HCV: n=4; ALD: n=9; NASH: n=4) was probed for immunolocalization of NeuN, DCX, TH and VAChT by multiplex IHC. (**A-F**) Masson's trichrome and IHC staining of a representative capsule-bearing tumor. (**G-J**) Staining of a representative tumor bulk. In every panel, DAPI-stained nuclei appear as grey. Scale bars: 50 µm. ALD, alcoholic liver disease; Cap, capsule; DCX, doublecortin; NASH, non-alcoholic steatohepatitis; NeuN, neuronal nuclear antigen; T, tumor; TH, tyrosine hydroxylase; Tu, tumoral bulk; VAChT, vesicular acetylcholine transporter (*SLC18A3*).

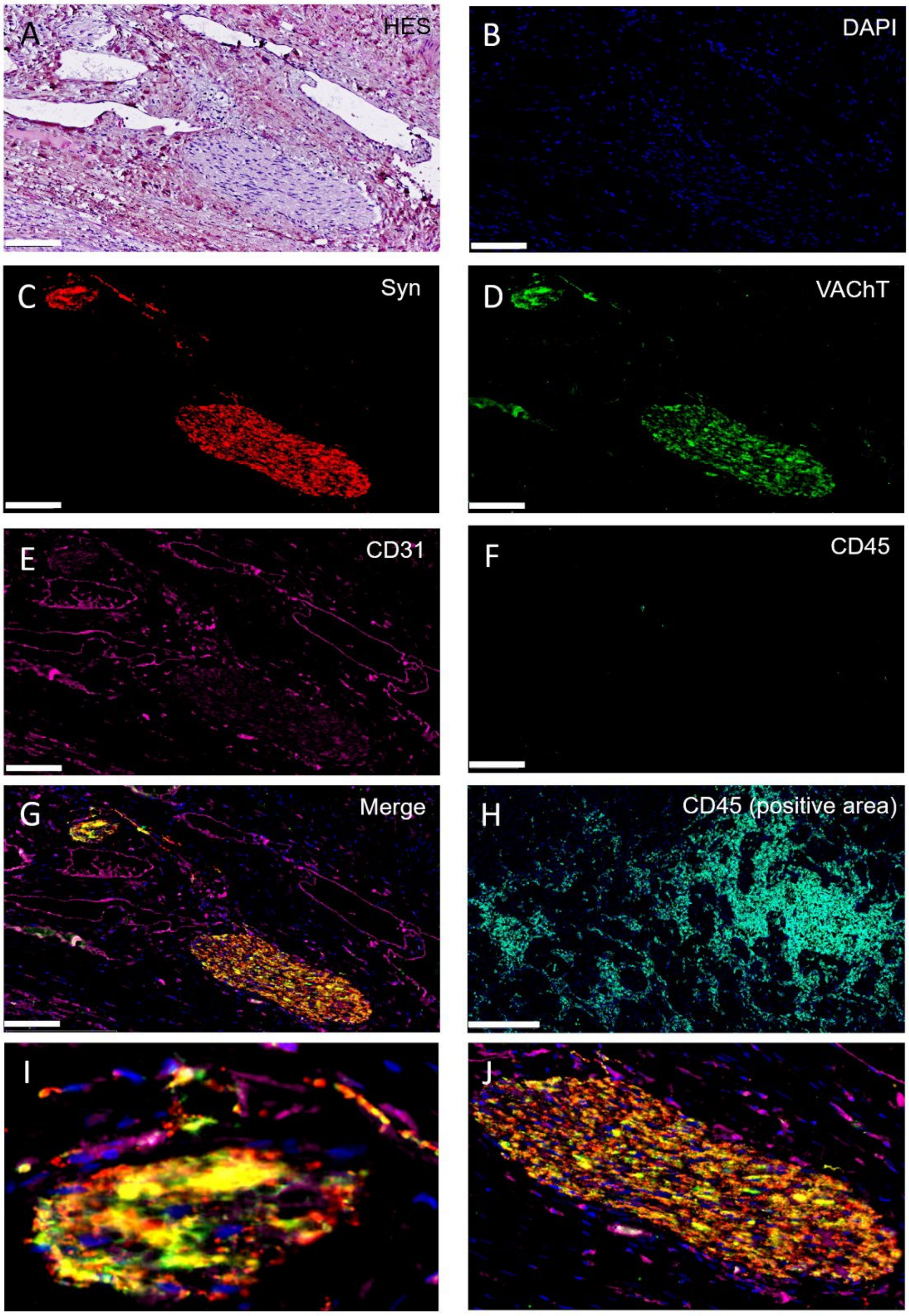


**Fig. S9. Human HCC samples harbor synaptophysin$^+$, VAChT$^+$, CD45$^-$, CD31$^-$ nucleated cells (IF approach).** Samples from the cohort described in **Fig. S8** showing (**A**) HES staining, (**B**) DAPI-stained nuclei, (**C**) Syn, (**D**) VAChT, (**E**) CD31 and (**F**) CD45. (**G**) Merged IF images. (**H**) Anti-CD45 Ab reactivity control sample + DAPI (HBV$^+$ liver tissue from the same cohort). Scale bars: 250 µm. (**I-J**) Focus on both structures of interest shown in (G). Syn, synaptophysin; VAChT, vesicular acetylcholine transporter (*SLC18A3*).

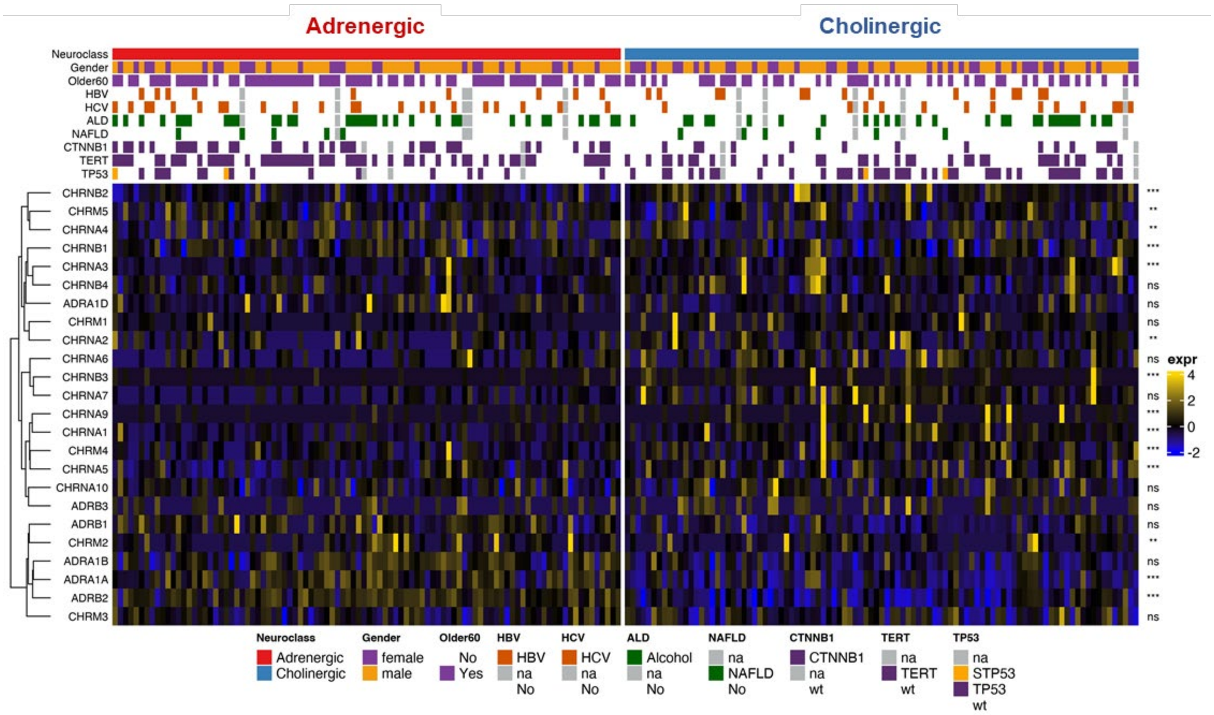


**Fig. S10. Heatmap of relative expression levels of all adrenergic and cholinergic receptors in HCC tumor samples**. Once samples were classified in cholinergic and adrenergic receptor expression classes based on previously obtained signature scores, differential expression analysis was performed and expression values normalized (variance stabilization transformation) and shown in the heatmap. T-test, ** p<0.01, *** p<0.001. Older60, patients older than 60; ALD, alcoholic liver disease; NAFLD, non-alcoholic fatty liver disease; Mutations: TP53, CTNNB1, TERT; Wt, wild type; Na, no information available.

**A**

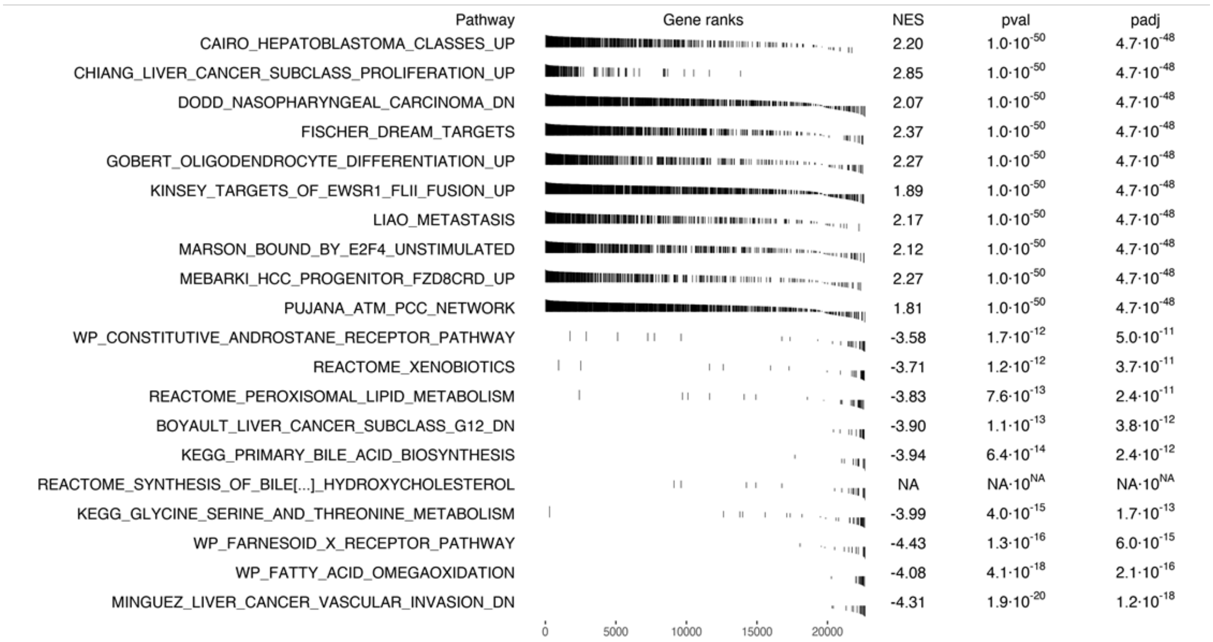


**B**

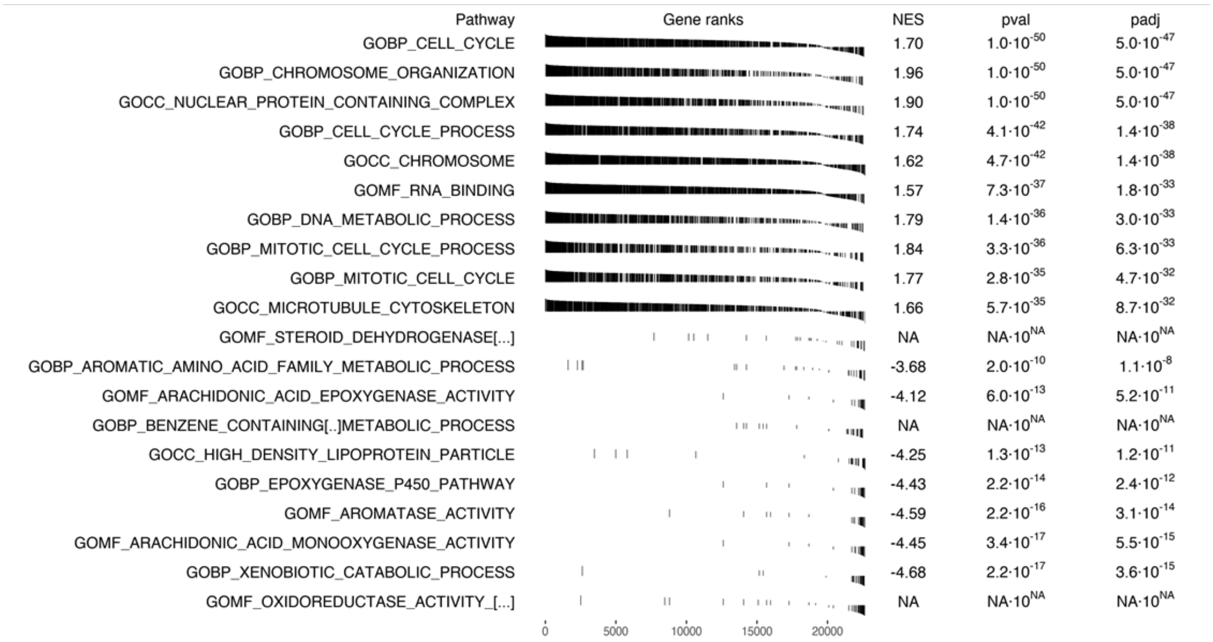


**Fig. S11. Confirmation of pathway analysis results found on the Hallmark collection dataset by gene set enrichment analyses on the C2 and C5 gene sets of the MSigDB.** (**A**) Adrenergic and (**B**) cholinergic samples.

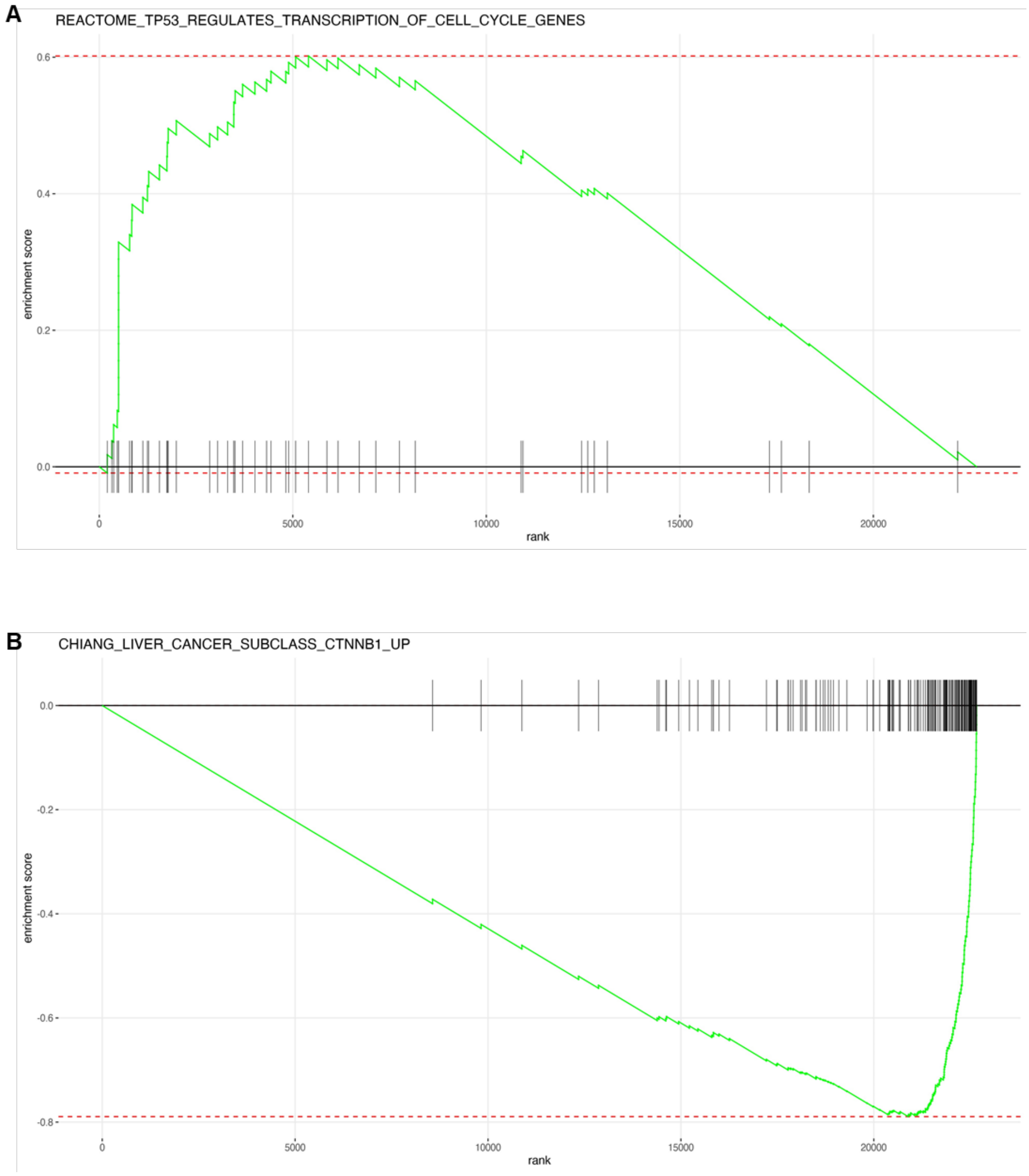


**Fig. S12. Example of (A) enrichment in *TP53* mutations and of (B) depletion in *CTNNB1* mutation-associated metabolic functions in the cholinergic samples.**

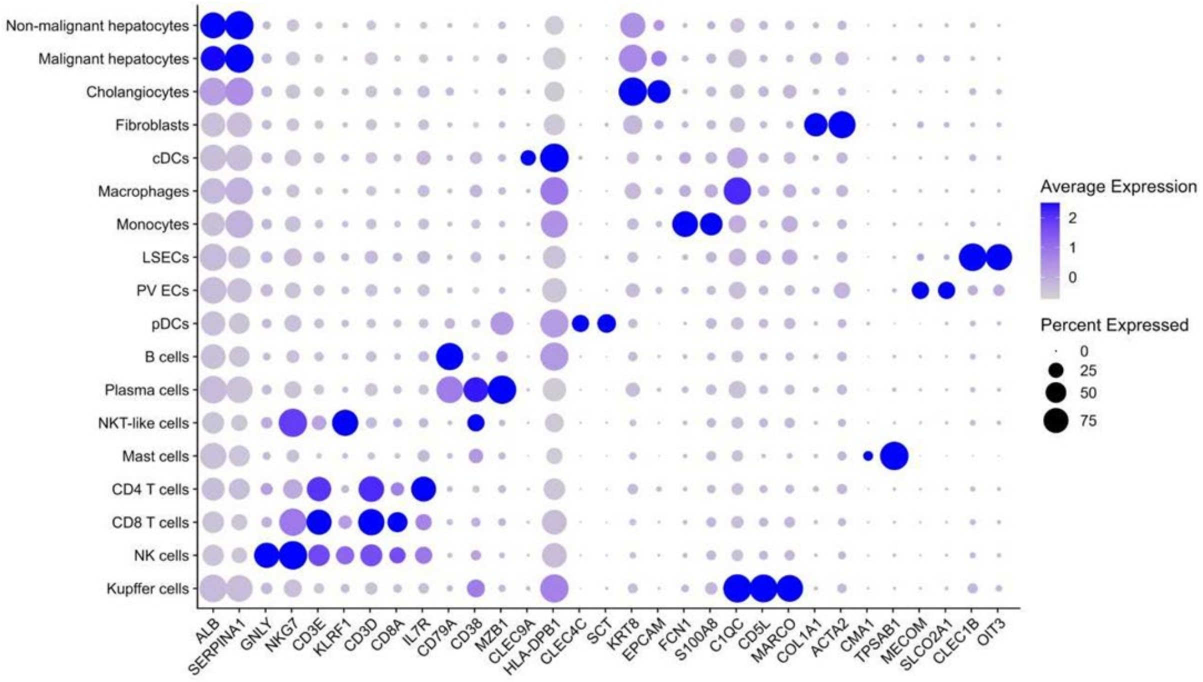


**Fig. S13. Identification of hepatic cell types in HCC samples.** Average expression levels and percentage of cells expressing canonical cell type markers in each of the 18 cell types identified. Data obtained from GSE149614 (n=10). cDCs, classical dendritic cells; NK, natural killer cells; LSECs, liver sinusoidal endothelial cells; PVECs, portal vein endothelial cells; pDCs, plasmacytoid dendritic cells.

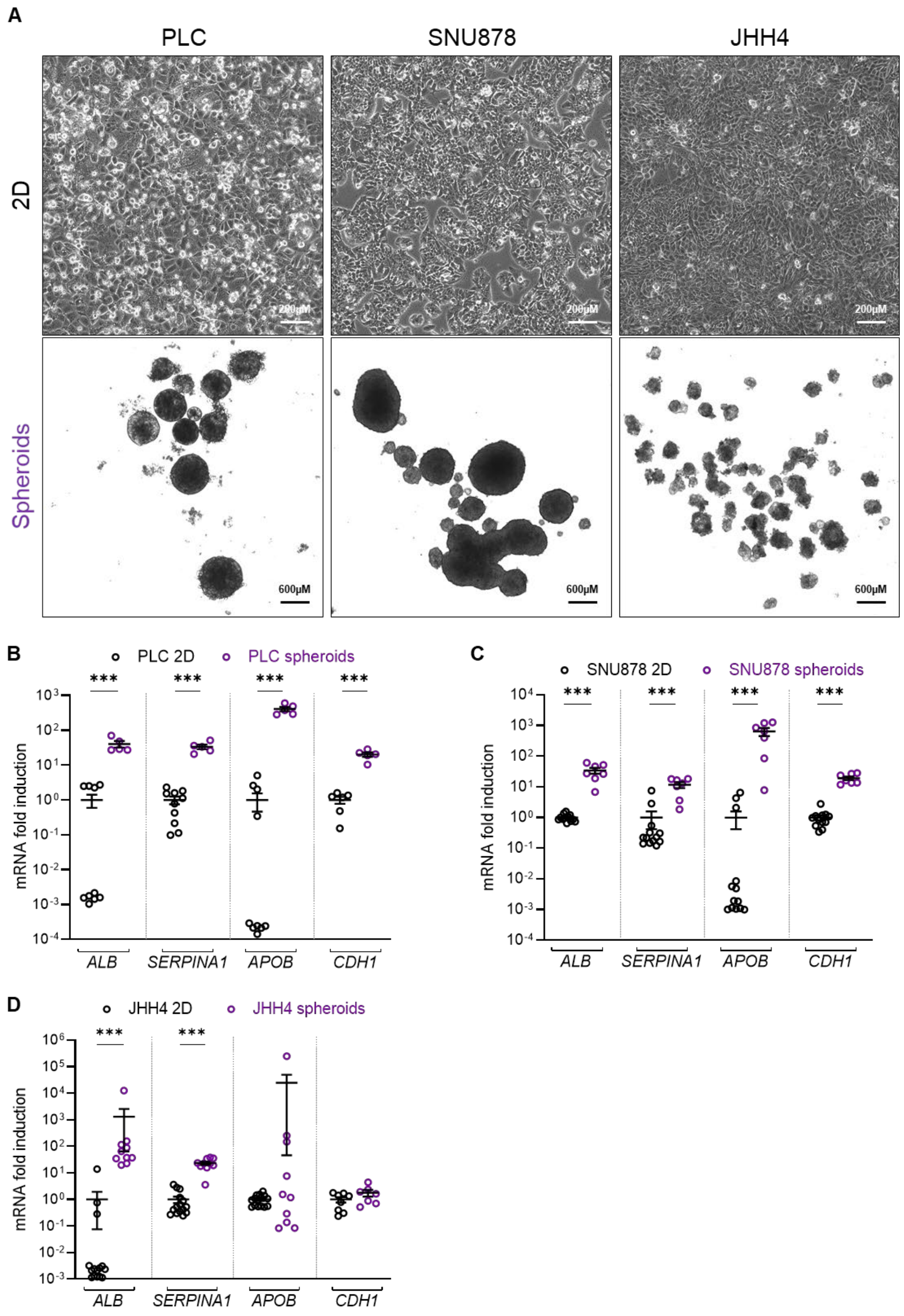


**Fig. S14. Experimental validation of generated spheroids.** (**A**) Corresponding micrographs of spheroids grown in the conditions described in Expanded materials. (**B-D**) Re-induction of HNF4α-controlled hepatocytic differentiation-related transcripts upon spheroid formation (n≥5 independent experiments for each cell line, Mann-Whitney test after normality test, *** $p<0.001$). ALB, albumin; APOB, apolipoprotein B; CDH1, cadherin 1; HNF4α, hepatocyte nuclear factor 4 alpha; SERPINA1, serpin family A member 1.

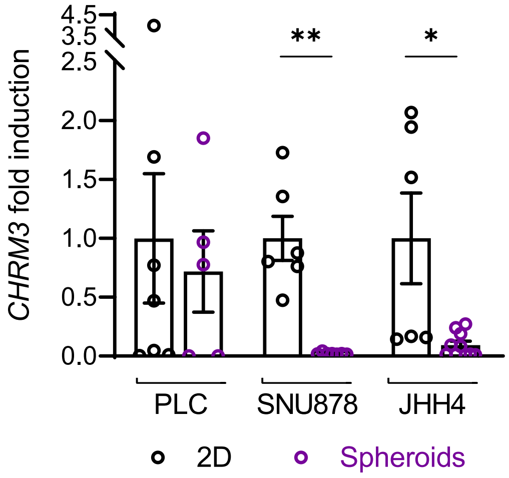


**Fig. S15. Experimental re-differentiation represses *CHRM3* expression.** PLC, SNU878, and JHH4 spheroids were analyzed by RT-qPCR. Mann-Whitney test or T-test (depending on normality, * $p<0.05$, ** $p<0.01$), ($n\geq5$ independent experiments for each cell line). CHRM3, cholinergic receptor muscarinic 3.

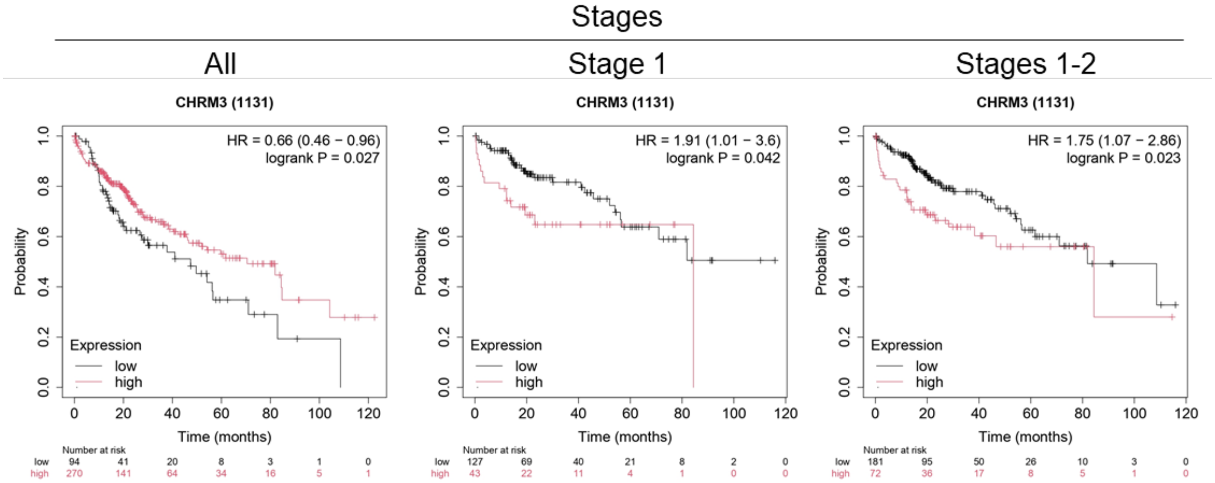


**Fig. S16. High *CHRM3* predicts shorter survival in stages 1-2 HCC.** Log-Rank test and Kaplan-Meier plot on the association between *CHRM3* expression (defined as below and above median) and survival. Left panel: all TCGA LIHC patients considered. Center panel: data derived from Stage 1 patients. Right panel: data derived from Stage 1-2 patients. Log-rank $p<0.05$.

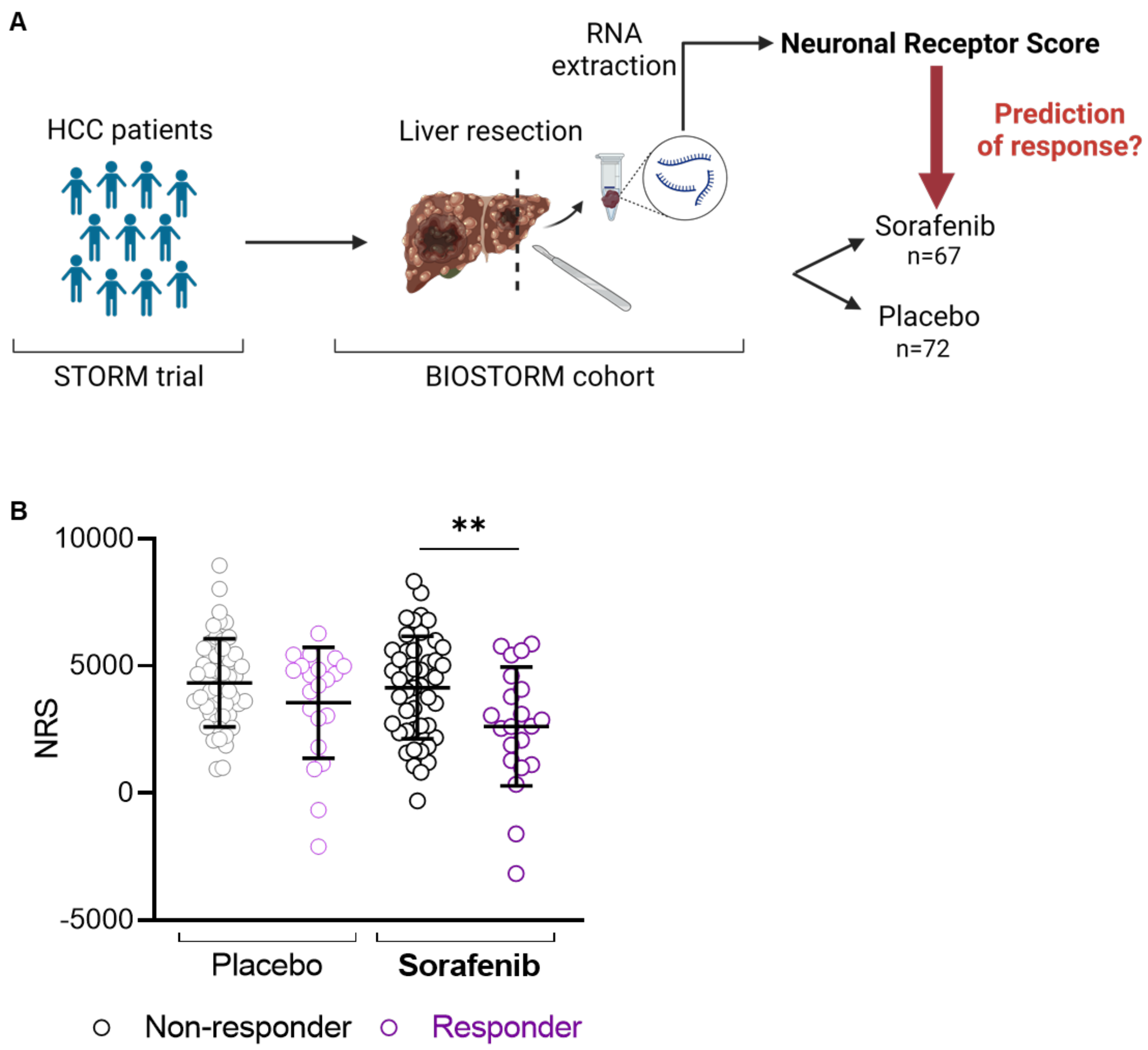


**Fig. S17. Cholinergic orientation (low NRS) predicts response to sorafenib in the STORM (BIOSTORM cohort) trial.** (**A**) Description of the experimental approach. Created with BioRender. (**B**) Comparison of NRS in tumor tissues from placebo non-responders to treatment (n=51), placebo responders (n=21), sorafenib non-responders (n=46) and sorafenib responders (n=21). Groups were defined in the original study based on objective response criteria [15]. Kruskal-Wallis test (GSE109211) after normality test (** $p<0.01$). Bars represent mean ± SD. NRS, neuronal receptor score.

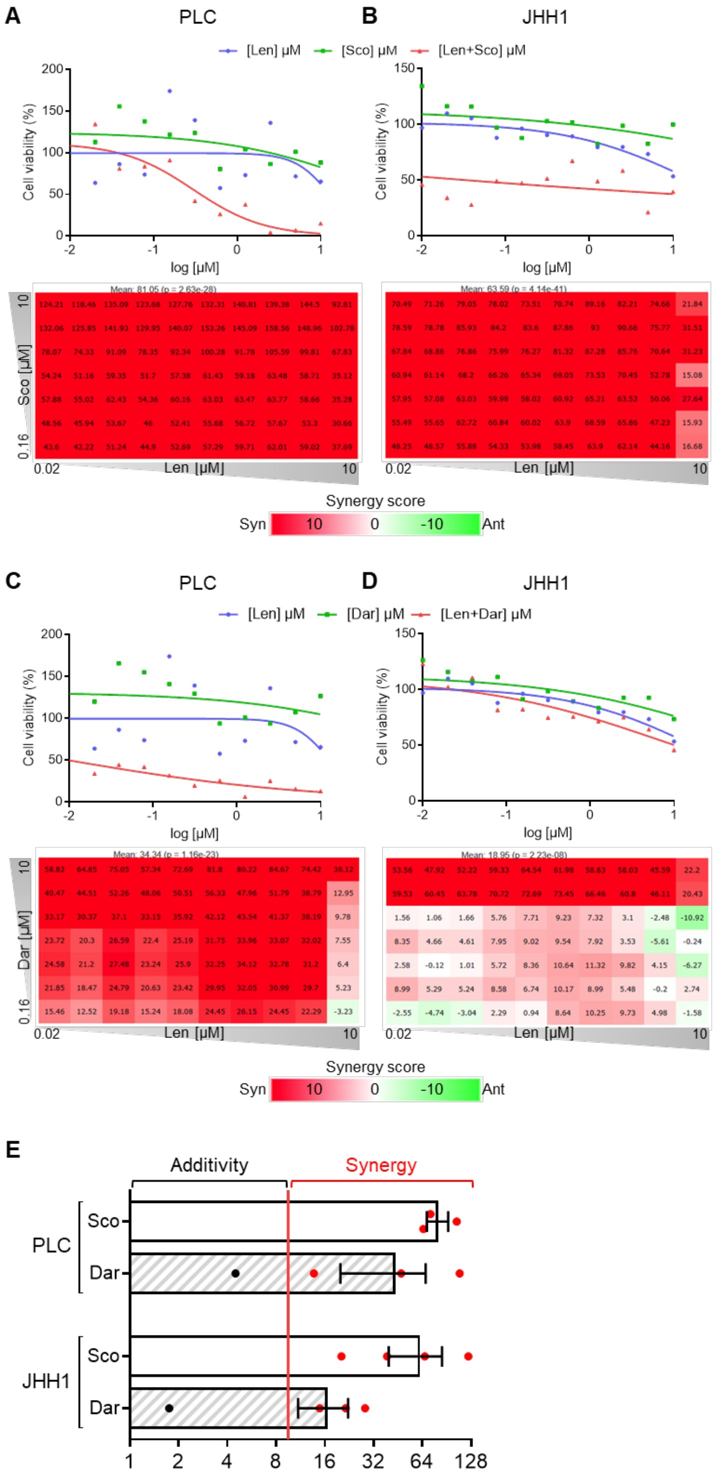


**Fig. S18. Muscarinic blockade synergizes with the standard-of-care TKI lenvatinib.** (**A-D**) Sigmoid activity curves and 2D matrices on (**A-B**) scopolamine and (**C-D**) darifenacin, respectively on PLC (class 1) and JHH1 (class 3) HCC lines. Chou-Talalay ZIP scores >10 indicate synergy. (**E**) Plate-wide average ZIP score values calculated from two separate 96-well matrices for each experiment. Sigmoids are representative of n=3-5 independent experiments. Dar, darifenacin; Len, lenvatinib; Sco, scopolamine.

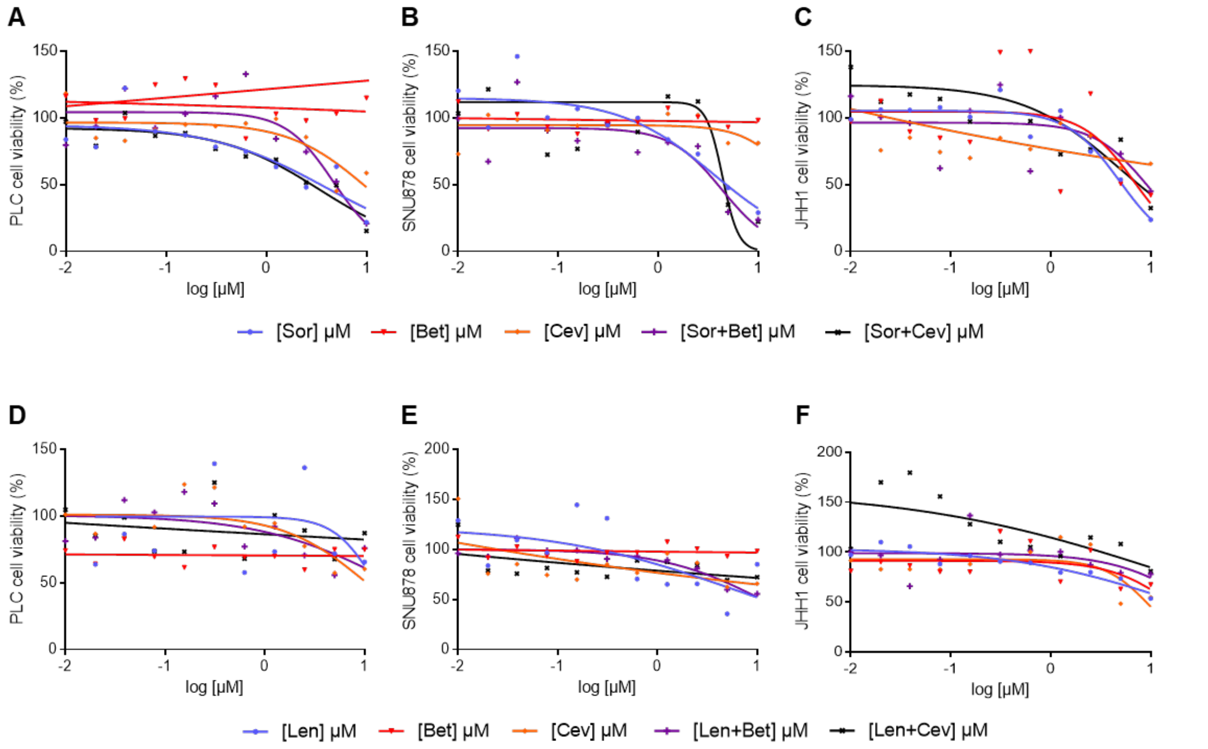


**Fig. S19. Muscarinic agonists bethanechol and cevimeline do not affect TKI activities.** (**A-F**) Activity curves of sorafenib (**A-C**) and lenvatinib (**D-F**), respectively on PLC (class 1), SNU878 (class 2) and JHH1 (class 3) treated HCC lines. Sigmoids are representative of n=3 independent experiments. Cev, cevimeline; Len, lenvatinib; Sor, sorafenib; TKI, tyrosine kinase inhibitor.

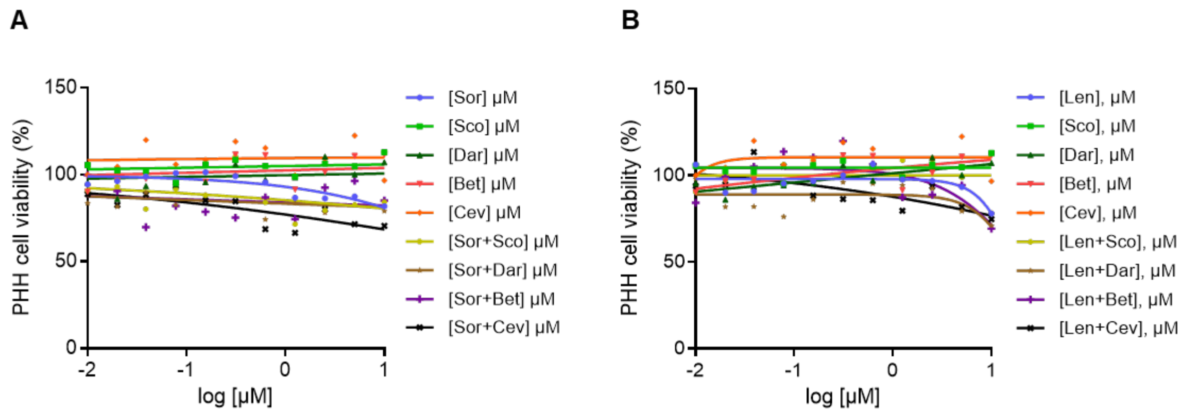


**Fig. S20. Muscarinic targeting does not affect viability of PHH with or without TKI combination**. (**A-B**) Activity curves of sorafenib and lenvatinib, respectively. n=3 independent experiments carried out on PHH, each derived from one distinct patient. Cev, cevimeline; Dar, darifenacin; Len, lenvatinib; PHH, primary human hepatocytes; Sco, scopolamine; Sor, sorafenib; TKI, tyrosine kinase inhibitor.

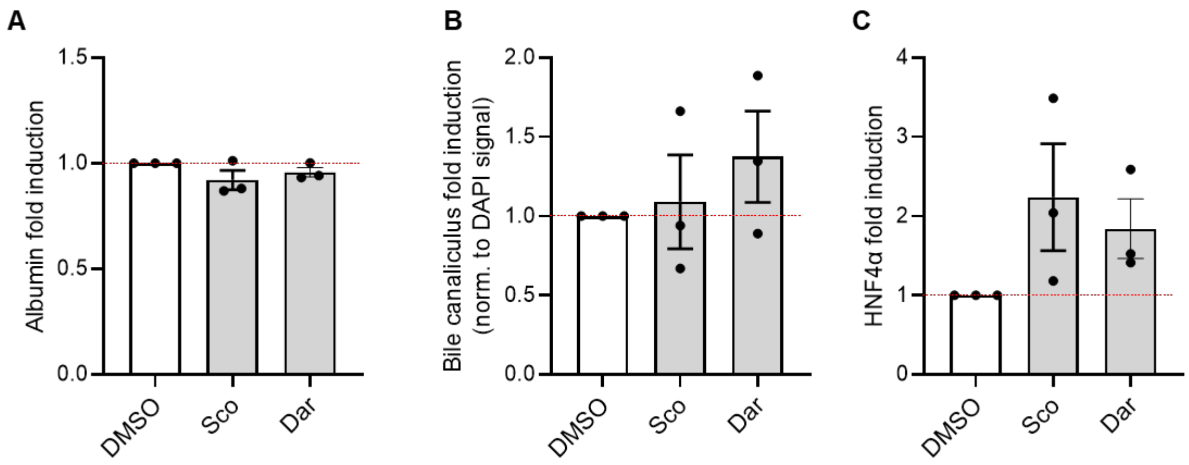


**Fig. S21. Muscarinic targeting preserves mature hepatocytic functions.** (**A**) Secreted human albumin quantified by Elisa. (**B**) Bile canaliculus density alterations normalized against DAPI signal. (**C**) Changes in HNF4α DNA binding activity. T-test after normality test. n.s. Cells underwent 28 days of differentiation. n=3 independent experiments. Dar, darifenacin; HNF4α, hepatocyte nuclear factor 4 alpha; Sco, scopolamine.

## SUPPLEMENTARY TABLES

**Table S1. Characteristics of paired F4/HCC samples used in the study.** Samples were obtained from the French liver biobank network. Median ± SD is shown for all qualitative data.

| Variable | Available data (Total: 166) | Values |
|---|---|---|
| **Demographics** | 166 | |
| Age, *y* ± SD | 166 | 69 ± 10 |
| Gender (male/female) | 166 | 140 (84%)/26 (16%) |
| **Etiology (%)** | 166 | |
| Alcoholic liver disease | | 36 (21%) |
| Hepatitis B virus | | 46 (28%) |
| Hepatitis C virus | | 39 (24%) |
| Non-alcoholic steato-hepatitis | | 45 (27%) |
| **Liver disease parameters** | | |
| Liver fibrosis score METAVIR (F4) (%) | 166 | 166 (100%) |
| Inflammation activity score (%) | 137 | |
| METAVIR 0 | | 51 (37%) |
| METAVIR 1 | | 55 (40%) |
| METAVIR 2 | | 23 (17%) |
| METAVIR 3 | | 8 (6%) |
| Serum alpha-fetoprotein, >100 ng/mL | 111 | 17 (16%) |
| Child-Pugh score (A/B/C) (%) | 87 | 72 (83%)/9 (11%)/6 (6%) |
| Prothrombin, % ± SD | 146 | 81 ± 22 |
| Bilirubin, µMol/L ± SD | 137 | 14 ± 75 |
| Albumin, g/L ± SD | 100 | 36 ± 7 |
| Platelet count, G/L ± SD | 149 | 131 ± 102 |
| Encephalopathy (%) | 164 | 18 (11%) |
| Ascites (%) | 164 | 37 (3%) |
| Jaundice (%) | 162 | 22 (14%) |
| Esophageal varices (%) | 156 | 62 (40%) |
| **Tumors characteristics** | | |
| Tumor size, *mm* ± SD | 165 | 34 ± 31 |
| Tumor localization (left liver/right liver/double) | 165 | 47 (29%)/112 (68%)/6 (4%) |
| Intact tumor capsule (%) | 143 | 91 (64%) |
| Satellite nodules (%) | 165 | 34 (21%) |
| Macrovascular invasion, Microvascular invasion (%) | 151 | 22 (15%), 81 (52%) |
| Differentiation grade (poor/moderately/well) (%) | 165 | 15 (9%)/74 (45%)/76 (46%) |
| Architectural pattern (%) | 136 | |
| Trabecular | | 104 (76%) |
| Pseudoglandular | | 10 (7%) |
| Compact | | 5 (4%) |
| Clear cells | | 4 (3%) |
| Others | | 13 (9%) |

| | | |
|---|---|---|
| Tumoral steatosis (%) | 165 | 108 (66%) |
| Tumoral necrosis (%) | 150 | 84 (56%) |

**Table S2. Significance assessment of neural features in HCC.** Wilcoxon matched-pairs signed rank test was used (* p<0.05, ** p<0.01).

| Evolution of values from cirrhosis to HCC (paired samples) | | | | | | |
|---|---|---|---|---|---|---|
| | DCX | NEUN | INA | TH | VAChT | **Neuronal score (TH-VACHT)** |
| HBV (n=14) | ↗* | ↗* | | | | |
| HCV (n=9) | | | | ↘** | | |
| ALCOHOL (n=14) | | | | | | |
| NASH (n=14) | | | | ↘** | | |
| **All etiologies (n=51)** | | ↗* | | ↘** | | ↘** |

**Table S3 (Excel file). List of adrenergic and cholinergic receptors expressed in TCGA HCC samples.** Their differential gene expression grouped by neural class was analyzed by the DESeq2 method.

**Table S4 (Excel file). Association levels between neural classes and major HCC-related clinico-biological variables.** Fisher test was used; TCGA cohort.

**Table S5. Association levels between neural classes and major HCC-related clinico-biological variables.** Fisher test was used; validation cohort.

| **Variable 1** | **p-value** |
|---|---|
| Gender | 0.1628 |
| Older60 | 0.3646 |
| HBV | 0.6338 |
| HBV / HDV | 0.8315 |
| HCV | 0.6389 |
| ALD | 0.3697 |
| CTNNB1 | 0.0562 |
| TP53 | 0.5948 |

**Table S6 (Excel file). List of the 100 genes most associated with the adrenergic class.** Log2foldchange: alteration levels considering the cholinergic class as a reference. Lfcse: Log2foldchange of standard error. padj: adjusted p-value.

**Table S7 (Excel file). List of the 100 genes most associated with the cholinergic class.** Log2foldchange: alteration levels considering the adrenergic class as a reference. Lfcse: Log2foldchange of standard error. padj: adjusted p-value.

**Table S8. Main differentially expressed genes of interest in adrenergic and cholinergic TCGA LIHC samples.**

| | **Gene symbol** | **Log2 fold change** | **Function** | **PMID** |
|---|---|---|---|---|
| Up-regulated in the cholinergic class | *LGALS14* | 8 | T-cell apoptosis related antigen | 12678492 |
| | *CT55* | 6 | Cancer testis antigen | 35189384 |
| | *BMP7* | 6 | TGF-β family growth factor | 12808448 |
| | *CEACAM7* | 5 | Carcinoembryonic antigen-related cell adhesion molecule 7 | 22195770 |
| | *MAGEA4, MAGEA10* | 4 | HCC progression drivers | 34166362 |
| | *XAGE2* | 4 | Fetal/reproductive tissue tumor-related antigen | 11992404 |
| | *GAGE2A* | 3 | Germ-cell and tumor antigen | 23029259 |
| | *AFP* | 4 | HCC diagnosis-related antigen | 32923383 |
| Up-regulated in the adrenergic class | *ALDH3A1* | 3 | Xenobiotic-inducible hepatocytic differentiation markers | 27279633 |
| | *CYP1A1, 3A4, 1A2* <br> *CYP2A13, 2A7P1, 3F36P* | 2 to 3 | | 20645049 |

**Table S9 (Excel file). Pathways enriched in the adrenergic class.** Over Representation Analysis with hypergeometric test. $p<0.01$. Besides signature names listed in this table ($p<0.01$ Wilcoxon test), attention needs to be paid to their expanded biological significance extracted from the ssGSEA database before drawing conclusions with respect to pathology.

**Table S10 (Excel file). Pathways enriched in the cholinergic class.** Over Representation Analysis with hypergeometric test. $p<0.01$. Besides signature names listed in this table ($p<0.01$ Wilcoxon test), attention needs to be paid to their expanded biological significance extracted from the ssGSEA database before drawing conclusions with respect to pathology.

**Table S11. Biological descriptions of prognosis pathways associated with both HCC neuroclasses.** Besides signature names listed in this table, attention needs to be paid to their expanded biological significance extracted from the ssGSEA database before drawing conclusions with respect to pathology.

| **Standard name** | **Brief description** | **Systematic name** |
|---|---|---|
| ANDERSEN_LIVER_CANCER_KRT19_DN | Genes under-expressed in KRT19-positive [GeneID=3880] hepatocellular carcinoma. | M424 |
| BOYAULT_LIVER_CANCER_SUBCLASS_G1_DN | Down-regulated genes in hepatocellular carcinoma (HCC) subclass G1, defined by unsupervised clustering | M1883 |
| BOYAULT_LIVER_CANCER_SUBCLASS_G12_DN | Down-regulated genes in hepatocellular carcinoma (HCC) subclass G12, defined by unsupervised clustering | M12228 |
| BOYAULT_LIVER_CANCER_SUBCLASS_G123_DN | Down-regulated genes in hepatocellular carcinoma (HCC) subclass G123, defined by unsupervised clustering. | M2218 |
| BOYAULT_LIVER_CANCER_SUBCLASS_G6_UP | Up-regulated genes in hepatocellular carcinoma (HCC) subclass G6, defined by unsupervised clustering. | M4342 |
| CHIANG_LIVER_CANCER_SUBCLASS_CTNNB1_DN | Top 200 marker genes down-regulated in the 'CTNNB1' subclass of hepatocellular carcinoma (HCC); characterized by activated CTNNB1 [GeneID=1499]. | M8689 |
| CHIANG_LIVER_CANCER_SUBCLASS_CTNNB1_UP | Top 200 marker genes up-regulated in the 'CTNNB1' subclass of hepatocellular carcinoma (HCC); characterized by activated CTNNB1 [GeneID=1499]. | M16496 |
| CHIANG_LIVER_CANCER_SUBCLASS_INTERFERON_DN | All marker genes down-regulated in the 'interferon' subclass of hepatocellular carcinoma (HCC). | M14353 |

| | | |
|---|---|---|
| CHIANG_LIVER_CANCER_SUBCLASS_POLYSOMY7_UP | Marker genes up-regulated in the 'chromosome 7 polysomy' subclass of hepatocellular carcinoma (HCC); characterized by polysomy of chromosome 7 and by a lack of gains of chromosome 8q. | M834 |
| CHIANG_LIVER_CANCER_SUBCLASS_PROLIFERATION_DN | Top 200 marker genes down-regulated in the 'proliferation' subclass of hepatocellular carcinoma (HCC); characterized by increased proliferation, high levels of serum AFP [GeneID=174], and chromosomal instability. | M16932 |
| CHIANG_LIVER_CANCER_SUBCLASS_PROLIFERATION_UP | Top 200 marker genes up-regulated in the 'proliferation' subclass of hepatocellular carcinoma (HCC); characterized by increased proliferation, high levels of serum AFP [GeneID=174], and chromosomal instability. | M3268 |
| DESERT_PERIPORTAL_HEPATOCELLULAR_CARCINOMA_SUBCLASS_UP | Genes up-regulated in the periportal-type subclass of hepatocellular carcinomas. | M34031 |
| DESERT_STEM_CELL_HEPATOCELLULAR_CARCINOMA_SUBCLASS_UP | Genes up-regulated in the stem cell-type subclass of hepatocellular carcinomas. | M34034 |
| HOSHIDA_LIVER_CANCER_LATE_RECURRENCE_DN | Genes whose expression correlated with lower risk of late recurrence of hepatocellular carcinoma (HCC). | M13658 |
| HOSHIDA_LIVER_CANCER_SUBCLASS_S1 | Genes from 'subtype S1' signature of hepatocellular carcinoma (HCC): aberrant activation of the WNT signaling pathway. | M5311 |
| HOSHIDA_LIVER_CANCER_SUBCLASS_S3 | Genes from 'subtype S3' signature of hepatocellular carcinoma (HCC): hepatocyte differentiation. | M1286 |
| HOSHIDA_LIVER_CANCER_SURVIVAL_DN | Survival signature genes defined in adjacent liver tissue: genes correlated with good survival of hepatocellular carcinoma (HCC) patients. | M5451 |
| HOSHIDA_LIVER_CANCER_SURVIVAL_UP | Survival signature genes defined in adjacent liver tissue: genes correlated with poor survival of hepatocellular carcinoma (HCC) patients. | M6939 |
| KIM_LIVER_CANCER_POOR_SURVIVAL_DN | Genes under-expressed in hepatocellular carcinoma (HCC) with poor survival | M534 |
| LEE_LIVER_CANCER_SURVIVAL_DN | Genes highly expressed in hepatocellular carcinoma with worse survival. | M7987 |
| LEE_LIVER_CANCER_SURVIVAL_UP | Genes highly expressed in hepatocellular carcinoma with better survival. | M6145 |
| VILLANUEVA_LIVER_CANCER_KRT19_DN | Genes under-expressed in KRT19-positive [GeneID=3880] hepatocellular carcinoma (HCC). | M373 |

| | | |
|---|---|---|
| VILLANUEVA_LIVER_CANCER_KRT19_UP | Genes over-expressed in KRT19-positive [GeneID=3880] hepatocellular carcinoma (HCC). | M336 |
| WOO_LIVER CANCER RECURRENCE_DN | Genes negatively correlated with recurrence free survival in patients with hepatitis B-related (HBV) hepatocellular carcinoma (HCC). | M9911 |
| WOO_LIVER_CANCER_RECURRENCE_UP | Genes positively correlated with recurrence free survival in patients with hepatitis B-related (HBV) hepatocellular carcinoma (HCC). | M12602 |
| YAMASHITA_LIVER_CANCER_STEM_CELL_DN | Genes down-regulated in hepatocellular carcinoma (HCC) cells with hepatic stem cell properties. | M9206 |

**Table S12 (Excel file). Association of adrenergic and cholinergic tumors with canonical HCC signatures.** Besides signature names listed in this table, attention needs to be paid to their expanded biological significance extracted from the ssGSEA database before drawing conclusions with respect to pathology (padj<0.001 Fischer test).

**Table S13. Suitability of HCC lines for the approaches used in this study.** Classes refer to the 2019 Caruso transcriptomic classification of HCC lines.

| | **Subgroups** | | | | |
|---|---|---|---|---|---|
| | **Cl. 1** | **Cl. 2** | | **Cl. 3** | |
| | **PLC** | **SNU878** | **HepaRG** | **JHH1** | **JHH4** |
| **Spheroids** | suitable | suitable | suitable | no growth | suitable |
| **Soft Agar** | suitable | suitable | suitable | no growth | no growth |
| **Anoikis** | suitable | suitable | suitable | no growth | suitable |

**SUPPLEMENTAL INFORMATION 1**

**Validation of anti-NeuN, DCX, INA, TH and VAChT antibodies by Western Blot.**

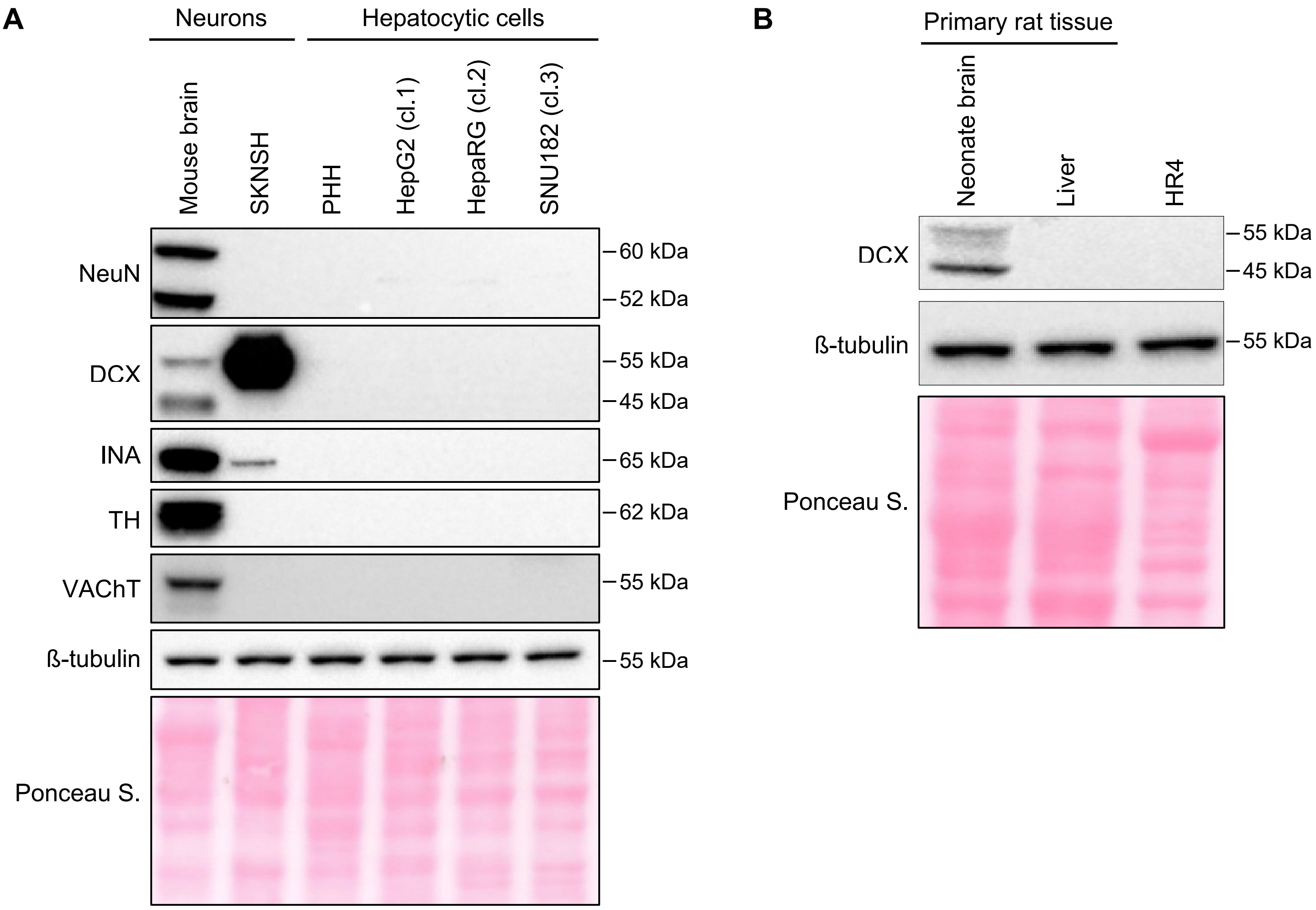


**Fig. S22. Validation of anti-NeuN, DCX, INA, TH and VAChT antibodies by Western Blot.** (**A**) Extracts from neonate mouse brain, SKNSH, PHH, and human HCC lines belonging to transcriptomic classes 1, 2 and 3 were processed for detection of the indicated targets. (**B**) The same strategy was used for the validation of an anti-DCX antibody specifically suitable for rat epitopes. DCX, doublecortin; INA, internexin neuronal intermediate filament protein alpha; NeuN, neuronal nuclear antigen; PHH, primary human hepatocytes; TH, tyrosine hydroxylase; VAChT, vesicular acetylcholine transporter (*SLC18A3*).

## SUPPLEMENTARY INFORMATION 2

**Neurogenesis of cholinergic orientation in a cirrhosis-associated HCC rat model**

HCC occurs in a cirrhotic background in 80% of cases. A rat model that reproduces the development of HCC from cirrhosis, via DEN treatment, has been extensively characterized [16] (also in **Fig. S3**) and shows documented clinical relevance in particular with respect to the proliferative class of HCC [17]. In this context, we herein evaluated the ability of this DEN-treated HCC rat model to recapitulate such neural, cancer-related, processes [18-22]. The methodology used for the experimental induction of HCC is shown in **Fig. S3A**. Total NeuN signals increased throughout disease progression. Interestingly, signals related to the DCX progenitor marker increased transiently, yet sharply, in samples harboring cirrhosis and small HCC nodules (**Fig. S3B** and **Fig. S4A**). In the case of autonomic nervous system (ANS)-specific markers, as in clinical samples, an increase in the cholinergic VAChT marker was observed in rats suffering from HCC. Unlike in humans, levels of the TH marker (adrenergic) remained unaltered throughout disease progression, while being of less functional importance than in the clinic because of microanatomical reasons [23]. Degradation of β-tubulin was correlated with DEN-treatment and was likely derived from hepatic cytolysis and release of cytosolic contents (**Fig. S4A**). Consistently, a tight correlation was observed between DCX expression and β-tubulin degradation throughout progression of HCC-predisposing chronic liver disease (CLD) (**Fig. S4B**). This suggests that HCC neural remodeling occurs as a consequence of cytolysis or parenchymal remodeling, as recently demonstrated in steato-hepatitis [24]. This prompted us to analyze the quantitative evolution of neural markers at different stages of liver injury: fibrosis, cirrhosis and HCC. The results confirmed neo-neurogenesis, and the cholinergic neural features of HCC in the rat (**Fig. S4C-F**). The ANS oscillates between two opposite polarities, prompting for the use of a unified immunoreactive score. Accordingly, as for human samples, the neuronal score (NS) was defined as the difference between adrenergic and cholinergic signals (see Methods, NS = TH - VAChT). The NS provides integrated estimation of ANS inputs to the liver, and decreased with disease progression (**Fig. S4G**), indicating evolution towards cholinergic functions. Interestingly, netrin-1, a neurogenic protein which is frequently upregulated in CLD [25-27], was sharply induced with disease progression in this model and was correlated with β-tubulin degradation and DCX induction (**Fig. S5**). Altogether, such data indicate that alteration of hepatic neural features and their cholinergic orientation is a novel signature of progression towards HCC *in vivo* in the rat model.

## SUPPLEMENTARY INFORMATION 3

**Identification of intra-hepatic neural cells by snRNA-seq**

In order to corroborate Western blot data with approaches combining high resolution and sensitivity, we tested by snRNA-seq the presence of immature and mature neuronal markers in the diseased liver. **Fig. S23** shows the average expression levels and percentage of cells expressing neuronal markers in each hepatic population.

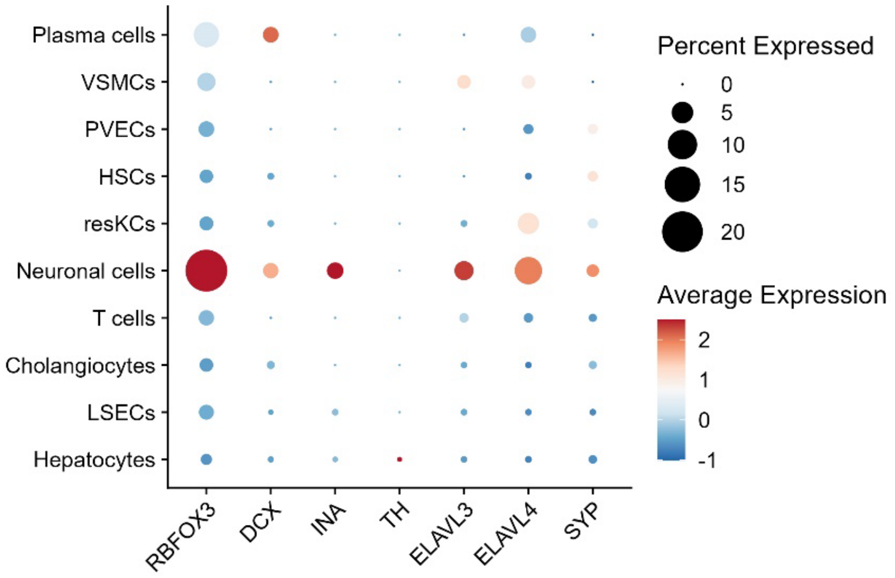


**Fig. S23. Average expression levels and percentage of cells expressing neuronal markers in each hepatic population.** Identified cell types following the analysis of liver snRNA-seq data from MASLD patients (n=2) and healthy individuals (n=2) (GSE174748) [28]. Interestingly, most markers of interest for general neuron identification were found, namely: *RBFOX3* (NeuN, mature neuron), *DCX* and *INA* (immature neuron), *TH* (sympathetic neuron, very low level as expected), *ELAVL3/4* (Huc proteins, immature neuron) and *SYP* (mature neuron/neuroendocrine cells). HSCs, hepatic stellate cells; MASLD, metabolic dysfunction-associated steatotic liver disease; LSECs, liver sinusoidal endothelial cells; PVECs, portal vein endothelial cells; resKCs, resident Kupffer cells; VSMCs, vascular smooth muscle cells.

Using these markers, the number of neuronal cells was estimated at around 5% in the Dimplot shown below (**Fig. S24**). This percentage is comparable to that of cholangiocytes, resident Kupffer cells and hepatic stellate cells. In that sense, the odds of irrelevant detection of neural cells in this setting are not greater than for these three traditionally-monitored liver resident cell

types. This population individualizes well in a uniform manifold approximation and projection (UMAP) plot (**Fig. S24**).

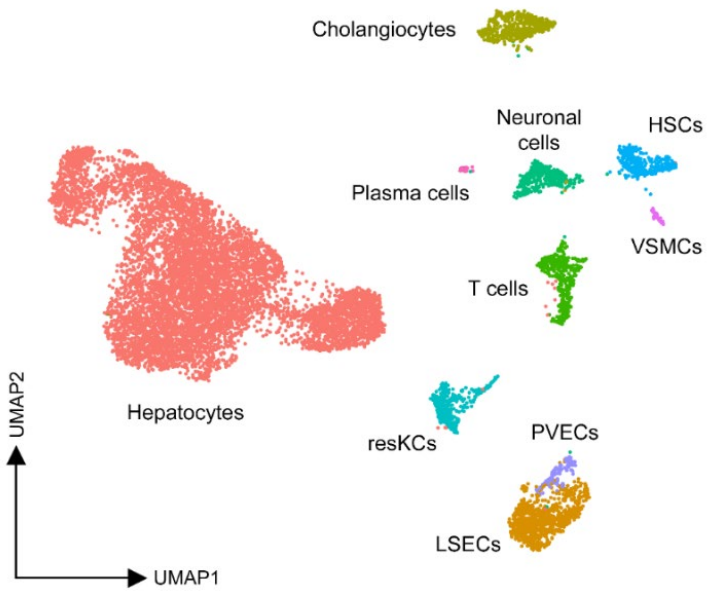


**Fig. S24. UMAP plot of 10 relevant liver cell types.** Populations identified following the analysis of liver snRNA-seq data from MASLD (n=2) patients and healthy individuals (n=2) (GSE174748) [28]. HSCs, hepatic stellate cells; MASLD, metabolic dysfunction-associated steatotic liver disease; LSECs, liver sinusoidal endothelial cells; PVECs, portal vein endothelial cells; resKCs, resident Kupffer cells; UMAP, uniform manifold approximation and projection; VSMCs, vascular smooth muscle cells.

These data compared neurons with several other liver-located cell types. None of the latter express such neural markers at appreciable levels, except in a minority of plasma cells for *DCX*. This work has been done in addition to immunohistochemistry (IHC) data, that shows coherent overlapping signals by several neural markers, *i.e.*, co-staining of the same structures by NeuN, DCX and VAChT antibodies, in the absence of TH signal, as was the case at the WB level in human and rat samples (**Fig. 1** and **Fig. S4**). The morphology of plasma cells being readily distinguishable from that of neurons, further supports the notion that the intrahepatic neural pool has genuinely been detected.

In order to further challenge these findings, we analyzed the potential selective enrichment of the previously identified neural candidate pool when searching for 'Reactome' genesets

associated with neural functions. Previously identified hepatic neural cells were enriched in several canonical cholinergic functions (**Fig. S25**).

Hence, we consider that the odds of falsely assigning non-neural cells as neural cells are limited at the level of this study.

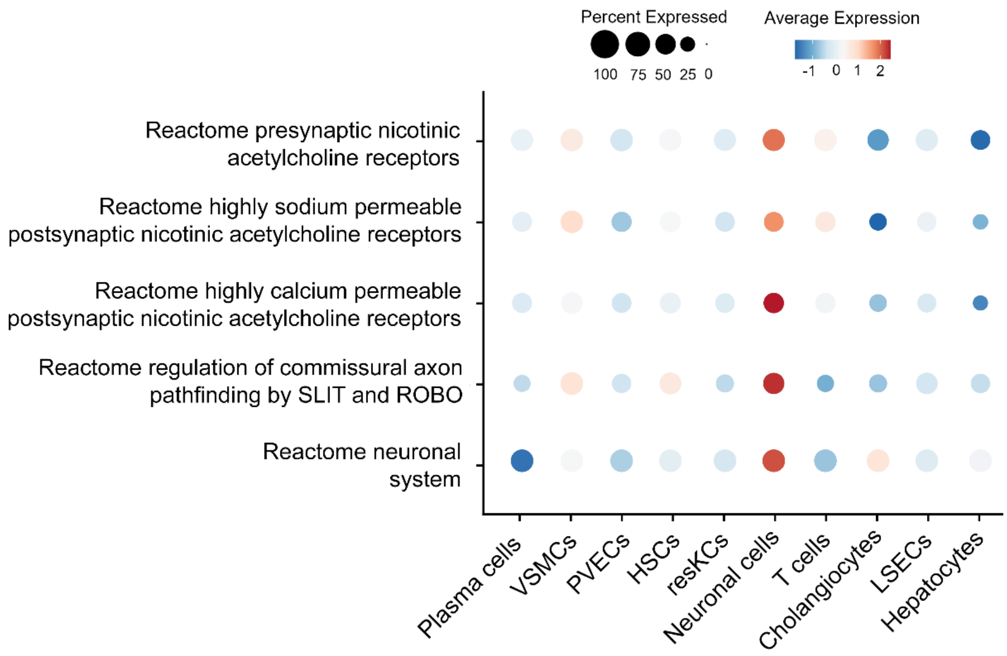


**Fig. S25. Average expression levels and percentage of cells expressing signatures associated with neural function.** Scores in each of the 10 relevant liver cell types identified following the analysis of liver snRNA-seq data from MASLD patients (n=2) and healthy individuals (n=2) (GSE174748). HSCs, hepatic stellate cells; MASLD, metabolic dysfunction-associated steatotic liver disease; LSECs, liver sinusoidal endothelial cells; PVECs, portal vein endothelial cells; resKCs, resident Kupffer cells; VSMCs, vascular smooth muscle cells.

**SUPPLEMENTARY INFORMATION 4**

**Additional arguments for specific consideration of the CHRM3 receptor**

RNA and protein data were considered. First, two independent datasets of paired HCC and non-tumor tissues were used for investigations at the RNA level (*i.e.*, GSE64041 and GSE124535).

In GSE64041, amongst all human cholinergic receptors (*CHRNA1* to *10* - *CHRNA8* being not expressed in humans, *CHRNB1* to *4*, *CHRNE-G*, and *CHRM1* to *5*), *CHRNA5* and *7* were the only ones to be significantly upregulated in paired comparisons with respect to non-tumoral tissues (see below), aside from *CHRM3* that was documented as HCC-induced in all tested datasets in the main body of the paper.

In GSE124535, *CHRNA1*, *2*, *5* and *7* were the only ones to be significantly up-regulated in paired comparisons with respect to non-tumoral tissues (see below), aside from *CHRM3* that was as said documented in the initial version of the paper.

Taking into consideration both cohorts, *CHRNA5* and *7* were repeatedly up-regulated across these datasets. Of note, differing from muscarinic receptors, nicotinic receptors are made of combinatory associations of several alpha and beta subunits, increasing their functional geometric diversity [29], and making their activity in the epithelia challenging to characterize, though they likely assemble in an organ-specific manner [29]. Specifically, we have not been able to identify *CHRNA5* and/or *7*-specific molecules with inverse agonist or antagonist activities in a controlled context. The identity of the beta chain partners of these alpha chains remains uncharted in the liver. Quantification data are below.

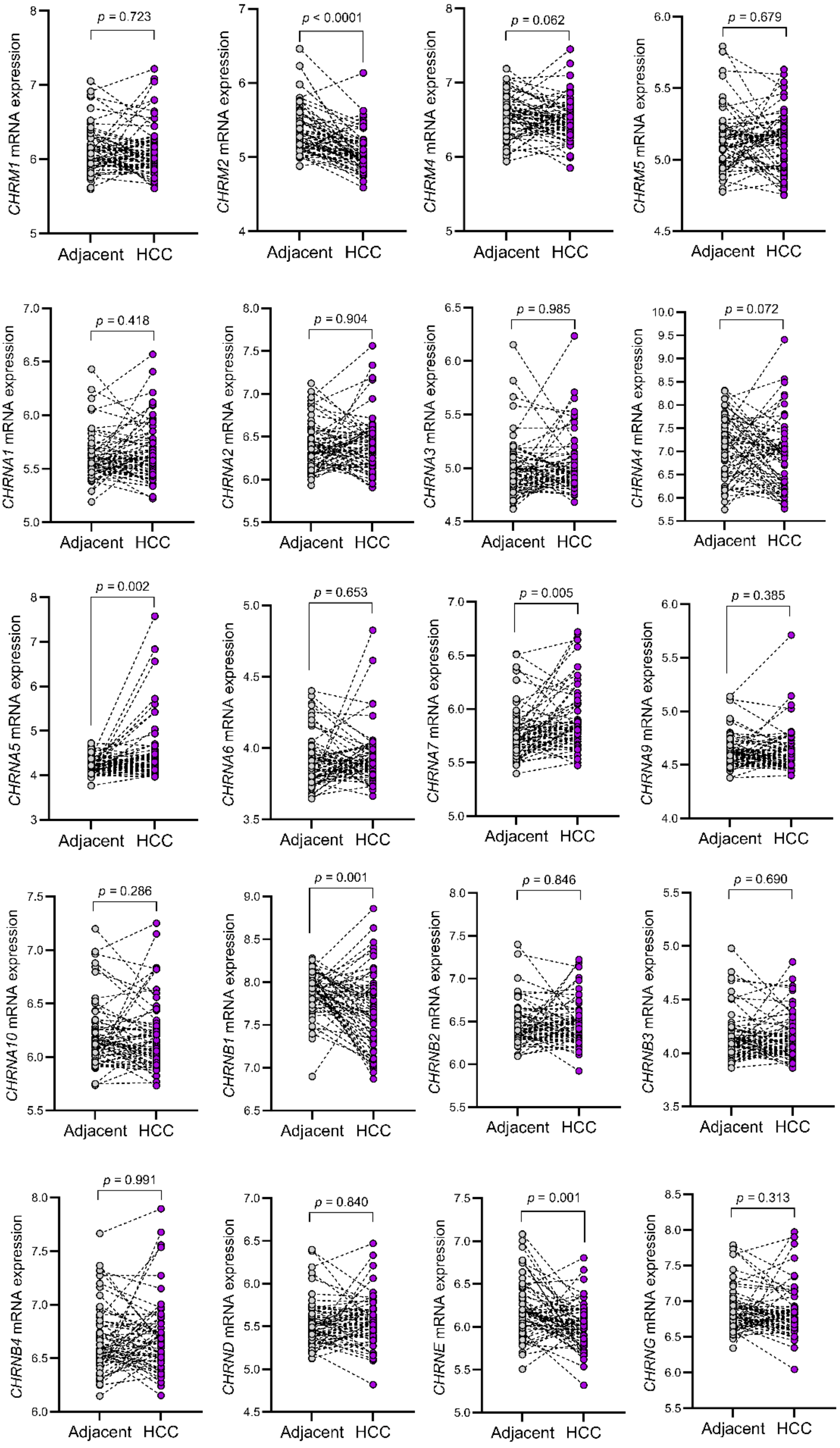


**Fig. S26. Comparison of the expression of neuronal receptors between adjacent and HCC tumor tissues.** Wilcoxon matched-pairs signed rank test, n=60 (GSE64041).

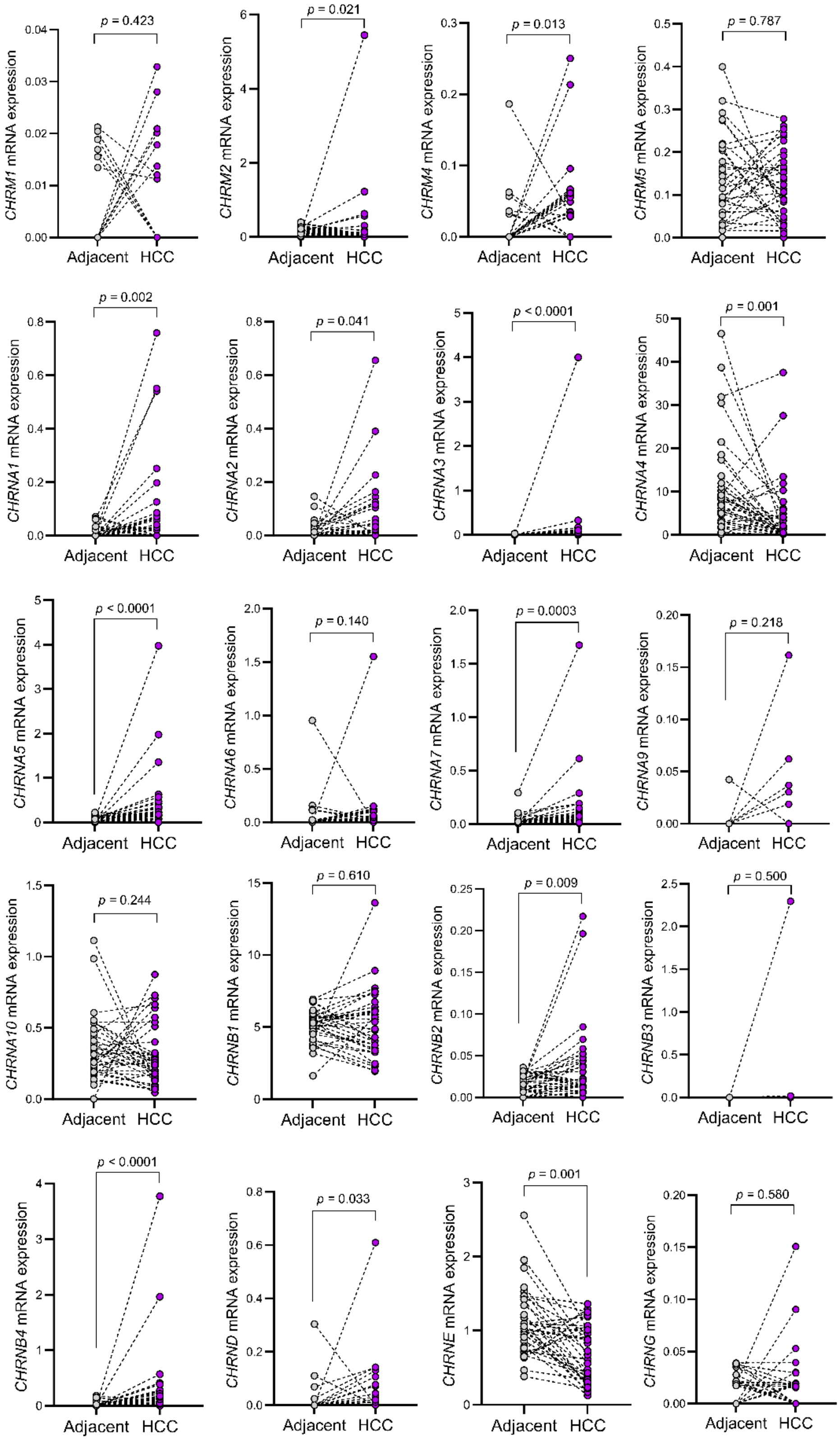


**Fig. S27. Comparison of the expression of neuronal receptors between adjacent and HCC tumor tissues.** Wilcoxon matched-pairs signed rank test, n=35 (GSE124535).

Regulation data are summarized in **Table S14** below.

**Table S14. Regulation data of cholinergic receptors in the GSE64041 and GSE124535 datasets, as non-tumoral / tumoral pairs**. Paired Wilcoxon test. Cut-off of 0.05. Ns, non-significant.

| | p-values (tumoral over non tumoral comparison) | | |
|---|---|---|---|
| | GSE64041 | GSE124535 | Commonly induced targets |
| *CHRNA1* | ns | 0.002 (induction) | |
| *CHRNA2* | ns | 0.04 (induction) | |
| *CHRNA3* | ns | <0.0001 (induction) | |
| *CHRNA4* | ns | 0.001 (repression) | |
| *CHRNA5* | 0.002 (induction) | <0.0001 (induction) | *CHRNA5* |
| *CHRNA6* | ns | ns | |
| *CHRNA7* | 0.005 (induction) | 0.0003 (induction) | *CHRNA7* |
| *CHRNA8* | Unexpressed in humans | Unexpressed in humans | |
| *CHRNA9* | ns | ns | |
| *CHRNA10* | ns | ns | |
| *CHRNB1* | 0.001 (repression) | 0.001 (repression) | |
| *CHRNB2* | ns | 0.009 (induction) | |
| *CHRNB3* | ns | ns | |
| *CHRNB4* | ns | <0.0001 (induction) | |
| *CHRND* | ns | 0.03 (induction) | |
| *CHRNE* | ns | 0.001 (repression) | |
| *CHRNG* | ns | ns | |
| | | | |
| *CHRM1* | ns | 0.021 (repression) | |
| *CHRM2* | <0.0001 (repression) | <0.0001 (repression) | |
| *CHRM3* | | | |
| *CHRM4* | ns | 0.013 (induction) | |
| *CHRM5* | ns | ns | |

We then explored these notions at the protein level, using the ProteinAtlas database that proposes HCC protein levels generated with antibodies benchmarked after technical validation by IHC. Protein expression data were available for *CHRNA1, 3, 4, 5* and *7* nicotinic receptors and for *CHRM1* to *5* muscarinic receptors. RNA data gathered on *CHRNA5* and *7* were likely not confirmed at the protein level (n=10 cases analyzed by IHC), while, again *CHRM3* expression was sharply induced at the protein level (n=11 cases). See **Fig. S28** below.

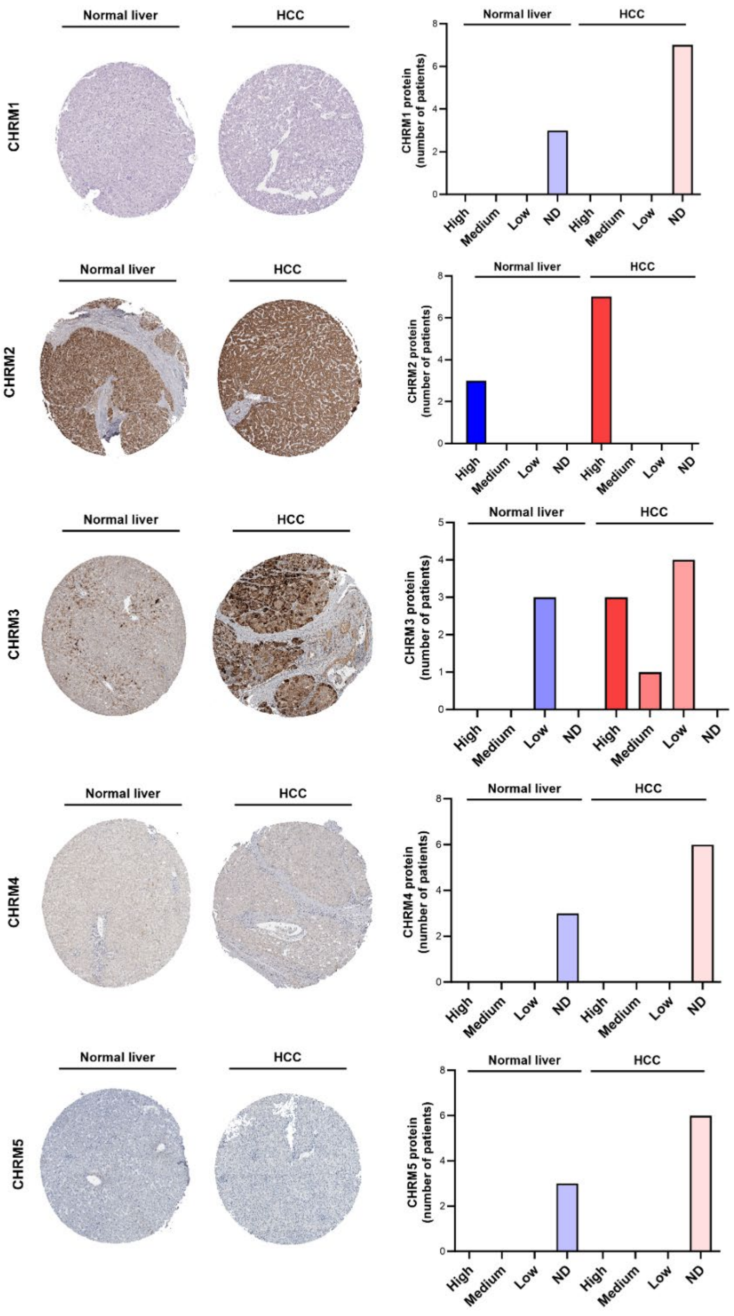

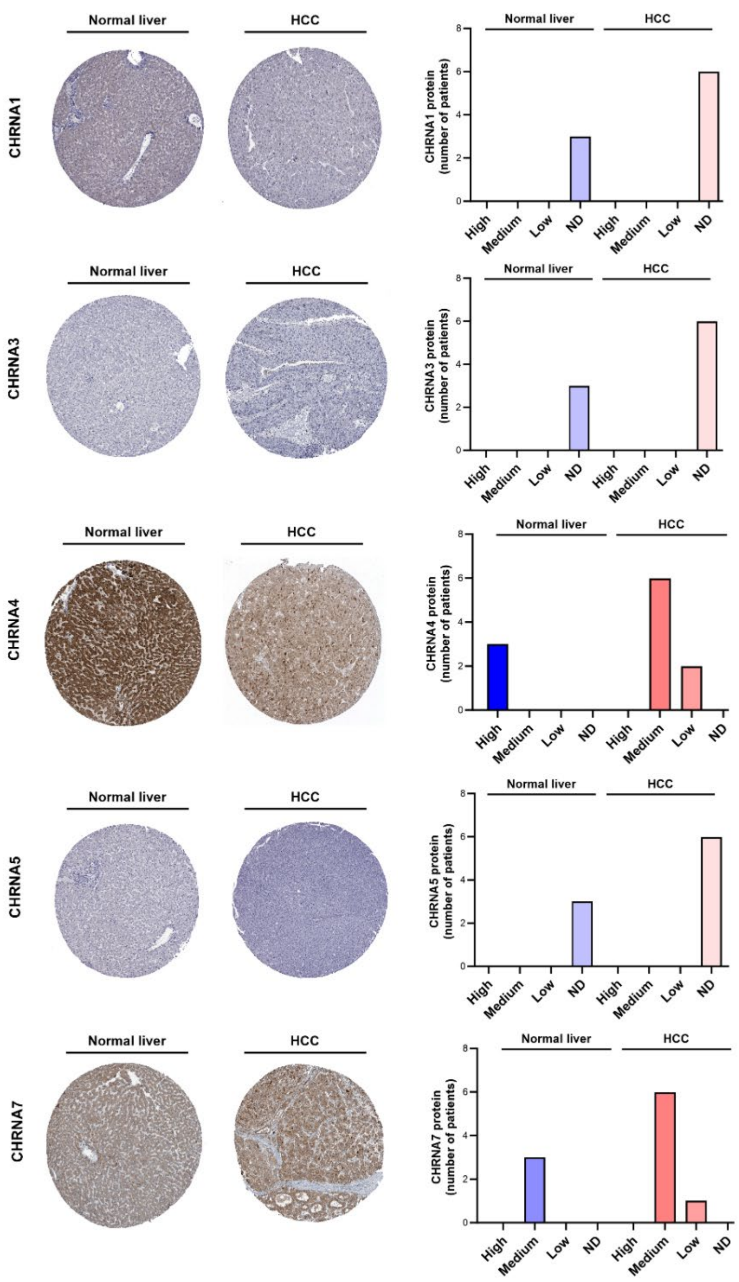


**Fig. S28. Cholinergic receptor protein expression data retrieved from the ProteinAtlas database.** n=10 to 11 cases.

We also explored ProteinAtlas data with respect to CHRM3 frequency of staining across cancer types. HCC ranks 2nd out of 20 cancer types in the ProteinAtlas database with respect to frequency of staining of this antigen using a validated antibody, see https://www.proteinatlas.org/ENSG00000133019-CHRM3/pathology in a context where hepatocytes themselves bear the signal (see 2D matrix of the following link): https://www.proteinatlas.org/ENSG00000133019-CHRM3/single+cell+type/liver. Such data

suggest that, in patients, HCC may more often rely on CHRM3 than other cancer types for its onset or development.

Interestingly, in the BIOSTORM cohort [15], only hepatocytic phosphorylated extracellular signal-regulated kinase (pERK) and microvascular invasion, as elsewhere [30], predicted poor recurrence-free survival after sorafenib. This is of interest with respect to the known ability of CHRM3 to activate ERK [31], and to be expressed by portal vein endothelial cells (PVECs) as a likely important cell type cytologically defining affected sites of microvascular invasion (see scRNA-seq data below).

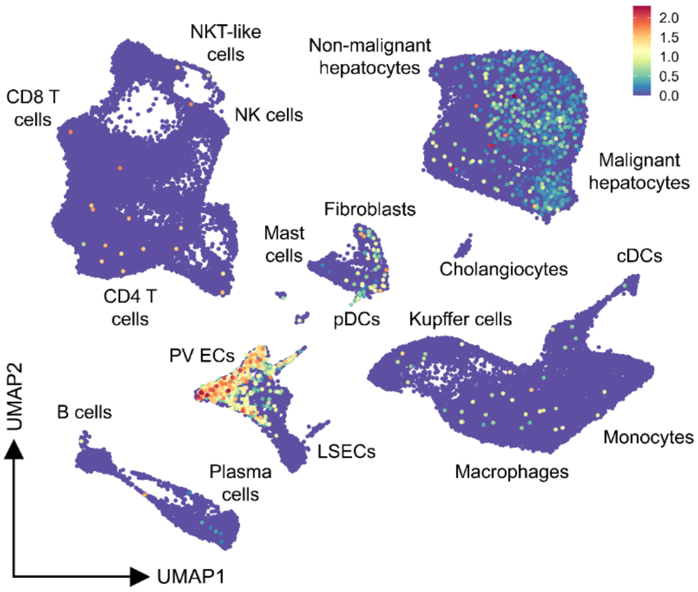


**Fig. S29. *CHRM3* expression level in each cell type represented as UMAP in HCC samples.** Data obtained from GSE149614 (n=10). cDCs, classical dendritic cells; CHRM3, cholinergic receptor muscarinic 3; NK, natural killer cells; LSECs, liver sinusoidal endothelial cells; PVECs, portal vein endothelial cells; pDCs, plasmacytoid dendritic cells; UMAP, uniform manifold approximation and projection.

Altogether, because of (i) the evidence for concordant increases from non-tumoral to tumoral tissues restricted to CHRM3 amongst all cholinergic receptors, both at the RNA and protein levels, (ii) the frequency of CHRM3-positive staining in HCC versus 20 other cancer types, (iii) the absence of robust structural features for selective targeting of any other induced receptor, and (iv) the association of CHRM3 with adverse markers of sensitivity to sorafenib and of poor survival (activation of Erk and microvascular invasion) in the literature, CHRM3 was selected as the most adequate cholinergic protein for subsequent HCC targeting in the study.

## SUPPLEMENTARY INFORMATION 5

**Engagement of the CHRM3 receptor impacts phosphorylation of EGFR, STAT3, MAPK and YAP pathways markers.**

HCC lines or interest and PHH were treated with darifenacin or cevimeline as agonist or antagonist of the CHRM3 receptor. Canonical pathways of interest in HCC pathology and susceptible to be functionally linked to CHRM3 [31, 32] were then probed considering their main respective markers.

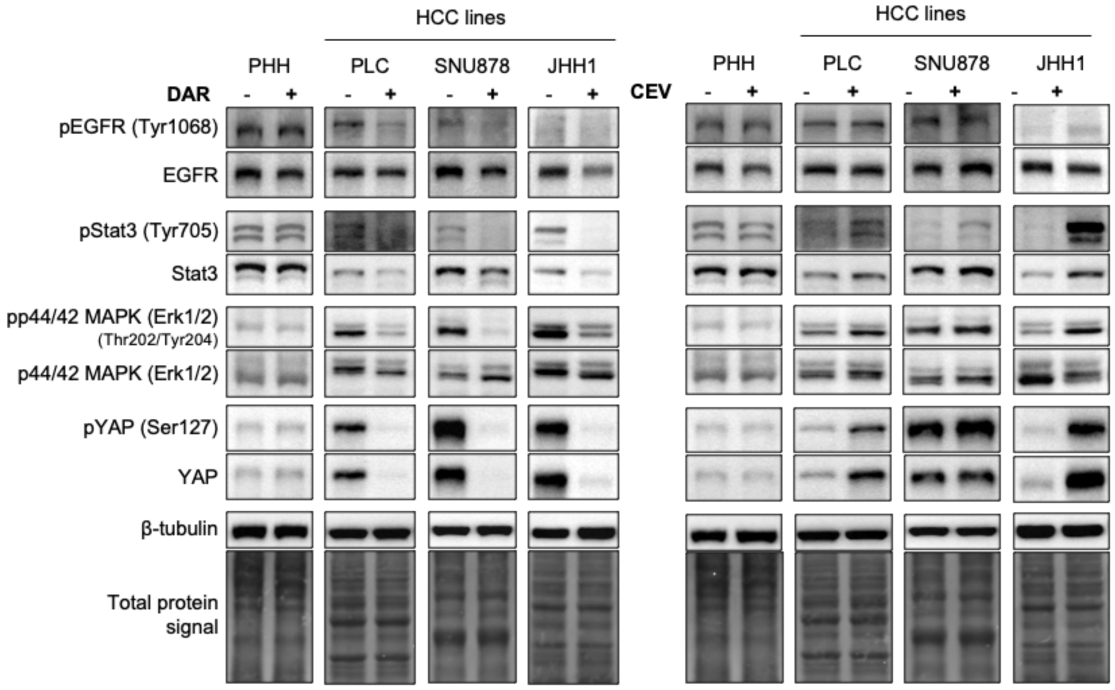


**Fig. S30. CHRM3 engagement modulates EGFR, STAT3, MAPK and YAP in class 1, 2, and 3 human HCC lines following treatment with antagonist darifenacin (A) or agonist cevimeline (B).** Incubation times vary from 4 to 48 h depending on cell line. PHH were used as non-cancer cell controls (n=3). EGFR, epidermal growth factor receptor; ERK, extracellular signal-regulated kinase; MAPK, mitogen-activated protein kinase; PHH, primary human hepatocytes; STAT3, signal transducer and activator of transcription 3; YAP, yes1 associated transcriptional regulator.

**SUPPLEMENTARY INFORMATION 6**

**Applicability of the NRS to other hepatic malignancies**

Using the same computational strategy as for all previous work in this study, we calculated the NRS values pertaining to cholangiocarcinoma (CCA, also named ICC, see below) in three publicly available bioinformatics datasets related to these conditions. Control tissues were paired adjacent tissues or normal livers. **Fig. S31** data below indicate that, as in HCC, lower NRS values characterize tumor tissues in these three malignancies.

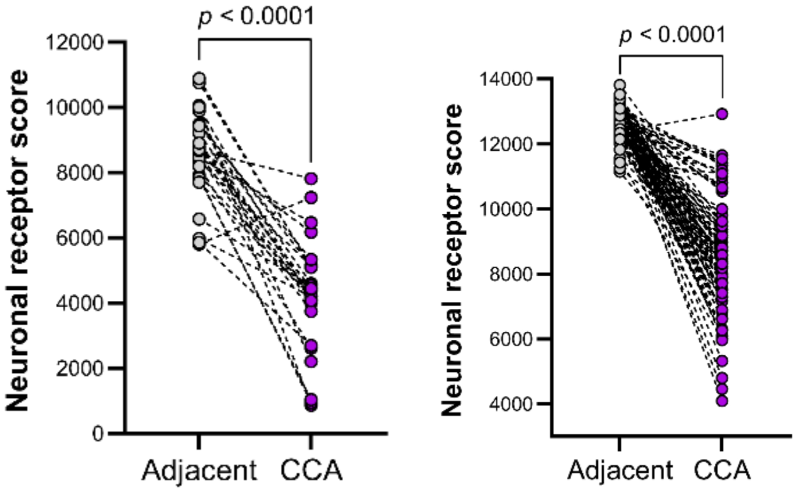


**Fig. S31. Comparison of neuronal receptor scores between adjacent and CCA tumor tissues.** From left to right: GSE107943, n=27 [33]; GSE76297, n=90 [34] ; Wilcoxon matched-pairs signed rank test. NRS values were calculated as depicted in the *Materials and Methods* section. CCA, cholangiocarcinoma.

Of note, such cholinergic orientation was confirmed in another comparative study that considered normal livers as controls (**Fig. S32**).

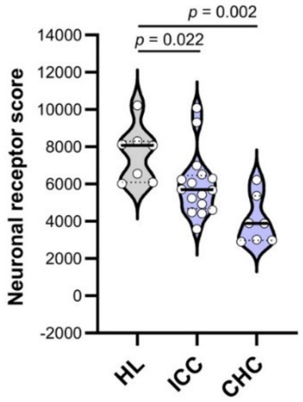


**Fig. S32. Comparison of neuronal receptor scores across types of liver cancer (GSE32879).** Samples include HL (n=7), ICC (n=16) and CHC (n=7). Mann-Whitney test versus HL. CHC, combined hepatocellular cholangiocarcinoma; HL, healthy liver; ICC, intrahepatic cholangiocarcinoma.

We then wondered whether *CHRM3* was also regulated in all these etiologies. **Fig. S33** below indicates that this potential target was up-regulated across all these types of cancers.

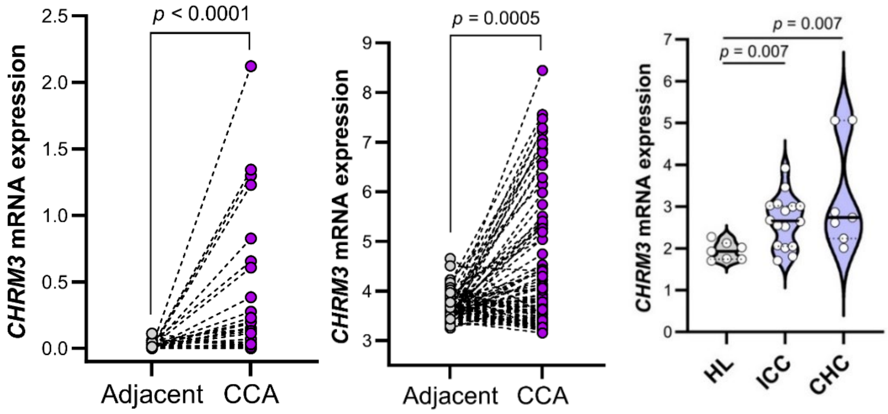


**Fig. S33. Comparison of *CHRM3* mRNA expression between adjacent and CCA tumor tissues**. Three panels above: Wilcoxon matched-pairs signed rank test, n=27 (GSE107943), between adjacent and CCA tumor tissues. Wilcoxon matched-pairs signed rank test, n=90 (GSE76297), between adjacent and CCA tumor tissues. Wilcoxon matched-pairs signed rank test, n=18 (GSE132037), and across liver disease etiologies (GSE32879). To the right, this last group of samples include HL (n=7), ICC (n=16) and CHC (n=7). CCA, cholangiocarcinoma; CHC, combined hepatocellular cholangiocarcinoma; CHRM3, cholinergic receptor muscarinic 3; HL, healthy liver; ICC, intrahepatic cholangiocarcinoma.

Altogether, these data indicate that a cholinergic oriented phenotype characterizes cancer lesions of the liver, with CHRM3 being a potential target for further investigations in CCA as well.

## SUPPLEMENTARY INFORMATION 7

**Transduction considerations related to targeted sub-groups of muscarinic receptors**

Scopolamine is a pan-muscarinic antagonist whereas darifenacin is a M3 selective antagonist. The family of muscarinic acetylcholine receptors encompasses five members in mammals, encoded by the *CHRM1*-5 genes. These are G-protein-coupled receptors, which can be divided into the following two subfamilies: CHRM1, CHRM3, and CHRM5 receptors coupling to $G_{q/11}$; and CHRM2 and CHRM4 receptors coupling to $G_{i/o}$. Activation of CHRM1, CHRM3 and CHRM5 results in phospholipid turnover and changes in cell calcium concentration. Activation of CHRM2 and CHRM4 results in inhibition of adenyl cyclase and reduced levels of cAMP [35, 36]. Of these five muscarinic receptor subtypes, those that activate phospholipid turnover (CHRM1, 3, 5) are conditional oncogenes when expressed in cells capable of proliferation [36].

Anchorage-independent growth assays described herein select colonies according to their ability to withstand stress related to the likely absence of integrin signaling, while this study's pharmacological assays challenge cells on their ability to shift towards dependence to CHRM3 signaling for survival upon inhibition of cellular kinases by sorafenib. The fact that scopolamine becomes active in the latter setting suggests that both phosphoinositides and cAMP participate to survival in these conditions, while phosphoinositides per se are instrumental for survival upon deprivation of integrin signaling, as reviewed [37]. Alternatively, at least in the context of anchorage-independent growth, this could also mean that pan-muscarinic inhibition by scopolamine may hinder the benefits of CHRM1-3-5 inhibition, expected to limit survival, through concomitant engagement of the likely anticancer M2-4 receptors. Given the complexity of processes with respect to this question, this issue will be further explored in a subsequent study.

## SUPPLEMENTARY INFORMATION 8

**Quantification of neuronal receptors and NRS levels in immune population present in the HCC microenvironment**

In order to gain further insights related to the potential of intrahepatic immune populations as targets for neural signals, we analyzed publicly available scRNA-seq data derived from HCC tissues (n=38) [38]. In this dataset constituted of lymphoid and myeloid cell types (**Fig. S34A, B**), quantification of individual neuronal receptors allowed us to observe that *ADRB2*, *CHRNB1* and *CHRNE* present the highest expression (**Fig. S34C**). Moreover, quantification of the NRS showed that CD8 TEMRA and cytotoxic NK cells are the sub-populations with the strongest adrenergic polarization (**Fig. S34D**). These observations are in line with previous reports describing how cholinergic signaling dampens CD8 T cell activity [39], as illustrated by the markedly lower NRS levels in exhausted CD8 populations. Similar results were obtained by re-clustering the lymphoid compartment from the previously described GSE149614 dataset (**Fig. S35**). Nonetheless, it is worth noting that in the particular case of *CHRM3*, malignant hepatocytes present higher expression levels as compared to immune populations (**Fig. S29**).

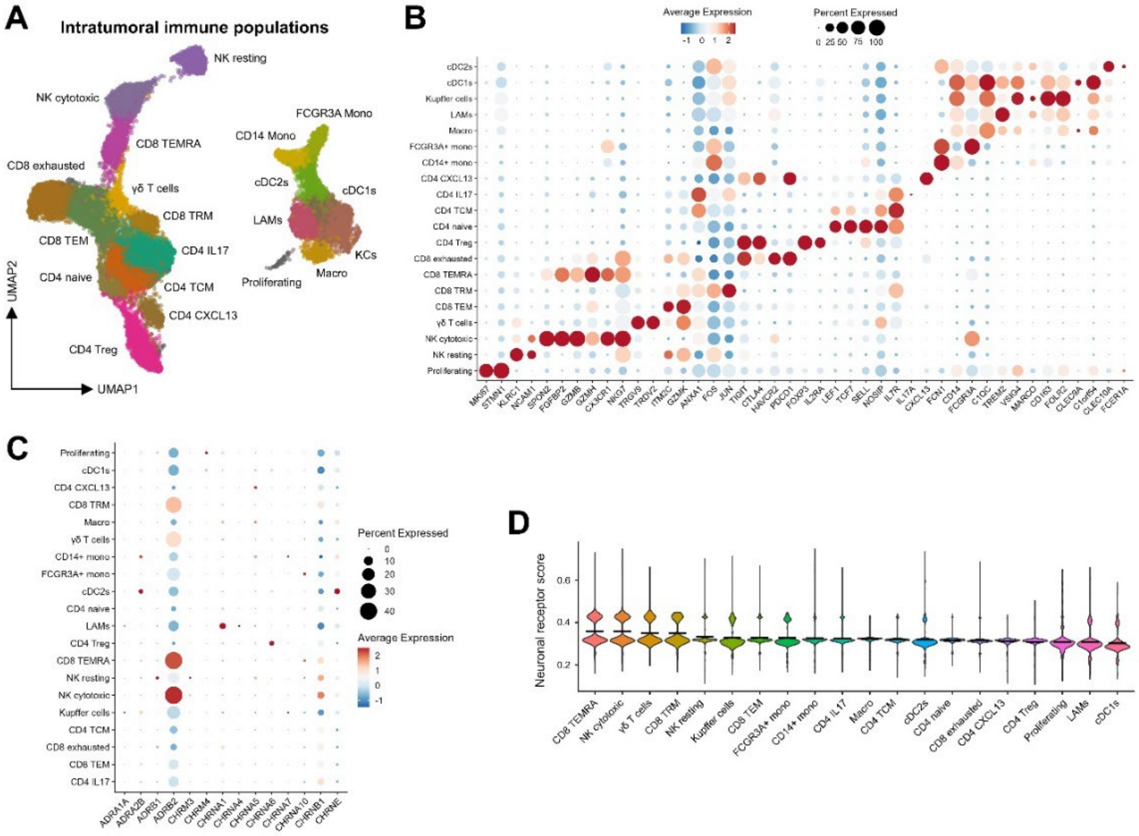

**Fig. S34. Expression of individual neuronal receptors and the NRS in each immune cell type found in HCC samples.** (**A**) UMAP plot of 20 cell populations identified following analysis of scRNA-seq data from 38 HCC patients [38]. (**B**) Dotplot showing the average expression levels and percentage of cells expressing canonical cell type markers in each of the 20 immune cell types. (**C**) Dotplot showing the average expression levels and percentage of cells expressing individual neuronal receptors in each of the identified cell types. (**D**) GSVA scores for the neuronal receptor signature in each cell type, represented as UMAP (top) and violin plot (bottom). cDCs, classical dendritic cells; KCs, Kupffer cells; NK, natural killer; NRS, neuronal receptor score; LAMs, lipid-associated macrophages; TCM, T central memory; TEM, T effector memory; TEMRA, T effector memory RA; Treg, regulatory T cells; TRM, tissue resident memory; UMAP, uniform manifold approximation and projection.

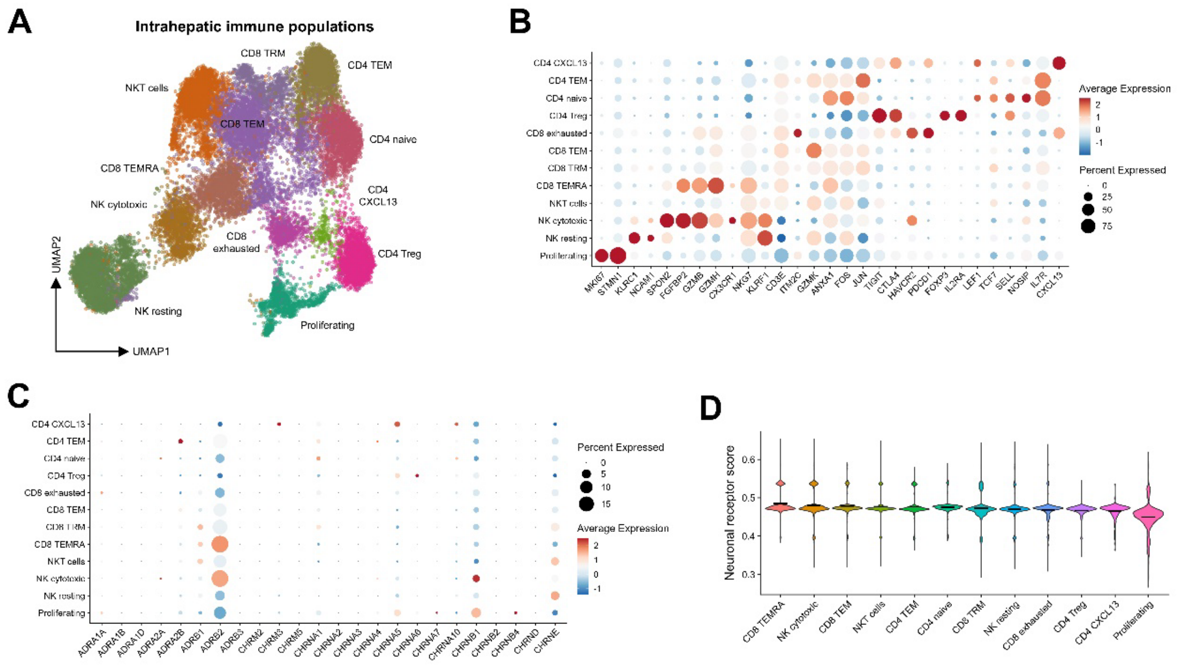


**Fig. S35. Expression of individual neuronal receptors and the NRS in each immune cell type found in HCC samples.** (**A**) UMAP plot showing 12 lymphoid sub-populations identified following re-analysis of scRNA-seq data from 10 patients (GSE149614). (**B**) Dotplot showing the average expression levels and percentage of cells expressing canonical cell type markers in each of the 20 immune cell types. (**C**) Dotplot showing the average expression levels and percentage of cells expressing individual neuronal receptors in each of the identified cell types. (**D**) GSVA scores for the neuronal receptor signature in each cell type, represented as UMAP (top) and violin plot (bottom). cDCs, classical dendritic cells; KCs, Kupffer cells; NK, natural

killer; NRS, neuronal receptor score; LAMs, lipid-associated macrophages; TCM, T central memory; TEM, T effector memory; TEMRA, T effector memory RA; Treg, regulatory T cells; TRM, tissue resident memory; UMAP, uniform manifold approximation and projection.

## SUPPLEMENTARY REFERENCES

[1] Gripon P, Rumin S, Urban S, et al. Infection of a human hepatoma cell line by hepatitis B virus. Proceedings of the National Academy of Sciences of the United States of America 2002;99:15655-15660

[2] Zheng S, Wang W, Aldahdooh J, et al. SynergyFinder Plus: Toward Better Interpretation and Annotation of Drug Combination Screening Datasets. Genomics Proteomics Bioinformatics 2022;20:587-596

[3] Horiuchi S, Kuroda Y, Oyafuso R, et al. Construction of a culture protocol for functional bile canaliculi formation to apply human iPS cell-derived hepatocytes for cholestasis evaluation. Sci Rep 2022;12:15192

[4] Satija R, Farrell JA, Gennert D, et al. Spatial reconstruction of single-cell gene expression data. Nat Biotechnol 2015;33:495-502

[5] Hanzelmann S, Castelo R, Guinney J. GSVA: gene set variation analysis for microarray and RNA-seq data. BMC Bioinformatics 2013;14:7

[6] Miranda A, Hamilton PT, Zhang AW, et al. Cancer stemness, intratumoral heterogeneity, and immune response across cancers. Proc Natl Acad Sci U S A 2019;116:9020-9029

[7] Guilliams M, Bonnardel J, Haest B, Vanderborght B, et al. Spatial proteogenomics reveals distinct and evolutionarily conserved hepatic macrophage niches. Cell 2022;185:379-396 e338

[8] Ragnum HB, Vlatkovic L, Lie AK, et al. The tumour hypoxia marker pimonidazole reflects a transcriptional programme associated with aggressive prostate cancer. Br J Cancer 2015;112:382-390

[9] Buffa FM, Harris AL, West CM, et al. Large meta-analysis of multiple cancers reveals a common, compact and highly prognostic hypoxia metagene. Br J Cancer 2010;102:428-435

[10] Winter SC, Buffa FM, Silva P, et al. Relation of a hypoxia metagene derived from head and neck cancer to prognosis of multiple cancers. Cancer Res 2007;67:3441-3449

[11] Liu J, Lichtenberg T, Hoadley KA, et al. An Integrated TCGA Pan-Cancer Clinical Data Resource to Drive High-Quality Survival Outcome Analytics. Cell 2018;173:400-416 e411

[12] Therneau T. A Package for Survival Analysis in R. R package 2021 [cited version 3.2-13; Available from: https://CRAN.R-project.org/package=survival

[13] Therneau TM GP. Modeling Survival Data: Extending the Cox Model. Springer, New-York 2000

[14] Kassambara AK, M.; Biecek, P. Survminer: Drawing Survival Curves using 'ggplot2'. R package Version 0.4.9

[15] Pinyol R, Montal R, Bassaganyas L, et al. Molecular predictors of prevention of recurrence in HCC with sorafenib as adjuvant treatment and prognostic factors in the phase 3 STORM trial. Gut 2019;68:1065-1075

[16] Roth GS, Macek Jilkova Z, Zeybek Kuyucu A, et al. Efficacy of AKT Inhibitor ARQ 092 Compared with Sorafenib in a Cirrhotic Rat Model with Hepatocellular Carcinoma. Mol Cancer Ther 2017;16:2157-2165

[17] Kurma K, Manches O, Chuffart F, et al. DEN-Induced Rat Model Reproduces Key Features of Human Hepatocellular Carcinoma. Cancers (Basel) 2021;13

[18] Thaker PH, Han LY, Kamat AA, et al. Chronic stress promotes tumor growth and angiogenesis in a mouse model of ovarian carcinoma. Nat Med 2006;12:939-944

[19] Magnon C, Hall SJ, Lin J, et al. Autonomic nerve development contributes to prostate cancer progression. Science 2013;341:1236361

[20] Hayakawa Y, Sakitani K, Konishi M, et al. Nerve Growth Factor Promotes Gastric Tumorigenesis through Aberrant Cholinergic Signaling. Cancer Cell 2017;31:21-34

[21] Renz BW, Takahashi R, Tanaka T, et al. beta2 Adrenergic-Neurotrophin Feedforward Loop Promotes Pancreatic Cancer. Cancer Cell 2018;33:75-90 e77

[22] Renz BW, Tanaka T, Sunagawa M, et al. Cholinergic Signaling via Muscarinic Receptors Directly and Indirectly Suppresses Pancreatic Tumorigenesis and Cancer Stemness. Cancer Discov 2018;8:1458-1473

[23] Jensen KJ, Alpini G, Glaser S. Hepatic nervous system and neurobiology of the liver. Compr Physiol 2013;3:655-665

[24] Adori C, Daraio T, Kuiper R, et al. Disorganization and degeneration of liver sympathetic innervations in nonalcoholic fatty liver disease revealed by 3D imaging. Sci Adv 2021;7

[25] Barnault R, Verzeroli C, Fournier C, et al. Hepatic inflammation elicits production of proinflammatory netrin-1 through exclusive activation of translation. Hepatology 2022;76:1345-1359

[26] Lahlali T, Plissonnier ML, Romero-Lopez C, et al. Netrin-1 Protects Hepatocytes Against Cell Death Through Sustained Translation During the Unfolded Protein Response. Cell Mol Gastroenterol Hepatol 2016;2:281-301 e289

[27] Plissonnier ML, Lahlali T, Michelet M, et al. Epidermal Growth Factor Receptor-Dependent Mutual Amplification between Netrin-1 and the Hepatitis C Virus. PLoS Biol 2016;14:e1002421

[28] Filliol A, Saito Y, Nair A, et al. Opposing roles of hepatic stellate cell subpopulations in hepatocarcinogenesis. Nature 2022;610:356-365

[29] Albuquerque EX, Pereira EF, Alkondon M, et al. Mammalian nicotinic acetylcholine receptors: from structure to function. Physiol Rev 2009;89:73-120

[30] Erstad DJ, Tanabe KK. Prognostic and Therapeutic Implications of Microvascular Invasion in Hepatocellular Carcinoma. Ann Surg Oncol 2019;26:1474-1493

[31] Felton J, Hu S, Raufman JP. Targeting M3 Muscarinic Receptors for Colon Cancer Therapy. Curr Mol Pharmacol 2018;11:184-190

[32] Guo L, Liu Y, Ding Z, et al. Signal transduction by M3 muscarinic acetylcholine receptor in prostate cancer. Oncol Lett 2016;11:385-392

[33] Ahn KS, O'Brien D, Kang YN, et al. Prognostic subclass of intrahepatic cholangiocarcinoma by integrative molecular-clinical analysis and potential targeted approach. Hepatol Int 2019;13:490-500

[34] Chaisaingmongkol J, Budhu A, Dang H, et al. Common Molecular Subtypes Among Asian Hepatocellular Carcinoma and Cholangiocarcinoma. Cancer Cell 2017;32:57-70 e53

[35] Von Rosenvinge EC, Raufman JP. Muscarinic receptor signaling in colon cancer. Cancers (Basel) 2011;3:971-981

[36] Gutkind JS, Novotny EA, Brann MR, et al. Muscarinic acetylcholine receptor subtypes as agonist-dependent oncogenes. Proc Natl Acad Sci U S A 1991;88:4703-4707

[37] DeMali KA, Wennerberg K, Burridge K. Integrin signaling to the actin cytoskeleton. Curr Opin Cell Biol 2003;15:572-582

[38] Cappuyns S, Philips G, Vandecaveye V, et al. PD-1- CD45RA+ effector-memory CD8 T cells and CXCL10+ macrophages are associated with response to atezolizumab plus bevacizumab in advanced hepatocellular carcinoma. Nat Commun 2023;14(1):7825

[39] Bauer KC, Trehan R, Ruf B, et al. The Gut Microbiome Controls Liver Tumors via the Vagus Nerve. bioRxiv [Preprint] 2024;2024.01.23.576951
